# Transcription factors CEP‐1/p53 and CEH‐23 collaborate with AAK‐2/AMPK to modulate longevity in Caenorhabditis *elegans*.

**DOI:** 10.1111/acel.12619

**Published:** 2017-05-30

**Authors:** Hsin‐Wen Chang, Steve Pisano, Amaresh Chaturbedi, Jennifer Chen, Sarah Gordon, Aiswarya Baruah, Siu Sylvia Lee

**Affiliations:** ^1^ Department of Molecular Biology and Genetics Cornell University Ithaca NY 14853 USA; ^2^ Present address: Department of Agricultural Biotechnology Assam Agricultural University Jorhat 785013 Assam India

**Keywords:** aging, AMPK, *C. elegans*, mitochondrial ETC, p53, transcription

## Abstract

A decline in mitochondrial electron transport chain (ETC) function has long been implicated in aging and various diseases. Recently, moderate mitochondrial ETC dysfunction has been found to prolong lifespan in diverse organisms, suggesting a conserved and complex role of mitochondria in longevity determination. Several nuclear transcription factors have been demonstrated to mediate the lifespan extension effect associated with partial impairment of the ETC, suggesting that compensatory transcriptional response to be crucial. In this study, we showed that the transcription factors CEP‐1/p53 and CEH‐23 act through a similar mechanism to modulate longevity in response to defective ETC in *Caenorhabditis elegans*. Genomewide gene expression profiling comparison revealed a new link between these two transcription factors and AAK‐2/AMP kinase (AMPK) signaling. Further functional analyses suggested that CEP‐1/p53 and CEH‐23 act downstream of AAK‐2/AMPK signaling and CRTC‐1 transcriptional coactivator to promote stress resistance and lifespan. As AAK‐2, CEP‐1, and CEH‐23 are all highly conserved, our findings likely provide important insights for understanding the organismal adaptive response to mitochondrial dysfunction in diverse organisms and will be relevant to aging and pathologies with a mitochondrial etiology in human.

## Introduction

The mitochondrial electron transport chain (ETC) produces the majority of ATP in cells and broadly influences diverse biological processes. Accordingly, perturbations in mitochondrial ETC function are usually detrimental. However, in some cases, moderate reduction of mitochondrial ETC function prolongs organismal longevity in organisms ranging from yeast to mice (Dillin *et al*., 2002; Kirchman *et al*., 1999; Liu *et al*., 2005; Lee *et al*., 2003; Copeland *et al*., 2009). In *Caenorhabditis elegans*, several mutants that harbor point mutations in distinct mitochondrial ETC subunits have been identified. These mutants show altered lifespan, with some living longer, while others living shorter, than wild‐type worms (Wong *et al*.,^1994^; Feng *et al*., 2001; Yang & Hekimi, 2010; Ishii *et al*., 1998; Kayser *et al*., 1999; Hartman *et al*., 2001). Moreover, RNAi knockdown of many different ETC subunits also impact lifespan (Dillin *et al*., 2002; Lee *et al*., 2003), and RNAi dilution experiments clearly demonstrating that the degree of knockdown of a single ETC subunit can determine whether the lifespan becomes extended or shortened (Rea *et al*., 2007). Together, these observations suggest that mitochondria play a crucial and complex role in determining the lifespan of an organism.

Proper communication between mitochondria and the nucleus is essential for organismal survival. In yeast, a retrograde signaling pathway relays signals from mitochondria to the nucleus and is key to the aging phenotypes associated with yeast cells with compromised mitochondrial ETC function (Parikh *et al*., 1987; Jazwinski *et al*., 2013). In *C. elegans*, mitochondrial ETC dysfunction is accompanied by specific changes in nuclear gene expression (Falk *et al*., 2008; Cristina *et al*., 2009; Yee *et al*., 2014), and particular transcription factors have been shown to mediate the altered longevity associated with the various ETC mutants (Lee *et al*., 2010; Khan *et al*., 2013; Walter *et al*., 2011; Ventura *et al*., 2010).

The *C. elegans* homeodomain protein CEH‐23 plays an important role in mediating lifespan extension associated with ETC dysfunction (Walter *et al*., 2011). Inactivation of *ceh‐23* partially suppresses the prolonged lifespan of the *isp‐1(qm150)* mutant without affecting its development and reproduction phenotypes, suggesting that CEH‐23 specifically mediates the longevity of the *isp‐1* mutant (Walter *et al*., 2011). *isp‐1* encodes the Rieske iron sulfur protein, a key subunit of the mitochondrial ETC complex III, and the *isp‐1* mutant harbors a point mutation that reduces the electron transport efficiency of complex III and extends lifespan by up to 50% (Feng *et al*., 2001). Interestingly, loss of *ceh‐23* has no impact on the lifespan of wild‐type animals or long‐lived mutants in the insulin‐like signaling and the *eat‐2* caloric restriction pathways, suggesting that CEH‐23 modulates longevity specifically in response to mitochondrial ETC dysfunction (Walter *et al*., 2011). How CEH‐23 performs this function is unknown. Besides longevity modulation, earlier studies suggested a role for CEH‐23 in neuronal differentiation (Altun‐Gultekin *et al*., 2001), although the *ceh‐23(ms23)* null mutant has no obvious neuronal or behavioral defects (Walter *et al*., 2011).

In addition to CEH‐23, CEP‐1 is another transcription factor that is required for the longevity of the *isp‐1* mutant (Baruah *et al*., 2014; Ventura *et al*., 2010). *cep‐1* encodes the sole *C. elegans* ortholog of the mammalian p53 family, which acts as a transcription factor in response to various stresses (Derry *et al*., 2001; Baruah *et al*., 2014). Mammalian p53 is well known to participate in DNA repair, cell cycle regulation, and apoptosis, processes that have also been implicated in aging (Levine, 1997). *C. elegans* CEP‐1, similar to its mammalian ortholog, participates in apoptosis and cell cycle regulation (Derry *et al*., 2001; Greiss *et al*., 2008). Recent studies in *C. elegans* revealed intriguing opposing roles of CEP‐1 in the longevity of different ETC mutants (Baruah *et al*., 2014; Ventura *et al*., 2010).

In this study, we investigated the possible collaboration of the transcription factors CEH‐23 and CEP‐1 in modulating longevity in response to ETC dysfunction. Our data suggested that *ceh‐23* and *cep‐1* act in the same pathway to mediate the longevity of the complex III *isp‐*1 mutant. We also demonstrated that CEH‐23 and CEP‐1 regulate a common set of target genes, which are overrepresented by kinases and phosphatases of specific families, with likely roles in signal transduction. Intriguingly, the majority of the CEH‐23 and CEP‐1 co‐regulated transcriptional targets are also regulated by activated AAK‐2/AMPK signaling, suggesting a link between these two transcription factors and AAK‐2/AMPK signaling. Further analyses indicated that CEH‐23 and CEP‐1 play key roles downstream of AAK‐2 to modulate stress response and lifespan. As AMPK, CEP‐1, and CEH‐23 are all highly conserved, the findings reported here suggested that the mammalian counterparts of these proteins likely have similar roles in mediating the adaptive response to mitochondrial dysfunction.

## Results

### *ceh‐23* and *cep‐1* act in the same genetic pathway to modulate longevity of the ETC mutants

**Figure 1:**
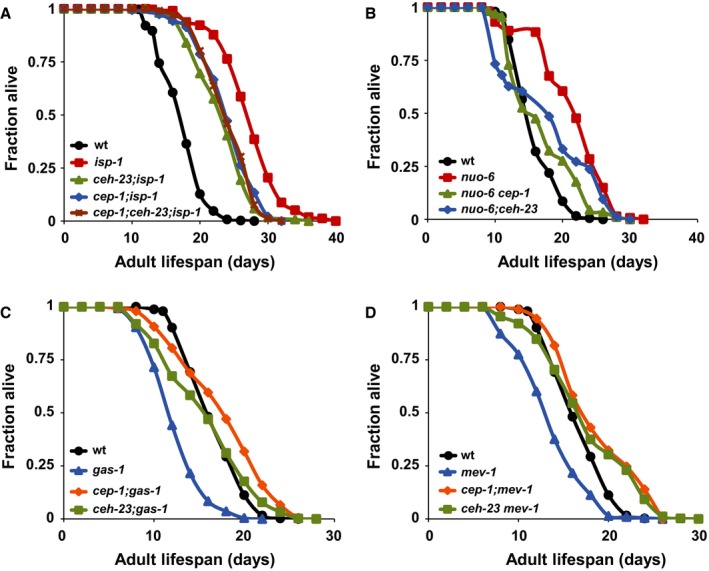
*ceh‐23* and *cep‐1* act in the same genetic pathway to modulate longevity when mitochondrial electron transport chain (ETC) function is impaired. (A) Both *ceh‐23* and *cep‐1* mutations partially suppressed the extended lifespan of the *isp‐1(qm150)* mutant (*P* < 0.0005), and inactivation of *ceh‐23* and *cep‐1* did not additively suppress *isp‐1* mutant lifespan, as the triple mutant lived as long as the double mutants (*P* = 0.529 compared to *cep‐1;isp‐1* and *P* = 0.003 compared to *ceh‐23;isp‐1*). Inactivation of *ceh‐23 and cep‐1* partially suppressed the long lifespan of the *nuo‐6(qm200)* mutant (*P* < 0.0005 and 0.001, respectively) (B) and restored lifespan in the short‐lived *gas‐1(fc21)* (C) and *mev‐1(kn1)* (D) mutants (all with *P *< 0.0005). Survival curves represent data pooled from multiple biological replicates. Quantitative data for the individual and pooled experiments are shown in Table S1 (Supporting information).

Both CEH‐23 and CEP‐1 have been shown to be critical for the extended lifespan associated with the *isp‐1* ETC mutant (Baruah *et al*., 2014; Ventura *et al*., 2010; Walter *et al*., 2011). We performed epistasis analysis to assess how these two transcription factors might interact to modulate the lifespan of mitochondrial ETC mutants. While *ceh‐23* and *cep‐1* contribute to part of the extended lifespan of the *isp‐1* mutant, we found that the triple mutant *cep‐1; ceh‐23; isp‐1* had a lifespan similar to the *cep‐1; isp‐1* and *ceh‐23; isp‐1* double mutants (Fig. 1A). Similar to that reported for each single mutation (Baruah *et al*., 2014; Walter *et al*., 2011), combining the *cep‐1* and *ceh‐23* mutations had no effect on wild‐type lifespan (Fig. S2A), suggesting that these two transcription factors are not required to maintain normal lifespan. Because combining the *ceh‐23* and *cep‐1* mutations did not result in an additive suppression of *isp‐1* mutant lifespan, we concluded that *ceh‐23* and *cep‐1* act in the same genetic pathway to modulate *isp‐1* mutant longevity.

*cep‐1* has been shown to have opposing effects on longevity in different mitochondrial ETC mutants. It promotes longevity in the *nuo‐6* and *isp‐1* mutants, as *cep‐1* inactivation suppresses the long life of these mutants. In contrast, *cep‐1* limits the lifespan of the short‐lived *gas‐1* and *mev‐1* mutants, as *cep‐1* mutation restores normal lifespan in these mutants (Baruah *et al*., 2014; Ventura *et al*., 2010). Because our data suggested that *ceh‐23* and *cep‐1* act in the same genetic pathway to modulate longevity of the *isp‐1* mutant, we expected that *ceh‐23* would have similar opposing roles in the various ETC mutants. Consistent with our hypothesis, the *ceh‐23* mutation suppressed the extended lifespan of the long‐lived *nuo‐6* mutant (Fig. 1B) but restored lifespan in the short‐lived *gas‐1* and *mev‐1* mutants (Fig. 1C,D). Thus, *ceh‐23* and *cep‐1* are required for the altered lifespans of the various ETC mutants, suggesting that they play a key role in modulating lifespan in responding to ETC dysfunction.

### CEH‐23 and CEP‐1 share a large group of transcriptional targets in response to mild ETC dysfunction

**Figure 2:**
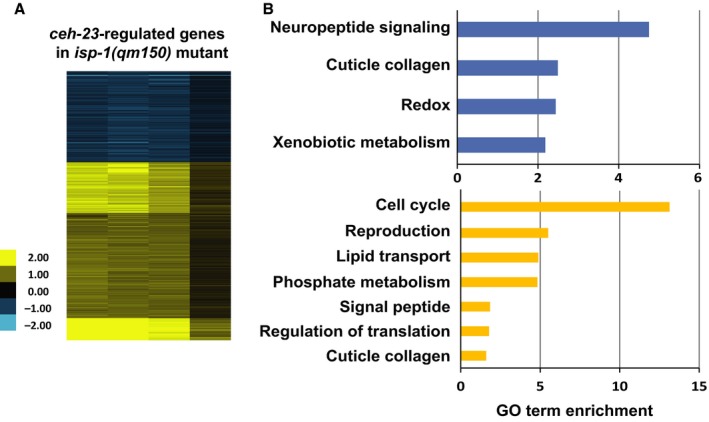
Transcriptomic targets of CEH‐23. (A) The heat map shows the genes whose expression changes significantly between the *isp‐1(qm150)* mutant compared to the *ceh‐23(ms23); isp‐1(qm150)* double mutant. Gene changes were identified by Statistical Analysis of Microarray (SAM) one‐class analysis false discovery rate (FDR) = 0, fold change > 1.5). Genes downregulated in *isp‐1* relative to *ceh‐23; isp‐1* are shown in blue and genes upregulated are shown in yellow. The intensity of the heat map represents the log2 ratio of the expression comparison. The significant gene lists are presented in Table S2 (Supporting information). (B) The most enriched Gene Ontology (GO) terms among the CEH‐23‐upregulated genes (yellow bars) and CEH‐23‐downregulated genes (blue bars). *X*‐axis represents GO term enrichment scores.

**Figure 3:**
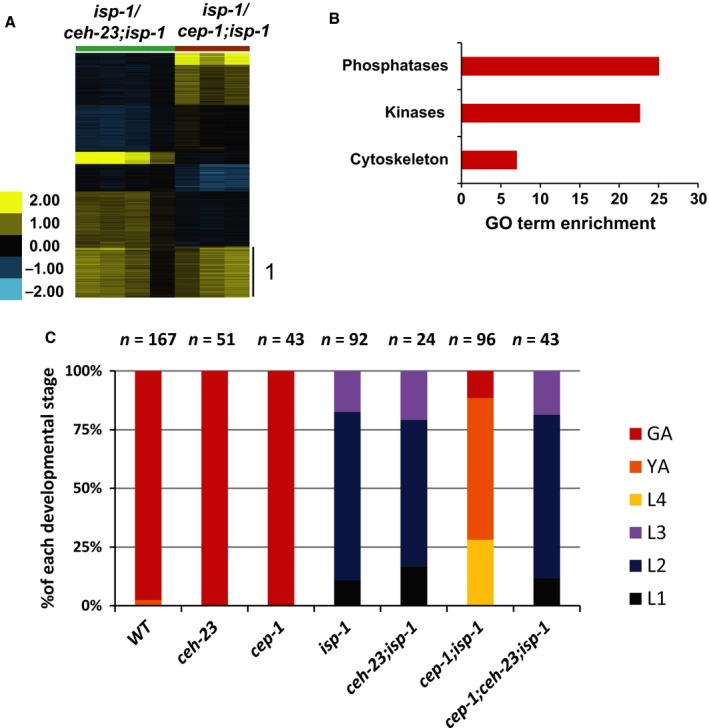
Transcriptomic analyses revealed that CEH‐23 and CEP‐1 co‐regulate a large set of genes in response to electron transport chain (ETC) dysfunction. (A) Genomewide comparison of expression changes between *isp‐1(qm150)* and *ceh‐23(ms23); isp‐1(qm150)* or *cep‐1(gk138); isp‐1(qm150)*. The common targets of CEH‐23 and CEP‐1 in the *isp‐1* mutant false discovery rate (FDR = 0%, fold change > 1.5 fold) are indicated as Cluster 1. Gene clustering was performed using K‐mean clustering with K = 7. The intensity of the heat map represents the log2 ratio of the expression comparison. The *isp‐1(qm150) vs. cep‐1(gk138);isp‐1(qm150)* microarray were performed in the *Baruah et al*. study. (B) The most enriched Gene Ontology (GO) terms among the CEH‐23 and CEP‐1 common targets. *X*‐axis represents GO term enrichment scores. Gene lists used for the GO terms analysis are presented in Table S3 (Supporting information). (C) Postembryonic developmental phenotypes of the indicated strains (wild‐type, *ceh‐23(ms23), cep‐1(gk138), isp‐1(qm150), ceh‐23(ms23);isp‐1(qm150), cep‐1(gk138);isp‐1(qm150), cep‐1(gk138);ceh‐23(ms23);isp‐1(qm150)*). Bar graph shows the fraction of each developmental stage 60 h after egg lay for each genotype. The sample size (n numbers) for each genotype is denoted above the bar graph. (L1‐L4: larval stage 1–4, YA: young adults, GA: gravid adults.)

To elucidate the possible molecular basis by which CEH‐23 and CEP‐1 modulate lifespan in the long‐lived ETC complex III *isp‐1* mutant, we compared the transcriptional outputs of these transcription factors in the *isp‐1* mutants. We previously identified the transcriptional responses regulated by CEP‐1 in the *isp‐1* mutant and found that CEP‐1 regulates the expression of a broad array of genes in response to ETC stress, including those involved in phosphate metabolism, lipid modification, and neuropeptide signaling (Baruah *et al*., 2014). We performed similar microarray analyses to profile the CEH‐23 dependent transcriptomic response in the long‐lived *isp‐1* mutant by comparing the global gene expression profiles between *isp‐1* and *ceh‐23; isp‐1* mutant worms synchronized at larval stage 4 (L4), which is a clearly identifiable developmental stage and a temporal window reported to be important for some ETC dysfunctions to influence adult lifespan (Dillin *et al*., 2002). Using the statistical tool SAM (Statistical Analysis of Microarray) (Tusher *et al*., 2001) with stringent parameters (false discovery rate (FDR) = 0.59%, fold change > 1.5‐fold), we identified 1878 genes whose expression change was *ceh‐23* dependent. Of these genes, 1244 were upregulated, and 634 were downregulated, in the *isp‐1* mutant with respect to the *ceh‐23; isp‐1* double mutant (Fig. 2). Gene Ontology (GO) analyses revealed that the 1244 genes (upregulated in the *isp‐1* mutant where CEH‐23 is functional) were enriched for genes involved in many fundamental biological processes, in particular cell cycle, reproduction, lipid transport, and phosphate metabolism (Fig. 2). The 634 genes (downregulated in the *isp‐1* mutant) were especially enriched for the biological function neuropeptide signaling (Fig. 2). These data suggested that CEH‐23 promotes the expression of development, reproduction, and metabolism genes and represses neuronal signaling genes in response to mild complex III dysfunction. Our previous works showed that *ceh‐23* deficiency in the *isp‐1* mutant did not affect the slow development or reduced reproduction phenotypes (Walter *et al*., 2011); however, *cep‐1* mutation partially restored normal development in the *isp‐1* mutant (Baruah *et al*., 2014). Interestingly, we found that functional *ceh‐23* was required for *cep‐1* to modulate development in the *isp‐1* mutant (Fig. 3C). Exactly how these two factors interact in this context awaits further investigation.

Given that earlier epistasis analyses revealed that *ceh‐23* and *cep‐1* likely act in the same genetic pathway to modulate longevity in ETC mutants (Fig. 1A), we hypothesized that these two factors would share a transcriptional outcome that is important for longevity determination during ETC dysfunction. We compared the CEH‐23‐dependent transcriptional response to *isp‐1* mutation with the previously published CEP‐1‐dependent response (Baruah *et al*., 2014) to identify any possible shared transcriptional outputs between these transcription factors. Consistent with our hypothesis, statistical analyses with stringent criteria (FDR = 0%, fold change > 1.5‐fold) revealed a substantial number of genes that were upregulated in the *isp‐1* mutant in a CEH‐23‐ and CEP‐1‐dependent manner (897 genes) (Fig. 3A Cluster 1, Table S3). Interestingly, this analysis revealed very few genes that were downregulated in the *isp‐1* mutant compared to the *ceh‐23; isp‐1* and *cep‐1; isp‐1* mutants (19 genes) (Table S3). We herein term the group of genes whose expression changed similarly when comparing *isp‐1 vs. ceh‐23; isp‐1* or *isp‐1 vs. cep‐1; isp‐1* the ‘CEH‐23 and CEP‐1 common target genes’ (916 genes). GO term analyses revealed that the CEH‐23 and CEP‐1 common target genes were enriched with kinases and phosphatases (Fig. 3B), suggesting that CEH‐23 and CEP‐1 regulate signaling networks to prolong *isp‐1* mutant lifespan.

### CEH‐23 and CEP‐1/p53 share many transcriptional targets with active AAK‐2/AMPK signaling

**Figure 4:**
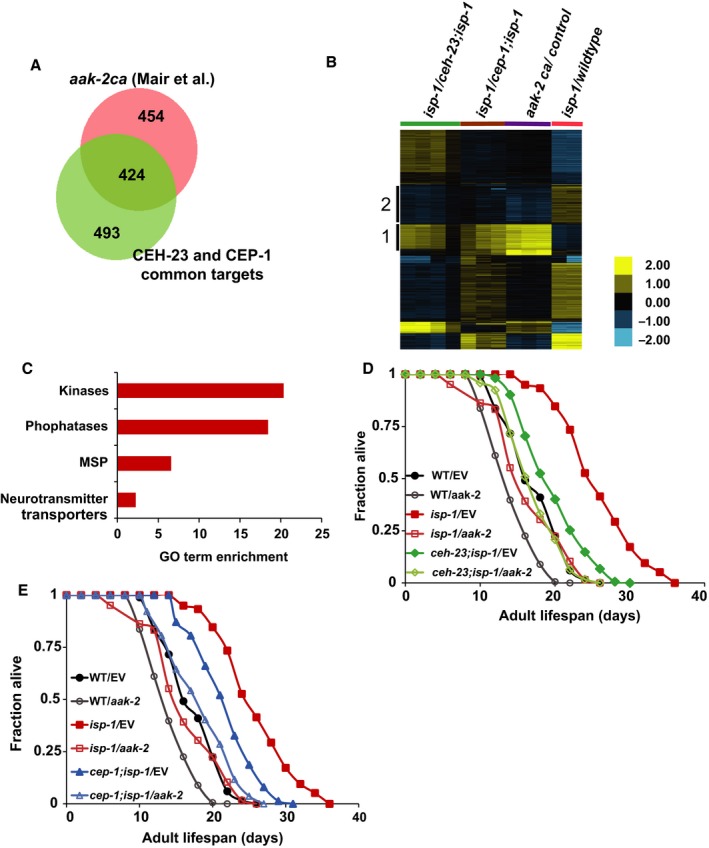
CEH‐23, CEP‐1, and AAK‐2 act in the same pathway to mediate *isp‐1* mutant lifespan. (A) The Venn diagram shows a substantial overlap between the genes that are commonly regulated by CEH‐23 and CEP‐1 in the *isp‐1* mutant and those regulated by constitutively active AAK‐2 (*aak‐2 ca*) (Representation factor: 7.0; *P* < 0.000e+00). The representation factor and *P*‐value (determined by hypergeometric probability test) were calculated using the web‐based tool (http://nemates.org/MA/progs/overlap_stats.html). The *aak‐2ca* microarray data are obtained from *Mair et al.,* 2011 *;* (B) The heat map represents a genomewide comparison of expression patterns between *isp‐1(qm150)* vs. *ceh‐23(ms23); isp‐1(qm150)* or *cep‐1(gk138); isp‐1(qm150), aak‐2ca* vs. transgenic control (data from *Mair et al.,* 2011), and *isp‐1(qm150)* vs. wild‐type. Genes without significant differences in any of the pairwise comparisons have been filtered out. Genes are grouped using K‐mean clustering with K = 7. Cluster 1 highlights genes that are commonly regulated by CEH‐23, CEP‐1, and AAK‐2, and Cluster 2 highlights genes that shared targets between CEH‐23 and AAK‐2. (C) The most enriched Gene Ontology (GO) terms among the CEH‐23, CEP‐1, and AAK‐2 common targets. *X*‐axis represents GO term enrichment scores. Gene lists for the GO terms are presented in Table S5 (Supporting information). *ceh‐23; isp‐1* (D) and *cep‐1; isp‐1* (E) mutants treated with *aak‐2* RNAi had similar lifespan as the *isp‐1* single mutant treated with *aak‐2* RNAi, suggesting that *ceh‐23* and *cep‐1* and *aak‐2* act in the same genetic pathway to modulate *isp‐1* mutant lifespan.

Although the CEH‐23 and CEP‐1 common transcriptional targets were enriched with kinases and phosphatases, these proteins had not been directly implicated in known longevity pathways. To further assess the roles of CEH‐23 and CEP‐1, we performed an unbiased search for factors that also regulate the expression of the CEH‐23 and CEP‐1 common target genes using the WormMine tool at www.wormbase.org. Interestingly, this analysis revealed a highly significant overlap between CEH‐23 and CEP‐1 common target genes and the genes that are upregulated by constitutively active AAK‐2, the AMP kinase (AMPK) catalytic subunit homolog in *C. elegans* (424 Genes) (Fig. 4A,B, Cluster 1) (Mair *et al*., 2011).

AMPK is a well‐conserved kinase that serves as a cellular energy sensor. It regulates diverse biological processes in response to different environmental stresses and cellular energy levels, in particular a change in AMP:ATP ratio. AMPK has been implicated in several pathways that are key to organismal lifespan (Apfeld *et al*., 2004; Greer *et al*., 2007a,b). Furthermore, overexpression of a constitutively active AAK‐2 is sufficient to extend *C. elegans* lifespan (Mair *et al*., 2011). Given that mitochondria are important for cellular fuel production, defects in the mitochondrial ETC are likely to trigger a cellular energy imbalance and thus activate AMPK. Indeed, AAK‐2/AMPK activation appears to be enhanced in the *isp‐1* mutant (Hwang *et al*., 2014) and is required for the extended lifespan of the *isp‐1* mutant (Curtis *et al*., 2006). Together, our findings suggest that AAK‐2 signaling, CEH‐23, and CEP‐1 converge to modulate longevity in the *isp‐1* mutant. GO term analyses of the genes that are commonly regulated by AAK‐2, CEH‐23, and CEP‐1 revealed a highly significant enrichment for kinases, phosphatases, major sperm proteins (MSPs), and neurotransmitter transport proteins (Fig. 4C). Therefore, AAK‐2, CEP‐1, CEH‐23 may regulate downstream signal transduction to promote longevity in mutant worms with mild ETC dysfunction. Although AAK‐2/AMPK is known to modulate lifespan under a broad range of conditions (Burkewitz *et al*., 2014), the mechanism by which AAK‐2 mediates the longevity of mitochondrial ETC mutants remains unclear. Our results revealed a new connection between AAK‐2 and the transcription factors CEH‐23 and CEP‐1.

We next compared the transcriptional profiles of the *isp‐1* mutants with or without *ceh‐23* or *cep‐1* with that of wild‐type worms (Fig. 4B). This comparison revealed a somewhat surprising pattern where the genes that were commonly upregulated in the *isp‐1* mutant in a CEH‐23‐ and CEP‐1‐dependent manner appeared to be downregulated in the *isp‐1* mutant relative to wild‐type worms (Fig. 4B, Cluster 1), suggesting that functional CEH‐23 and CEP‐1 are required for maintaining the expression of these genes in the *isp‐1* mutant, albeit at levels lower than wild‐type. Inactivation of *ceh‐23* and *cep‐1* in the *isp‐1* mutant results in further repression of these genes. As the *isp‐1* mutant is long‐lived compared to the *ceh‐23; isp‐1* or *cep‐1; isp‐1* mutants, we proposed that activation of AAK‐2, CEH‐23, and CEP‐1 in the *isp‐1* mutant acts to buffer the downregulation of these genes, allowing the worms to live long, and the absence of AAK‐2, CEH‐23, or CEP‐1 results in further repression of these genes and a detrimental effect on lifespan.

### CEH‐23 and CEP‐1 are downstream effectors of AAK‐2 signaling

To further delineate the relationship of AAK‐2, CEH‐23, and CEP‐1 in response to ETC stress, we assessed the effect of *aak‐2* depletion in *isp‐1* mutants lacking functional *ceh‐23* or *cep‐1*. First, we sought to confirm the role of *aak‐2* in lifespan modulation. Consistent with previous findings (Curtis *et al*., 2006), we found that RNAi knockdown of *aak‐2* slightly shortened the lifespan of wild‐type animals and substantially suppressed the extended lifespan of the *isp‐1* mutant (Fig. 4D,E, Table S1C), indicating that AAK‐2 signaling is essential for normal lifespan and required for the full lifespan extension of *isp‐1* mutant. If AAK‐2, CEH‐23, and CEP‐1 act together to modulate lifespan in the *isp‐1* mutant, we would predict that loss of *aak‐2* and *ceh‐23* or *cep‐1* would not additively suppress *isp‐1* mutant lifespan. Indeed, we observed that *isp‐1* mutants devoid of both *ceh‐23* and *aak‐2* (*ceh‐23; isp‐1; aak‐2*(RNAi or mutation)) or *cep‐1* and *aak‐2* (*cep‐1; isp‐1; aak‐2*(RNAi or mutation)) lived as long as *isp‐1* mutants with only *aak‐2* depleted (Fig. 4D,E, Table S1C). Taken together, our data showed that inactivation of *ceh‐23* or *cep‐1* and depletion of *aak‐2* do not additively suppress *isp‐1* mutant lifespan, thus supporting the model that AAK‐2/AMPK signaling acts with CEH‐23 and CEP‐1/p53 to prolong the lifespan of the *isp‐1* mutant. It is important to note that *aak‐2* RNAi had a greater effect in suppressing the extended lifespan of the *isp‐1* mutant compared to *ceh‐23* or *cep‐1* inactivation, suggesting that AAK‐2 likely works with additional effectors, beyond CEH‐23 and CEP‐1, to modulate longevity in the *isp‐1* mutant.

**Figure 5:**
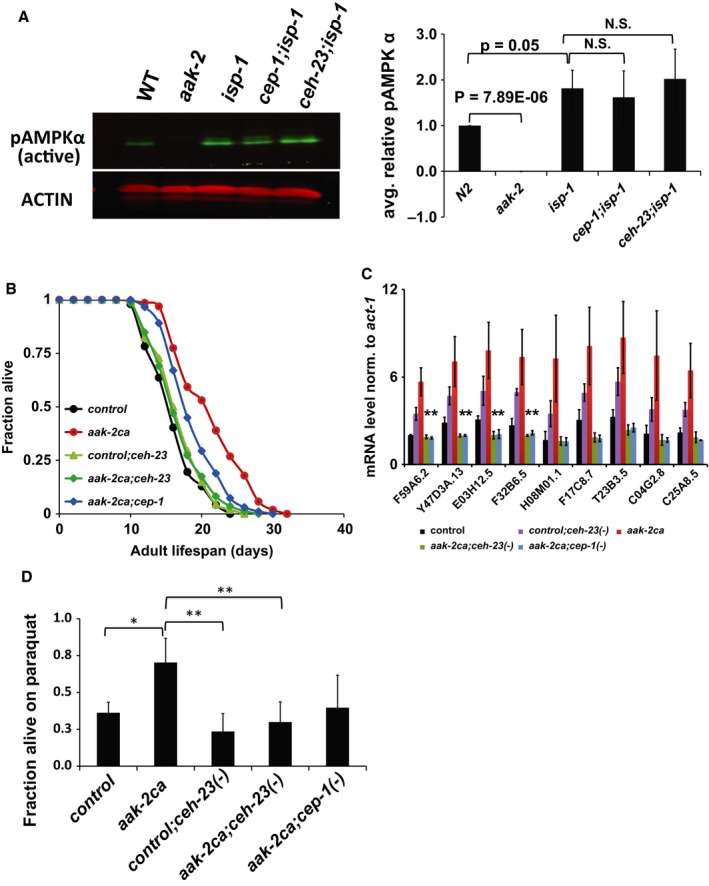
CEH‐23, CEP‐1 act downstream of AAK‐2/AMPK signaling. (A) Western blot analysis showed that activated AAK‐2 levels are similarly elevated in the *isp‐1* single mutant and in the *ceh‐23; isp‐1* and *cep‐1; isp‐1* double mutants. Quantified pAAK‐2/AMPKα levels from three independent experiments are presented in the bar graph. Both *ceh‐23* and *cep‐1* mutations suppressed the extended lifespan (B), transcriptional response (C), and oxidative stress resistance (D) of *aak‐2ca* worms. In (D), *P*‐value is represented as following: *< 0.05, **< 0.01. Although the difference in oxidative stress resistance between *aak‐2ca; cep‐1(‐)* and *aak‐2ca* is not significant, we consistently observed this difference in all our trials. *aak‐2ca* refers to WBM60 and control refers to WBM59. Our attempt to introduce the *cep‐1* mutation into the control strain was not successful, so only control strain carrying the *ceh‐23* mutation was included in this experiment.

As the microarray and epistasis data suggested that AAK‐2, CEH‐23, and CEP‐1 act together to modulate the lifespan of the *isp‐1* mutant, we tested whether CEH‐23 and CEP‐1 could influence AAK‐2/AMPK activity by measuring the levels of phosphorylated form of AAK‐2 in *isp‐1* mutants with or without *ceh‐23* or *cep‐1*. We found that upregulation of active AAK‐2/AMPK in the *isp‐1* mutant did not depend on functional *ceh‐23* or *cep‐1*, as neither *ceh‐23* nor *cep‐1* mutation reduced phospho‐AAK‐2 levels in the *isp‐1* mutants (Fig. 5A). Moreover, inactivation of CEH‐23 or CEP‐1 also had no detectable effect on the mRNA levels of *aak‐2* in the *isp‐1* mutants (Fig. S1C). Taken together, our data suggested that CEP‐1 and CEH‐23 likely do not regulate AAK‐2 activity in the *isp‐1* mutant.

We next tested whether *aak‐2* RNAi, like that of *ceh‐23* or *cep‐1* deletion, would restore lifespan in the short‐lived *gas‐1* and *mev‐1* mutants. To our surprise, knocking down *aak‐2* has no effect on the lifespan of the short‐lived ETC *mev‐1* and *gas‐1* mutants (Fig.S2B). Therefore, whereas *aak‐2, ceh‐23, cep‐1* appear to act in the same pathway to promote lifespan in the *isp‐1* mutant, they do not act the same way in response to the complex I *gas‐1* and complex II *mev‐1* mutations. As discussed above, CEH‐23 and CEP‐1 likely represent only a subset of the effectors of AAK‐2/AMPK signaling, and it is therefore possible that *aak‐2* RNAi has a different effect on the lifespan of the *gas‐1* and *mev‐1* mutants due to additional outputs beyond CEH‐23 and CEP‐1.

We next investigated whether CEH‐23 and CEP‐1 could mediate the biological outputs of AAK‐2 beyond the context of mitochondrial dysfunction by testing whether functional CEH‐23 and CEP‐1 are required for the extended lifespan and the increased resistance to oxidative stress of worms overexpressing constitutively active AAK‐2 (*aak‐2ca*) in an otherwise wild‐type background (Mair *et al*., 2011; Hwang *et al*., 2014). The data showed that both *ceh‐23* and *cep‐1* were required for the extended lifespan of the *aak‐2ca* animals (Fig. 5B), suggesting that CEH‐23 and CEP‐1 act downstream of AMPK signaling for longevity determination. Consistent with this finding, functional CEH‐23 and CEP‐1 were also required for the increased tolerance to oxidative stress of the *aak‐2ca* animals (Fig. 5D). Interestingly, *ceh‐23* inactivation appeared to have a greater effect on suppressing the prolonged lifespan of the *aak‐2ca* animals compared to *cep‐1* inactivation. This observation suggested that CEH‐23 mediates a greater portion of the longevity effect of AMPK than CEP‐1, which might be reflected by the gene expression comparison where CEH‐23, but not CEP‐1, repressed the expression of a group of genes in the *isp‐1* mutants that are also downregulated in *aak‐2ca* worms (Fig. 4B, Cluster 2). To further test whether CEH‐23 and CEP‐1 act downstream of AAK‐2, we asked whether CEH‐23 and CEP‐1 could mediate some of the transcriptional responses caused by constitutive active AAK‐2. Microarray analyses revealed a group of genes that were regulated by CEH‐23 and CEP‐1 in the *isp‐1* mutant and were upregulated when AAK‐2 is overactive (Fig. 4B, Cluster 1). Quantitative PCR analyses demonstrated that the induction of these genes in the *aak‐2ca* animals required *ceh‐23* and *cep‐1* (Fig. 5C). Together, our data supported the model that CEH‐23 and CEP‐1 act downstream of AAK‐2 to modulate some of its biological outputs, including longevity and oxidative stress response.

### CEH‐23 and CEP‐1 likely act downstream of CRTC‐1 to modulate longevity

**Figure 6:**
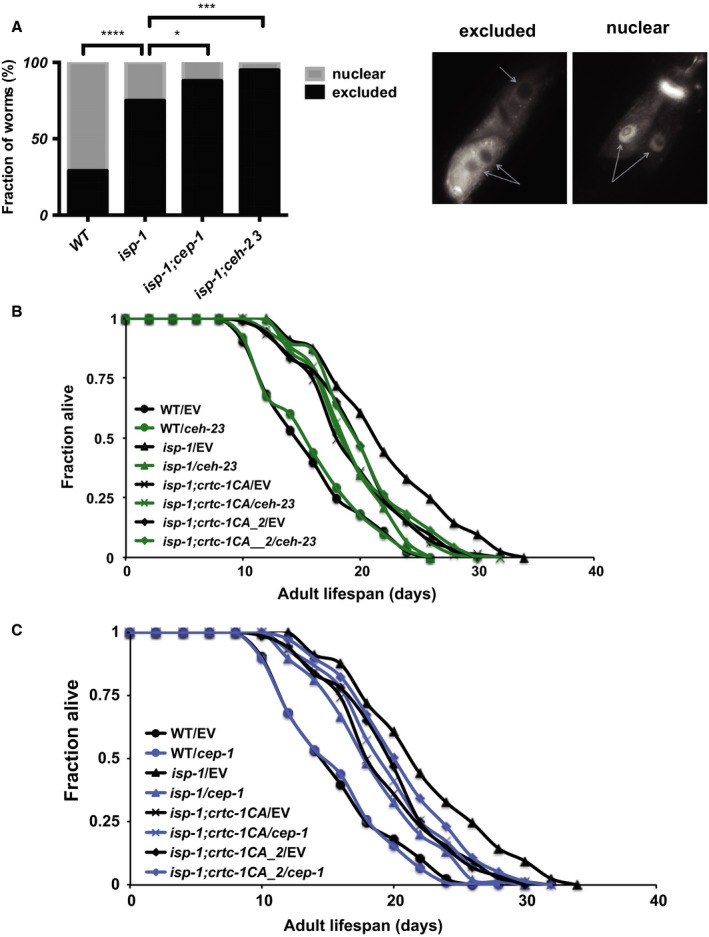
CEH‐23, CEP‐1 likely act downstream of CRTC‐1. (A) More CRTC‐1 was excluded from the nucleus in the *isp‐1* mutant compared to wild‐type worms, and the nuclear exclusion was slightly enhanced when *ceh‐23* and *cep‐1* were mutated. CRTC‐1 localization experiments were performed using 40 young adults (YA) worms for each genotypes and the localization of CRTC‐1 in intestinal cells was scored. Microscopic images show representative nuclear‐excluded and nuclear‐localized CRTC‐1 pattern. Gray arrows denote intestinal nuclei (*P*‐value: ****< 0.0001, ***< 0.0005, *< 0.05). (B, C) Forced expression of constitutively nuclear CRTC‐1 partially suppressed the prolonged lifespan of the *isp‐1* mutant and *ceh‐23* (B) or *cep‐1* (C) RNAi knockdown did not further impact the suppressed lifespan.

AAK‐2 is well established to modulate longevity by acting through the transcription factor DAF‐16*/*FOXO and the transcriptional coactivator CRTC‐1/CRTCs. Both DAF‐16 and CRTC‐1 can be phosphorylated by AMPK and both play crucial roles in mediating the longevity effect of AAK‐2, albeit in an opposing manner (Greer *et al*., 2007a,b; Mair *et al*., 2011). *daf‐16* encodes the sole *C. elegans* ortholog of the FOXO transcription factors and is a key effector of the insulin/IGF‐1 signaling (IIS) pathway (Kenyon *et al*., 1993; Ogg *et al*., 1997; Lin *et al*., 1997). However, IIS and *daf‐16* have been shown to modulate longevity through a mechanism distinct from that associated with mild ETC dysfunction (Feng *et al*., 2001), and we have previously demonstrated that *ceh‐23* and *daf‐16* likely act separately to mediate the lifespan of the *isp‐1* mutant worms (Walter *et al*., 2011). *crtc‐1* encodes the sole *C. elegans* ortholog of the CREB‐regulated transcription coactivator, and AAK‐2 has been shown to promote longevity by sequestering CRTC‐1 outside of the nucleus through phosphorylation (Mair *et al*., 2011). The subcellular localization and the longevity effect of CRTC‐1 in worms with a mild ETC dysfunction have not been explored. We used a tdTomato‐fused *crtc‐1* transgene to monitor the subcellular localization of CRTC‐1 in the *isp‐1(qm150)* mutant. Similar to that seen in the *aak‐2ca* animals (Mair *et al*., 2011), we observed greater nuclear exclusion of CRTC‐1 in the *isp‐1* mutants compared to wild‐type (Fig. 6A), which is consistent with the idea that AAK‐2 is activated in the *isp‐1* mutant. We next tested whether CEH‐23 or CEP‐1 might influence the subcellular location of CRTC‐1 in the *isp‐1* mutant. We found that the nuclear exclusion of CRTC‐1::tdTomato in the *isp‐1* mutant was not reduced by either *cep‐1* or *ceh‐23* mutations, suggesting that *cep‐1* and *ceh‐23* are not required for active AAK‐2 signaling to expel CRTC‐1 from the nucleus. In fact, the data suggested that more CRTC‐1 was excluded from the nucleus when *cep‐1* and *ceh‐23* were mutated, which might point to a possible feedback regulation.

It is known that constitutively nuclear‐localized CRTC‐1 (CRTC‐1CA) has a negative impact on the extended lifespan of the *aak‐2ca* mutant (Mair *et al*., 2011); we therefore tested whether CRTC‐1CA expression also suppressed the extended lifespan of the *isp‐1* mutants. We found that constitutive nuclear expression of CRTC‐1 (CRTC‐1CA) partially suppressed the extended lifespan of the *isp‐1* mutant, consistent with the expectation that AAK‐2 activation and CRTC‐1 nuclear exclusion are important for mediating the prolonged lifespan of the *isp‐1* mutant (Fig. 6B,C). We next tested whether *crtc‐1* and *ceh‐23 and cep‐1* might act in parallel or interdependently to mediate the lifespan of the *isp‐1* mutant. We found that inactivating either *ceh‐23* or *cep‐1* with either RNAi or mutations did not further reduce the shortened lifespan of the *isp‐1; crtc‐1ca* worms (Fig. 6B,C, Fig. S3B,C), indicating that *ceh‐23, cep‐1*, and *crtc‐1* likely act in the same genetic pathway to mediate the extended longevity of the *isp‐1* mutant. As it is the forced expression of CRTC‐1 in the nucleus and the loss of CEH‐23 and CEP‐1 that suppressed the extended lifespan of the *isp‐1(qm150)* mutant, we interpreted the results to suggest that nuclear CRTC‐1 can act as a negative regulator of CEH‐23 and CEP‐1 to limit longevity in the *isp‐1* mutants.

## Discussion

Highly regulated communications between mitochondria and the nucleus are required for maintaining biological functions in response to compromised mitochondrial function. The transcription factors CEH‐23 and CEP‐1 have previously been identified as key mediators of the longevity extension phenotype of the mitochondrial ETC complex III mutant *isp‐1(qm150)* (Baruah *et al*., 2014; Ventura *et al*., 2010; Walter *et al*., 2011). In this study, we investigated how these transcription factors mediate longevity specifically focusing on the possible collaboration between them. Epistasis analyses demonstrated that *ceh‐23* and *cep‐1* act through the same genetic pathway to mediate the longevity of several different ETC mutants. Consistent with the genetic results, microarray analyses revealed that CEH‐23 and CEP‐1 share a large set of transcriptional targets in response to mitochondrial complex III dysfunction. Intriguingly, the majority of the CEH‐23 and CEP‐1 co‐regulated genes are also transcriptional targets of constitutively active AAK‐2 (Fig. 4A,B), thus pointing to a new link between CEH‐23, CEP‐1, and the AAK‐2 signaling pathway. We note that only one allele of *cep‐1* and *ceh‐23* mutants were used, so we cannot rule out allele‐specific interactions. Nevertheless, further functional analyses suggested that CEH‐23 and CEP‐1 act downstream of AAK‐2 to modulate lifespan, stress response, and gene expression, either with or without mitochondrial dysfunction.

The new link between *ceh‐23, cep‐1,* and *aak‐2* is bolstered by their genetic interactions with the AAK‐2/AMPK target, CRTC‐1 (Fig. 6B,C). CRTC‐1 is known to be regulated by AMPK through phosphorylation, and the nuclear exclusion of CRTC‐1 is important for the lifespan extension associated with constitutively active AAK‐2/AMPK (Mair *et al*., 2011; Burkewitz *et al*., 2015). Our data indicated that nuclear‐excluded CRTC‐1 is also required for the extended lifespan of the *isp‐1* mutant (Fig. 6B,C), suggesting that CRTC‐1 plays a broad role in modulating longevity in response to various stresses that activate AMPK activity. Additional functional analyses suggested a model that CRTC‐1 acts as a negative regulator of CEH‐23 and CEP‐1 in the nucleus and when AAK‐2/AMPK is activated, like in the *isp‐1* mutant, phosphorylated CRTC‐1 is expelled from the nucleus, enabling CEH‐23 and CEP‐1 to regulate gene expression that prolong lifespan. We noted that the expression of many of the AAK‐2, CEH‐23, and CEP‐1 common targets were repressed in the *isp‐1* mutant relative to wild‐type worms, but their expression were further reduced in the *ceh‐23; isp‐1* and *cep‐1; isp‐1* double mutants relatively to the *isp‐1* single mutant. We therefore propose that the AAK‐2, CRTC‐1, CEH‐23, CEP‐1 signaling nexus act to maintain the expression levels of a subset of target genes that is important for the prolongevity effect associated with the *isp‐1* mutation.

In further support of the notion that CEH‐23 and CEP‐1 might act downstream of AAK‐2/AMPK, sequence alignment analyses revealed that both CEH‐23 and CEP‐1 contain putative AMPK phosphorylation motifs that appear to be highly conserved (http://scansite3.mit.edu/#home). Mammalian p53 is a known substrate of AMPK (Imamura *et al*., 2001), and multiple sequence alignment analysis revealed that *C*. *elegans* CEP‐1 also contains a highly conserved AMPK motif (Fig. S1A). Moreover, the predicted AMPK site on CEH‐23 is conserved across various nematode species (Fig. S1B), and EMX2, the putative mammalian homolog of CEH‐23, also contains two predicted AMPK motifs. The conserved nature of the predicted AMPK motifs in both CEP‐1 and CEH‐23 suggests that these factors could be AMPK substrates in diverse organisms. It is important to note that whereas we discovered a connection between AAK‐2 and CEH‐23 & CEP‐1 by studying the mitochondrial mutant *isp‐1*, our results with constitute active *aak‐2* animals suggested that CEH‐23 and CEP‐1 are likely downstream mediators of AAK‐2 in a broad contexts beyond mitochondrial dysfunction. Further elaboration of the molecular nature of the regulatory relationship among CEP‐1, CEH‐23, and AAK‐2 will provide important mechanistic insights that will likely be broadly relevant.

Bioinformatic analyses of the AAK‐2, CEH‐23, and CEP‐1 common targets revealed a possible interesting connection to neuronal function. Among the kinases that are commonly regulated by CEH‐23 and CEP‐1 in the *isp‐1* mutant and by activated AAK‐2, most belong to the TTBKL (Tau‐tubulin kinase‐like) serine/threonine kinase family. Human TTBK kinases have been associated with neurodegeneration, where TTBK1 is thought to phosphorylate Tau protein and promote the progression of Alzheimer's disease (Sato *et al*., 2006) and TTBK2 has been linked to another Tau‐related disease, spinocerebellar ataxia 11 (SCA11) (Ikezu & Ikezu, 2014). *C. elegans* has a single TTBK ortholog and 31 kinases that display sequence similarity to TTBK (Manning, 2005). One of these TTBK‐like kinases has recently been implicated in the pathogenesis of a *C. elegans* model of the neurodegenerative disease amyotrophic lateral sclerosis (ALS) (Ikezu & Ikezu, 2014). Another interesting group of genes revealed as CEH‐23, CEP‐1, and AAK‐2 common targets encode MSPs. The MSP domain is highly conserved, and a substitution mutation in the human MSP domain protein VAPB has been associated with ALS (Nishimura *et al*., 2004), suggesting a possible role of MSP domain proteins in neuronal function. Lastly, several sodium:neurotransmitter symporters are overrepresented among the common transcriptional targets of CEH‐23, CEP‐1, and AAK‐2. Taken together, it appears that neuronal signaling might represent a key downstream output of active AAK‐2 and CEH‐23 and CEP‐1 in the *isp‐1* mutant. Consistent to our bioinformatic observation, recent study has demonstrated that CRTC1‐dependent transcription is impaired in the early stage of Alzheimer's disease (Parra‐Damas *et al*., 2014). Moreover, it is interesting to note that ETC dysfunction in the neurons are thought to have a particularly key role in modulating longevity (Dillin *et al*., 2002). Furthermore, CEH‐23, CRTC‐1, and AAK‐2 are all expressed in neurons (Mair *et al*., 2011; Spencer *et al*., 2011; Walter *et al*., 2011), and the action of AAK‐2 and CRTC‐1 in neurons has also been demonstrated to play a particularly important role in modulating longevity (Burkewitz *et al*., 2015). An intriguing possibility is that the ETC dysfunction in the *isp‐1* mutant induces the activation of AMPK, CEH‐23, and CEP‐1 in the neurons, which elicits a protective effect on lifespan.

AMPK is a critical metabolic sensor and regulator and its deregulation has been linked to major diseases, such as cancer and neurodegenerative disease (Burkewitz *et al*., 2014; Cai *et al*., 2012). Likewise, mitochondrial ETC dysfunction is tightly linked to aging and many diseases (García‐Escudero *et al*., 2013). AMPK has been implicated as a key responder to mitochondrial dysfunction (Curtis *et al*., 2006; Hwang *et al*., 2014; Burkewitz *et al*., 2014), especially because of its ability to respond directly to altered AMP/ATP ratio and redox imbalance (Burkewitz *et al*., 2014). Our study reveals a new link between the transcription factors CEP‐1/p53 and CEH‐23 to AAK‐2/AMPK and implicates additional downstream signaling as an adaptive response that leads to prolonged lifespan in mutants with reduced ETC function. Importantly, our findings suggest that CEH‐23 and CEP‐1/p53 are key effectors of AAK‐2/AMPK in contexts beyond mitochondrial ETC dysfunction. Further investigation of the molecular relationship of AAK‐2/AMPK, CEH‐23, and CEP‐1/p53 will likely provide important insights that are broadly relevant to aging and age‐related diseases in diverse organisms.

## Experimental procedures

### *C. elegans* strains

All strain stocks were maintained on OP50 *Escherichia coli* at 20 °C and were handled under standard growth conditions. We used the following strains: Wild‐type N2 Bristol, IU279.1 *ceh‐23(ms23),* IU445 *cep‐1(gk138),* IU291.4 *isp‐1(qm150)*, IU398.1 *nuo‐6(qm200)*, IU231.2 *mev‐1(kn1)*, IU338.1 *gas‐1(fc21)*, IU293.2 *ceh‐23(ms23)*;* isp‐1(qm150)*, IU448 *cep‐1(gk138); isp‐1(qm150)*, IU418.1 *cep‐1(gk138); ceh‐23(ms23)*;* isp‐1(qm150)*, IU450.1 *mev‐1(kn1); cep‐1(gk138*), IU451.1 *cep‐1(gk138); gas‐1(fc21)*. Standard genetic methods were used to construct the following strains: IU399.2 *nuo‐6(qm200); ceh‐23(ms23)*, IU481.3 *ceh‐23(ms23) mev‐1(kn1)*, IU477.2 *ceh‐23(ms23); gas‐1(fc21)*, IU507.1 *uthIs248; ceh‐23(ms23),* IU508.1 *uthIs248; cep‐1(gk138),* and IU506.1 *uthIs272; ceh‐23(ms23)*. We failed to generate *uthIs272; cep‐1(gk138)* using the same cross‐strategy possibly because the transgene and *cep‐1(gk138)* are linked. *nuo‐6(qm200) cep‐1(gk138)* was generously provided by Dr. Siegfried Hekimi at McGill University. WBM60 *uthIs248[Paak‐2::aak‐2 genomic (aa1‐321)::GFP::unc54 3′UTR, Pmyo‐2::tdTomato]* is the transgenic strain with overexpressed *aak‐2ca* and WBM59 *uthIs272[Pmyo‐2::tdTomato, unc‐54 3′UTR]* is the transgenic control. Both WBM59 and WBM60 were generous gifts from Dr. Willian B. Mair at Harvard University.

### Lifespan analysis

All lifespan experiments were performed at 20 °C on Nematode Growth Media (NGM) plates seeded with *E. coli* OP50 or HT115 for RNAi experiments. For experiments with OP50, bacteria were cultured overnight at 37 °C, the OD600 was measured, and the overnight culture was concentrated to OD600 = 4.0. For RNAi experiments, HT115 bacteria containing vectors expressing dsRNA were concentrated to OD600 = 4. IPTG (4 mm) was added to RNAi bacteria‐seeded plates at room temperature overnight prior to use to induce dsRNA expression. Well‐fed gravid adult worms were allowed to lay eggs at 20 °C, and the progeny were grown at 20 °C. Survival was scored every other day, and the survival curves of each population were estimated using the Kaplan–Meier method, and a log‐rank test was performed for statistical analysis. A *P* ≤ 0.01 was considered significantly different from the control population. The independent trials were analyzed both separately and pooled between independent trials, and the pooled experiments are presented in the figures. Data from individual trials and pooled trials are presented in Table S1 (Supporting information).

### Oxidative stress assay

L4 worms were transferred to NGM plates with 10 mm paraquat or vehicle control (M9). Survival of worms was scored 96 h after exposure to paraquat. The experiment was performed at 20 °C.

### Microarray

*isp‐1(qm150) and ceh‐23(ms23); isp‐1(qm150)* mutant worms were grown on NGM plates with live OP50 bacteria. Worms were harvested when the majority of the population reached mid‐L4 stage. Total RNA was isolated using Tri‐reagent (Molecular Research Center, Inc., Cincinnati, OH USA) and purified with the RNeasy kit (Qiagen). cRNA synthesis/amplification, Cy3/Cy5 dye labeling, and hybridization onto Agilent (Santa Clara, CA USA) 4X44K *C. elegans* oligonucleotide microarrays (v2) were performed as previously described (Shaw *et al*., 2007). Hybridized microarray slides were washed according to Agilent instructions and scanned immediately on the Agilent DNA microarray scanner (G2505B) using one color scan setting for 4x44k array slides (scan area 61 × 21.6 mm, scan resolution 5um, dye channel is set to red & green and both the Red and Green PMT is set to 100%). The scanned images were analyzed with Feature Extraction Software 9.1 (Agilent) using parameters (protocol GE1‐105_DEC8 and Grid: 012391_D_20060331) to obtain background subtracted and spatially detrended processed signal intensities. Features flagged in Feature Extraction as Feature Nonuniform outliers were excluded. Two of the four arrays were dye‐flip replicates, and three independent biological experiments were performed. The microarray result was validated using RT‐qPCR. Detailed description of microarray analyses is listed in the Data S1 (supporting information).

### Western blot

Worms were grown on 10‐cm NGM plates seeded with OP50 *E. coli*, synchronized at the mid‐L4 stage, and harvested in M9 buffer. Worms were lysed by boiling. The protein concentrations were quantified using Quick Start Bradford Protein Assay (Bio‐Rad, Hercules, CA USA); 100 ng of total protein was loaded on 8% SDS‐PAGE, electrophoresed, and transferred to a nitrocellulose membrane. The membranes were blocked in TBST with 5% BSA and subsequently incubated with primary antibody, anti‐actin (mouse, Chemicon, Temecula, CA USA), and anti‐phospho‐AMPKα (rabbit, #4188 Cell Signaling Beverly, MA, USA). Anti‐phopho‐AMPKα incubation was performed at 4 °C for 6–9 h, and anti‐actin incubation was performed at room temperature for 1 h. Anti‐mouse or anti‐rabbit secondary antibodies conjugated to fluorescent dyes were used for anti‐actin and anti‐phopho‐AMPKα, respectively. The Western blot images were obtained with the Odyssey infrared imaging system and quantified using Image Studio ver. 2.0. Phospho‐AMPKα levels were normalized to actin as an internal control and normalized to WT.

### Development assay

Gravid adult worms were placed onto 60‐mm NGM plates seeded with OP50 *E. coli* and allowed to lay eggs for 3–5 h. Subsequently, the adults were removed, and the embryos were allowed to develop at 20 °C. The developmental stages were scored 60 h after egg lay. The L1‐L3 larval stages were scored based on gonad structure using a DIC microscope (Leica DM 5000B microscope, Mannheim, Germany). The L4 larval stage and adult worms were identified based on vulval morphology.

### Accession number

The GEO accession number for the *isp‐1(qm150) vs. ceh‐23(ms23); isp‐1(qm150)* microarray dataset in this paper is GSE67754.

## Conflict of interest

None declared.

## Funding

This work was supported by NIH R01 grant AG024425 to S.S.L. [Correction added on 15 June 2017, after first online publication: The funding information has been added in this current version.]

## Author contribution

H‐W. C. and S.S.L. conceived all the experiments, interpreted the results, and wrote the paper. H‐W. C designed and performed most of the experiments. S.B. performed the CRTC‐1 subcellular localization assay. A.C. performed some of the pAAK‐2 Western blots. J.C. performed the oxidative stress assay, and S.G. performed the developmental assay. A.B. performed the microarray experiment comparing transcription profile between *isp‐1* mutant and *cep‐1; isp‐1* mutant.

## Supporting information


**Fig. S1** CEH‐23 and CEP‐1 harbor putative AMPK phosphorylation sites.

**Fig. S2** (A) Wild‐type *C. elegans* lifespan was not altered by the combined loss of *ceh‐23* and *cep‐1*. The *ceh‐23(ms23)* and *cep‐1(gk138)* single and double mutants were used in the lifespan analysis. (B) *aak‐2* RNAi had little effect on the lifespan of the short‐lived *gas‐1(fc21)* and *mev‐1(kn1)* mutants. EV: empty vector RNAi control.

**Fig. S3** CRTC‐1 antagonizes CEH‐23 and CEP‐1 to modulate the lifespan of *isp‐1(qm150)* mutant.

**Table S1** Quantitative data of individual lifespan experiments.

**Table S2** Genes that are differentially expressed between *isp‐1(qm150)* and *ceh‐23(ms23); isp‐1(qm150)* (identified by Statistical Analysis of Microarray (SAM) 1 class analysis with false discovery rate (FDR) = 0.59%, 1.5 fold change cutoff).

**Table S3** Genes that are commonly regulated by CEH‐23 and CEP‐1 in the *isp‐1* mutant (identified by Statistical Analysis of Microarray (SAM) 1 class analysis with false discovery rate (FDR) = 0, 1.5 fold change cutoff).

**Table S4** Genes that are differentially expressed between *aak‐2ca* strain and transgenic control strain. (Data from Mair *et al*. and reanalyzed using SAM analysis. Gene list was identified by SAM 1 class analysis with FDR = 0, 1.5 fold change cutoff.)

**Table S5** Genes that are commonly regulated by CEH‐23, CEP‐1 and AAK‐2 (identified by overlap between gene lists in Table S3 and S4).

**Data S1** Supplemental experimental procedures.
