# Supplementary figures and images for: Transcription factors CEP‐1/p53 and CEH‐23 collaborate with AAK‐2/AMPK to modulate longevity in Caenorhabditis elegans
Source: Aging Cell. 2017 May 30;16(4):814–24. doi: 10.1111/acel.12619 (PMC5506430; doi:10.1111/acel.12619)

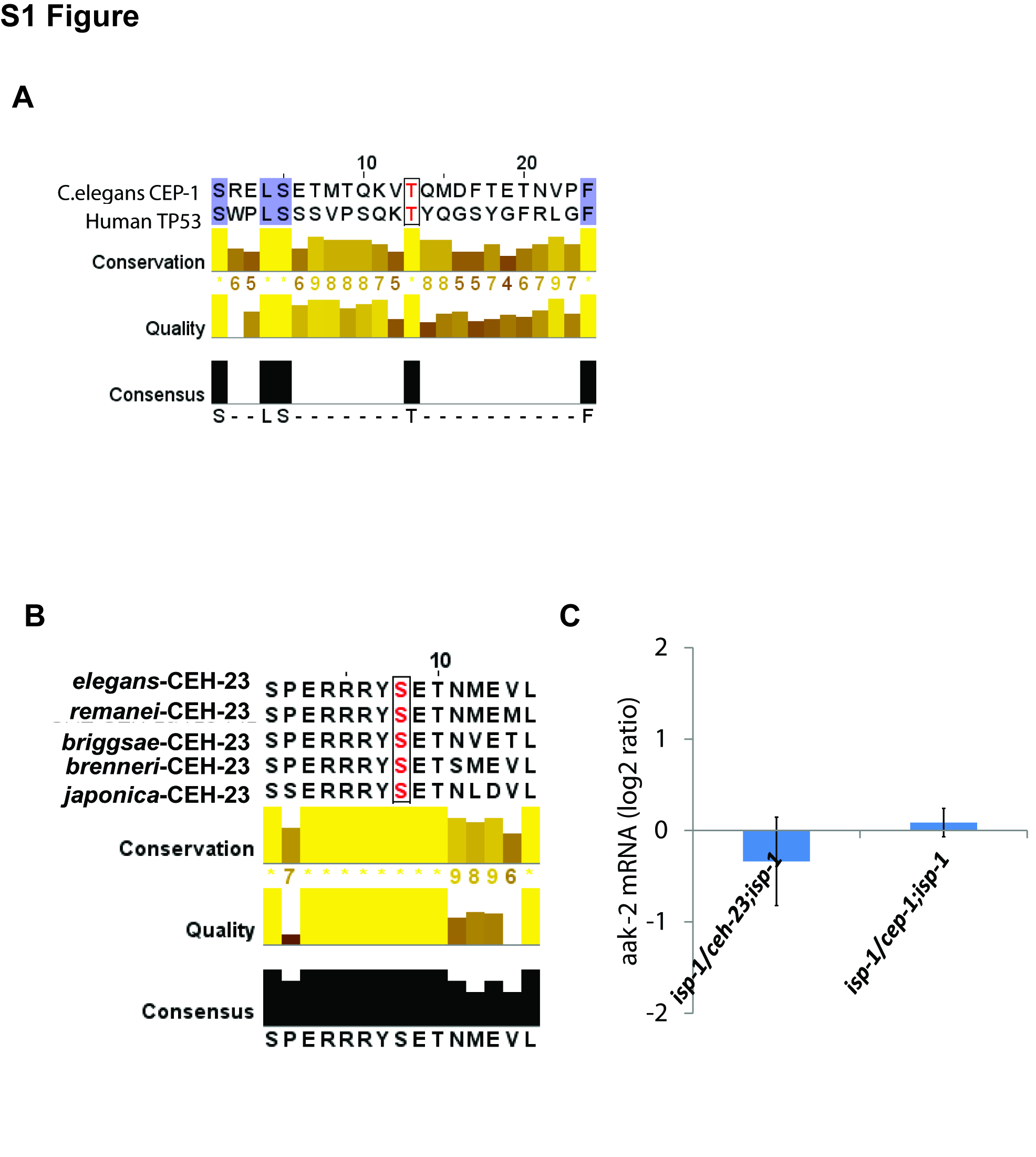

Supplement: Supplementary file 1 — Fig. S1 CEH‐23 and CEP‐1 harbor putative AMPK phosphorylation sites. [file ACEL-16-814-s001.tif]

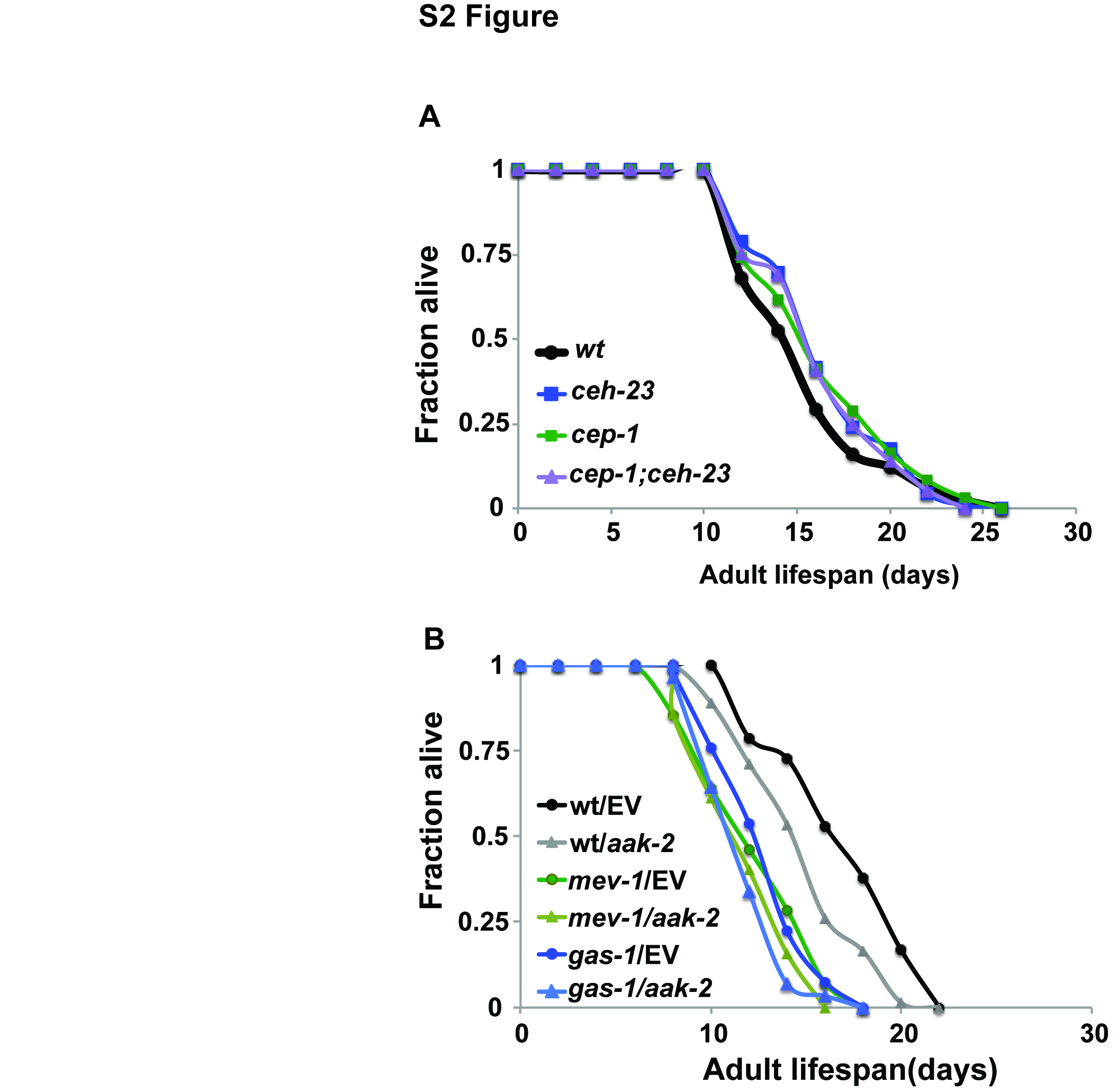

Supplement: Supplementary file 2 — Fig. S2 (A) Wild‐type C. elegans lifespan was not altered by the combined loss of ceh‐23 and cep‐1. The ceh‐23(ms23) and cep‐1(gk138) single and double mutants were used in the lifespan analysis. (B) aak‐2 RNAi had little effect on the lifespan of the short‐lived gas‐1(fc21) and mev‐1(kn1) mutants. EV: empty vector RNAi control. [file ACEL-16-814-s002.tif]

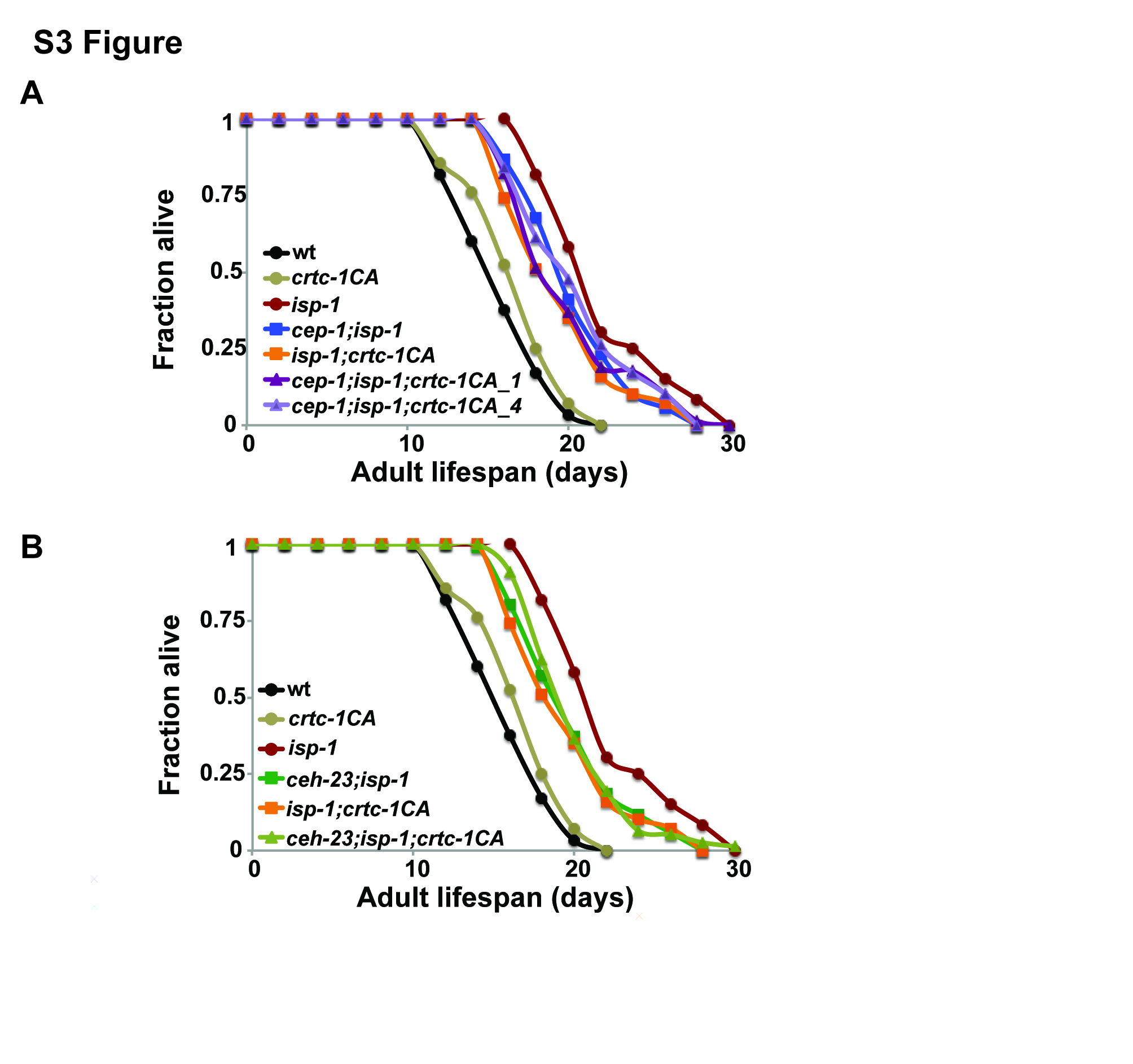

Supplement: Supplementary file 3 — Fig. S3 CRTC‐1 antagonizes CEH‐23 and CEP‐1 to modulate the lifespan of isp‐1(qm150) mutant. [file ACEL-16-814-s003.tif]
