# Supplementary material for: Transcription factors CEP‐1/p53 and CEH‐23 collaborate with AAK‐2/AMPK to modulate longevity in Caenorhabditis elegans
Source: Aging Cell. 2017 May 30;16(4):814–24. doi: 10.1111/acel.12619 (PMC5506430; doi:10.1111/acel.12619)
Supplement: Supplementary file 4 — Table S1 Quantitative data of individual lifespan experiments. [file ACEL-16-814-s004.pdf]

Table S1: Adult lifespan of all individual experiments.

A. Epistasis between *ceh-23(ms23)* and *cep-1(gk138)* on *isp-1(qm150)* longevity

A.1 LS32

Strain	N (post censoring)	mean LS ± SEM (days)	% change to wt	median LS ± SEM (days)	% change to wt	*P*-value *vs wt*	*P*-value *vs ceh-23;isp-1*	*P*-value *vs cep-1;isp-1*
WT(N2)	97	20.21±0.21		20±0.18			1.97E-24	5.81E-24
isp-1(qm150)	91	27.72±0.31	37.19	28±0.36	40.00	5.81E-24	4.59E-04	1.17E-07
ceh-23(ms23);isp-1(qm150)	96	25.44±0.40	25.89	24±0.45	20.00	3.59E-38		3.04E-01
cep-1(gk138);isp-1(qm150)(qm150)	96	25.00±0.33	23.66	24±0.40	20.00	1.97E-24	3.04E-01	
cep-1(gk138);ceh-23(ms23);isp-1(qm150) iso.2	88	23.64±0.32	16.99	24±0.36	20.00	5.03E-16	2.68E-04	2.08E-03
cep-1(gk138);ceh-23(ms23);isp-1(qm150) iso.22	75	24.00±0.41	18.78	24±0.44	20.00	6.44E-15	3.57E-02	9.12E-02

A.2 LS35

Strain	N (post censoring)	mean LS ± SEM (days)	% change to wt	median LS ± SEM (days)	% change to wt	*P*-value *vs wt*	*P*-value *vs ceh-23;isp-1*	*P*-value *vs cep-1;isp-1*
WT (N2)	100	19.5±0.19		20±0.21			2.43E-26	2.80E-28
isp-1(qm150)	97	28.52±0.3	46.23	28±0.37	40.00	2.30E-46	3.54E-17	4.48E-21
ceh-23(ms23);isp-1(qm150)	92	24.35±0.34	24.86	24±0.47	20.00	2.43E-26		3.29E-01
cep-1(gk138);isp-1(qm150)	100	24.18±0.28	24.02	24±0.38	20.00	2.80E-28	3.29E-01	
cep-1(gk138);ceh-23(ms23);isp-1(qm150) iso. 1	86	24.4±0.34	25.12	24±0.5	20.00	1.01E-25	9.31E-01	3.16E-01
cep-1(gk138);ceh-23(ms23);isp-1(qm150) iso. 2	77	24.42±0.36	25.21	24±0.7	20.00	3.54E-23	7.36E-01	2.31E-01
cep-1(gk138);ceh-23(ms23);isp-1(qm150) iso. 3	85	24.5±0.36	25.62	24±0.64	20.00	1.17E-24	4.94E-01	1.14E-01

B. Requirement of CEH-23 and CEP-1 in various ETC mutants

B.1 LS33

Strain	N (post censoring)	mean LS ± SEM (days)	% change to wt	median LS ± SEM (days)	% change to wt	*P*-value *vs wt*	*P*-value *vs nuo-6(qm200)*
WT(N2)	106	18.85±0.25		20±0.25			1.01E-32
nuo-6(qm200)	80	30.88±0.67	63.84	30±0.78	50.00	1.01E-32	
nuo-6(qm200);ceh-23(ms23) iso.1	98	27.99±0.74	48.52	28±0.66	40.00	6.36E-21	5.22E-03
nuo-6(qm200);ceh-23(ms23) iso.2	104	25.29±0.57	34.20	26±0.38	30.00	8.17E-20	4.13E-09
nuo-6(qm200);ceh-23(ms23) iso.3	107	27.5±0.59	45.92	28±0.55	40.00	6.06E-23	2.89E-04

B.2 LS42

Strain	N (post censoring)	mean LS ± SEM (days)	% change to wt	median LS ± SEM (days)	% change to wt	*P*-value *vs wt*	*P*-value *vs nuo-6(qm200)*
WT(N2)	98	14.96±0.29		14±0.38			9.30E-02
nuo-6(qm200)	100	16.1±1.38	7.61	12±0.88	-14.29	9.30E-02	
nuo-6(qm200) cep-1(gk138) iso.1	93	15.3±1.12	2.24	16±1.64	40.00	3.87E-01	4.50E-01
nuo-6(qm200) cep-1(gk138) iso.2	90	13.78±0.6	-7.89	14±0.84	30.00	9.60E-02	2.50E-01
nuo-6(qm200);ceh-23(ms23)	94	16.46±1.22	10.02	12±1.07	40.00	3.30E-02	9.98E-01

B.3 LS45

Strain	N (post censoring)	mean LS ± SEM (days)	% change to wt	median LS ± SEM (days)	% change to wt	*P*-value *vs wt*	*P*-value *vs nuo-6(qm200)*	*P*-value *vs mev-1(kn1)*	*P*-value *vs gas-1(fc21)*
WT(N2)	103	16.97±0.34		16±0.38			1.79E-24	1.77E-07	3.44E-15
nuo-6(qm200)	153	22.85±0.33	34.65	24±0.49	50.00	1.79E-24			
nuo-6(qm200) cep-1(gk138) iso.1	194	18.51±0.42	9.07	18±0.84	12.50	1.28E-04	1.05E-08		
nuo-6(qm200) cep-1(gk138) iso.2	123	15.88±0.46	-6.42	14±0.39	-12.50	2.52E-01	3.11E-21		

nuo-6(qm200);ceh-23(ms23)	174	18.46±0.82	8.78	20±1.9	25.00	1.27E-03	1.75E-03		
mev-1(kn1)	108	14±0.36	-17.50	18±0.17	-12.50	1.77E-07			
ceh-23(ms23) mev-1(kn1)iso.1	107	15.86±0.37	-6.54	16±0.5	0.00	7.79E-02		5.34E-04	
ceh-23(ms23) mev-1(kn1)iso.2	96	15.21±0.41	-10.37	16±0.56	0.00	5.66E-03		2.16E-02	
cep-1(gk138);mev-1(kn1)	105	19.84±0.49	16.91	18±0.95	12.50	1.35E-07		1.79E-17	
gas-1(fc21)	106	13.07±0.28	-22.98	14±0.27	-12.50	3.44E-15			
ceh-23(ms23);gas-1(fc21) iso.1	41	13.46±0.58	-20.68	12±0.9	-25.00	6.60E-07			3.42E-01
ceh-23(ms23);gas-1(fc21) iso.2	82	16.15±0.48	-4.83	16±0.53	0.00	3.78E-01			3.09E-09
cep-1(gk138);gas-1(fc21)	55	16.53±0.7	-2.59	16±0.91	0.00	7.51E-01			2.02E-07

B.4 LS47

Strain	N (post censoring)	mean LS ± SEM (days)	% change to wt	median LS ± SEM (days)	% change to wt	*P*-value *vs wt*	*P*-value *vs mev-1(kn1)*	*P*-value *vs gas-1(fc21)*
WT(N2)	108	16.18±0.27		16±0.33			6.05E-04	3.54E-08
cep-1(gk138)	89	17.63±0.32	8.96	18±0.52	12.50	3.80E-04		
ceh-23(ms23)	104	16.04±0.28	-0.87	16±0.31	0.00	7.36E-01		
mev-1(kn1)	107	13.92±0.38	-13.97	14±0.78	-12.50	6.05E-04		
cep-1(gk138);mev-1(kn1)	89	17.73±0.43	9.58	18±0.6	12.50	9.64E-04	1.59E-08	
ceh-23(ms23) mev-1(kn1)iso.1	106	17.85±0.53	10.32	18±0.67	12.50	4.00E-04	7.13E-10	
ceh-23(ms23) mev-1(kn1)iso.2	99	20.48±0.48	26.58	22±0.64	37.50	2.54E-13	1.67E-21	
ceh-23(ms23) mev-1(kn1)iso.3	88	21.92±0.56	35.48	24±0.72	50.00	1.28E-15	1.26E-22	
gas-1(fc21)	90	13.31±0.35	-17.74	12±0.48	-25.00	3.54E-08		
cep-1(gk138);gas-1(fc21)	101	18.82±0.55	16.32	20±0.92	25.00	1.98E-07		4.80E-16
ceh-23(ms23);gas-1(fc21) iso.1	93	17.46±0.45	7.91	18±0.53	12.50	4.38E-04		1.58E-12
ceh-23(ms23);gas-1(fc21) iso.2	72	15.26±0.63	-5.69	16±0.62	0.00	8.90E-01		6.89E-04
ceh-23(ms23);gas-1(fc21) iso.3	88	14.4±0.62	-11.00	12±1.41	-25.00	6.09E-01		1.21E-02

B.5 LS49

Strain	N (post censoring)	mean LS ± SEM (days)	% change to wt	median LS ± SEM (days)	% change to wt	*P*-value *vs wt*	*P*-value *vs gas-1(fc21)*
WT(N2)	98	17.44±0.37		18±0.63			3.12E-21
ceh-23(ms23)	100	17.45±0.41	0.06	18±0.8	0.00	6.71E-01	2.59E-20
cep-1(gk138)	100	18.46±0.34	5.85	18±0.53	0.00	2.97E-02	1.51E-27
gas-1(fc21)	84	11.81±0.32	-32.28	12±0.39	-33.33	3.12E-21	
ceh-23(ms23);gas-1(fc21) iso.1	80	18.9±0.56	8.37	20±0.89	11.11	1.90E-03	4.57E-20
cep-1(gk138);gas-1(fc21)	117	18.07±0.56	3.61	18±0.8	0.00	8.73E-02	1.80E-17

C. Longevity effects of knockdown *aak-2* on the *isp-1;cep-1* and *isp-1;ceh-23* mutants

C.1 LS51

Strain/treatment	N (post censoring)	mean LS ± SEM (days)	% change to wt/control	median LS ± SEM (days)	% change to wt/control	P-value vs wt/control	% change to isp-1/control (mean LS)	*P*-value *vs isp-1/control*	*% change to L4440 control*	*P*-value *vs control treatment*
N2/L4440	92	16.81±0.36		16±0.41			-29.50			
N2/cep-1	73	16.58±0.39	-1.37	16±0.44	0.00	6.73E-01	-30.47	6.73E-01	-1.382	6.73E-01
N2/aak-2	81	14.49±0.36	-13.80	14±0.68	-12.50	1.10E-05	-39.24	1.10E-05	-13.812	1.10E-05
isp-1(qm150)/L4440	25	23.84±1.12	41.82	24±2	50.00	5.15E-10		5.15E-10		
isp-1(qm150)/cep-1	70	18.86±0.58	12.20	18±0.64	12.50	6.16E-04	-20.90	6.16E-04	-20.901	2.32E-05
isp-1(qm150)/aak-2	43	14.89±0.66	-11.42	14±0.99	-12.50	7.59E-02	-37.55	7.59E-02	-37.547	2.14E-09
cep-1(gk138);isp-1(qm150)/L4440	73	19.47±0.46	15.82	19±0.6	18.75	1.17E-05	-18.35	1.17E-05		
cep-1(gk138);isp-1(qm150)/cep-1	68	20.24±0.43	20.40	19±0.59	18.75	5.54E-08	-15.12	5.54E-08	3.953	3.91E-01
cep-1(gk138);isp-1(qm150)/aak-2	47	17.22±0.54	2.44	17±0.81	6.25	4.88E-01	-27.76	4.88E-01	-11.530	1.10E-03
ceh-23(ms23);isp-1(qm150)/L4440	61	19.12±0.63	13.74	18±0.63	12.50	4.05E-04	-19.80	4.05E-04		
ceh-23(ms23);isp-1(qm150)/cep-1	79	19.01±0.47	13.09	18±0.51	12.50	1.69E-04	-20.25	1.69E-04	-.565	5.35E-01
ceh-23(ms23);isp-1(qm150)/aak-2	69	17.33±0.41	3.09	16±0.55	0.00	2.61E-01	-27.29	2.61E-01	-9.348	6.34E-03

C.2 LS52

Strain/treatment	N (post censoring)	mean LS ± SEM (days)	% change to wt/control	median LS ± SEM (days)	% change to wt/control	P-value vs wt/control	% change to isp-1/control (mean	*P-value vs isp-1/control*	*% change to L4440*	*P-value vs control*
N2/L4440	91	18.17±0.43		18±0.71			-31.71	9.96E-27		
N2/cep-1	97	17.94±0.38	-1.29	18±0.61	0.00	4.05E-01	-32.59	3.93E-30	-1.290	4.05E-01
N2/aak-2	88	14.18±0.31	-21.97	14±0.39	-22.22	7.70E-13	-46.72	1.33E-46	-21.974	7.70E-13
isp-1(qm150)/L4440	91	26.61±0.49	46.43	26±0.58	44.44	9.96E-27				
isp-1(qm150)/cep-1	87	21.59±0.35	18.81	22±0.49	22.22	4.33E-07	-18.86	1.34E-13	-18.861	1.34E-13
isp-1(qm150)/aak-2	85	17.2±0.48	-5.37	16±0.73	-11.11	2.47E-01	-35.38	1.37E-28	-35.376	1.37E-28
*cep-1(gk138);isp-1(qm150)/*L4440	78	24.16±0.36	32.93	23±0.38	27.78	4.59E-19	-9.22	1.70E-05		
cep-1(gk138);isp-1(qm150)/cep-1	85	24.41±0.33	34.31	25±0.48	38.89	3.79E-22	-8.28	5.07E-05	1.041	7.61E-01
cep-1(gk138);isp-1(qm150)/aak-2	90	19.17±0.5	5.51	19±0.90	5.56	1.44E-02	-27.94	5.72E-20	-20.626	7.53E-11
*ceh-23(ms23);isp-1(qm150)/*L4440	101	20.49±0.36	12.76	20±0.58	11.11	3.91E-04	-23.00	4.33E-18		
ceh-23(ms23);isp-1(qm150)/cep-1	87	21.04±0.36	15.77	20±0.58	11.11	1.98E-05	-20.94	1.40E-15	2.669	4.42E-01
ceh-23(ms23);isp-1(qm150)/aak-2	101	17.48±0.38	-3.80	18±0.55	0.00	1.07E-01	-34.30	8.12E-33	-14.682	2.79E-07

C.3 LS56

Strain/treatment	N (post censoring)	mean LS ± SEM (days)	% change to N2	median LS ± SEM (days)	% change to wt/control	P-value vs wt/control	% change to isp-1 (mean LS)	*P-value vs isp-1*	*% change to isp-1;aak-*	*P-value vs isp-1;aak-2*
N2	92	17.11±0.24		16±0.37			-19.59	2.03E-12	2.75	2.62E-01
isp-1(qm150)	83	21.28±0.50	24.36	20±0.69	25.00	2.03E-12			27.79	7.25E-14
isp-1(qm150);aak-2(ok524)	80	16.65±0.27	-2.68	16±0.32	0.00	2.62E-01	-21.75	7.25E-14		
ceh-23(ms23);isp-1(qm150);aak-2(ok524)_1	78	17.03±0.31	-0.49	16±0.40	0.00	9.29E-01	-19.98	2.12E-11	2.26	3.09E-01
ceh-23(ms23);isp-1(qm150);aak-2(ok524)_2	66	17.00±0.35	-0.64	16±0.47	0.00	9.33E-01	-20.10	1.16E-10	2.10	3.32E-01
cep-1(gk138);isp-1(qm150);aak-2(ok524)_1	60	17.07±0.35	-0.25	16±0.55	0.00	8.62E-01	-19.79	7.90E-10	2.50	2.92E-01
cep-1(gk138);isp-1(qm150);aak-2(ok524)_2	69	17.13±0.29	0.13	18±0.35	12.50	8.89E-01	-19.49	7.12E-11	2.89	2.57E-01

D. Requirement of CEH-23 and CEP-1 on longevity of the AAK-2 ca

D.1 LS53

Strains	N (post censoring)	mean LS ± SEM (days)	% change to WBM59	median LS ± SEM (days)	% change to WBM59	P-value vsWBM59	P-value vsWBM60
RFP control (WBM59)	109	16.08±0.39		16±0.48			9.18E-12
aak-2 ca (WBM60)	107	20.56±0.46	27.86	20±0.77	25.00	9.18E-12	
WBM59; *ceh-23(ms23)*_1	105	17.11±0.35	6.41	18±0.46	12.50	2.10E-01	3.99E-09
WBM59;*ceh-23(ms23)*_2	105	17.2±0.4	6.97	16±0.53	0.00	5.70E-02	1.07E-07
WBM60;*ceh-23(ms23)*_1	108	16.24±0.4	1.00	16±0.47	0.00	8.41E-01	7.18E-11
WBM60;*ceh-23(ms23)*_2	110	16.94±0.43	5.35	16±0.42	0.00	2.52E-01	2.02E-08
WBM60;*cep-1(gk138)*_1	107	19.65±0.36	22.20	20±0.65	25.00	1.92E-08	2.87E-02
WBM60;*cep-1(gk138)*_2	104	19.34±0.35	20.27	18±0.54	12.50	1.92E-07	6.09E-03

D.2 LS54

Strains	N (post censoring)	mean LS ± SEM (days)	% change to WBM59	median LS ± SEM (days)	% change to WBM59	P-value vsWBM59	P-value vsWBM60
RFP control (WBM59)	106	16.63±0.35		16±0.45			7.43E-15
aak-2 ca (WBM60)	120	22.55±0.59	35.60	22±1	37.50	7.43E-15	
WBM59; *ceh-23(ms23)*_1	107	16.54±0.38	-0.54	16±0.48	0.00	9.11E-01	6.31E-15
WBM59;*ceh-23(ms23)*_2	92	17.66±0.45	6.19	18±0.48	12.50	7.11E-02	1.07E-09
WBM60;*ceh-23(ms23)*_1	104	18.21±0.45	9.50	18±0.57	12.50	4.56E-03	9.83E-09
WBM60;*ceh-23(ms23)*_2	117	17.19±0.43	3.37	18±0.46	12.50	2.32E-01	1.55E-11
WBM60;*cep-1(gk138)*_1	98	17.64±0.41	6.07	18±0.34	12.50	4.35E-02	1.50E-10
WBM60;*cep-1(gk138)*_2	115	19.71±0.46	18.52	20±0.5	25.00	3.74E-07	1.53E-04

E. Epistasis between *ceh-23, cep-1* and *crtc-1* on *isp-1(qm150)* longevity

E.1 LS57

Strains	N (post censoring)	mean LS ± SEM (days)	% change to wt/control	median LS ± SEM (days)	% change to wt/control	P-value vs wt/control	% change to isp-1/control (mean	*P*-value *vs isp-1/control*	*% change to L4440*	*P*-value *vs control*
N2/L4440	117	16.02±0.43		16±0.54			-23.42	2.07E-09		
N2/*ceh-23*	73	15.86±0.47	-0.96	16±0.49	0.00	4.39E-01	-24.15	2.52E-09	-0.96	4.39E-01
N2/*cep-1*	107	14.80±0.36	-7.58	14±0.489	-12.50	5.64E-03	-29.22	9.51E-15	-7.58	5.64E-03
crtc-1 CA/L4440	99	17.57±0.35	9.68	18±0.53	12.50	1.56E-01	-16.00	7.67E-07		
crtc-1 CA/*ceh-23*	98	16.78±0.43	4.74	16±0.66	0.00	5.11E-01	-19.79	7.46E-08	-4.51	5.02E-01
crtc-1 CA/*cep-1*	83	16.80±0.45	4.86	16±0.57	0.00	5.97E-01	-19.70	1.94E-07	-4.40	4.36E-01
isp-1(qm150)/L4440	70	20.91±0.67	30.57	18±0.93	12.50	2.07E-09				
isp-1(qm150)/ceh-23	92	18.5±0.42	15.50	18±0.41	12.50	4.52E-04	-11.54	2.71E-04	-11.54	2.71E-04
isp-1(qm150)/cep-1	79	16.99±0.55	6.06	16±0.48	0.00	1.03E-01	-18.78	1.96E-05	-18.78	1.96E-05
isp-1(qm150);crtc-1 CA iso2/L4440	89	18.63±0.56	16.31	18±0.47	12.50	2.19E-04	-10.93	1.38E-02		
isp-1(qm150);crtc-1CA iso2/*ceh-23*	88	18.93±0.51	18.20	18±0.42	12.50	3.99E-05	-9.48	1.13E-02	1.62	8.76E-01
isp-1(qm150);crtc-1CA iso2/*cep-1*	63	18.83±0.74	17.53	18±0.86	12.50	2.95E-04	-9.99	4.53E-02	1.05	7.72E-01
isp-1(qm150);crtc-1 CA iso1/L4440	45	17.65±0.75	10.21	16±0.95	0.00	4.51E-02	-15.60	6.05E-04		
isp-1(qm150);crtc-1CA iso1/*ceh-23*	28	20.29±1.048	26.65	18±1.76	12.50	8.56E-05	-3.01	4.43E-01	14.92	2.62E-02
isp-1(qm150);crtc-1CA iso1/*cep-1*	47	19.12±0.82	19.37	18±0.83	12.50	4.72E-04	-8.58	1.13E-01	8.32	1.12E-01

E.2 LS60

Strains	N (post censoring)	mean LS ± SEM (days)	% change to wt/control	median LS ± SEM (days)	% change to wt/control	P-value vs wt/control	% change to isp-1/control (mean	*P*-value *vs isp-1/control*	*% change to L4440*	*P*-value *vs control*
N2/L4440	127	16.25± 0.38		16± 0.74			-32.70	9.03E-24		
N2/*ceh-23*	131	16.77± 0.40	3.19	16± 0.71	0.00	2.77E-01	-30.55	3.72E-20	3.19	2.77E-01
N2/*cep-1*	99	17.45± 0.45	7.40	18± 0.50	12.50	7.02E-02	-27.71	5.65E-17	7.40	7.02E-02
crtc-1 CA/L4440	85	20.56± 0.38	26.48	20± 0.59	25.00	5.34E-09	-14.87	4.93E-08		
crtc-1 CA/*ceh-23*	71	19.89± 0.43	22.37	20± 0.65	25.00	7.59E-06	-17.64	3.43E-09	-3.25	2.59E-01
crtc-1 CA/*cep-1*	69	19.38± 0.44	19.26	20± 0.60	25.00	1.00E-04	-19.73	9.73E-11	-5.70	6.94E-02
isp-1(qm150)/L4440	90	24.15± 0.50	48.58	24± 0.71	50.00	9.03E-24	0.00			
isp-1(qm150)/ceh-23	81	20.74± 0.27	27.62	20± 0.26	25.00	2.80E-10	-14.11	5.31E-09	-14.11	5.31E-09
isp-1(qm150)/cep-1	77	20.93± 0.32	28.79	20± 0.37	25.00	9.45E-11	-13.32	1.95E-07	-13.32	1.95E-07
isp-1(qm150);crtc-1 CA iso1/L4440	63	21.15± 0.41	30.15	20± 0.46	25.00	6.06E-11	-12.41	9.89E-06		
isp-1(qm150);crtc-1CA iso1/ceh-23	77	21.23± 0.36	30.63	20± 0.35	25.00	7.45E-12	-12.08	4.37E-06	0.37	9.67E-01
isp-1(qm150);crtc-1CA iso1/cep-1	81	21.00± 0.33	29.23	20± 0.50	25.00	2.06E-11	-13.02	3.06E-07	-0.70	5.54E-01
isp-1(qm150);crtc-1 CA iso2/L4440	74	21.67± 0.41	33.34	22± 0.42	37.50	3.52E-13	-10.26	1.31E-04		
isp-1(qm150);crtc-1CA iso2/ceh-23	61	21.12± 0.46	29.98	20± 0.51	25.00	9.60E-10	-12.52	2.18E-05	-2.52	1.40E-03
isp-1(qm150);crtc-1CA iso2/cep-1	91	22.28± 0.38	37.07	22± 0.58	37.50	3.14E-17	-7.74	9.35E-04	2.80	5.27E-04

E.3 LS61

Strains	N (post censoring)	mean LS ± SEM (days)	% change to wt	median LS ± SEM (days)	% change to wt	P-value vs wt/control	% change to isp-1 (mean LS)	*P*-value *vs isp-1*	*% change to isp-1;crtc-*	*P*-value *vsisp-1;crtc-1CA*
N2	93	16.00± 0.29		16.00± 0.45			-28.54	3.35E-24	-19.48	1.24E-11
crtc-1CA	84	16.93± 0.32	5.81	18.00± 0.37	12.50	3.58E-02	-24.38	6.73E-19	-14.80	2.25E-07
isp-1(qm150)	72	22.39± 0.43	39.93	22.00± 0.39	37.50	3.35E-24			12.68	1.63E-04
ceh-23(ms23);isp-1(qm150)	86	20.20± 0.37	26.25	20.00± 0.53	25.00	8.68E-15	-9.78	3.06E-04	1.66	6.88E-01
cep-1(gk138);isp-1(qm150)	90	20.69± 0.34	29.31	20.00± 0.39	25.00	1.01E-18	-7.59	2.18E-03	4.13	2.56E-01
isp-1(qm150);crtc-1CA	69	19.87± 0.43	24.19	20.00± 0.58	25.00	1.24E-11	-11.25	1.63E-04		
cep-1(gk138);isp-1(qm150)crtc-1CA iso1	68	20.38± 0.47	27.38	20.00± 0.51	25.00	1.52E-12	-8.97	3.72E-03	2.57	3.91E-01
cep-1(gk138);isp-1(qm150)crtc-1CA iso2	88	20.93± 0.40	30.81	20.00± 0.60	25.00	3.20E-18	-6.52	1.87E-02	5.33	9.76E-02
ceh-23(ms23);isp-1(qm150)crtc-1CA	78	20.68± 0.41	29.25	20.00± 0.43	25.00	4.09E-16	-7.63	1.07E-02	4.08	2.25E-01
aak-2 ca (WBM60)	107	20.37± 0.40	27.31	20.00± 0.34	25.00	2.73E-16	-9.02	2.40E-03	2.52	3.13E-01
WBM60;*crtc-1CA*	87	18.22± 0.30	13.88	18.00± 0.54	12.50	1.46E-06	-18.62	3.38E-12	-8.30	1.59E-03

pooled data

Figure 1A

Strain	N (post censoring)	mean LS ± SEM (days)	% change to wt	median LS ± SEM (days)	% change to wt	*P*-value *vs wt*	*P*-value *vs ceh-23;isp-1*	*P*-value *vs cep-1;isp-1*
WT(N2)	404	17.611±0.161		18±0.2			1.48E-86	2.19E-95
isp-1(qm150)	333	27.668±0.246	57.11	28±0.26	55.56	4.26E-136	4.39E-37	5.05E-25
ceh-23(ms23);isp-1(qm150)	410	23.479±0.203	33.32	24±0.28	33.33	1.48E-86		3.91E-03
cep-1(gk138);isp-1(qm150)	339	24.329±0.221	38.15	24±0.27	33.33	2.19E-95	3.91E-03	
cep-1(gk138);ceh-23(ms23);isp-1(qm150)	560	24.379±0.155	38.43	24±0.25	33.33	6.59E-137	2.52E-03	5.29E-01

Figure 1B

Strain	N (post censoring)	mean LS ± SEM (days)	% change to wt	median LS ± SEM (days)	% change to wt	*P*-value *vs wt*	*P*-value *vs nuo-6(qm200)*
WT(N2)	188	16.05±0.24		16±0.28			7.04E-30
nuo-6(qm200)	143	21.59±0.43	34.51	22±0.52	37.50	7.04E-30	
nuo-6(qm200) cep-1(gk138)	275	17.09±0.3	6.46	16±0.45	0.00	2.22E-04	6.75E-14
nuo-6(qm200);ceh-23(ms23)	90	17.95±0.68	11.80	18±1.15	12.50	2.11E-05	1.02E-03

Figure 1C and D

Strain	N (post censoring)	mean LS ± SEM (days)	% change to wt	median LS ± SEM (days)	% change to wt	*P*-value *vs wt*	*P*-value *vs mev-1(kn1)*	*P*-value *vs gas-1(fc21)*
WT(N2)	513	16.97±0.15		16±0.24			1.77E-07	3.44E-15
mev-1(kn1)	215	13.96±0.26	-17.71	14±0.29	-12.50	1.77E-07		
cep-1(gk138);mev-1(kn1)	194	18.94±0.35	11.65	18±0.52	12.50	1.35E-07	4.98E-04	
ceh-23(ms23) mev-1(kn1)	496	17.97±0.24	5.92	18±0.28	12.50	1.48E-02	1.79E-17	
gas-1(fc21)	280	12.78±0.19	-24.67	12±0.23	-25.00	3.44E-15		
cep-1(gk138);gas-1(fc21)	273	17.97±0.35	5.91	18±0.52	12.50	2.04E-07		8.07E-07
ceh-23(ms23);gas-1(fc21)	456	16.15±0.24	-4.84	16±0.36	0.00	9.97E-01		2.02E-07

Figure 4D and E

Strain/RNAi treatment	N (post censoring)	mean LS ± SEM (days)	% change to wt/control	median LS ± SEM (days)	% change to wt/control	P-value vs wt/control	*P*-value *vs isp-1/control*	P-value vs. L4440
N2/L4440	203	17.49±0.28		16±0.48			4.89E-40	
N2/aak-2	185	14.33±0.24	-18.07	14±0.35	-12.50	8.28E-17	1.60E-58	8.28E-17
isp-1(qm150)/L4440	162	26.04±0.46	48.89	26±0.57	62.50	4.89E-40		
isp-1(qm150)/aak-2	198	16.38±0.41	-6.35	16±0.54	0.00	2.56E-01	4.57E-36	4.57E-36
cep-1(gk138);isp-1(qm150)/L4440	204	21.93±0.34	25.39	23±0.46	43.75	1.92E-21	1.73E-12	
cep-1(gk138);isp-1(qm150)/aak-2	175	18.51±0.39	5.83	19±0.69	18.75	2.78E-03	2.78E-28	6.92E-10
ceh-23(ms23);isp-1(qm150)/L4440	199	19.98±0.33	14.24	20±0.51	25.00	2.70E-08	2.67E-21	
ceh-23(ms23);isp-1(qm150)/aak-2	216	17.45±0.28	-0.23	18±0.38	12.50	7.86E-01	1.33E-39	9.34E-09

Figure 5B

Strains	N (post censoring)	mean LS ± SEM (days)	% change to WBM59	median LS ± SEM (days)	% change to WBM59	P-value vsWBM59	P-value vsWBM60
RFP control (WBM59)	215	16.34±0.27		16±0.33			8.33E-26
aak-2 ca (WBM60)	227	21.53±0.38	31.76	22±0.76	37.50	8.33E-26	
WBM59; *ceh-23(ms23)*	409	17.12±0.2	4.77	16±0.26	0.00	2.55E-02	1.10E-25
WBM60;*ceh-23(ms23)*	439	17.15±0.22	4.96	16±0.27	0.00	1.77E-02	1.03E-22
WBM60;*cep-1(gk138)*	424	19.15±0.2	17.20	18±0.24	12.50	1.08E-14	1.10E-09

Figure 6B and C

Strains	N (post censoring)	mean LS ± SEM (days)	% change to wt/control	median LS ± SEM (days)	% change to wt/control	P-value vs wt/control	% change to isp-1/control (mean	*P*-value *vs isp-1/control*	*% change to L4440*	*P*-value *vs control*
N2/L4440	244	16.14±0.28		16±0.44			-29.16	1.50E-30		
N2/*ceh-23*	204	16.44±0.31	1.88	16±0.43	0.00	5.56E-01	-27.82	2.56E-27	1.88	5.56E-01
N2/*cep-1*	206	16.08±0.30	-0.34	16±0.57	0.00	6.76E-01	-29.40	9.56E-31	-0.34	6.76E-01
crtc-1 CA/L4440	184	18.96±0.28	17.48	18±0.41	12.50	9.08E-08	-16.78	7.16E-15		

crtc-1 CA/*ceh-23*	169	18.10±0.33	12.13	18±0.50	12.50	3.97E-04	-20.56	1.65E-17	-4.55	1.38E-01
crtc-1 CA/*cep-1*	152	18.00±0.33	11.56	18±0.40	12.50	1.38E-03	-20.97	7.87E-18	-5.04	6.29E-02
isp-1(qm150)/L4440	160	22.78±0.42	41.16	22±0.48	37.50	1.50E-30				
isp-1(qm150)/ceh-23	173	19.59±0.27	21.40	20±0.33	25.00	1.64E-11	-13.99	6.19E-12	-13.99	6.19E-12
isp-1(qm150)/cep-1	156	19.09±0.35	18.28	18±0.47	12.50	4.42E-09	-16.20	3.73E-11	-16.20	3.73E-11
isp-1(qm150);crtc-1 CA iso1/L4440	152	19.79±0.37	22.63	18±0.43	12.50	1.07E-12	-13.13	1.84E-07		
isp-1(qm150);crtc-1CA iso1/ceh-23	165	20.06±0.33	24.27	20±0.35	25.00	1.32E-14	-11.96	9.40E-08	1.34	8.77E-01
isp-1(qm150);crtc-1CA iso1/cep-1	144	20.20±0.37	25.15	20±0.51	25.00	1.89E-14	-11.34	1.91E-06	2.06	5.81E-01
isp-1(qm150);crtc-1 CA iso2/L4440	119	20.32±0.41	25.92	20±0.46	25.00	3.02E-13	-10.79	1.31E-05		
isp-1(qm150);crtc-1CA iso2/ceh-23	89	20.92±0.45	29.59	20±0.56	25.00	1.14E-13	-8.19	9.24E-04	2.92	4.47E-01
isp-1(qm150);crtc-1CA iso2/cep-1	138	21.24±0.39	31.61	22±0.49	37.50	1.10E-19	-6.76	1.83E-03	4.52	1.09E-01

Figure S2A

Strain	N (post censoring)	mean LS ± SEM (days)	% change to wt	median LS ± SEM (days)	% change to wt	*P*-value *vs wt*
WT(N2)	83	15.73±0.41		16±0.51		
ceh-23(ms23)	113	16.74±0.34	6.451898114	16±0.33	.000	1.18E-01
cep-1(gk138)	97	16.68±0.41	6.051668118	16±0.48	.000	1.30E-01
cep-1(gk138);ceh-23(ms23)	93	16.58±0.37	5.417362515	16±0.36	.000	2.33E-01

Figure S2B

Strain	N (post censoring)	mean LS ± SEM (days)	% change to wt	median LS ± SEM (days)	% change to EV control	*P*-value *vs EV control*
wt/EV	66	17.18±0.434		18±0.69		
wt/*aak-2*	73	15.51±0.374	-11.8214105	16±0.45	-11.8	1.57E-04
mev-1(kn1)/EV	57	12.63±0.407	-26.4898731	12±0.74		
mev-1(kn1)/aak-2	58	12.06±0.340	-29.8193654	12±0.62	-4.53	1.31E-01
gas-1(fc21)/EV	54	13.15±0.341	-23.4763864	14±0.42		
gas-1(fc21)/aak-2	59	12.10±0.284	-29.566855	12±0.40	-8	1.80E-02

Figure S3A and S3B

Strains	N (post censoring)	mean LS ± SEM (days)	% change to wt	median LS ± SEM (days)	P-value vs wt/control	% change to isp-1/control	*P*-value *vs isp-1/control*	*% change to wildtype crtc-*	*P*-value *vs wildtype*
wildtype	112	16.00±0.30		16±0.45		-28.54	3.35E-24		
crtc-1 CA	105	16.93±0.32	5.80	18±0.37	3.58E-02	-24.39	6.73E-19	5.80	3.58E-02
isp-1(qm150)	122	22.40±0.43	39.93	22±0.39	3.35E-24				
ceh-23(ms23);isp-1(qm150)	114	20.20±0.37	26.26	20±0.53	8.68E-15	-9.77	3.06E-04		
cep-1(gk138);isp-1(qm150)	112	20.69±0.34	29.31	20±0.39	1.01E-18	-7.59	2.18E-03		
isp-1(qm150);crtc-1 CA	104	19.88±0.73	24.22	20±0.58	1.24E-11	-11.23	1.63E-04	-11.23	1.63E-04
cep-1(gk138);isp-1(qm150);crtc-1CA iso 1	100	20.38±0.47	27.39	20±0.51	1.52E-12	-8.96	3.72E-03	-1.48	9.07E-01
cep-1(gk138);isp-1(qm150);crtc-1CA iso 2	133	20.93±0.40	30.82	20±0.61	3.20E-18	-6.51	1.87E-02	1.17	4.40E-01
ceh-23(ms23);isp-1(qm150);crtc-1CA	105	20.68±0.41	29.25	20±0.43	4.09E-16	-7.64	1.07E-02	2.36	4.63E-01
