# Supplementary material for: Transcription factors CEP‐1/p53 and CEH‐23 collaborate with AAK‐2/AMPK to modulate longevity in Caenorhabditis elegans
Source: Aging Cell. 2017 May 30;16(4):814–24. doi: 10.1111/acel.12619 (PMC5506430; doi:10.1111/acel.12619)
Supplement: Supplementary file 5 — Table S2 Genes that are differentially expressed between isp‐1(qm150) and ceh‐23(ms23); isp‐1(qm150) (identified by Statistical Analysis of Microarray (SAM) 1 class analysis with false discovery rate (FDR) = 0.59%, 1.5 fold change cutoff). [file ACEL-16-814-s005.pdf]

Table S2: Genes differentially expressed between *isp-1(qm150)* and *ceh-23(ms23);isp-1(qm150)* (identified by 1 class SAM with FDR=0.59, 1.5 fold gene cutoff)

A. CEH-23 activating genes

Gene ID	public name	seq name	Score(d)	Numerator(r)	Denominator(s+s0)	q-value(%)
WBGene00013297		Y57G11B.5	4.780825373	2.8325	0.592470919	0.312483699
WBGene00000388	cdc-25.3	ZK637.11	4.143042469	2.7575	0.665573675	0.312483699
WBGene00015899		C17E7.4	4.167841413	2.705	0.649017017	0.312483699
WBGene00008822		F14H3.3	4.299866478	2.685	0.624437994	0.312483699
WBGene00012405	ztf-25	Y6G8.3	4.087441357	2.5975	0.635483123	0.345525782
A_12_P159890			4.450922521	2.595	0.583025202	0.312483699
WBGene00015905		C17E7.12	4.130818491	2.5875	0.626389178	0.345525782
WBGene00022722		ZK370.8	20.32009787	2.5875	0.127336985	0
WBGene00013393		Y62H9A.5	4.937047465	2.525	0.51143928	0.312483699
WBGene00015683		C10G8.4	3.810701538	2.51	0.658671369	0.345525782
WBGene00008825		F14H3.6	4.197952033	2.4775	0.590168725	0.312483699
WBGene00045184		T23F6.7	4.140936743	2.475	0.59769085	0.345525782
WBGene00007315		C04H5.7	5.215052865	2.465	0.472670185	0.312483699
WBGene00016824		C50E3.13	3.999074588	2.45	0.612641737	0.345525782
WBGene00004821	skr-15	F54D10.1	5.54349233	2.44	0.440155746	0.312483699
WBGene00013391		Y62H9A.3	5.145493978	2.42	0.470314417	0.312483699
WBGene00010555		K04C1.5	4.813999488	2.4	0.498545961	0.312483699
WBGene00013931	clec-97	ZK39.6	4.311281375	2.4	0.556679045	0.312483699
WBGene00006927	vit-3	F59D8.1	4.445880598	2.3875	0.537013972	0.312483699
WBGene00008789		F14D7.2	4.319380748	2.3675	0.548110977	0.312483699
WBGene00021263		Y22D7AR.10	3.795872833	2.3275	0.613165957	0.345525782
WBGene00007283	clec-222	C03E10.6	4.932371419	2.325	0.471375694	0.312483699
WBGene00016823		C50E3.12	4.186541579	2.32	0.554156684	0.312483699
WBGene00015180		B0416.4	4.665406699	2.2975	0.492454388	0.312483699
A_12_P175764			4.052164053	2.2925	0.565747085	0.345525782
WBGene00017712		F22E5.17	4.431983426	2.29	0.516698683	0.312483699
WBGene00011741		T12G3.6	3.446623899	2.2875	0.663692955	0.414186921
WBGene00013392		Y62H9A.4	4.680960254	2.2875	0.488681782	0.312483699
WBGene00010492	meg-1	K02B9.1	4.33369685	2.285	0.527263461	0.312483699
WBGene00021730		Y49F6C.8	4.059429906	2.285	0.562886921	0.345525782
WBGene00010085		F55B11.3	5.332204717	2.28	0.427590485	0.312483699
WBGene00018808		F54D10.7	4.43638407	2.2725	0.512241493	0.312483699
WBGene00007459		C08F11.12	4.9179544	2.27	0.461574024	0.312483699
WBGene00045268	ttr-42	H01M10.3	5.055669509	2.2625	0.447517385	0.312483699
WBGene00017263		F08F3.6	3.969832286	2.25	0.566774573	0.345525782
WBGene00013394		Y62H9A.6	5.318587446	2.24	0.421164458	0.312483699
WBGene00008377		D1054.10	4.884638541	2.235	0.457556886	0.312483699
WBGene00012878		Y45F10C.2	4.797754259	2.235	0.465842951	0.312483699
WBGene00019905		R05G9.3	4.216542458	2.2325	0.529462236	0.312483699
WBGene00045261		H29C22.1	5.122282994	2.23	0.435352752	0.312483699
WBGene00017177		F02E8.4	3.904466836	2.215	0.567298966	0.345525782
WBGene00022821		ZK813.2	4.142201732	2.2125	0.534136226	0.312483699
WBGene00011011		R04D3.3	4.201912582	2.205	0.524761036	0.312483699
WBGene00006925	vit-1	K09F5.2	4.092462083	2.2	0.537573704	0.345525782
WBGene00006926	vit-2	C42D8.2	3.882107928	2.18	0.561550591	0.345525782
WBGene00008378		D1054.11	4.594487005	2.18	0.474481699	0.312483699
WBGene00016084	cht-3	C25A8.4	4.054744411	2.165	0.533942409	0.345525782
WBGene00008535		F02H6.2	3.505492194	2.1575	0.615462788	0.414186921
WBGene00013867		ZC373.2	4.342360496	2.155	0.496273859	0.312483699
WBGene00006930	vit-6	K07H8.6	4.145916028	2.15	0.518582621	0.312483699
WBGene00045473		F30A10.14	4.449314881	2.1475	0.482658579	0.312483699
WBGene00022586		ZC308.4	3.960777095	2.1425	0.540929204	0.345525782
WBGene00009606		F40G12.11	3.713378656	2.14	0.576294582	0.345525782
WBGene00008536		F02H6.3	3.601902278	2.1375	0.593436422	0.414186921
WBGene00010084		F55B11.2	4.811535546	2.1375	0.444244874	0.312483699
WBGene00009897		F49E12.1	4.741444923	2.125	0.448175616	0.312483699
WBGene00010613		K07A1.6	5.371709907	2.12	0.39466018	0.312483699
WBGene00012557		Y37D8A.19	4.767699167	2.1175	0.444134566	0.312483699
WBGene00012212		W02D9.6	5.857696038	2.1025	0.358929515	0.300971142
WBGene00006929	vit-5	C04F6.1	4.247653635	2.0975	0.493802033	0.312483699
WBGene00017402		F12E12.1	3.978871604	2.0925	0.525902871	0.345525782
WBGene00010000		F53F8.3	4.2638256	2.08	0.48782483	0.312483699
WBGene00008113		C46C2.5	4.745881324	2.0775	0.437747988	0.312483699
WBGene00019989		R09F10.8	3.910027844	2.075	0.530686758	0.345525782
WBGene00004241	puf-5	F54C9.8	4.78607434	2.0725	0.433027123	0.312483699

WBGene00017843		F26G5.1	5.305625709	2.0675	0.389680711	0.312483699
WBGene00008393		D1086.6	4.688790299	2.065	0.440412104	0.312483699
WBGene00010196		F57C2.4	4.922372281	2.0625	0.419005285	0.312483699
WBGene00011012		R04D3.4	3.496271924	2.0625	0.58991407	0.414186921
WBGene00017541		F17E9.4	4.230804719	2.0625	0.487495911	0.312483699
WBGene00044080		D1086.10	4.713986752	2.0525	0.4354064	0.312483699
WBGene00045239		Y49E10.29	5.18589675	2.0525	0.395784972	0.312483699
WBGene00044535		K11D12.13	4.620537456	2.0375	0.440966017	0.312483699
WBGene00006928	vit-4	F59D8.2	3.969120129	2.0275	0.510818502	0.345525782
WBGene00045038		F13G11.3	4.222793005	2.0175	0.477764361	0.312483699
WBGene00011000		R03G8.6	6.568061184	2.015	0.306787642	0.300971142
WBGene00012213		W02D9.7	5.311219253	2.0025	0.377032072	0.312483699
WBGene00004078	pos-1	F52E1.1	4.235714641	1.9925	0.470404682	0.312483699
WBGene00045061		Y57G11B.8	4.649550659	1.9875	0.427460661	0.312483699
WBGene00006619	try-1	ZK546.15	3.574259185	1.985	0.555359837	0.414186921
WBGene00044773		C08A9.10	4.321767136	1.9825	0.458724392	0.312483699
WBGene00045394		ZK813.7	5.312990982	1.98	0.37267144	0.312483699
WBGene00021988	rgs-8.2	Y59E1A.2	5.408414494	1.9775	0.365633958	0.312483699
WBGene00004352	rgs-9	ZC53.7	4.566643327	1.975	0.432483962	0.312483699
WBGene00006541	tbh-1	H13N06.6	3.368533858	1.95	0.578886864	0.463367002
WBGene00045265		K10C2.8	4.467293757	1.945	0.435386636	0.312483699
WBGene00021136		W10G11.3	3.972652452	1.94	0.488338717	0.345525782
WBGene00016303	fbxc-32	C32B5.10	3.877108882	1.9375	0.499728034	0.345525782
WBGene00008912		F17C8.7	3.24342461	1.935	0.596591638	0.463367002
WBGene00019153		H04M03.3	4.257042639	1.935	0.454540902	0.312483699
WBGene00019622		K10C2.7	3.819295836	1.935	0.506637894	0.345525782
WBGene00050914		T12B5.15	4.254392914	1.93	0.453648744	0.312483699
WBGene00044081		D1086.11	4.699786629	1.925	0.409593063	0.312483699
WBGene00012672		Y39B6A.10	4.082612158	1.9225	0.470899494	0.345525782
WBGene00012880		Y45F10C.4	3.880352482	1.915	0.493511868	0.345525782
WBGene00016822		C50E3.11	4.833197635	1.915	0.396218021	0.312483699
WBGene00021005		W03F11.1	3.387891142	1.9125	0.564510464	0.463367002
WBGene00020218		T04G9.7	3.938086777	1.91	0.485007088	0.345525782
WBGene00003539	nas-20	T11F9.3	4.102246583	1.905	0.464379691	0.345525782
WBGene00004374	rme-2	T11F8.3	4.01551223	1.905	0.47441021	0.345525782
WBGene00011431		T04D3.1	4.610170642	1.905	0.413216809	0.312483699
WBGene00017105		E02H9.7	4.379058153	1.905	0.435025052	0.312483699
WBGene00017134		EEED8.3	3.954354546	1.9	0.480482966	0.345525782
WBGene00007225		C01G6.3	3.959387942	1.8975	0.479240738	0.345525782
WBGene00020290		T06D4.1	3.707149797	1.8975	0.511848753	0.345525782
WBGene00003987	pes-23	F14B8.3	4.745992892	1.8925	0.398757445	0.312483699
WBGene00010065		F54F7.3	5.625611924	1.8925	0.336407848	0.312483699
WBGene00015912	fbxc-50	C17F4.5	4.166767355	1.8875	0.452989053	0.312483699
WBGene00001569	gei-12	F52D2.4	4.893150625	1.88	0.384210531	0.312483699
WBGene00000597	col-8	F11H8.3	3.272494428	1.875	0.572957431	0.463367002
WBGene00015102	cpg-2	B0280.5	4.069166513	1.875	0.460782323	0.345525782
WBGene00009659		F43G6.7	3.764778445	1.865	0.495381077	0.345525782
WBGene00018446	ceh-83	F45C12.15	4.145277849	1.8625	0.449306432	0.312483699
WBGene00007458		C08F11.11	4.245634022	1.855	0.436919431	0.312483699
WBGene00007196		B0513.4	3.871086179	1.845	0.476610417	0.345525782
WBGene00021153		Y4C6A.3	4.216183592	1.8425	0.437006587	0.312483699
WBGene00016638	perm-4	C44B12.5	4.957166583	1.835	0.370171139	0.312483699
WBGene00011010		R04D3.2	5.641406949	1.8325	0.324830316	0.312483699
A_12_P111211			4.831563113	1.8175	0.376172257	0.312483699
WBGene00016896	nep-4	C53B7.7	4.374600877	1.815	0.414894993	0.312483699
WBGene00007332		C05C10.5	4.12237995	1.8125	0.439673204	0.345525782
WBGene00006394	taf-11.2	K10D3.3	4.283706327	1.8075	0.421947693	0.312483699
A_12_P100632			4.378366663	1.805	0.41225419	0.312483699
WBGene00011501	rmd-1	T05G5.7	4.043628673	1.805	0.446381245	0.345525782
WBGene00045271		F07G6.10	5.929345444	1.805	0.304418087	0.300971142
WBGene00000757	col-184	F15A2.1	4.521426519	1.7975	0.397551523	0.312483699
WBGene00021898		Y54G2A.36	3.771926593	1.7975	0.47654692	0.345525782
WBGene00022820		ZK813.1	5.627627292	1.795	0.318962132	0.312483699
WBGene00016485		C36C9.1	4.345124973	1.785	0.410805215	0.312483699
WBGene00016534		C39D10.7	3.754815461	1.785	0.475389541	0.345525782
WBGene00021853		Y54F10AM.11	3.718798475	1.7825	0.479321483	0.345525782
WBGene00008394		D1086.7	3.78131575	1.7775	0.470074471	0.345525782
WBGene00000608	col-19	ZK1193.1	3.809732713	1.77	0.464599523	0.345525782
WBGene00003784	nos-2	ZK1127.1	4.384156693	1.77	0.403726446	0.312483699
WBGene00018605		F48E3.4	3.527350029	1.77	0.501793127	0.414186921

WBGene00010183	fipr-13	F57A8.8	4.437604215	1.7625	0.397173771	0.312483699
WBGene00014516	hpo-42	T10C6.8	4.072975171	1.75	0.429661347	0.345525782
WBGene00016627		C44B7.5	3.790335546	1.75	0.461700548	0.345525782
WBGene00010007		F53H4.2	4.35421867	1.745	0.400760764	0.312483699
WBGene00008844		F15A4.10	4.163601925	1.74	0.417907387	0.312483699
WBGene00000716	col-143	T15B7.3	3.602585063	1.7225	0.478128891	0.414186921
WBGene00011320		T01C3.3	4.509171693	1.72	0.381444779	0.312483699
WBGene00007313	clec-147	C04H5.2	3.936171337	1.7125	0.435067443	0.345525782
WBGene00007709	clec-87	C25A1.8	4.731152842	1.7125	0.361962519	0.312483699
WBGene00004984	spn-4	ZC404.8	4.484380196	1.7075	0.380766109	0.312483699
WBGene00016178	mesp-1	C28C12.2	3.726596131	1.7075	0.45819293	0.345525782
WBGene00011342		T01G5.7	5.928478893	1.695	0.285908077	0.300971142
WBGene00050913		T12B5.14	4.155616543	1.69	0.406678524	0.312483699
WBGene00001607	gln-6	C28D4.3	3.834276787	1.6775	0.437501019	0.345525782
WBGene00010066		F54F7.6	7.860905865	1.655	0.210535532	0
WBGene00010234	ttr-50	F58B3.9	4.208659577	1.6475	0.391454802	0.312483699
WBGene00001647	gna-2	T23G11.2	3.717878123	1.645	0.442456677	0.345525782
WBGene00016434		C35B1.4	3.723852799	1.645	0.441746784	0.345525782
WBGene00006047	ssp-19	C55C2.2	3.962060602	1.6425	0.414557011	0.345525782
WBGene00050938		F23H12.11	4.912787512	1.6425	0.334331578	0.312483699
WBGene00014097		ZK829.9	2.682549939	1.64	0.611358609	0.593984595
WBGene00015083	egg-1	B0244.8	4.429234317	1.64	0.370267157	0.312483699
WBGene00020167		T02G5.11	5.318014704	1.635	0.307445558	0.312483699
A_12_P159150			5.097186792	1.6325	0.320274706	0.312483699
A_12_P181533			4.38569519	1.63	0.371662856	0.312483699
WBGene00020433		T11F8.1	4.063853704	1.63	0.401097116	0.345525782
WBGene00007649		C17G1.2	3.881135385	1.6275	0.419336055	0.345525782
WBGene00011489		T05F1.2	3.920448389	1.6275	0.415131087	0.345525782
WBGene00020797		T25D3.3	4.483074896	1.62	0.36135912	0.312483699
WBGene00014117	clec-91	ZK858.3	4.166305209	1.6175	0.388233679	0.312483699
WBGene00006555	tbx-36	ZK829.5	3.667659991	1.61	0.438971989	0.345525782
WBGene00003465	msp-78	T13F2.11	5.264217568	1.6025	0.30441371	0.312483699
WBGene00012231		W04A4.2	4.951082689	1.6025	0.32366658	0.312483699
WBGene00016636	perm-2	C44B12.1	5.760814967	1.5975	0.277304515	0.312483699
WBGene00000465	cpg-1	C07G2.1	4.098370615	1.5825	0.386129062	0.345525782
WBGene00003865	oma-2	ZC513.6	3.900130166	1.5825	0.405755688	0.345525782
WBGene00009186	trcs-1	F27C8.6	4.549169167	1.5775	0.346766616	0.312483699
WBGene00017548		F18A1.7	4.649298454	1.5725	0.338223071	0.312483699
WBGene00021596	spsb-2	Y46E12BL.3	4.237099263	1.5725	0.371126543	0.312483699
WBGene00016658		C45B2.1	4.074687619	1.5675	0.384692066	0.345525782
WBGene00011461			3.956919643	1.565	0.395509674	0.345525782
WBGene00007521		C11E4.7	4.798507142	1.555	0.32405912	0.312483699
WBGene00077443		Y71A12B.19	3.440859003	1.5525	0.451195471	0.414186921
WBGene00008207		C49F5.3	5.176932988	1.5475	0.298922162	0.312483699
WBGene00000754	col-181	W03G11.1	3.603658875	1.545	0.428730924	0.414186921
WBGene00000752	col-179	C34F6.3	3.894829098	1.5425	0.396037916	0.345525782
WBGene00000680	col-106	Y77E11A.15	3.202158926	1.54	0.480925537	0.489403914
WBGene00000698	col-124	C24F3.6	2.757328946	1.54	0.558511527	0.495348337
WBGene00017648	ddo-3	F20H11.5	3.665891104	1.5375	0.419406894	0.345525782
WBGene00011350	perm-1	T01H3.4	4.047413722	1.535	0.379254533	0.345525782
WBGene00020350		T08B2.12	5.847168757	1.5325	0.262092658	0.300971142
WBGene00044474		F56D6.12	4.815929118	1.5175	0.315100153	0.312483699
WBGene00017747		F23F1.6	3.754434749	1.51	0.402191036	0.345525782
WBGene00022875		ZK1248.4	3.019187667	1.505	0.498478454	0.495348337
WBGene00000866	cyb-2.1	Y43E12A.1	3.87190035	1.5025	0.388052342	0.345525782
WBGene00001606	gln-5	F26D10.10	4.185394212	1.5025	0.358986495	0.312483699
WBGene00012747		Y40H7A.10	3.358232116	1.5025	0.447408025	0.463367002
WBGene00020786		T25B2.2	4.924455587	1.4925	0.303079188	0.312483699
WBGene00000867	cyb-2.2	H31G24.4	4.858045404	1.4875	0.306193104	0.312483699
WBGene00010353		H02I12.5	3.806166841	1.4875	0.390813136	0.345525782
A_12_P167224			3.852613212	1.475	0.382857016	0.345525782
WBGene00003184	mei-2	F57B10.12	4.63542137	1.475	0.318201924	0.312483699
WBGene00011929		T22G5.1	5.375279924	1.475	0.274404314	0.312483699
WBGene00003425	msp-10	K07F5.2	3.19537733	1.4725	0.460821946	0.489403914
WBGene00003464	msp-77	F32B6.6	3.477919244	1.4675	0.421947693	0.414186921
WBGene00003466	msp-79	T13F2.10	3.20279931	1.4675	0.45819293	0.489403914
WBGene00003463	msp-76	ZK1251.6	3.042333161	1.465	0.48153832	0.495348337
WBGene00022002		Y59E9AR.7	3.904246388	1.465	0.375232466	0.345525782
WBGene00008590		F08H9.2	4.352197315	1.4625	0.336037154	0.312483699
WBGene00003228	mex-1	W03C9.7	4.800732888	1.46	0.304120232	0.312483699

WBGene00003443	msp-50	C34F11.4	3.749212543	1.455	0.388081493	0.345525782
WBGene00011986		T24D1.3	3.415999775	1.455	0.425936796	0.463367002
WBGene00004239	puf-3	Y45F10A.2	4.418870391	1.4525	0.328703916	0.312483699
WBGene00003452	msp-59	ZK354.11	3.146894268	1.45	0.460771757	0.489403914
WBGene00022257	puf-11	Y73B6BL.38	4.411851258	1.45	0.328660219	0.312483699
WBGene00000473	cey-2	F46F11.2	4.793847755	1.4475	0.301949514	0.312483699
WBGene00007097		B0024.4	5.768241325	1.445	0.25050963	0.312483699
WBGene00014116		ZK858.2	3.41452426	1.44	0.421727857	0.463367002
WBGene00017986		F32D1.7	3.930104767	1.44	0.366402446	0.345525782
WBGene00021763		Y51F10.2	5.186476753	1.44	0.277645128	0.312483699
WBGene00003426	msp-19	F36H12.7	3.555931133	1.4375	0.404254173	0.414186921
WBGene00016447	msd-4	C35D10.11	2.981172269	1.43	0.479677077	0.495348337
WBGene00017718		F22F4.5	2.905828621	1.43	0.492114363	0.495348337
WBGene00007278	lab-1	C03D6.6	2.939111872	1.4275	0.485690937	0.495348337
WBGene00016893		C53B7.2	4.936964672	1.4225	0.288132505	0.312483699
WBGene00018084		F36A4.5	2.910892223	1.4225	0.488681782	0.495348337
WBGene00021997		Y59E9AR.1	3.690548839	1.4175	0.384089213	0.345525782
WBGene00000657	col-81	F38A3.1	3.48329056	1.415	0.406225084	0.414186921
WBGene00000989	dhs-26	ZK816.5	2.891480254	1.4125	0.488504114	0.495348337
WBGene00020948		W02F12.3	3.79843008	1.4125	0.371864157	0.345525782
WBGene00021207		Y18H1A.1	4.166431159	1.4125	0.339019162	0.312483699
WBGene00045105		Y7A9C.11	3.740780124	1.4125	0.377595034	0.345525782
WBGene00008218	nasp-2	C50B6.2	3.87926502	1.41	0.363470913	0.345525782
WBGene00000301	cav-1	T13F2.8	4.596250622	1.4075	0.306227862	0.312483699
WBGene00014675		C12D8.3	3.162181116	1.4075	0.44510417	0.489403914
WBGene00009701	egg-3	F44F4.2	4.280939662	1.405	0.328198973	0.312483699
WBGene00012789		Y43D4A.3	6.083541686	1.4025	0.230540049	0.300971142
WBGene00000713	col-140	F26F12.1	2.976767148	1.395	0.468629198	0.495348337
WBGene00008464		E02H4.6	4.249645937	1.395	0.328262641	0.312483699
A_12_P154291			5.357664125	1.3925	0.259908043	0.312483699
WBGene00012224		W03G11.2	3.618546988	1.3925	0.38482297	0.414186921
WBGene00003457	msp-64	ZK1248.6	3.172208255	1.385	0.436604374	0.489403914
WBGene00009005		F21C3.6	3.968917	1.385	0.348961694	0.345525782
WBGene00009626		F42A8.1	3.258225188	1.385	0.425078047	0.463367002
WBGene00016243			3.662726749	1.385	0.378133586	0.345525782
WBGene00003231	mex-6	AH6.5	4.539428578	1.3825	0.304553751	0.312483699
WBGene00003444	msp-51	ZK354.5	3.371697756	1.38	0.409289355	0.463367002
WBGene00011063	cpg-3	R06C7.4	3.344225538	1.38	0.412651594	0.463367002
WBGene00011898		T21C9.13	4.264660896	1.38	0.32358962	0.312483699
A_12_P172558			3.993018015	1.3775	0.344977156	0.345525782
WBGene00003438	msp-45	F58A6.8	2.943215554	1.375	0.467176112	0.495348337
WBGene00003462	msp-74	F09C12.7	3.410890382	1.375	0.403120548	0.463367002
WBGene00007757		C27A7.6	4.106147109	1.375	0.334863794	0.345525782
WBGene00000496	chs-1	T25G3.2	4.490631695	1.3725	0.305636288	0.312483699
WBGene00003434	msp-38	K08F4.8	3.888103211	1.3725	0.352999889	0.345525782
WBGene00011173	acs-18	R09E10.3	3.266954432	1.37	0.419350814	0.463367002
WBGene00011434		T04D3.5	2.981765877	1.37	0.459459279	0.495348337
WBGene00012879	fbxa-215	Y45F10C.3	5.216772648	1.3675	0.26213525	0.312483699
WBGene00007301		C04F12.7	4.199224531	1.365	0.325060018	0.312483699
WBGene00010633	nspd-2	K07F5.5	3.802052155	1.3625	0.358359103	0.345525782
WBGene00016752		C48E7.7	4.358714653	1.36	0.31201859	0.312483699
WBGene00003448	msp-55	C09B9.6	3.010698027	1.3575	0.450892115	0.495348337
WBGene00007751		C26G2.2	3.648118402	1.3575	0.372109633	0.345525782
WBGene00009130		F25H5.8	3.490136931	1.355	0.388236916	0.414186921
WBGene00013349		Y59A8B.12	3.080061747	1.3525	0.439114573	0.489403914
WBGene00000598	col-9	F54B11.1	3.668380851	1.3475	0.367328272	0.345525782
WBGene00003541	nas-22	T11F9.6	3.840785999	1.3475	0.350839646	0.345525782
WBGene00003395	mom-2	F38E1.7	5.194652226	1.345	0.258920124	0.312483699
WBGene00003469	msp-142	K05F1.2	3.339593016	1.34	0.401246497	0.463367002
WBGene00009035		F22B3.4	4.829738421	1.34	0.277447738	0.312483699
WBGene00017985		F32D1.6	4.387486147	1.34	0.30541407	0.312483699
WBGene00003456	msp-63	K05F1.7	3.065173082	1.3375	0.436353825	0.495348337
WBGene00020684		T22D1.5	5.259262453	1.3375	0.254313226	0.312483699
WBGene00013567		Y75B12B.1	4.405237354	1.335	0.30304837	0.312483699
WBGene00017258		F08F1.4	6.878010671	1.335	0.19409682	0.300971142
WBGene00008080		C44B9.3	3.944718419	1.3325	0.337793439	0.345525782
WBGene00022653		ZK105.1	4.077635918	1.33	0.326169385	0.345525782
WBGene00011690		T10C6.9	6.800931574	1.3275	0.195193847	0.300971142
WBGene00017978		F32B5.4	3.718040548	1.3275	0.357042905	0.345525782
WBGene00004908	snf-9	C49C3.1	3.535215491	1.325	0.374800349	0.414186921

WBGene00009695		F44F1.3	3.389893583	1.325	0.390867727	0.463367002
WBGene00019920	acs-15	R07C3.4	3.417884824	1.325	0.387666662	0.463367002
WBGene00022652		ZK84.5	3.233384775	1.325	0.409787295	0.489403914
WBGene00007643	41336	C17E4.3	3.935938809	1.3225	0.33600624	0.345525782
WBGene00017851		F27C1.1	3.317178418	1.3225	0.398682203	0.463367002
WBGene00004966	spe-12	T02E1.1	3.635302626	1.32	0.363105946	0.414186921
WBGene00009682	msd-2	F44D12.5	3.518705191	1.32	0.375137992	0.414186921
WBGene00019811	egg-2	R01H2.3	3.917120969	1.32	0.336982189	0.345525782
WBGene00019341		K02F6.7	3.046696581	1.3175	0.432435579	0.495348337
WBGene00021781		Y51H7C.3	3.845336454	1.315	0.341972677	0.345525782
WBGene00007794		C28D4.7	3.342388458	1.3125	0.392683261	0.463367002
WBGene00011336	ubxn-5	T01E8.9	3.764700225	1.3125	0.348633336	0.345525782
WBGene00013383		Y62E10A.17	4.139033584	1.3125	0.317103008	0.345525782
WBGene00021056		W06B4.1	3.532107569	1.3125	0.371591174	0.414186921
WBGene00022005		Y59H11AM.1	3.524347207	1.3125	0.372409392	0.414186921
WBGene00008801	acp-3	F14E5.3	3.368728246	1.31	0.388870786	0.463367002
WBGene00006050	ssq-1	K07F5.11	2.912016496	1.3075	0.449001577	0.495348337
WBGene00006977	zif-1	F59B2.6	4.077420971	1.3075	0.320668386	0.345525782
WBGene00012716		Y39E4B.5	5.639294202	1.3075	0.231855256	0.312483699
WBGene00002090	ins-7	ZK1251.2	2.906995167	1.305	0.448917155	0.495348337
WBGene00014194		ZK1037.6	4.319207722	1.3025	0.301559935	0.312483699
WBGene00011711			4.013982635	1.2975	0.323245046	0.345525782
WBGene00020910	dgtr-1	W01A11.2	4.406217597	1.2975	0.294470251	0.312483699
WBGene00017584		F19B10.2	3.867961338	1.295	0.334801692	0.345525782
WBGene00004243	puf-7	B0273.2	5.040350245	1.2925	0.256430593	0.312483699
WBGene00003424	msp-3	F26G1.7	3.287076056	1.2875	0.391685491	0.463367002
WBGene00017059		D2062.7	4.30920908	1.2875	0.298778726	0.312483699
WBGene00022237		Y73B6BL.14	4.271475264	1.2875	0.30141811	0.312483699
WBGene00003431	msp-33	R05F9.8	3.137074167	1.285	0.409617348	0.489403914
WBGene00044475		F56D6.13	3.577216316	1.285	0.359217863	0.414186921
A_12_P107060			3.717289911	1.28	0.344336877	0.345525782
WBGene00012790		Y43D4A.4	5.264448117	1.28	0.243140396	0.312483699
WBGene00016558		C41A3.1	3.004895395	1.28	0.425971567	0.495348337
WBGene00020860	nspd-10	T27C10.7	4.736979276	1.28	0.270214397	0.312483699
WBGene00003429	msp-31	R05F9.13	3.864143758	1.2775	0.330603642	0.345525782
WBGene00006056	sss-1	F32B6.5	3.371560461	1.2775	0.378904669	0.463367002
WBGene00018128		F36H12.15	3.294723038	1.2775	0.387741241	0.463367002
WBGene00014674		C12D8.2	3.573547891	1.275	0.356788278	0.414186921
WBGene00022760		ZK546.3	4.263726938	1.275	0.299034159	0.312483699
WBGene00019606	clec-88	K10B2.3	4.025579097	1.2725	0.316103589	0.345525782
WBGene00022537		ZC190.2	5.387793075	1.2725	0.236182048	0.312483699
WBGene00000638	col-62	C15A11.6	4.023784305	1.27	0.31562328	0.345525782
WBGene00021206		Y17G9B.9	3.772540986	1.27	0.336643129	0.345525782
WBGene00003435	msp-40	C33F10.9	3.541249925	1.265	0.357218504	0.414186921
WBGene00003449	msp-56	K07F5.3	3.240224738	1.265	0.390405019	0.489403914
WBGene00022706		ZK354.3	3.611205663	1.2625	0.349606231	0.414186921
WBGene00003783	nos-1	R03D7.7	4.415525235	1.26	0.285356766	0.312483699
WBGene00009449		F35H8.4	3.705388804	1.26	0.340045287	0.345525782
WBGene00015698	kca-1	C10H11.10	3.825631337	1.2575	0.328703916	0.345525782
WBGene00004242	puf-6	F18A11.1	3.937897667	1.255	0.318697972	0.345525782
WBGene00016399		C34D4.3	3.410621782	1.255	0.367968095	0.463367002
A_12_P136645			3.697818155	1.2525	0.338713249	0.345525782
WBGene00011172		R09E10.2	4.24708143	1.2525	0.294908403	0.312483699
WBGene00015907		C17F3.1	4.851319125	1.2525	0.258177202	0.312483699
WBGene00019189		H11L12.1	4.167416097	1.25	0.299946051	0.312483699
WBGene00022884		ZK1248.17	2.713586775	1.25	0.460644934	0.593984595
WBGene00003442	msp-49	C34F11.6	3.092353983	1.2475	0.403414359	0.489403914
WBGene00012683	asp-17	Y39B6A.24	4.656845436	1.2475	0.267885206	0.312483699
WBGene00021114		W09C3.7	3.315367263	1.2425	0.374769943	0.463367002
WBGene00004026	phy-3	T20B3.7	2.926004733	1.24	0.423786054	0.495348337
WBGene00015987		C18G1.9	3.532424235	1.24	0.351033714	0.414186921
WBGene00021035		W05F2.3	4.537149784	1.24	0.273299331	0.312483699
WBGene00009681	gipc-2	F44D12.4	3.658827877	1.2375	0.338223071	0.345525782
WBGene00012294	nspa-8	W06A7.5	3.158183307	1.2375	0.391839194	0.489403914
WBGene00016742		C48B6.4	4.330111961	1.2375	0.285789377	0.312483699
WBGene00021658		Y48G1A.2	5.096207187	1.2375	0.242827647	0.312483699
WBGene00013474		Y69E1A.2	3.770423691	1.2325	0.32688634	0.345525782
WBGene00016568		C41G11.1	3.506863889	1.23	0.35074073	0.414186921
WBGene00044062	snb-6	T14D7.3	5.758079104	1.2225	0.212310386	0.312483699
WBGene00011462	scl-22	T05A10.5	2.857364311	1.22	0.426966906	0.495348337

WBGene00009324		F32B6.10	3.096236943	1.2175	0.39321926	0.489403914
WBGene00019950	clec-175	R08C7.6	2.872062527	1.2175	0.423911384	0.495348337
WBGene00043743		Y59E9AL.2	3.390416509	1.2175	0.359100422	0.463367002
WBGene00000474	cey-3	M01E11.5	3.758711136	1.215	0.323249102	0.345525782
WBGene00012020	gyg-2	T25E12.5	3.814493983	1.2125	0.317866539	0.345525782
WBGene00020035	egg-5	R12E2.10	3.252022656	1.2125	0.372844881	0.463367002
WBGene00015937		C17H12.12	3.715583036	1.21	0.325655486	0.345525782
WBGene00044554		F41E6.15	3.735191925	1.21	0.323945871	0.345525782
WBGene00008433	41335	D2089.2	4.414621317	1.205	0.272956594	0.312483699
WBGene00011748		T13F2.9	3.284130512	1.205	0.366915991	0.463367002
WBGene00017944		F31D5.1	3.021293445	1.205	0.398835804	0.495348337
WBGene00018081		F36A4.2	3.714381377	1.205	0.324414722	0.345525782
WBGene00003433	msp-37	K08F4.10	3.517023904	1.2025	0.341908396	0.414186921
WBGene00007320		C05B5.2	3.899061436	1.2025	0.308407554	0.345525782
WBGene00019501		K07E12.2	2.793792564	1.2	0.429523657	0.495348337
WBGene00003470	msp-152	ZK546.6	3.341886393	1.1975	0.358330553	0.463367002
WBGene00016501		C37C3.9	4.188019576	1.195	0.28533773	0.312483699
WBGene00022754	nspd-1	ZK484.8	3.68502482	1.195	0.324285468	0.345525782
WBGene00016351		C33F10.1	3.953508522	1.1925	0.301630816	0.345525782
WBGene00008754		F13E9.5	3.181339879	1.19	0.37405623	0.489403914
WBGene00011176		R09E10.6	3.874747015	1.19	0.307116825	0.345525782
WBGene00013858	ssp-34	ZC168.6	3.342624785	1.185	0.354511821	0.463367002
WBGene00017902		F28E10.4	3.206648283	1.1825	0.368765108	0.489403914
WBGene00013380		Y62E10A.14	3.435266518	1.18	0.34349591	0.414186921
WBGene00000656	col-80	C09G5.5	2.724671204	1.1775	0.432162236	0.593984595
WBGene00006052	ssq-3	ZC477.1	3.51770076	1.1775	0.334735693	0.414186921
WBGene00018840		F54H5.3	3.012552819	1.1775	0.390864516	0.495348337
WBGene00007795		C28D4.8	4.073669479	1.175	0.288437736	0.345525782
WBGene00010493	meg-2	K02B9.2	3.254541365	1.175	0.361033973	0.463367002
WBGene00013887	nspa-5	ZC412.6	3.235594685	1.175	0.363148081	0.489403914
WBGene00001683	gpd-1	T09F3.3	5.423198123	1.1725	0.216200842	0.312483699
WBGene00010381		H12D21.5	3.040229806	1.17	0.38483933	0.495348337
WBGene00010091	ssp-35	F55C5.1	3.398764374	1.1675	0.34350719	0.463367002
WBGene00000868	cyb-3	T06E6.2	4.765224914	1.165	0.244479541	0.312483699
WBGene00015312		C01G8.1	4.018151294	1.165	0.289934329	0.345525782
WBGene00016058	nspd-3	C24D10.7	3.744154465	1.165	0.311151693	0.345525782
WBGene00014239		ZK1225.5	2.91990121	1.1625	0.398129908	0.495348337
WBGene00014987		ZK637.6	3.683432346	1.1625	0.315602376	0.345525782
A_12_P178289			3.572647656	1.16	0.324689169	0.414186921
A_12_P159213			4.336337397	1.155	0.266353813	0.312483699
WBGene00013888	nspa-3	ZC412.7	2.79514283	1.155	0.413216809	0.495348337
WBGene00016288		C31H1.5	3.786727786	1.155	0.305012683	0.345525782
WBGene00020652	egg-4	T21E3.1	3.855053419	1.155	0.299606743	0.345525782
A_12_P159211			4.512194015	1.1525	0.255418982	0.312483699
WBGene00009377		F34D10.8	3.816416007	1.1525	0.301984898	0.345525782
WBGene00010563		K04G2.4	3.442056901	1.1525	0.334828863	0.414186921
WBGene00013696		Y106G6A.4	3.663027019	1.1525	0.314630494	0.345525782
WBGene00009457		F36A2.10	3.38190248	1.15	0.340045287	0.463367002
WBGene00010650		K08C9.1	3.172195655	1.1475	0.36173683	0.489403914
WBGene00020713		T23B3.5	4.268425174	1.1475	0.268834512	0.312483699
WBGene00008440		DY3.8	4.507732038	1.145	0.254008	0.312483699
WBGene00010351	cbd-1	H02I12.1	4.091574787	1.1425	0.27923234	0.345525782
WBGene00012211	ssp-37	W02D9.5	3.592928106	1.14	0.317289956	0.414186921
A_12_P166055			3.475722984	1.1375	0.32727004	0.414186921
WBGene00004813	skr-7	Y47D7A.1	3.498356885	1.1375	0.325152647	0.414186921
WBGene00007601		C15C6.2	2.986566533	1.1375	0.380872145	0.495348337
WBGene00017058		D2062.6	3.692014601	1.1375	0.308097373	0.345525782
WBGene00015780		C14F11.4	3.93923311	1.1325	0.287492506	0.345525782
WBGene00019336			2.718215992	1.1325	0.416633558	0.593984595
WBGene00021996		Y59E9AL.6	3.938200382	1.1325	0.287567897	0.345525782
A_12_P159214			4.369765528	1.13	0.258595111	0.312483699
WBGene00000654	col-78	W07A12.5	3.733772596	1.13	0.302642963	0.345525782
WBGene00003230	mex-5	W02A2.7	3.00059173	1.13	0.376592386	0.495348337
WBGene00010933		M162.7	3.300710841	1.13	0.342350498	0.463367002
WBGene00013827		Y116F11B.11	3.713068258	1.13	0.304330522	0.345525782
WBGene00022780		ZK622.1	2.859179312	1.13	0.395218304	0.495348337
WBGene00007793		C28D4.5	3.387254694	1.1275	0.332865433	0.463367002
WBGene00011460	ttr-14	T05A10.3	3.334203367	1.1275	0.338161736	0.463367002
WBGene00001413	fem-3	C01F6.4	4.097994858	1.125	0.274524503	0.345525782
WBGene00012781	nspd-7	Y43C5A.1	3.459527851	1.125	0.325188884	0.414186921

WBGene00019026		F58A6.9	3.488354539	1.125	0.322501623	0.414186921
A_12_P173482			5.285648432	1.1225	0.212367511	0.312483699
WBGene00006040	ssp-11	T28H11.6	3.6056541	1.12	0.310623251	0.414186921
WBGene00007159		B0379.7	3.611378448	1.1175	0.30943863	0.414186921
WBGene00011551		T06G6.12	6.254851383	1.1175	0.178661319	0.300971142
WBGene00012185		W01F3.2	3.806310259	1.1175	0.293591411	0.345525782
WBGene00013208		Y54G9A.5	2.800694825	1.1175	0.399008128	0.495348337
WBGene00017386	nspd-5	F11G11.8	2.927873645	1.115	0.380822445	0.495348337
WBGene00006048	ssp-31	ZK1225.6	3.348808265	1.1125	0.332207732	0.463367002
WBGene00019430		K06A5.2	3.067317028	1.1125	0.362694821	0.495348337
WBGene00006051	ssq-2	T28H11.5	3.438548492	1.11	0.322810629	0.414186921
WBGene00009651		F43D2.2	5.852002044	1.11	0.189678676	0.300971142
WBGene00015244		B0524.5	2.712662264	1.11	0.409192112	0.593984595
WBGene00021795		Y52E8A.2	3.631508984	1.11	0.305658063	0.414186921
WBGene00009959		F53B6.4	3.213704381	1.1075	0.344617883	0.489403914
WBGene00013988		ZK512.10	3.185881992	1.1075	0.34762744	0.489403914
WBGene00010611		K07A1.4	3.892405379	1.105	0.283886156	0.345525782
WBGene00013862	wdr-5.3	ZC302.2	5.922959875	1.105	0.186562128	0.300971142
WBGene00016612		C43G2.3	2.826486127	1.105	0.390944781	0.495348337
WBGene00010136		F55H12.5	4.310652403	1.1	0.255181791	0.312483699
WBGene00016421	cdc-7	C34G6.5	3.041448345	1.1	0.361669795	0.495348337
WBGene00000712	col-139	F41F3.4	4.236015203	1.0975	0.259087833	0.312483699
A_12_P159210			4.979741197	1.095	0.219890945	0.312483699
A_12_P159212			3.483574516	1.095	0.314332303	0.414186921
WBGene00009085		F23D12.1	3.245132077	1.095	0.337428485	0.463367002
WBGene00014158		ZK938.1	3.349419358	1.095	0.326922336	0.463367002
WBGene00013303		Y57G11C.5	3.266194929	1.0925	0.334487079	0.463367002
WBGene00015850		C16C8.12	3.197848194	1.0925	0.34163598	0.489403914
WBGene00012937		Y47D3A.31	2.708817878	1.09	0.402389547	0.593984595
WBGene00018013	phf-10	F33E11.6	3.832590291	1.09	0.284402954	0.345525782
WBGene00010486		K01H12.4	3.412012213	1.0875	0.318726878	0.463367002
WBGene00013653		Y105C5B.14	3.290065437	1.0875	0.330540538	0.463367002
WBGene00016036			2.803767931	1.0875	0.387870903	0.495348337
WBGene00007378		C06C3.8	2.916849429	1.085	0.371976691	0.495348337
WBGene00009269		F30A10.12	3.18003858	1.08	0.339618521	0.489403914
WBGene00010137	ztf-26	F55H12.6	3.029565691	1.08	0.356486741	0.495348337
WBGene00012787		Y43D4A.1	4.257425064	1.08	0.253674459	0.312483699
WBGene00013087		Y51B9A.5	3.540228253	1.08	0.305065076	0.414186921
WBGene00016440	gipc-1	C35D10.2	3.386858112	1.08	0.318879612	0.463367002
WBGene00019260		H34I24.1	3.601076033	1.08	0.299910357	0.414186921
WBGene00016360		C33G8.2	4.28510566	1.0775	0.251452376	0.312483699
WBGene00045355		D1086.17	3.953437991	1.0775	0.272547591	0.345525782
WBGene00017452	bath-29	F14D2.4	3.038794847	1.075	0.353758662	0.495348337
WBGene00021869		Y54G2A.3	3.726974204	1.075	0.288437736	0.345525782
WBGene00022162		Y71G12B.27	3.628208528	1.0725	0.29560043	0.414186921
WBGene00011134	ssp-33	R08A2.3	4.402458076	1.07	0.24304604	0.312483699
WBGene00022006	mpst-5	Y59H11AM.2	3.370898248	1.07	0.317422812	0.463367002
WBGene00007573		C14B1.2	3.539094631	1.0675	0.301630816	0.414186921
WBGene00015908		C17F3.3	3.269183656	1.0675	0.326534117	0.463367002
WBGene00009656		F43G6.3	3.338608933	1.065	0.318995133	0.463367002
WBGene00018087		F36D4.1	2.826076509	1.065	0.376847547	0.495348337
WBGene00022632		ZC581.2	3.240585373	1.065	0.328644327	0.489403914
WBGene00000682	col-108	Y41D4A.2	2.866922905	1.0625	0.370606408	0.495348337
WBGene00011295		R102.8	2.9170025	1.06	0.36338673	0.495348337
WBGene00013956		ZK265.3	3.448346726	1.06	0.307393683	0.414186921
WBGene00015348		C02F5.5	3.512322094	1.06	0.301794645	0.414186921
WBGene00007507		C10C5.3	3.147552603	1.0575	0.335975322	0.489403914
WBGene00012457	acbp-6	Y17G7B.1	3.024577069	1.0575	0.34963566	0.495348337
WBGene00016414		C34F11.2	3.212217278	1.0575	0.329211852	0.489403914
WBGene00002144	inx-22	Y47G6A.2	3.746791418	1.055	0.281574254	0.345525782
WBGene00009631		F42E8.2	3.589339384	1.055	0.293925953	0.414186921
WBGene00009884		F49C12.15	2.690299971	1.055	0.392149579	0.593984595
WBGene00021398		Y38C1AA.7	4.649623247	1.055	0.226900104	0.312483699
WBGene00022558	fbxb-21	ZC204.8	2.94207076	1.055	0.358590967	0.495348337
WBGene00010373		H08M01.1	3.095626603	1.0525	0.339995786	0.489403914
WBGene00018189	nhr-181	F38H12.3	5.495615017	1.0525	0.191516327	0.312483699
WBGene00018526		F47B3.2	3.14865839	1.0525	0.334269352	0.489403914
WBGene00020715	nspd-4	T23B7.1	2.964068774	1.0525	0.355086228	0.495348337
WBGene00008541		F07A5.2	3.496472239	1.05	0.300302685	0.414186921
WBGene00015765		C14C11.1	3.641011307	1.05	0.288381417	0.345525782

WBGene00009548		F38H4.4	2.71190827	1.0475	0.386259377	0.593984595
WBGene00004163	pqn-82	Y39A3CR.7	3.227052059	1.0425	0.323050258	0.489403914
WBGene00010542		K03H1.7	4.1399868	1.0425	0.251812397	0.345525782
WBGene00014104		ZK849.6	2.721100879	1.0425	0.383116998	0.593984595
WBGene00010549		K03H4.2	3.233780336	1.04	0.321605023	0.489403914
WBGene00013723		Y106G6H.13	3.324111818	1.04	0.312865528	0.463367002
WBGene00017112		E03H12.5	3.21092907	1.0375	0.323115204	0.489403914
WBGene00009864		F49B2.4	3.448364708	1.035	0.300142267	0.414186921
WBGene00009948		F52H3.6	3.649772427	1.035	0.283579325	0.345525782
WBGene00010401		H16D19.3	2.966443151	1.035	0.348902692	0.495348337
WBGene00014247		ZK1307.4	3.708607101	1.035	0.27908052	0.345525782
WBGene00018347		F42C5.5	3.286024668	1.035	0.314970247	0.463367002
WBGene00044362		F42G2.7	4.331195509	1.035	0.238964045	0.312483699
WBGene00000693	col-119	C53B4.5	2.738298956	1.0325	0.377058903	0.593984595
WBGene00009016		F21D9.2	3.57087035	1.0325	0.289145194	0.414186921
WBGene00014240	htas-1	ZK1251.1	3.440495235	1.0325	0.300102145	0.414186921
WBGene00019132	bath-19	F59H6.1	3.279877617	1.0325	0.314798331	0.463367002
WBGene00000865	cyb-1	ZC168.4	3.952070438	1.03	0.260622885	0.345525782
WBGene00011746		T13F2.6	3.71558628	1.03	0.277210626	0.345525782
WBGene00015688		C10G11.8	3.19265493	1.03	0.32261551	0.489403914
WBGene00022589		ZC317.6	3.207250403	1.03	0.32114736	0.489403914
WBGene00013809		Y116A8C.33	2.93332114	1.025	0.349433271	0.495348337
WBGene00016274		C30G12.2	9.439283044	1.025	0.108588756	0
WBGene00017055		D2062.1	3.273515373	1.025	0.313119043	0.463367002
WBGene00022763		ZK546.7	3.459391238	1.025	0.296294905	0.414186921
WBGene00001597	gld-3	T07F8.3	3.473199462	1.0225	0.294397143	0.414186921
WBGene00012509		Y26D4A.12	3.613590751	1.0225	0.282959546	0.414186921
WBGene00022433		Y108F1.5	3.917251806	1.0225	0.261024833	0.345525782
WBGene00003864	oma-1	C09G9.6	4.350864274	1.02	0.23443618	0.312483699
WBGene00009404		F35C11.5	3.657171333	1.02	0.278904078	0.345525782
WBGene00011203		R10E4.7	3.099316775	1.02	0.329104791	0.489403914
WBGene00014246		ZK1307.3	2.971029225	1.02	0.343315371	0.495348337
WBGene00015819	fbxc-44	C16A11.6	3.476117243	1.02	0.293430839	0.414186921
WBGene00007060	wht-6	T26A5.1	3.605254176	1.0175	0.28222698	0.414186921
WBGene00007763		C27B7.6	3.103303435	1.0175	0.327876413	0.489403914
WBGene00012220		W03C9.2	5.233657603	1.0175	0.194414705	0.312483699
WBGene00013829		Y116F11B.14	2.874634789	1.0175	0.353958007	0.495348337
WBGene00015193	clec-117	B0432.12	3.52493043	1.0175	0.288658179	0.414186921
WBGene00020019		R12B2.3	3.980657799	1.015	0.254982983	0.345525782
WBGene00008529		F02E9.3	3.004232255	1.0125	0.337024542	0.495348337
WBGene00013521		Y73F8A.12	3.432844839	1.0125	0.294944877	0.414186921
WBGene00013579		Y79H2A.2	4.697688449	1.0125	0.215531535	0.312483699
WBGene00017542		F17E9.5	2.815975487	1.0125	0.359555687	0.495348337
WBGene00014121		ZK858.8	3.186909802	1.0075	0.316136967	0.489403914
WBGene00016291		C31H1.8	3.647785803	1.0075	0.276194945	0.345525782
WBGene00021787		Y51H7C.9	4.684223911	1.0075	0.215083655	0.312483699
WBGene00044261		Y87G2A.20	3.392917354	1.0075	0.296942099	0.463367002
WBGene00000417	ced-3	C48D1.2	3.461853476	1.005	0.290306914	0.414186921
WBGene00001046	dnj-28	Y54E10BL.4	5.072023521	1.005	0.198145769	0.312483699
WBGene00018949	acbp-4	F56C9.5	3.867313111	1.005	0.259870347	0.345525782
WBGene00012926		Y47D3A.13	4.86501454	1.0025	0.206063105	0.312483699
WBGene00013165		Y53F4B.19	2.803287019	1.0025	0.357615896	0.495348337
WBGene00013523		Y73F8A.14	3.236511325	1.0025	0.309747101	0.489403914
WBGene00045245	ttr-34	F09A5.9	3.696413004	1.0025	0.271208872	0.345525782
WBGene00016410		C34E10.9	3.137929715	1	0.318681453	0.489403914
WBGene00004394	rol-1	Y57A10A.11	3.555391594	0.9975	0.280559813	0.414186921
WBGene00010046		F54D1.1	2.806895661	0.9975	0.355374806	0.495348337
WBGene00011834	dvc-1	T19B10.6	4.339249216	0.9975	0.229878477	0.312483699
WBGene00017325		F10C1.3	4.231364172	0.9975	0.235739577	0.312483699
WBGene00044370		C41H7.9	3.448931313	0.9975	0.289220025	0.414186921
A_12_P173977			5.307777434	0.995	0.187460762	0.312483699
WBGene00000596	col-7	C15A11.5	3.527402261	0.995	0.28207727	0.414186921
WBGene00003229	mex-3	F53G12.5	4.655212123	0.995	0.213738918	0.312483699
WBGene00007792		C28D4.4	3.669240891	0.995	0.271173256	0.345525782
WBGene00011376	gla-3	T02E1.3	4.38921125	0.995	0.22669221	0.312483699
WBGene00017436		F13H8.3	4.531693364	0.995	0.219564723	0.312483699
WBGene00012595		Y38E10A.17	3.237378989	0.9925	0.306575166	0.489403914
WBGene00013611		Y102A5C.2	3.379703921	0.9925	0.293664778	0.463367002
WBGene00018806		F54D10.5	4.491628309	0.9925	0.220966637	0.312483699
WBGene00005031	sra-5	AH6.9	3.786149508	0.99	0.261479373	0.345525782

WBGene00008243		C50H2.7	3.565196122	0.99	0.277684583	0.414186921
WBGene00018636		F49E8.2	4.430299478	0.99	0.223461191	0.312483699
A_12_P178359			3.095378358	0.9875	0.319024005	0.489403914
WBGene00001399	fat-7	F10D2.9	3.538467443	0.9875	0.279075621	0.414186921
WBGene00002368	let-99	K08E7.3	4.670116613	0.9875	0.211450823	0.312483699
WBGene00017460	bath-30	F14D2.12	3.666532469	0.9875	0.269328039	0.345525782
WBGene00020416		T10E9.6	2.951458571	0.9875	0.334580336	0.495348337
WBGene00022822		ZK813.3	4.362262627	0.9875	0.226373349	0.312483699
WBGene00077450		K06B4.15	3.784407148	0.9875	0.260939154	0.345525782
WBGene00001599	glh-2	C55B7.1	3.688292291	0.985	0.267061264	0.345525782
WBGene00011378		T02E1.6	3.28646702	0.985	0.299713946	0.463367002
WBGene00013610	fbxa-206	Y102A5C.1	3.949165992	0.985	0.249419751	0.345525782
WBGene00013647		Y105C5B.5	3.369305121	0.985	0.292345147	0.463367002
WBGene00007624	hrde-1	C16C10.3	3.11805625	0.9825	0.315100153	0.489403914
WBGene00011214		R10E9.2	4.473792305	0.9825	0.219612341	0.312483699
WBGene00015094		B0261.6	4.177345578	0.9825	0.235197204	0.312483699
WBGene00009160		F26E4.5	2.71431965	0.98	0.361048118	0.593984595
WBGene00021428		Y38F2AR.10	3.789708157	0.98	0.258595111	0.345525782
WBGene00008710		F11E6.7	4.517772254	0.9775	0.216367702	0.312483699
WBGene00011425		T04B2.7	3.099881352	0.9775	0.31533465	0.489403914
WBGene00011691		T10C6.10	4.90226736	0.9775	0.199397529	0.312483699
WBGene00018809		F54D10.8	3.528528388	0.9775	0.277027671	0.414186921
WBGene00045399		Y47G6A.33	3.573834172	0.9775	0.273515768	0.414186921
WBGene00001424	fis-1	F41G3.4	3.03117917	0.9725	0.320832239	0.495348337
WBGene00008623		F09E8.1	4.648845272	0.9725	0.209191733	0.312483699
WBGene00009652		F43D2.3	3.731829267	0.9725	0.260596059	0.345525782
WBGene00012527	set-22	Y32F6A.1	4.278324514	0.9725	0.22730861	0.312483699
WBGene00014007		ZK596.2	2.840562032	0.9725	0.342361825	0.495348337
WBGene00015820		C16A11.7	2.936550269	0.9725	0.331170902	0.495348337
WBGene00016576		C41H7.6	3.620796704	0.9725	0.268587297	0.414186921
WBGene00021537		Y42H9AR.2	3.099210433	0.9725	0.313789599	0.489403914
WBGene00015842		C16C8.4	8.093895999	0.97	0.119843398	0
WBGene00020843		T27A3.7	3.16269002	0.97	0.306700939	0.489403914
WBGene00022532		ZC155.4	5.099398092	0.97	0.190218528	0.312483699
WBGene00009031		F21H7.5	3.07569229	0.9675	0.314563327	0.489403914
WBGene00009714		F44G4.5	2.985603597	0.9675	0.324055076	0.495348337
WBGene00010061		F54E12.2	3.683670661	0.9675	0.26264563	0.345525782
WBGene00012925	wht-8	Y47D3A.11	3.155864047	0.965	0.305779966	0.489403914
WBGene00016707		C46E10.1	3.479357337	0.9625	0.276631546	0.414186921
WBGene00020905		T28H11.7	2.784732589	0.9625	0.345634624	0.495348337
WBGene00010637		K07F5.12	5.940980269	0.96	0.161589495	0.300971142
WBGene00011162	chil-19	R09D1.6	3.90032016	0.96	0.24613364	0.345525782
WBGene00015034		B0207.11	2.809603811	0.96	0.341685186	0.495348337
WBGene00016564		C41D11.5	3.77775694	0.96	0.254119049	0.345525782
WBGene00018773		F53G12.8	2.674487143	0.96	0.358947323	0.593984595
WBGene00015505		C06A5.8	3.559490737	0.9575	0.268999155	0.414186921
WBGene00017555	nep-10	F18A12.6	2.693464175	0.9575	0.355490156	0.593984595
WBGene00018792		F54C1.8	3.083062859	0.9575	0.310567784	0.489403914
WBGene00020940		W02D7.4	2.758623102	0.9575	0.347093446	0.495348337
WBGene00000751	col-178	C34F6.2	4.969061697	0.955	0.192189202	0.312483699
WBGene00002068	ify-1	C27A2.3	4.619650214	0.955	0.206725608	0.312483699
WBGene00007337		C05C12.5	4.305022907	0.955	0.221833895	0.312483699
WBGene00009463		F36D1.4	3.248712752	0.955	0.293962585	0.463367002
WBGene00011945	hpo-24	T23D8.7	4.262053344	0.955	0.224070401	0.312483699
WBGene00015032		B0207.9	2.703574289	0.955	0.353236086	0.593984595
WBGene00018991		F56F11.4	5.848755813	0.955	0.16328259	0.300971142
WBGene00010064		F54F7.2	4.007196349	0.9525	0.237697362	0.345525782
WBGene00012786		Y43C5B.3	3.432895736	0.9525	0.277462549	0.414186921
WBGene00004965	spe-11	F48C1.7	3.349805983	0.95	0.283598514	0.463367002
WBGene00008865		F15G9.1	3.254722865	0.95	0.29188353	0.463367002
WBGene00012297		W06D4.3	3.321202846	0.95	0.286040945	0.463367002
WBGene00008802	acp-2	F14E5.4	4.754805115	0.9475	0.1992721	0.312483699
WBGene00013194		Y54E2A.9	3.053886825	0.9475	0.310260352	0.495348337
WBGene00023451		K08E3.10	3.760037829	0.9475	0.251992146	0.345525782
A_12_P173631			4.239514436	0.945	0.222902885	0.312483699
WBGene00007766		C27C7.1	3.917589677	0.945	0.241219749	0.345525782
WBGene00011911		T22B3.3	3.890041123	0.945	0.242928023	0.345525782
WBGene00014032		ZK637.15	3.305258144	0.945	0.285908077	0.463367002
WBGene00018010		F33E11.2	4.630384247	0.945	0.204086734	0.312483699
WBGene00012223		W03C9.8	3.022503209	0.9425	0.311827626	0.495348337

WBGene00013800		Y116A8C.23	3.015942714	0.9425	0.312505936	0.495348337
WBGene00018011		F33E11.3	3.080572185	0.9425	0.305949656	0.489403914
WBGene00004798	sip-1	F43D9.4	3.15577366	0.94	0.297866736	0.489403914
WBGene00010544		K03H1.9	3.532182757	0.94	0.266124395	0.414186921
WBGene00016512		C38C3.3	3.067652019	0.94	0.306423282	0.495348337
WBGene00022617		ZC477.2	3.564236679	0.94	0.263731083	0.414186921
WBGene00020987		W03D8.5	2.916436654	0.9375	0.321453922	0.495348337
WBGene00006376	syp-2	C24G6.1	2.955045368	0.935	0.316408002	0.495348337
WBGene00007190	rmd-3	B0491.3	3.641154739	0.935	0.25678667	0.345525782
WBGene00010181		F57A8.6	3.270716314	0.935	0.2858701	0.463367002
WBGene00021465		Y39G10AR.7	4.130226023	0.935	0.226379863	0.345525782
WBGene00005012		F26F4.2	2.890865292	0.93	0.321702987	0.495348337
WBGene00008299		C54D10.4	2.939088145	0.93	0.316424671	0.495348337
WBGene00011491		T05F1.5	3.087004838	0.93	0.30126289	0.489403914
WBGene00012637		Y38H8A.3	3.420061249	0.93	0.271924955	0.463367002
WBGene00019255		H32C10.1	3.145896977	0.93	0.295623158	0.489403914
A_12_P173627			4.842929148	0.9275	0.191516327	0.312483699
WBGene00016825		C50E10.1	2.979272036	0.9275	0.31131766	0.495348337
WBGene00077519		T27C10.8	4.400750928	0.9275	0.210759485	0.312483699
WBGene00013371		Y60A9A.1	2.740572407	0.925	0.33752073	0.593984595
WBGene00015106		B0280.11	2.84958668	0.925	0.32460848	0.495348337
WBGene00044684		T08G11.2	2.829972156	0.925	0.32685834	0.495348337
A_12_P173630			4.349739509	0.9225	0.212081666	0.312483699
WBGene00017209		F07E5.4	2.799182633	0.9225	0.32956049	0.495348337
WBGene00018332		F42A9.3	3.311142136	0.9225	0.278604772	0.463367002
WBGene00002130	inx-8	ZK792.2	3.974537102	0.9175	0.230844492	0.345525782
WBGene00009308		F32A11.3	2.781072744	0.9175	0.329908666	0.495348337
WBGene00010883		M7.7	2.8464196	0.9175	0.322334767	0.495348337
WBGene00017910		F28H1.5	3.294589484	0.9175	0.278486896	0.463367002
WBGene00001605	gln-4	T25C8.3	3.345266622	0.915	0.273520799	0.463367002
WBGene00001977	hmg-12	Y17G7A.1	5.273843554	0.9125	0.173023714	0.312483699
WBGene00008660	clec-153	F10F2.8	3.185300233	0.9125	0.286472211	0.489403914
WBGene00016843		C50F7.3	3.248822817	0.9125	0.280870965	0.463367002
WBGene00000913	daf-18	T07A9.6	4.034222145	0.91	0.225570127	0.345525782
WBGene00008382		D1081.4	2.972444754	0.91	0.306145303	0.495348337
WBGene00018930		F56B3.6	3.721124329	0.91	0.244549743	0.345525782
WBGene00021189		Y13C8A.2	3.536871606	0.91	0.257289521	0.414186921
WBGene00000710	col-137	Y51H4A.9	6.659852147	0.9075	0.136264286	0.300971142
WBGene00007508		C10C5.4	4.424568635	0.9075	0.20510474	0.312483699
WBGene00019561		K09C6.7	2.824834446	0.9075	0.321257765	0.495348337
WBGene00050915		Y6E2A.10	2.707830064	0.9075	0.3351392	0.593984595
WBGene00007249		C01G12.9	3.132298246	0.905	0.288925233	0.489403914
WBGene00008423		D2045.5	2.928834214	0.905	0.308996664	0.495348337
WBGene00011795		T16A9.5	2.880351262	0.905	0.31419779	0.495348337
WBGene00013148		Y53F4A.2	2.85722242	0.905	0.31674118	0.495348337
WBGene00016669	ilys-2	C45G7.2	5.786012224	0.905	0.156411699	0.312483699
WBGene00019562		K09C6.8	2.804772605	0.905	0.322664304	0.495348337
WBGene00004904	snf-5	Y46G5A.30	3.321718801	0.9025	0.271696689	0.463367002
WBGene00007631	wht-3	C16C10.12	2.837467297	0.9025	0.31806534	0.495348337
WBGene00021366		Y37E11AM.2	3.373151316	0.9025	0.267553962	0.463367002
WBGene00007985	swah-1	C36F7.2	2.924553265	0.9	0.307739309	0.495348337
WBGene00016161		C27D6.3	3.135610374	0.9	0.287025457	0.489403914
WBGene00013473		Y69E1A.1	3.435553108	0.8975	0.261238867	0.414186921
WBGene00019087		F59A6.5	3.044824787	0.8975	0.294762445	0.495348337
WBGene00022858		ZK1193.2	3.119775749	0.8975	0.287680934	0.489403914
WBGene00007239		C01G10.14	2.748981747	0.895	0.325575097	0.593984595
WBGene00007445		C08F8.3	3.547197053	0.895	0.252311892	0.414186921
WBGene00013700		Y106G6D.3	3.370234475	0.895	0.265560158	0.463367002
WBGene00016336		C33C12.4	3.106159101	0.895	0.288137204	0.489403914
WBGene00017358		F10E9.7	3.252601705	0.895	0.27516434	0.463367002
WBGene00017922		F29B9.7	3.208762938	0.895	0.27892369	0.489403914
A_12_P154290			4.234684855	0.8925	0.210759485	0.312483699
WBGene00010574		K04H4.5	2.797016643	0.8925	0.319089986	0.495348337
WBGene00011597	zim-1	T07G12.6	3.230483957	0.8925	0.276274395	0.489403914
WBGene00012636		Y38H8A.2	2.921762883	0.89	0.304610619	0.495348337
WBGene00018500		F46F5.9	3.427912528	0.89	0.259633229	0.463367002
WBGene00022452		Y110A2AR.1	5.268531908	0.89	0.168927514	0.312483699
WBGene00014106		ZK856.5	2.932370193	0.8875	0.302656193	0.495348337
WBGene00015661		C09H5.7	4.385463946	0.8875	0.202373115	0.312483699
WBGene00016586		C42C1.8	3.664857932	0.8875	0.242164912	0.345525782

WBGene00018833	ztf-1	F54F2.5	2.71820632	0.8875	0.326502074	0.593984595
WBGene00006057	sss-2	F47B8.11	3.43011694	0.885	0.258008696	0.414186921
WBGene00007171		B0393.6	4.60961011	0.885	0.191990207	0.312483699
WBGene00009270		F30F8.1	4.601665062	0.885	0.19232169	0.312483699
WBGene00015677	cpg-4	C10F3.1	3.097046426	0.885	0.28575613	0.489403914
WBGene00018099	ztf-28	F36F12.8	3.363514878	0.885	0.263117611	0.463367002
WBGene00020404		T10B11.8	3.17201891	0.885	0.27900212	0.489403914
WBGene00020467		T12F5.2	3.707235801	0.885	0.238722339	0.345525782
WBGene00010612		K07A1.5	3.38702514	0.8825	0.26055313	0.463367002
WBGene00013656		Y105C5B.17	3.123925186	0.8825	0.282497162	0.489403914
WBGene00019406	acdh-8	K05F1.3	2.764820245	0.8825	0.319188924	0.495348337
WBGene00044005		F37C12.18	3.44797337	0.8825	0.255947452	0.414186921
A_12_P173628			3.586127858	0.88	0.245390024	0.414186921
WBGene00017630		F20B6.6	2.814840922	0.88	0.312628679	0.495348337
WBGene00020978		W03B1.9	3.387146529	0.88	0.259805707	0.463367002
WBGene00013190		Y54E2A.5	2.68626078	0.8775	0.326662253	0.593984595
WBGene00016799		C50C3.1	4.143152246	0.8775	0.211795258	0.312483699
WBGene00020982	ilys-6	W03D2.7	4.541055473	0.8775	0.193237014	0.312483699
WBGene00022035		Y65B4BL.3	4.492552811	0.8775	0.195323246	0.312483699
WBGene00044243		F16C3.4	2.921921777	0.8775	0.300316048	0.495348337
WBGene00044777		T02B11.9	2.800372213	0.8775	0.313351202	0.495348337
A_12_P111814			3.759950354	0.875	0.232715839	0.345525782
WBGene00001603	gln-2	K03H1.1	2.961489177	0.875	0.295459462	0.495348337
WBGene00013556		Y75B8A.23	3.116118181	0.875	0.280798079	0.489403914
WBGene00015689		C10G11.9	2.741287792	0.875	0.319193046	0.593984595
WBGene00005421	srh-211	D1065.5	2.709827023	0.8725	0.321976271	0.593984595
WBGene00011252		R11H6.4	3.200326352	0.8725	0.272628446	0.489403914
WBGene00016573		C41H7.3	4.229243525	0.8725	0.20630167	0.312483699
WBGene00020661		T21G5.4	2.906652632	0.8725	0.300173468	0.495348337
WBGene00022244		Y73B6BL.23	3.657190674	0.8725	0.238571099	0.345525782
A_12_P177537			4.080667546	0.87	0.213200411	0.345525782
WBGene00011988	har-2	T24D1.5	4.086114786	0.87	0.212916192	0.345525782
WBGene00015467	basl-1	C05D2.3	3.074383089	0.87	0.282983602	0.489403914
WBGene00021113	gsp-3	W09C3.6	4.224915799	0.87	0.205921264	0.312483699
WBGene00044638		F23A7.8	3.453951117	0.87	0.251885441	0.414186921
WBGene00015093		B0261.5	2.97873468	0.8675	0.29123104	0.495348337
WBGene00015384			2.927154725	0.8675	0.296362878	0.495348337
WBGene00016169		C27F2.7	3.432796654	0.8675	0.252709405	0.414186921
WBGene00016949		C55C2.3	2.981392986	0.8675	0.290971369	0.495348337
WBGene00021541		Y42H9B.3	3.59781763	0.8675	0.241118392	0.414186921
WBGene00000696	col-122	T05A1.2	3.304054092	0.865	0.261799588	0.463367002
WBGene00012481		Y18D10A.11	3.56488665	0.865	0.242644461	0.414186921
WBGene00019410		K05F1.9	3.440554587	0.865	0.251412956	0.414186921
WBGene00044127		BE10.5	2.805944882	0.865	0.308274053	0.495348337
A_12_P177536			3.554670125	0.8625	0.242638549	0.414186921
WBGene00007168		B0393.3	4.471044791	0.8625	0.192907931	0.312483699
WBGene00009203		F28C6.2	4.062448447	0.8625	0.212310386	0.345525782
WBGene00010081		F55A11.8	4.947090297	0.8625	0.174344907	0.312483699
WBGene00014174		ZK970.8	3.115633889	0.8625	0.276829702	0.489403914
WBGene00016670	ilys-3	C45G7.3	4.94271381	0.8625	0.17449928	0.312483699
WBGene00020219		T05A7.1	2.993805067	0.8625	0.28809491	0.495348337
WBGene00020986		W03D8.3	3.40638569	0.8625	0.253200923	0.463367002
WBGene00001482	fog-2	Y113G7B.5	3.054046434	0.86	0.281593623	0.495348337
WBGene00002136	inx-14	F07A5.1	5.474086518	0.86	0.157103838	0.312483699
WBGene00009590	ttr-4	F40F12.1	3.020227026	0.86	0.284746806	0.495348337
WBGene00011066	ztf-15	R06C7.9	4.32576966	0.86	0.198808551	0.312483699
WBGene00015097		B0273.1	2.917739191	0.86	0.294748757	0.495348337
WBGene00017450	bath-27	F14D2.1	3.257768554	0.86	0.263984376	0.463367002
WBGene00009670		F43G9.8	3.389028826	0.8575	0.253022339	0.463367002
WBGene00015134		B0304.2	3.410799597	0.8575	0.251407324	0.463367002
WBGene00001595	gld-1	T23G11.3	3.609511418	0.855	0.236874164	0.414186921
WBGene00016054		C24D10.2	2.740370374	0.855	0.312001621	0.593984595
WBGene00018083		F36A4.4	3.555048912	0.855	0.24050302	0.414186921
WBGene00019082	pup-3	F59A3.9	7.004707888	0.855	0.122060765	0.300971142
WBGene00022030		Y65B4A.7	3.21353934	0.855	0.266061781	0.489403914
WBGene00077543		F27D4.8	4.279687905	0.855	0.199780923	0.312483699
WBGene00003499	mut-2	K04F10.6	3.196194343	0.8525	0.266723456	0.489403914
WBGene00004985	spo-11	T05E11.4	3.196194343	0.8525	0.266723456	0.489403914
WBGene00006049	ssp-32	F32B6.7	4.10399156	0.8525	0.207724599	0.345525782
WBGene00010829		M02B1.4	2.997356434	0.8525	0.284417292	0.495348337

WBGene00013429		Y66D12A.3	2.882896836	0.8525	0.295709506	0.495348337
WBGene00013437		Y66D12A.11	3.108603018	0.8525	0.274238941	0.489403914
WBGene00019015		F57F4.1	3.273504249	0.8525	0.260424284	0.463367002
WBGene00022673		ZK177.8	3.949198932	0.8525	0.215866563	0.345525782
WBGene00008659	clec-151	F10F2.7	3.627255827	0.85	0.234336931	0.414186921
WBGene00010609	dut-1	K07A1.2	3.357239674	0.85	0.253184188	0.463367002
WBGene00015051		B0218.7	3.062332585	0.85	0.277566194	0.495348337
WBGene00020353		T08B6.4	2.679779572	0.85	0.317190268	0.593984595
WBGene00006039	ssp-10	K07F5.9	4.118789806	0.8475	0.205764324	0.345525782
WBGene00006311	sun-1	F57B1.2	3.489450886	0.8475	0.24287489	0.414186921
WBGene00007977	gska-3	C36B1.10	2.747066999	0.8475	0.308510859	0.593984595
WBGene00008772	irld-4	F13G11.2	4.158977093	0.8475	0.203776068	0.312483699
WBGene00015165		B0361.11	3.325553453	0.8475	0.254844799	0.463367002
WBGene00015291		C01B12.8	3.207151992	0.8475	0.264253145	0.489403914
WBGene00018950		F56C9.6	2.956058306	0.8475	0.286699352	0.495348337
WBGene00020834		T27A1.2	3.02200432	0.8475	0.280443014	0.495348337
WBGene00045479			3.048399556	0.8475	0.278014737	0.495348337
WBGene00001372	exo-3	R09B3.1	3.287867845	0.845	0.257005464	0.463367002
WBGene00012784		Y43C5A.4	3.570983498	0.845	0.236629489	0.414186921
WBGene00019254		H31G24.3	3.078049471	0.845	0.274524503	0.489403914
WBGene00000797	crn-4	AH9.2	3.082075918	0.8425	0.27335472	0.489403914
WBGene00002073	ima-2	F26B1.3	5.629348719	0.8425	0.149662073	0.312483699
WBGene00009550		F38H4.6	2.884097886	0.8425	0.292119073	0.495348337
WBGene00011805		T16G12.4	3.20050874	0.8425	0.2632394	0.489403914
WBGene00013170		Y53F4B.24	2.970196393	0.8425	0.283651277	0.495348337
WBGene00013381	cyp-31A5	Y62E10A.15	4.013995338	0.8425	0.209890627	0.345525782
WBGene00013707		Y106G6E.3	2.952296758	0.8425	0.285371041	0.495348337
WBGene00022043		Y65B4BR.8	2.879733632	0.8425	0.292561781	0.495348337
WBGene00007080	sfxn-1.1	AH6.2	3.462867967	0.84	0.242573499	0.414186921
WBGene00017057		D2062.5	3.351650528	0.84	0.250622788	0.463367002
WBGene00017808	nspd-9	F26A1.10	2.861968705	0.84	0.293504258	0.495348337
WBGene00044439		Y51H7C.15	2.700091196	0.84	0.311100603	0.593984595
WBGene00013538	tag-349	Y73F8A.34	4.316490783	0.8375	0.19402335	0.312483699
WBGene00021200	cyp-31A3	Y17G9B.3	4.621165562	0.8375	0.181231334	0.312483699
A_12_P136854			3.543431974	0.835	0.23564725	0.414186921
A_12_P177538			3.223854803	0.835	0.259006702	0.489403914
WBGene00002214	klc-1	M7.2	5.276601153	0.835	0.158245806	0.312483699
WBGene00004819	skr-13	C52D10.8	4.20602132	0.835	0.198524909	0.312483699
WBGene00011132		R08A2.1	2.953313305	0.835	0.282733294	0.495348337
WBGene00016416		C34F11.5	2.698995865	0.835	0.309374316	0.593984595
WBGene00016541		C39H7.1	3.15626615	0.835	0.264553102	0.489403914
WBGene00019139	bath-1	F59H6.9	2.736651017	0.835	0.305117457	0.593984595
WBGene00022775		ZK616.5	3.148767657	0.835	0.26518311	0.489403914
A_12_P173978			3.912735321	0.8325	0.212766756	0.345525782
WBGene00007710	rsa-1	C25A1.9	2.949349847	0.8325	0.282265599	0.495348337
WBGene00010440	ttr-51	JC8.8	5.126003884	0.8325	0.162407212	0.312483699
WBGene00044122		T28B8.6	3.829963655	0.8325	0.217364987	0.345525782
WBGene00009458		F36A2.11	2.999682317	0.83	0.276695967	0.495348337
WBGene00015026		B0207.1	3.877602566	0.83	0.214049786	0.345525782
WBGene00018954		F56C9.11	3.006151095	0.83	0.27610056	0.495348337
WBGene00022849	acs-6	ZK1127.2	3.320156657	0.83	0.249988204	0.463367002
WBGene00001855	hil-4	C18G1.5	4.491391244	0.8275	0.184241353	0.312483699
WBGene00008871	tag-314	F15H10.4	3.185402744	0.8275	0.259778768	0.489403914
WBGene00016263		C30F12.4	3.074807381	0.8275	0.269122549	0.489403914
WBGene00016322		C32E8.4	3.109265266	0.8275	0.266140046	0.489403914
WBGene00017801		F26A1.1	3.465755753	0.8275	0.238764662	0.414186921
WBGene00018660		F52C6.3	4.878944319	0.8275	0.169606363	0.312483699
WBGene00021633		Y47G6A.3	2.806303138	0.8275	0.294871922	0.495348337
WBGene00044979		Y41E3.19	3.166562156	0.8275	0.261324414	0.489403914
A_12_P172784			5.169247853	0.825	0.159597687	0.312483699
WBGene00018119		F36H12.3	2.689460562	0.825	0.306752964	0.593984595
A_12_P136850			3.270997128	0.8225	0.251452376	0.463367002
WBGene00007572		C14A6.8	3.530648905	0.8225	0.232960009	0.414186921
WBGene00010114		F55D12.6	2.703782226	0.8225	0.304203494	0.593984595
WBGene00011919		T22C1.9	3.260506213	0.8225	0.252261442	0.463367002
A_12_P164704			3.404442549	0.82	0.240861753	0.463367002
A_12_P177535			3.084654655	0.82	0.265832027	0.489403914
WBGene00002225	klp-15	M01E11.6	4.420918594	0.82	0.185481814	0.312483699
WBGene00007633		C16D2.1	3.993718191	0.82	0.205322449	0.345525782
WBGene00021649		Y47G6A.25	3.051313881	0.82	0.268736692	0.495348337

WBGene00021877		Y54G2A.12	3.847171368	0.82	0.213143612	0.345525782
A_12_P136851			3.859859785	0.8175	0.211795258	0.345525782
A_12_P181510			3.033020171	0.8175	0.269533321	0.495348337
WBGene00001514	xnd-1	C05D2.5	4.294080841	0.8175	0.190378344	0.312483699
WBGene00007714		C25D7.1	3.252278189	0.8175	0.251362261	0.463367002
WBGene00011954		T23F11.2	2.878946818	0.8175	0.283958007	0.495348337
WBGene00016632		C44B7.12	2.89541635	0.8175	0.282342814	0.495348337
WBGene00021038		W05F2.6	2.883620773	0.8175	0.28349775	0.495348337
A_12_P177534			3.777311106	0.815	0.215761947	0.345525782
WBGene00000099	air-2	B0207.4	3.772436279	0.815	0.21604076	0.345525782
WBGene00004044	plk-3	F55G1.8	5.257684867	0.815	0.155011192	0.312483699
WBGene00007148		B0334.10	2.945260241	0.815	0.276715785	0.495348337
WBGene00007778		C27D8.2	3.496108425	0.815	0.23311634	0.414186921
WBGene00008312		C54G4.2	3.009528219	0.815	0.270806565	0.495348337
WBGene00009365		F33H1.4	3.901260709	0.815	0.208906828	0.345525782
WBGene00010538	ttr-3	K03H1.3	3.005458619	0.815	0.271173256	0.495348337
WBGene00011466		T05C12.1	2.765580608	0.815	0.294693996	0.495348337
WBGene00012715		Y39E4B.2	2.813515857	0.815	0.28967315	0.495348337
WBGene00021470	tpxl-1	Y39G10AR.12	4.342794229	0.815	0.187667192	0.312483699
WBGene00010409		H21P03.2	2.680817531	0.8125	0.303079188	0.593984595
WBGene00011915		T22C1.4	3.108145199	0.8125	0.261409924	0.489403914
WBGene00017854		F27C1.4	3.845638708	0.8125	0.211278298	0.345525782
WBGene00020247		T05B11.4	4.072215479	0.8125	0.199522841	0.345525782
WBGene00003183	mei-1	T01G9.5	3.486277765	0.81	0.232339491	0.414186921
WBGene00010474		K01D12.15	3.034192161	0.81	0.266957383	0.495348337
WBGene00013894		ZC434.8	2.849988328	0.81	0.28421169	0.495348337
WBGene00015747		C13G5.2	3.620494332	0.81	0.223726355	0.414186921
WBGene00018125	rmd-4	F36H12.11	3.628239998	0.81	0.223248738	0.414186921
WBGene00018772		F53G12.4	2.919468747	0.81	0.277447738	0.495348337
WBGene00003067	lrg-1	F55H2.4	3.202183018	0.8075	0.25217172	0.489403914
WBGene00009921		F52B5.2	4.005978995	0.8075	0.201573698	0.345525782
WBGene00012877		Y45F10C.1	4.464588724	0.8075	0.180867724	0.312483699
WBGene00013085	mpz-6	Y51B9A.3	4.222233271	0.8075	0.1912495	0.312483699
WBGene00018336		F42A9.7	5.103186875	0.8075	0.158234456	0.312483699
WBGene00008385		D1081.7	3.313094794	0.805	0.242975239	0.463367002
WBGene00009501		F37A8.1	3.009613025	0.805	0.267476248	0.495348337
WBGene00010781		K11H3.4	3.418267415	0.805	0.235499422	0.463367002
WBGene00022034	deps-1	Y65B4BL.2	3.830960197	0.805	0.210130087	0.345525782
WBGene00022830		ZK973.1	3.916658805	0.805	0.205532327	0.345525782
A_12_P173496			4.177193898	0.8025	0.192114616	0.312483699
WBGene00000386	cdc-25.1	K06A5.7	3.133794394	0.8025	0.25607934	0.489403914
WBGene00002061	ife-3	B0348.6	2.977368425	0.8025	0.269533321	0.495348337
WBGene00008210		C49F5.6	2.994761327	0.8025	0.267967932	0.495348337
WBGene00012547		Y37D8A.5	2.858766816	0.8025	0.280715445	0.495348337
WBGene00015449	ugt-63	C04F5.7	4.379343754	0.8025	0.183246634	0.312483699
WBGene00003912	pal-1	C38D4.6	4.048444143	0.8	0.197606777	0.345525782
WBGene00010195	pot-2	F57C2.3	2.929786299	0.8	0.273057458	0.495348337
WBGene00002072	ima-1	T19B10.7	3.758316526	0.7975	0.212196071	0.345525782
WBGene00009372	evl-18	F34D10.2	3.508446067	0.7975	0.22730861	0.414186921
WBGene00011749	ssp-36	T13F2.12	3.193997046	0.7975	0.249687144	0.489403914
WBGene00012650	orc-1	Y39A1A.12	2.97243559	0.7975	0.268298497	0.495348337
WBGene00015997		C18H7.7	3.46308632	0.7975	0.230285914	0.414186921
WBGene00020588		T19H12.2	3.098897912	0.7975	0.257349555	0.489403914
WBGene00010184		F57A10.2	3.573416455	0.795	0.222476168	0.414186921
WBGene00012180		W01D2.3	2.949486421	0.795	0.269538451	0.495348337
WBGene00014915		Y53C10A.1	2.75336251	0.795	0.28873786	0.495348337
WBGene00015696		C10H11.7	3.377218799	0.795	0.235400798	0.463367002
WBGene00016741		C48B6.3	4.142268842	0.795	0.191923806	0.312483699
WBGene00016950		C55C2.4	3.854554458	0.795	0.206249518	0.345525782
WBGene00019024		F58A6.5	3.287963255	0.795	0.241791023	0.463367002
WBGene00020091	rnp-8	R119.7	4.753460875	0.795	0.167246564	0.312483699
WBGene00021447		Y39A3CR.8	2.869698187	0.795	0.277032617	0.495348337
WBGene00023427		F14D2.15	2.936116952	0.795	0.270765781	0.495348337
WBGene00016181		C28C12.11	2.866465526	0.7925	0.276472887	0.495348337
WBGene00017384		F11G11.4	2.753346438	0.7925	0.28783156	0.495348337
WBGene00017461	bath-28	F14D2.13	3.631224115	0.7925	0.218245962	0.414186921
WBGene00021720		Y49F6B.8	3.403334232	0.7925	0.23285988	0.463367002
A_12_P136852			2.890603492	0.79	0.273299331	0.495348337
WBGene00004086	pph-4.2	Y49E10.3	3.714353632	0.79	0.212688419	0.345525782
WBGene00007771		C27C12.1	3.715349316	0.79	0.21263142	0.345525782

WBGene00009182		F26H11.4	3.376943653	0.79	0.233939349	0.463367002
WBGene00010035		F54C8.1	2.735158106	0.79	0.288831566	0.593984595
WBGene00010623		K07A12.5	2.714135115	0.79	0.291068781	0.593984595
WBGene00014053		ZK669.3	5.064302315	0.79	0.155993847	0.312483699
WBGene00017451		F14D2.2	3.898223955	0.79	0.202656392	0.345525782
WBGene00022094		Y69A2AR.23	3.900580311	0.79	0.202533966	0.345525782
WBGene00002229	klp-19	Y43F4B.6	4.123423067	0.7875	0.190982101	0.345525782
WBGene00006440	tag-63	Y47G6A.28	4.158624628	0.7875	0.189365492	0.312483699
WBGene00008074	nkb-2	C43F9.6	2.906284486	0.7875	0.270964527	0.495348337
WBGene00011275		R53.6	3.635903726	0.7875	0.216589893	0.414186921
WBGene00015851		C16C8.13	3.494096413	0.7875	0.225380158	0.414186921
WBGene00002120	ins-37	F08G2.6	3.485336466	0.785	0.225229331	0.414186921
WBGene00006060	sth-1	ZC513.12	3.961729815	0.785	0.198145769	0.345525782
WBGene00013657		Y105C5B.18	3.605832588	0.785	0.217702841	0.414186921
WBGene00009215	thn-2	F28D1.5	2.759789264	0.7825	0.283536142	0.495348337
WBGene00009938		F52F12.5	2.753461202	0.7825	0.28418777	0.495348337
WBGene00016381	sgo-1	C33H5.15	3.022230735	0.7825	0.258914712	0.495348337
WBGene00021188		Y13C8A.1	2.984615419	0.7825	0.262177832	0.495348337
WBGene00004721	san-1	ZC328.4	3.985967623	0.78	0.195686487	0.345525782
WBGene00019712		M01E11.3	3.253915635	0.78	0.239711193	0.463367002
WBGene00020064	kbp-1	R13F6.1	3.830441812	0.78	0.203631862	0.345525782
WBGene00021908		Y55B1AR.4	3.03779985	0.78	0.256764777	0.495348337
WBGene00004244	puf-8	C30G12.7	2.809792438	0.7775	0.276710831	0.495348337
WBGene00008124		C47A4.3	3.141529359	0.7775	0.247490923	0.489403914
WBGene00012435	flh-1	Y11D7A.12	3.761159244	0.7775	0.206718182	0.345525782
WBGene00013879		ZC376.8	2.705127933	0.7775	0.28741709	0.593984595
WBGene00018659		F52C6.2	3.72606259	0.7775	0.208665309	0.345525782
WBGene00019951		R08C7.8	2.714409256	0.7775	0.286434331	0.593984595
A_12_P164706			3.098177582	0.775	0.250147056	0.489403914
WBGene00004132	ifet-1	F56F3.1	3.957158538	0.775	0.195847599	0.345525782
WBGene00044633		F54H12.7	3.697393605	0.775	0.209607113	0.345525782
A_12_P159807			3.016125647	0.7725	0.256123282	0.495348337
A_12_P173629			3.030075879	0.77	0.254119049	0.495348337
WBGene00001839	hdl-1	ZK829.2	3.919412665	0.77	0.196458007	0.345525782
WBGene00003430	msp-32	R05F9.3	4.449000596	0.77	0.173072577	0.312483699
WBGene00008561		F07H5.10	4.569188448	0.77	0.168520079	0.312483699
WBGene00015084		B0244.9	3.380828342	0.77	0.227754835	0.463367002
WBGene00017955		F31E8.5	2.977627837	0.77	0.258595111	0.495348337
WBGene00000935	daz-1	F56D1.7	4.390547285	0.7675	0.174807365	0.312483699
WBGene00006940	wee-1.3	Y53C12A.1	3.217723432	0.7675	0.238522675	0.489403914
WBGene00007584		C14C10.1	2.716841451	0.7675	0.282497162	0.593984595
WBGene00010227		F58A4.2	3.633639322	0.7675	0.211220744	0.414186921
WBGene00011642		T09B9.1	6.040329747	0.7675	0.1270626	0.300971142
WBGene00013712	dlc-6	Y106G6G.3	2.690558019	0.7675	0.285256811	0.593984595
WBGene00015516		C06A8.6	3.433479758	0.7675	0.223534156	0.414186921
WBGene00016387	kbp-5	C34B2.2	4.124863927	0.7675	0.186066744	0.345525782
A_12_P161664			2.696927979	0.765	0.283656073	0.593984595
A_12_P161667			2.817693536	0.765	0.271498653	0.495348337
WBGene00001499	fsn-1	C26E6.5	3.936041495	0.765	0.194357707	0.345525782
WBGene00014229		ZK1128.3	2.829586836	0.765	0.270357492	0.495348337
WBGene00014672		C08F11.6	3.700557501	0.765	0.206725608	0.345525782
WBGene00018016	lrr-1	F33G12.4	2.744229776	0.765	0.278766744	0.593984595
A_12_P164707			3.303089417	0.7625	0.230844492	0.463367002
A_12_P171289			2.957384698	0.7625	0.257829156	0.495348337
WBGene00008387		D1081.9	4.292952735	0.7625	0.177616677	0.312483699
WBGene00018001		F33D11.2	3.028047899	0.7625	0.251812397	0.495348337
WBGene00018163		F38A5.6	3.48240291	0.7625	0.218958007	0.414186921
WBGene00020461		T12C9.7	3.860691421	0.7625	0.197503482	0.345525782
WBGene00022083		Y69A2AR.12	2.88549073	0.7625	0.264253145	0.495348337
WBGene00001862	him-3	ZK381.1	3.205478713	0.76	0.237094072	0.489403914
WBGene00000098	air-1	K07C11.2	3.533891047	0.7575	0.214352958	0.414186921
WBGene00008417		D2030.7	5.14888132	0.7575	0.147119336	0.312483699
WBGene00010728		K09G1.2	3.23776053	0.7575	0.233958007	0.489403914
WBGene00001511	fzy-1	ZK177.6	3.040390787	0.755	0.248323342	0.495348337
WBGene00002125	inx-3	F22F4.2	4.771058528	0.755	0.158245806	0.312483699
WBGene00006974	zen-4	M03D4.1	3.839930058	0.755	0.196618165	0.345525782
WBGene00009488	oac-20	F36G9.12	2.822680539	0.755	0.267476248	0.495348337
WBGene00011241	mpz-5	R11A8.8	2.777952013	0.755	0.271782953	0.495348337
WBGene00017696	polk-1	F22B7.6	3.160110038	0.755	0.238915731	0.489403914
WBGene00018897		F55F8.9	3.377459695	0.755	0.223540788	0.463367002

WBGene00019833		R02F2.4	2.700388289	0.755	0.279589422	0.593984595
WBGene00020103		R148.4	4.059013961	0.755	0.186005766	0.345525782
A_12_P172783			4.017311512	0.7525	0.187314326	0.345525782
WBGene00004823	skr-17	C06A8.4	3.266234593	0.7525	0.230387616	0.463367002
WBGene00007777		C27D8.1	2.775864331	0.7525	0.271086736	0.495348337
WBGene00011513	fbxa-197	T06C12.4	2.752427867	0.7525	0.273394994	0.593984595
WBGene00018380	decr-1.1	F43C11.3	3.538632037	0.7525	0.212652797	0.414186921
WBGene00022650		ZK84.2	2.839514879	0.7525	0.265010057	0.495348337
WBGene00001214	ego-1	F26A3.3	3.29152178	0.75	0.227858131	0.463367002
WBGene00004339	rfc-3	C39E9.13	2.865954624	0.75	0.261692908	0.495348337
WBGene00007244		C01G12.3	3.003417085	0.75	0.249715567	0.495348337
WBGene00021880		Y54G2A.15	3.597684884	0.75	0.208467396	0.414186921
WBGene00003037	lin-54	JC8.6	2.718581148	0.7475	0.274959606	0.593984595
WBGene00014752		F38E11.8	3.33846813	0.7475	0.223905088	0.463367002
WBGene00017559	mpz-3	F18C5.4	3.131332157	0.7475	0.238716292	0.489403914
WBGene00018327		F42A6.5	3.978927547	0.7475	0.187864692	0.345525782
WBGene00019595		K09H9.2	3.684757569	0.7475	0.202862735	0.345525782
A_12_P149955			2.690752104	0.745	0.276874261	0.593984595
A_12_P164705			2.72054179	0.745	0.273842513	0.593984595
A_12_P177573			3.135441509	0.745	0.237606091	0.489403914
WBGene00002992	lin-3	F36H1.4	3.235019532	0.745	0.230292273	0.489403914
WBGene00007376		C06C3.5	2.742386895	0.745	0.271661158	0.593984595
WBGene00011410		T04A8.8	3.441334545	0.745	0.216485782	0.414186921
WBGene00012273		W05B2.7	4.524749589	0.745	0.164649996	0.312483699
WBGene00012638		Y38H8A.4	3.194459188	0.745	0.233216315	0.489403914
WBGene00012689		Y39B6A.30	2.805390708	0.745	0.265560158	0.495348337
WBGene00014851		W01G7.2	3.680065035	0.745	0.202442074	0.345525782
WBGene00018498		F46F5.7	3.139950123	0.745	0.237264915	0.489403914
WBGene00018613	skpt-1	F48E8.7	3.611086155	0.745	0.206309118	0.414186921
WBGene00019431		K06A5.3	4.524749589	0.745	0.164649996	0.312483699
A_12_P159809			2.800007425	0.7425	0.265177868	0.495348337
WBGene00002212	kin-31	B0523.1	5.98294548	0.7425	0.124102752	0.300971142
WBGene00007733		C25G4.6	2.931420736	0.7425	0.253290151	0.495348337
WBGene00022036		Y65B4BL.4	4.470956544	0.7425	0.166071845	0.312483699
A_12_P171291			3.024233939	0.74	0.244690065	0.495348337
WBGene00007610		C15H7.3	2.736496259	0.74	0.270418787	0.593984595
WBGene00008493		F01D5.2	2.748155161	0.74	0.26927155	0.593984595
WBGene00010242		F58D5.2	3.815741068	0.74	0.193933495	0.345525782
WBGene00008219		C50B6.3	3.5195874	0.7375	0.209541607	0.414186921
WBGene00009006		F21D5.1	3.930009109	0.7375	0.187658598	0.345525782
WBGene00009163	drsh-1	F26E4.10	3.833566366	0.7375	0.192379609	0.345525782
WBGene00009535		F38C2.1	3.73577833	0.735	0.196746149	0.345525782
WBGene00009753	scrm-6	F46A8.10	2.848741188	0.735	0.258008696	0.495348337
WBGene00010621	egg-6	K07A12.2	2.794778323	0.735	0.262990447	0.495348337
WBGene00007500	nasp-1	C09H10.6	2.769756423	0.7325	0.264463689	0.495348337
WBGene00011038		R05H5.3	3.917760274	0.7325	0.186969071	0.345525782
WBGene00012191		W02A2.8	2.914120824	0.7325	0.251362261	0.495348337
WBGene00015894	acdh-2	C17C3.12	3.930878647	0.7325	0.186345106	0.345525782
A_12_P108807			3.063852034	0.73	0.238262159	0.495348337
WBGene00012905		Y46G5A.14	2.868666945	0.7275	0.253602113	0.495348337
WBGene00015816		C16A11.3	3.020180156	0.7275	0.24087967	0.495348337
WBGene00018494		F46F5.2	2.95327807	0.7275	0.246336438	0.495348337
WBGene00019151	pck-3	H04M03.1	3.051263942	0.7275	0.238425785	0.495348337
WBGene00001829	hcp-1	ZK1055.1	3.120771216	0.725	0.23231437	0.489403914
WBGene00013857	orc-5	ZC168.3	3.65892244	0.725	0.198145769	0.345525782
WBGene00022357	kbp-4	Y92C3B.1	2.890967545	0.725	0.250781093	0.495348337
WBGene00000405	cdk-1	T05G5.3	4.054096262	0.7225	0.178214811	0.345525782
WBGene00009185		F27C8.5	2.90958015	0.7225	0.248317614	0.495348337
WBGene00016449		C35D10.13	3.621139303	0.7225	0.199522841	0.414186921
WBGene00018548	clec-79	F47C12.4	2.714736139	0.7225	0.266140046	0.593984595
WBGene00020375		T09B4.1	3.28744578	0.7225	0.219775488	0.463367002
WBGene00020858	mop-25.3	T27C10.3	3.213935022	0.7225	0.224802305	0.489403914
WBGene00002086	ins-3	ZK75.3	6.023578064	0.72	0.119530285	0.300971142
WBGene00012512	ekl-5	Y26E6A.1	5.784015661	0.72	0.124480991	0.312483699
WBGene00014755		F40F12.8	2.827397181	0.72	0.25465117	0.495348337
A_12_P164708			2.842755661	0.7175	0.252395944	0.495348337
A_12_P172782			4.704375744	0.7175	0.152517579	0.312483699
WBGene00006719	ubc-24	F49E12.4	4.288218881	0.7175	0.167318885	0.312483699
WBGene00017672		F21F3.2	2.683781449	0.7175	0.267346658	0.593984595
WBGene00019789		M116.2	3.281803533	0.7175	0.218629785	0.463367002

WBGene00020105		R148.7	2.792304061	0.7175	0.256956257	0.495348337
WBGene00016971	toe-2	C56E6.3	3.653201803	0.715	0.195718725	0.345525782
WBGene00000264	brc-1	C36A4.8	2.729177575	0.7125	0.261067659	0.593984595
WBGene00000804	csc-1	Y48E1B.12	3.882163103	0.7125	0.183531702	0.345525782
WBGene00000871	cye-1	C37A2.4	3.353230074	0.7125	0.212481692	0.463367002
WBGene00009470		F36D3.4	3.454678341	0.7125	0.206242066	0.414186921
WBGene00010634		K07F5.6	2.673392582	0.7125	0.26651529	0.593984595
WBGene00015327	snpn-1	C02B10.2	3.633407243	0.7125	0.196096928	0.414186921
WBGene00018902		F55G1.6	3.636986549	0.7125	0.195903941	0.345525782
WBGene00045247		F54H12.8	2.675065666	0.7125	0.266348602	0.593984595
A_12_P166050			5.185033072	0.71	0.136932588	0.312483699
WBGene00003222	mes-4	Y2H9A.1	6.749829622	0.71	0.10518784	0.300971142
WBGene00015931		C17H12.5	2.765648497	0.71	0.256720983	0.495348337
WBGene00002297	ect-2	T19E10.1	3.604367716	0.7075	0.196289629	0.414186921
WBGene00014681		C25D7.14	5.604764541	0.7075	0.126231886	0.312483699
A_12_P172785			4.104206355	0.705	0.171774989	0.345525782
WBGene00001998	hpr-17	F32A11.2	2.854904482	0.705	0.246943463	0.495348337
WBGene00005013	jmjd-1.1	F43G6.6	4.729272145	0.705	0.149071565	0.312483699
WBGene00012565	fbxa-210	Y37H2A.5	4.654755565	0.705	0.151458007	0.312483699
WBGene00018668	bath-2	F52C6.11	3.016490659	0.705	0.233715294	0.495348337
WBGene00014084	snb-7	ZK795.4	3.576557276	0.7025	0.196417937	0.414186921
WBGene00017446		F14B8.4	3.851695233	0.7025	0.182387224	0.345525782
A_12_P161219			2.930792668	0.7	0.238843234	0.495348337
WBGene00001831	hcp-3	F58A4.3	3.639108755	0.7	0.19235479	0.345525782
WBGene00015241		B0524.2	3.58306151	0.7	0.195363657	0.414186921
WBGene00022674		ZK177.9	2.878995064	0.7	0.243140396	0.495348337
WBGene00045484		F34D10.9	3.672191309	0.7	0.190621877	0.345525782
WBGene00008174		C48B4.11	3.141303455	0.6975	0.222041586	0.489403914
WBGene00018830	ska-3	F54E7.8	3.25912507	0.6975	0.214014493	0.463367002
WBGene00001864	him-5	D1086.4	2.859818182	0.695	0.243022443	0.495348337
WBGene00009129		F25H5.7	2.717211103	0.695	0.255776962	0.593984595
WBGene00016807		C50D2.3	3.348047689	0.6925	0.206836958	0.463367002
WBGene00022890	fbxa-224	ZK1290.9	2.793411705	0.6925	0.247904739	0.495348337
WBGene00003590	nex-3	C28A5.3	3.350795289	0.69	0.205921264	0.463367002
WBGene00015796		C15F1.5	2.817204006	0.69	0.24492369	0.495348337
WBGene00017647		F20H11.4	2.714314394	0.69	0.25420784	0.593984595
WBGene00021743		Y50D4B.4	2.906648046	0.69	0.237386842	0.495348337
A_12_P173474			4.084833258	0.6875	0.168305524	0.345525782
WBGene00006411	octr-1	F14D12.6	3.200606416	0.6875	0.214803044	0.489403914
WBGene00009272	gras-1	F30F8.3	5.07214226	0.6875	0.135544305	0.312483699
WBGene00011560		T07C4.3	3.841683378	0.6875	0.178958007	0.345525782
WBGene00011910		T22B3.2	2.731182617	0.6875	0.251722457	0.593984595
WBGene00013886		ZC412.5	3.555389457	0.6875	0.193368408	0.414186921
WBGene00019980	chil-14	R09B5.12	3.830609909	0.6875	0.179475336	0.345525782
WBGene00022229		Y73B6A.2	2.964567933	0.6875	0.231905632	0.495348337
WBGene00012795		Y43E12A.3	2.745371897	0.685	0.249510822	0.593984595
WBGene00019608	ani-2	K10B2.5	3.397091785	0.685	0.201643065	0.463367002
WBGene00019692		M01A10.1	2.699127592	0.685	0.253785705	0.593984595
WBGene00001856	hil-5	B0414.3	4.016382906	0.6825	0.169929017	0.345525782
WBGene00003839	ocr-2	T09A12.3	4.678826572	0.6825	0.145869908	0.312483699
WBGene00008921		F17C11.10	3.300647931	0.6825	0.206777583	0.463367002
WBGene00013214		Y54G11A.3	3.257109696	0.6825	0.209541607	0.463367002
A_12_P159901			3.92367733	0.68	0.17330681	0.345525782
WBGene00013481		Y69H2.3	2.682956232	0.68	0.253451768	0.593984595
WBGene00015011		B0041.8	3.010393907	0.68	0.225884061	0.495348337
WBGene00002064	iff-1	T05G5.10	3.498927013	0.6775	0.19363079	0.414186921
WBGene00010263	wago-4	F58G1.1	3.552437419	0.6775	0.190714127	0.414186921
A_12_P180119			4.138303841	0.675	0.163110305	0.345525782
WBGene00015634		C09D4.3	4.018144882	0.675	0.16798797	0.345525782
WBGene00018416	retr-1	F44E2.2	2.8567033	0.675	0.236286352	0.495348337
WBGene00021175	math-46	Y8G1A.1	2.729340848	0.675	0.24731246	0.593984595
WBGene00021563		Y45G12B.2	3.689133221	0.675	0.182969809	0.345525782
WBGene00044188		F28F8.9	3.242942829	0.675	0.20814428	0.463367002
WBGene00001609	glp-1	F02A9.6	2.856306104	0.6725	0.235443953	0.495348337
WBGene00003835	nxf-2	C15H11.6	3.246699001	0.6725	0.207133461	0.463367002
WBGene00009444		F35G12.11	3.299208946	0.6725	0.203836741	0.463367002
WBGene00010371		H06O01.4	3.569294675	0.6725	0.188412575	0.414186921
WBGene00022218		Y73B3A.16	3.073667691	0.6725	0.218793984	0.495348337
WBGene00008172		C48B4.9	2.728794257	0.67	0.245529687	0.593984595
WBGene00012276	his-74	W05B10.1	4.427971494	0.67	0.151310821	0.312483699

WBGene00013719		Y106G6H.6	2.87071753	0.67	0.233391127	0.495348337
WBGene00021953		Y55H10A.2	4.279767732	0.67	0.156550552	0.312483699
WBGene00044696		F52E1.14	4.776089711	0.67	0.140282122	0.312483699
A_12_P108193			3.178468825	0.6675	0.210006779	0.489403914
WBGene00000864	cya-2	F59H6.7	4.240421896	0.6675	0.157413582	0.312483699
WBGene00007092		B0001.7	2.758548297	0.6675	0.2419751	0.495348337
WBGene00008266		C53A5.6	3.085819019	0.6675	0.216312102	0.489403914
WBGene00015524		C06E1.9	5.170763067	0.6675	0.129091198	0.312483699
WBGene00015839	math-10	C16C4.15	4.753015156	0.6675	0.140437171	0.312483699
WBGene00045434		Y95D11A.3	4.357255648	0.6675	0.153192756	0.312483699
WBGene00000517	cki-2	T05A6.2	4.986747714	0.665	0.133353448	0.312483699
WBGene00018409		F44B9.8	2.753559117	0.665	0.241505619	0.495348337
WBGene00019459		K06H7.8	2.891470275	0.665	0.229986801	0.495348337
WBGene00020187	gsp-4	T03F1.5	3.827670623	0.665	0.173734907	0.345525782
WBGene00020922		W01H2.2	3.162960803	0.665	0.210246045	0.489403914
WBGene00012741		Y40H4A.2	2.885801991	0.6625	0.229572231	0.495348337
WBGene00017700		F22D3.4	2.978651712	0.6625	0.222416067	0.495348337
WBGene00020120		R160.3	2.9046334	0.6625	0.228083861	0.495348337
WBGene00017691	ilys-5	F22A3.6	2.859931302	0.66	0.230774774	0.495348337
WBGene00020819		T26A5.2	4.529063625	0.66	0.145725486	0.312483699
WBGene00050912		F43E2.12	3.204178969	0.66	0.205981004	0.489403914
WBGene00011166	chil-22	R09D1.10	2.742677649	0.6575	0.23972923	0.593984595
WBGene00018909	slx-1	F56A3.2	3.008874878	0.6575	0.21852022	0.495348337
WBGene00021006	dct-9	W03F11.3	3.270859938	0.6575	0.201017473	0.463367002
WBGene00004899	snb-5	C30A5.5	2.896703472	0.655	0.226119106	0.495348337
WBGene00013726		Y106G6H.16	4.076800062	0.655	0.160665225	0.345525782
WBGene00015146	abi-1	B0336.6	4.175333049	0.655	0.156873713	0.312483699
WBGene00016072		C24H12.2	3.330245907	0.655	0.196682172	0.463367002
WBGene00017132	mel-47	EEED8.1	2.76632374	0.655	0.236776336	0.495348337
WBGene00018666	bath-6	F52C6.9	3.59249956	0.655	0.182324309	0.414186921
WBGene00022735		ZK418.6	3.136247465	0.655	0.208848315	0.489403914
A_12_P149956			3.151044253	0.6525	0.20707421	0.489403914
WBGene00000885	cyn-9	T27D1.1	2.760405307	0.6525	0.236378331	0.495348337
WBGene00001872	him-14	ZK1127.11	2.964544559	0.6525	0.220101262	0.495348337
WBGene00011352	rskn-1	T01H8.1	2.703466548	0.6525	0.241356787	0.593984595
WBGene00019247	syp-4	H27M09.3	3.573332152	0.6525	0.182602672	0.414186921
WBGene00020597		T20B6.1	4.184418175	0.6525	0.155935658	0.312483699
WBGene00007644		C17E4.4	4.029100355	0.65	0.161326337	0.345525782
WBGene00012182	osta-3	W01D2.5	2.944047243	0.65	0.220784501	0.495348337
WBGene00013704		Y106G6D.8	3.988197675	0.65	0.162980888	0.345525782
A_12_P177572			2.775871999	0.6475	0.233260035	0.495348337
WBGene00012651	orc-4	Y39A1A.13	3.654765368	0.6475	0.177165956	0.345525782
WBGene00015267	chin-1	BE0003N10.2	3.078134641	0.6475	0.210354671	0.489403914
WBGene00021192		Y14H12B.2	4.667414929	0.6475	0.138727756	0.312483699
WBGene00021878		Y54G2A.13	3.481237971	0.6475	0.185997052	0.414186921
A_12_P159904			3.450885475	0.645	0.18690855	0.414186921
WBGene00012924		Y47D3A.5	2.853137674	0.645	0.226066904	0.495348337
WBGene00014777		F58A4.12	3.20657803	0.645	0.201149011	0.489403914
WBGene00021559		Y45G5AM.7	5.457849752	0.645	0.118178409	0.312483699
A_12_P159900			2.964916627	0.6425	0.216700866	0.495348337
WBGene00001110	duo-1	F38B7.5	3.481931524	0.6425	0.184524019	0.414186921
WBGene00004333	rec-8	W02A2.6	4.401445341	0.6425	0.145974777	0.312483699
WBGene00012566	fbxa-211	Y37H2A.6	3.245804109	0.6425	0.197947867	0.463367002
WBGene00015843		C16C8.5	2.893602107	0.6425	0.222041586	0.495348337
WBGene00021460	zwl-1	Y39G10AR.2	4.250713919	0.6425	0.15115108	0.312483699
WBGene00021227	mct-1	Y19D10A.12	2.856582225	0.64	0.224043962	0.495348337
WBGene00000144	apc-10	F15H10.3	3.45483478	0.6375	0.184524019	0.414186921
WBGene00003157	mcm-5	R10E4.4	2.985081686	0.6375	0.213561995	0.495348337
WBGene00003764	nlp-26	Y43F8C.2	3.553480474	0.6375	0.179401577	0.414186921
WBGene00007785		C27H6.3	3.595268121	0.6375	0.1773164	0.414186921
WBGene00008546		F07A11.2	4.379803751	0.6375	0.145554467	0.312483699
WBGene00017992		F32E10.5	3.983557296	0.6375	0.160032843	0.345525782
A_12_P136540			3.987713793	0.635	0.159239111	0.345525782
WBGene00008580		F08G5.1	3.329444444	0.635	0.19072251	0.463367002
WBGene00010093	capg-2	F55C5.4	4.181778605	0.635	0.151849263	0.312483699
WBGene00011985		T24D1.2	3.754494477	0.635	0.169130626	0.345525782
WBGene00044290		B0035.18	2.689652692	0.635	0.236089961	0.593984595
A_12_P162828			5.620567197	0.6325	0.112533127	0.312483699
A_12_P177571			2.870144049	0.6325	0.220372214	0.495348337
WBGene00007998		C38C6.5	3.867798567	0.6325	0.163529716	0.345525782

WBGene00013669		Y105E8A.8	3.765380553	0.6325	0.167977709	0.345525782
WBGene00017279		F09C12.8	2.90911334	0.6325	0.217420199	0.495348337
WBGene00015262		B0563.5	3.568722931	0.63	0.176533738	0.414186921
WBGene00015351		C02F5.10	2.828033209	0.63	0.222769661	0.495348337
WBGene00017827		F26F4.5	2.883299579	0.63	0.218499668	0.495348337
WBGene00018302		F41G3.6	6.979621968	0.63	0.090262768	0.300971142
WBGene00018585		F48A11.4	3.340547977	0.63	0.188591813	0.463367002
WBGene00044039		C47G2.7	3.045331128	0.63	0.206874055	0.495348337
A_12_P113956			3.367407988	0.6275	0.186345106	0.463367002
WBGene00001598	glh-1	T21G5.3	3.552467571	0.6275	0.176637784	0.414186921
WBGene00010666		K08E4.2	3.369923523	0.6275	0.186206006	0.463367002
WBGene00011155		R09B3.2	4.933178146	0.6275	0.127199947	0.312483699
WBGene00017397		F12A10.8	4.119516625	0.6275	0.152323696	0.345525782
WBGene00008035		C39E9.12	3.3352567	0.625	0.187391873	0.463367002
WBGene00009937	lsl-1	F52F12.4	3.979425172	0.625	0.15705786	0.345525782
WBGene00013979		ZK507.3	2.69616005	0.625	0.231811164	0.593984595
WBGene00017774	nop-1	F25B5.2	2.938964682	0.625	0.212659922	0.495348337
WBGene00000886	cyn-10	B0252.4	4.572337482	0.6225	0.136144806	0.312483699
WBGene00001102	dsh-2	C27A2.6	3.425251834	0.6225	0.181738462	0.463367002
WBGene00009286		F31C3.4	3.281427313	0.6225	0.189704034	0.463367002
WBGene00011819	hal-2	T16H12.11	3.637583041	0.6225	0.171130114	0.345525782
WBGene00014250		ZK1307.9	3.322068378	0.6225	0.187383259	0.463367002
WBGene00019141	bath-5	F59H6.11	2.778882536	0.6225	0.224010908	0.495348337
WBGene00022751		ZK484.5	3.513654736	0.6225	0.177165956	0.414186921
A_12_P173493			2.946896056	0.62	0.210390862	0.495348337
WBGene00000275	bub-1	R06C7.8	5.61453365	0.62	0.110427693	0.312483699
WBGene00001867	him-8	T07G12.12	3.190529269	0.62	0.194325125	0.489403914
WBGene00001985	hop-1	C18E3.8	3.657920332	0.62	0.169495217	0.345525782
WBGene00006804	unc-71	Y37D8A.13	3.041989384	0.62	0.203813992	0.495348337
WBGene00015012	bath-20	B0047.1	3.056598988	0.62	0.202839824	0.495348337
WBGene00000498	chk-1	Y39H10A.7	2.861302889	0.6175	0.215810777	0.495348337
WBGene00012167		W01A8.5	3.875904085	0.6175	0.159317668	0.345525782
WBGene00018068		F35H10.2	2.873224246	0.6175	0.214915352	0.495348337
WBGene00003159	mcm-7	F32D1.10	3.37978682	0.615	0.181964139	0.463367002
WBGene00005007	spr-2	C27B7.1	4.081766998	0.615	0.150670041	0.345525782
WBGene00007762		C27B7.5	3.487495537	0.615	0.176344312	0.414186921
WBGene00007978		C36B1.11	3.472612924	0.615	0.177100072	0.414186921
WBGene00010583		K05C4.7	3.549418741	0.615	0.173267807	0.414186921
WBGene00010608		K07A1.1	3.353359662	0.615	0.183398162	0.463367002
WBGene00015310	fan-1	C01G5.8	3.113275125	0.6125	0.196738154	0.489403914
WBGene00003158	mcm-6	ZK632.1	3.246276624	0.61	0.187907585	0.463367002
WBGene00010617		K07A1.13	2.94527707	0.61	0.207111245	0.495348337
WBGene00015745		C13F10.6	3.046209169	0.61	0.200248888	0.495348337
WBGene00044763		ZK688.12	3.443648158	0.61	0.177137725	0.414186921
WBGene00009346		F32H2.10	2.933730856	0.6075	0.20707421	0.495348337
A_12_P173476			2.703236263	0.605	0.223805817	0.593984595
WBGene00002034	htp-3	F57C9.5	3.612063132	0.605	0.167494304	0.414186921
WBGene00004737	scc-1	F10G7.4	6.160126328	0.605	0.098212272	0.300971142
WBGene00010056	smc-6	F54D5.14	2.851040644	0.605	0.212203218	0.495348337
WBGene00012313		W06G6.11	5.57147926	0.605	0.108588756	0.312483699
WBGene00018120		F36H12.4	2.689915844	0.605	0.2249141	0.593984595
WBGene00022298		Y76B12C.4	2.893598486	0.605	0.209082222	0.495348337
WBGene00011601	zim-3	T07G12.11	3.18167789	0.6025	0.189365492	0.489403914
WBGene00001016	dna-2	F43G6.1	3.725263171	0.6	0.161062446	0.345525782
WBGene00001830	hcp-2	T06E4.1	2.759195421	0.6	0.217454695	0.495348337
WBGene00008648		F10D11.2	2.880581985	0.6	0.208291242	0.495348337
WBGene00011886		T21B10.4	2.834748119	0.6	0.211659017	0.495348337
WBGene00021345		Y37B11A.3	3.1609915	0.6	0.189813861	0.489403914
WBGene00022610		ZC416.6	2.752917646	0.6	0.217950581	0.495348337

B. CEH-23 repressed genes

Gene ID	public name	seq name	Score(d)	Numerator(r)	Denominator(s+s0)	q-value(%)
A_12_P161556			-3.07119558	-0.6	0.195363657	0.50676114
WBGene00001594	glc-4	C27H5.8	-3.331306401	-0.6	0.180109521	0.489403914
WBGene00003778	nnt-1	C15H9.1	-3.423892775	-0.6	0.175239133	0.463367002
WBGene00006761	unc-24	F57H12.2	-3.463627509	-0.6	0.173228789	0.463367002
WBGene00010290		F58H1.7	-3.202435319	-0.6	0.187357414	0.489403914

WBGene00011924		T22C8.3	-3.521518276	-0.6	0.170381055	0.463367002
WBGene00012124		T28D6.4	-3.792657926	-0.6	0.1582004	0.439880899
WBGene00012777	lact-8	Y42A5A.2	-3.356043623	-0.6	0.178781943	0.463367002
WBGene00016625	aff-1	C44B7.3	-3.016069319	-0.6	0.19893442	0.50676114
WBGene00003569	ncx-4	F35C12.2	-2.847042117	-0.6025	0.211623143	0.50676114
WBGene00006759	unc-22	ZK617.1	-4.001763009	-0.6025	0.150558641	0.439880899
WBGene00010345		F59F5.7	-3.94037986	-0.6025	0.15290404	0.439880899
WBGene00011956		T23F11.4	-2.942704333	-0.6025	0.204743641	0.50676114
WBGene00012891	sorb-1	Y45F10D.13	-3.286639134	-0.6025	0.183317966	0.489403914
WBGene00016863		C52A10.2	-3.781752569	-0.6025	0.159317668	0.439880899
WBGene00018133		F37A4.3	-3.285361522	-0.6025	0.183389254	0.489403914
WBGene00019904	twk-49	R05G9.2	-4.413834195	-0.6025	0.136502635	0.439880899
A_12_P159590			-4.066723985	-0.605	0.148768395	0.439880899
A_12_P159591			-5.051546983	-0.605	0.119765292	0.439880899
WBGene00000557	cnc-3	R09B5.8	-5.283698613	-0.605	0.114503124	0.439880899
WBGene00001721	grl-12	F28A12.2	-3.11281713	-0.605	0.194357707	0.50676114
WBGene00006831	unc-104	C52E12.2	-3.479207876	-0.605	0.173890156	0.463367002
WBGene00007801	trpa-1	C29E6.2	-5.581670381	-0.605	0.108390492	0.439880899
WBGene00007988	best-8	C37A5.1	-4.253620007	-0.605	0.142231793	0.439880899
WBGene00009395	clec-64	F35C5.7	-3.709146406	-0.605	0.163110305	0.439880899
WBGene00020229		T05A8.3	-4.835214048	-0.605	0.125123726	0.439880899
WBGene00044079	tag-241	C34E11.3	-5.655500943	-0.605	0.106975493	0.439880899
A_12_P130711			-3.230172736	-0.6075	0.188070438	0.489403914
WBGene00000914	daf-19	F33H1.1	-3.990761383	-0.6075	0.152226591	0.439880899
WBGene00001454	flp-11	K02G10.4	-3.848089871	-0.6075	0.157870533	0.439880899
WBGene00004921	snt-1	F31E8.2	-3.32558914	-0.6075	0.1826744	0.489403914
WBGene00007353		C06A1.2	-3.481389727	-0.6075	0.17449928	0.463367002
WBGene00008783		F14B6.2	-3.965592219	-0.6075	0.153192756	0.439880899
WBGene00012099	acc-4	T27E9.9	-4.364615181	-0.6075	0.139187529	0.439880899
WBGene00013683	zoo-1	Y105E8A.26	-2.952406852	-0.6075	0.205764324	0.50676114
WBGene00018579	acs-16	F47G6.2	-4.99628165	-0.6075	0.121590423	0.439880899
WBGene00019074	cpd-1	F59A3.1	-3.132121277	-0.6075	0.193958007	0.50676114
WBGene00020657	lgc-53	T21F2.1	-4.4011355	-0.6075	0.13803256	0.439880899
WBGene00022499	set-30	ZC8.3	-4.13511598	-0.6075	0.146912445	0.439880899
WBGene00022518	zfh-2	ZC123.3	-3.33609127	-0.6075	0.182099334	0.489403914
A_12_P130714			-3.500945435	-0.61	0.174238648	0.463367002
WBGene00000006	aat-5	C55C2.5	-2.773759876	-0.61	0.219918099	0.593984595
WBGene00000723	col-150	B0024.2	-3.919785023	-0.61	0.155620779	0.439880899
WBGene00001116	dyc-1	C33G3.1	-3.45543014	-0.61	0.176533738	0.463367002
WBGene00009958	madd-4	F53B6.2	-4.839737902	-0.61	0.126039883	0.439880899
WBGene00014051	spv-1	ZK669.1	-3.919785023	-0.61	0.155620779	0.439880899
WBGene00021156		Y4C6B.2	-2.737599337	-0.61	0.222822965	0.593984595
WBGene00022283	lgc-27	Y74E4A.1	-3.919785023	-0.61	0.155620779	0.439880899
WBGene00044701		AH9.6	-3.362330787	-0.61	0.181421769	0.463367002
WBGene00050974		Y6G8.9	-3.297463013	-0.61	0.184990703	0.489403914
WBGene00004740	scd-2	T10H9.2	-3.775425916	-0.6125	0.162233352	0.439880899
WBGene00009058		F22E12.1	-2.994191451	-0.6125	0.204562738	0.50676114
WBGene00009889		F49E11.2	-3.625161137	-0.6125	0.168958007	0.439880899
WBGene00013866	cbs-1	ZC373.1	-4.929691137	-0.6125	0.124247135	0.439880899
WBGene00013898		ZC443.3	-3.812537266	-0.6125	0.160654167	0.439880899
WBGene00015824		C16B8.4	-4.340586064	-0.6125	0.141109977	0.439880899
WBGene00016916		C54D2.2	-2.813557114	-0.6125	0.217695954	0.50676114
WBGene00018206	ugt-61	F39G3.1	-4.624040912	-0.6125	0.132459901	0.439880899
WBGene00020012		R11G1.6	-3.751412562	-0.6125	0.163271832	0.439880899
WBGene00020846		T27A10.6	-2.873362169	-0.6125	0.213164914	0.50676114
WBGene00021917		Y55D5A.3	-4.810071477	-0.6125	0.127336985	0.439880899
WBGene00006367	sym-2	ZK1067.6	-3.348162117	-0.615	0.183682862	0.463367002
WBGene00008987		F20G2.3	-3.078372002	-0.615	0.199780923	0.50676114
WBGene00012226		W03G11.4	-3.466723554	-0.615	0.177400935	0.463367002
WBGene00020880		T28A11.18	-2.805173135	-0.615	0.219237805	0.50676114
WBGene00000481	cha-1	ZC416.8	-3.918230807	-0.6175	0.157596637	0.439880899
WBGene00004830	slo-1	Y51A2D.19	-5.047364748	-0.6175	0.122341069	0.439880899
WBGene00009018		F21D9.4	-2.709175692	-0.6175	0.227929108	0.593984595
WBGene00011326		T01D3.1	-4.418345757	-0.6175	0.139758189	0.439880899
WBGene00012186	mlt-11	W01F3.3	-2.868733698	-0.6175	0.215251768	0.50676114
A_12_P159568			-3.32020489	-0.62	0.186735464	0.489403914
WBGene00002176	jip-1	F56D12.4	-3.812219251	-0.62	0.162634927	0.439880899
WBGene00003995	pgp-1	K08E7.9	-3.330112313	-0.62	0.186179907	0.489403914
WBGene00006844	unc-120	D1081.2	-3.792106504	-0.62	0.163497518	0.439880899
WBGene00016817		C50E3.6	-3.590434658	-0.62	0.172681043	0.439880899

WBGene00019188		H11E01.3	-2.701501887	-0.62	0.229501968	0.593984595
WBGene00019268		H41C03.1	-3.804127013	-0.62	0.162980888	0.439880899
WBGene00045410		E01G4.7	-3.050177247	-0.62	0.203266876	0.50676114
WBGene00001500	ftn-1	C54F6.14	-2.751783313	-0.6225	0.22621694	0.593984595
WBGene00004212	ptl-1	F42G9.9	-3.824790989	-0.6225	0.162753991	0.439880899
WBGene00005644	srp-3	Y32G9A.4	-3.398384737	-0.6225	0.183175258	0.463367002
WBGene00020678		T22B11.4	-3.346827239	-0.6225	0.185997052	0.463367002
WBGene00002087	ins-4	ZK75.1	-3.432022903	-0.625	0.182108342	0.463367002
WBGene00008999		F21A10.2	-3.920496794	-0.625	0.159418572	0.439880899
WBGene00011422		T04B2.3	-4.460697844	-0.625	0.140112606	0.439880899
WBGene00016539	trim-9	C39F7.2	-5.755660392	-0.625	0.108588756	0.439880899
WBGene00000170	aqp-2	C01G6.1	-4.295605947	-0.6275	0.146079507	0.439880899
WBGene00000526	clc-5	C01C10.4	-5.186702008	-0.6275	0.120982466	0.439880899
WBGene00001805	gur-4	K09E4.5	-4.746348417	-0.6275	0.132206898	0.439880899
WBGene00003746	nlp-8	D2005.2	-3.531398952	-0.6275	0.17769162	0.463367002
WBGene00010321		F59B10.5	-6.20125978	-0.6275	0.101189117	0
WBGene00012252		W04E12.7	-4.358910032	-0.6275	0.143958007	0.439880899
WBGene00013882		ZC410.5	-4.317359357	-0.6275	0.145343472	0.439880899
WBGene00014206	nit-1	ZK1058.6	-3.104473949	-0.6275	0.202127642	0.50676114
WBGene00014989	best-23	ZK675.3	-2.758051317	-0.6275	0.227515709	0.593984595
WBGene00019230	ttll-11	H23L24.3	-4.209743718	-0.6275	0.149058955	0.439880899
A_12_P130061			-3.118145673	-0.63	0.202043158	0.50676114
A_12_P167754			-2.965260004	-0.63	0.21246029	0.50676114
WBGene00001053	dop-2	K09G1.4	-3.568722931	-0.63	0.176533738	0.439880899
WBGene00001374	exp-2	F12F3.1	-3.615730531	-0.63	0.174238648	0.439880899
WBGene00001759	gst-11	R11G1.3	-5.229770408	-0.63	0.120464179	0.439880899
WBGene00006575	tir-1	F13B10.1	-2.70629012	-0.63	0.232791006	0.593984595
WBGene00008358	nspc-18	D1025.7	-2.843747825	-0.63	0.221538631	0.50676114
WBGene00018115		F36H9.7	-3.304972178	-0.63	0.190621877	0.489403914
WBGene00019520		K08B12.1	-2.839619514	-0.63	0.221860709	0.50676114
WBGene00020051		R13A5.9	-5.557409771	-0.63	0.113362164	0.439880899
WBGene00002991	lin-2	F17E5.1	-3.896616241	-0.6325	0.162320321	0.439880899
WBGene00003001	lin-12	R107.8	-3.564050121	-0.6325	0.17746664	0.439880899
WBGene00003751	nlp-13	E03D2.1	-3.194263308	-0.6325	0.198011228	0.489403914
WBGene00003909	pag-3	F45B8.4	-4.593905394	-0.6325	0.137682417	0.439880899
WBGene00007900		C33D9.6	-6.110467775	-0.6325	0.103510897	0
WBGene00011019		R05A10.2	-4.027473124	-0.6325	0.157046361	0.439880899
WBGene00011223		R10H10.4	-3.324681696	-0.6325	0.190243776	0.489403914
WBGene00016407		C34D10.2	-2.722677462	-0.6325	0.23230809	0.593984595
WBGene00017557	nep-11	F18A12.8	-2.756351345	-0.6325	0.229470021	0.593984595
WBGene00020930	hlh-30	W02C12.3	-3.494218257	-0.6325	0.181013306	0.463367002
WBGene00017866	stn-2	F27D9.8	-5.745444056	-0.635	0.110522354	0.439880899
WBGene00044330	alr-1	R08B4.2	-2.930212957	-0.635	0.216707799	0.50676114
A_12_P173491			-3.100910975	-0.6375	0.205584748	0.50676114
WBGene00012540		Y37A1B.7	-3.395882648	-0.6375	0.187727335	0.463367002
WBGene00000631	col-54	F33D11.3	-3.110693702	-0.64	0.20574189	0.50676114
WBGene00001777	gst-29	Y53F4B.32	-2.980545663	-0.64	0.214725783	0.50676114
WBGene00003759	nlp-21	Y47D3B.2	-5.038933085	-0.64	0.127011014	0.439880899
WBGene00017571	jmjd-3.1	F18E9.5	-5.141347255	-0.64	0.124480991	0.439880899
WBGene00044467		R05C11.4	-4.357488522	-0.64	0.146873594	0.439880899
WBGene00003648	nhr-58	R11G11.2	-6.906124016	-0.6425	0.093033371	0
WBGene00006683	twk-31	Y47D3B.5	-3.451769865	-0.6425	0.186136395	0.463367002
WBGene00011798		T16G1.4	-3.127054775	-0.6425	0.205464901	0.50676114
WBGene00015436		C04E12.6	-3.691775566	-0.6425	0.174035498	0.439880899
WBGene00018803	fbxa-24	F54D10.2	-3.708341325	-0.6425	0.173258054	0.439880899
WBGene00019185		H10E21.5	-2.878384136	-0.6425	0.223215516	0.50676114
WBGene00003754	nlp-16	T13A10.5	-3.202641332	-0.645	0.201396264	0.489403914
WBGene00005936	srx-45	K01B6.2	-3.516938182	-0.645	0.183398162	0.463367002
WBGene00008549	din-1	F07A11.6	-3.248962577	-0.645	0.198524909	0.489403914
WBGene00010632		K07C5.9	-6.342476151	-0.645	0.101695298	0
WBGene00016355	lact-5	C33F10.7	-3.138192459	-0.645	0.205532327	0.50676114
WBGene00018837		F54G2.1	-4.048230178	-0.645	0.159328885	0.439880899
A_12_P161554			-5.564926816	-0.6475	0.116353731	0.439880899
WBGene00000403	casy-1	B0034.3	-3.510371415	-0.6475	0.184453416	0.463367002
WBGene00004202	pry-1	C37A5.9	-2.755324487	-0.6475	0.234999545	0.593984595
WBGene00007104		B0024.15	-4.001920597	-0.6475	0.161797313	0.439880899
WBGene00009932		F52D10.6	-3.687908486	-0.6475	0.175573771	0.439880899
WBGene00010446		K01A6.4	-4.474556619	-0.6475	0.144707075	0.439880899
WBGene00010607		K06G5.3	-3.934867212	-0.6475	0.164554473	0.439880899
WBGene00015067		B0238.1	-3.470855044	-0.6475	0.186553455	0.463367002

WBGene00017039	trk-1	D1073.1	-3.284778168	-0.6475	0.197121378	0.489403914
WBGene00018738		F53B1.6	-3.499677201	-0.6475	0.185017064	0.463367002
WBGene00019407		K05F1.5	-3.416868135	-0.6475	0.189501021	0.463367002
A_12_P178849			-4.397808061	-0.65	0.147800902	0.439880899
WBGene00000246	bcc-1	M7.3	-5.157097764	-0.65	0.126039883	0.439880899
WBGene00000431	ceh-6	K02B12.1	-3.549032992	-0.65	0.183148481	0.463367002
WBGene00002974	lev-1	F09E8.7	-3.436672339	-0.65	0.189136448	0.463367002
WBGene00008177		C48D1.1	-3.079343388	-0.65	0.211083961	0.50676114
WBGene00016028	flp-24	C24A1.1	-2.709965739	-0.65	0.239855431	0.593984595
WBGene00016172		C27H5.2	-6.627190134	-0.65	0.098080783	0
WBGene00020126	flp-26	R173.4	-3.273628149	-0.65	0.198556455	0.489403914
WBGene00020673		T22B7.4	-5.016529043	-0.65	0.129571661	0.439880899
WBGene00020741		T23F4.3	-3.096315393	-0.65	0.209926935	0.50676114
A_12_P159567			-3.13053668	-0.6525	0.208430715	0.50676114
WBGene00012818		Y43F8B.7	-3.065098093	-0.6525	0.212880626	0.50676114
WBGene00015464		C05C8.7	-2.929472609	-0.6525	0.222736338	0.50676114
WBGene00017660		F21C10.11	-3.228158171	-0.6525	0.202127642	0.489403914
WBGene00020245		T05B11.1	-2.971144508	-0.6525	0.219612341	0.50676114
WBGene00021268		Y23B4A.2	-3.686121898	-0.6525	0.177015307	0.439880899
A_12_P130713			-3.106850439	-0.655	0.210824439	0.50676114
A_12_P161553			-4.949023866	-0.655	0.132349331	0.439880899
WBGene00000443	ceh-20	F31E3.1	-3.231583981	-0.655	0.202686981	0.489403914
WBGene00000482	chd-3	T14G8.1	-3.955812194	-0.655	0.165579145	0.439880899
WBGene00001172	egl-3	C51E3.7	-4.601603377	-0.655	0.14234169	0.439880899
WBGene00007452		C08F11.3	-2.808551367	-0.655	0.233216315	0.50676114
WBGene00011971	lron-9	T23G11.6	-5.52744217	-0.655	0.118499657	0.439880899
WBGene00012289		W05H12.1	-4.261998587	-0.655	0.153683768	0.439880899
WBGene00018074		F35H12.1	-3.843419866	-0.655	0.170421141	0.439880899
WBGene00021059	atat-2	W06B11.1	-3.065297978	-0.655	0.213682325	0.50676114
WBGene00021163	nhr-275	Y5H2A.2	-3.441581337	-0.655	0.190319489	0.463367002
A_12_P171548			-4.876990726	-0.6575	0.134816742	0.439880899
WBGene00003029	lin-44	E01A2.3	-4.065923846	-0.6575	0.161709866	0.439880899
WBGene00003168	mec-4	T01C8.7	-6.101250928	-0.6575	0.107764786	0
WBGene00003969	pef-1	F23H11.8	-3.490948569	-0.6575	0.188344224	0.463367002
WBGene00016974	lron-15	C56E6.6	-3.406035716	-0.6575	0.193039667	0.463367002
WBGene00003750	nlp-12	M01D7.5	-2.913108083	-0.66	0.22656214	0.50676114
WBGene00007053	chd-7	T04D1.4	-3.218221782	-0.66	0.205082199	0.489403914
WBGene00013418		Y65A5A.1	-3.707082624	-0.66	0.178037575	0.439880899
WBGene00016659		C45B2.2	-3.378315138	-0.66	0.195363657	0.463367002
WBGene00020887		T28B4.4	-3.691647985	-0.66	0.178781943	0.439880899
WBGene00003706	nhr-116	F09C6.9	-5.476000128	-0.6625	0.120982466	0.439880899
WBGene00004777	ser-2	C02D4.2	-3.575324132	-0.6625	0.185297885	0.439880899
WBGene00011763		T14B1.1	-3.151178556	-0.6625	0.210238801	0.489403914
WBGene00015231	cutl-14	B0511.5	-4.698639398	-0.6625	0.140998264	0.439880899
WBGene00000952	del-1	E02H4.1	-5.689939293	-0.665	0.116872952	0.439880899
WBGene00007073	ugt-2	AC3.8	-4.327067268	-0.665	0.153683768	0.439880899
WBGene00007316	nlp-41	C04H5.8	-3.41353019	-0.665	0.194812983	0.463367002
WBGene00007408		C07B5.3	-3.183237266	-0.665	0.208906828	0.489403914
WBGene00007531		C11H1.7	-2.892752601	-0.665	0.229884851	0.50676114
WBGene00010722		K09E4.4	-4.862710836	-0.665	0.136754996	0.439880899
WBGene00020045	glb-23	R13A1.8	-3.654566236	-0.665	0.181964139	0.439880899
WBGene00022076	daao-1	Y69A2AR.5	-4.488430918	-0.665	0.14815868	0.439880899
A_12_P178594			-3.406163098	-0.6675	0.195968302	0.463367002
WBGene00001788	gst-40	F56B3.10	-3.716122537	-0.6675	0.17962271	0.439880899
WBGene00007214		C01A2.2	-3.16365257	-0.6675	0.210990298	0.489403914
WBGene00009779		F46C5.2	-3.441466589	-0.6675	0.193958007	0.463367002
WBGene00015935		C17H12.10	-7.249494633	-0.6675	0.092075384	0
WBGene00016294		C31H2.4	-3.997296735	-0.6675	0.166987853	0.439880899
WBGene00022696		ZK328.7	-3.48534254	-0.6675	0.191516327	0.463367002
A_12_P130091			-3.542416103	-0.67	0.189136448	0.463367002
A_12_P172497			-3.236829125	-0.67	0.2069927	0.489403914
WBGene00000932	dao-6	T05G5.12	-2.80860087	-0.67	0.238552942	0.50676114
WBGene00003089	ltd-1	K02C4.4	-4.807227212	-0.67	0.139373483	0.439880899
WBGene00010311		F59B2.9	-4.651964266	-0.67	0.144025182	0.439880899
WBGene00014099		ZK836.3	-3.022837336	-0.67	0.221646065	0.50676114
WBGene00015276		C01B7.5	-3.580007357	-0.67	0.187150453	0.439880899
WBGene00017980		F32B5.7	-3.594641559	-0.67	0.186388542	0.439880899
WBGene00019104	asp-7	F59D6.2	-5.202897318	-0.67	0.128774404	0.439880899
WBGene00020331		T07H6.4	-3.638456272	-0.67	0.18414403	0.439880899
WBGene00021775			-4.295041204	-0.67	0.155993847	0.439880899

WBGene00022100		Y69A2AR.31	-2.831137773	-0.67	0.236653972	0.50676114
WBGene00011817		T16H12.9	-4.336094137	-0.6725	0.155093496	0.439880899
WBGene00012648		Y39A1A.9	-2.774318304	-0.6725	0.24240189	0.593984595
WBGene00020379		T09B4.5	-3.490894276	-0.6725	0.192644047	0.463367002
WBGene00000220	atf-2	K08F8.2	-3.509744539	-0.675	0.19232169	0.463367002
WBGene00001461	flp-18	Y48D7A.2	-3.814651266	-0.675	0.176949334	0.439880899
WBGene00009475		F36F2.1	-2.835613222	-0.675	0.238043748	0.50676114
WBGene00011382	dop-5	T02E9.3	-3.299599532	-0.675	0.20457028	0.489403914
WBGene00011760		T13H5.6	-3.325246077	-0.675	0.202992496	0.489403914
WBGene00017936		F30B5.7	-2.810538075	-0.675	0.240167534	0.50676114
WBGene00020946		W02D7.11	-3.188659371	-0.675	0.21168771	0.489403914
A_12_P157859			-2.911981554	-0.6775	0.232659441	0.50676114
A_12_P159569			-3.107424729	-0.6775	0.218026198	0.50676114
WBGene00006681	twk-29	F46A9.3	-5.016284583	-0.6775	0.13506012	0.439880899
WBGene00008307	ncs-5	C54E10.2	-3.306098752	-0.6775	0.204924308	0.489403914
WBGene00009387	glb-15	F35B12.8	-3.796830873	-0.6775	0.178438288	0.439880899
WBGene00015293		C01C4.3	-6.422861986	-0.6775	0.105482572	0
WBGene00044663		F26G1.10	-2.828944774	-0.6775	0.239488592	0.50676114
WBGene00002222	klp-11	F20C5.2	-3.130268252	-0.68	0.217233778	0.50676114
WBGene00004364	ric-4	Y22F5A.3	-3.43017428	-0.68	0.198240656	0.463367002
WBGene00006580	tlp-1	T23G4.1	-5.456393858	-0.68	0.124624435	0.439880899
WBGene00009038		F22B3.7	-4.150380125	-0.68	0.163840415	0.439880899
WBGene00016196		C28H8.5	-4.141742406	-0.68	0.164182108	0.439880899
WBGene00010759	cysl-2	K10H10.2	-4.936171867	-0.6825	0.13826504	0.439880899
WBGene00017991	clec-180	F32E10.3	-3.605432613	-0.6825	0.189297672	0.439880899
WBGene00003006	lin-17	Y71F9B.5	-4.270587466	-0.685	0.160399478	0.439880899
WBGene00011869	dod-6	T20G5.7	-2.954991414	-0.685	0.231811164	0.50676114
WBGene00077439		F55B12.10	-4.479580951	-0.685	0.152916089	0.439880899
A_12_P161555			-5.495341365	-0.6875	0.125105968	0.439880899
WBGene00001710	grl-1	C24G6.7	-5.552733369	-0.6875	0.123812896	0.439880899
WBGene00008437			-3.593526242	-0.6875	0.19131626	0.439880899
WBGene00009800	rrc-1	F47A4.3	-5.61953565	-0.6875	0.122341069	0.439880899
WBGene00011070		R06F6.7	-2.978195376	-0.6875	0.230844492	0.50676114
WBGene00016456		C35E7.4	-3.793494118	-0.6875	0.181231334	0.439880899
WBGene00022825		ZK816.1	-3.342173022	-0.6875	0.205704491	0.489403914
WBGene00045409			-5.18039306	-0.6875	0.132711937	0.439880899
WBGene00000740	col-167	T10E10.2	-3.690961915	-0.69	0.186943137	0.439880899
WBGene00000741	col-168	T10E10.1	-3.704708085	-0.69	0.186249492	0.439880899
WBGene00001447	flp-4	C18D1.3	-3.38644807	-0.69	0.203753309	0.463367002
WBGene00007750	syg-2	C26G2.1	-4.369093643	-0.69	0.157927492	0.439880899
WBGene00008897		F16H6.7	-5.272065295	-0.69	0.1308785	0.439880899
WBGene00016423	tag-275	C34H3.1	-3.708867566	-0.69	0.186040614	0.439880899
WBGene00002069	ikb-1	C04F12.3	-4.230266807	-0.6925	0.163701258	0.439880899
WBGene00004088	ppk-2	Y48G9A.8	-3.51420118	-0.6925	0.197057586	0.463367002
WBGene00004719	sad-1	F15A2.6	-3.285735868	-0.6925	0.210759485	0.489403914
WBGene00022591	cuti-1	ZC328.1	-2.96245426	-0.6925	0.233758883	0.50676114
WBGene00023500		C11H1.9	-3.602133419	-0.6925	0.192247182	0.439880899
WBGene00023416	isl-1	R10H1.4	-2.95939152	-0.6975	0.235690342	0.50676114
WBGene00045102		F52B11.8	-6.34510005	-0.6975	0.109927345	0
WBGene00000068	acy-1	F17C8.1	-5.125315676	-0.7	0.136576953	0.439880899
WBGene00000288	cal-4	T07G12.1	-3.256564768	-0.7	0.214950431	0.489403914
WBGene00001459	flp-16	F15D4.8	-2.793042107	-0.7	0.250622788	0.50676114
WBGene00007397		C07A4.2	-4.028226582	-0.7	0.17377374	0.439880899
WBGene00010078			-2.791530651	-0.7	0.250758486	0.50676114
WBGene00013312	hhat-2	Y57G11C.17	-2.854770064	-0.7	0.245203636	0.50676114
WBGene00016504		C37C3.12	-2.74885837	-0.7	0.25465117	0.593984595
WBGene00017255		F08D12.12	-5.01426555	-0.7	0.139601701	0.439880899
WBGene00044350		Y73B6BL.44	-4.341396106	-0.7	0.161238455	0.439880899
A_12_P162823			-4.761693892	-0.7025	0.147531533	0.439880899
WBGene00006467	magu-2	C01B7.4	-3.513193792	-0.7025	0.199960504	0.463367002
WBGene00013962		ZK287.1	-5.253882565	-0.7025	0.13371064	0.439880899
WBGene00019753		M03E7.1	-2.885888302	-0.705	0.244292199	0.50676114
WBGene00045411		C25F9.11	-2.93077273	-0.705	0.240550894	0.50676114
WBGene00001760	gst-12	F37B1.2	-2.772833151	-0.7075	0.255154191	0.593984595
WBGene00009848		F48F5.6	-3.227189784	-0.7075	0.219230987	0.489403914
WBGene00013273	41338	Y57A10B.1	-4.683798009	-0.7075	0.151052628	0.439880899
WBGene00020497		T14A8.2	-3.963303874	-0.7075	0.178512681	0.439880899
A_12_P159566			-3.462026463	-0.71	0.205082199	0.463367002
WBGene00004004	pgp-10	C54D1.1	-2.883525359	-0.71	0.246226376	0.50676114
WBGene00011673	cyp-13A6	T10B9.3	-5.849292711	-0.71	0.121382197	0

WBGene00016671		C45G7.4	-2.836017192	-0.71	0.250351092	0.50676114
WBGene00017193		F07C3.2	-3.457975507	-0.71	0.205322449	0.463367002
WBGene00017314	lgc-39	F09G2.5	-2.707371203	-0.71	0.262247009	0.593984595
WBGene00001064	dpy-2	T14B4.6	-3.05126582	-0.7125	0.233509646	0.50676114
WBGene00001091	drh-2	C01B10.1	-3.829271072	-0.7125	0.186066744	0.439880899
WBGene00004944	sol-1	C15A11.3	-3.310077344	-0.7125	0.215251768	0.489403914
WBGene00011498		T05G5.1	-3.968275035	-0.7125	0.179549047	0.439880899
WBGene00044728		Y53F4B.45	-4.108664941	-0.7125	0.173413995	0.439880899
A_12_P159565			-4.951513494	-0.715	0.144400293	0.439880899
WBGene00006444	shn-1	C33B4.3	-2.844946565	-0.715	0.251322822	0.50676114
WBGene00008760		F13E9.11	-5.423178962	-0.715	0.131841491	0.439880899
WBGene00016481		C36C5.12	-3.311269055	-0.715	0.215929297	0.489403914
WBGene00020317	pdf-1	T07E3.6	-4.102657086	-0.715	0.1742773	0.439880899
WBGene00021948	kvs-5	Y55F3C.3	-3.26292301	-0.715	0.219128676	0.489403914
WBGene00000969	dhs-5	F56D1.5	-6.02351491	-0.7175	0.119116498	0
WBGene00004122	pqn-35	F35D11.2	-2.774433266	-0.7175	0.258611374	0.593984595
WBGene00006650	tts-1	F09E10.11	-4.399154402	-0.7175	0.163099527	0.439880899
WBGene00006982	zig-5	Y48A6A.1	-3.48699904	-0.7175	0.205764324	0.463367002
WBGene00008027	scl-5	C39E9.2	-4.324780047	-0.7175	0.165904391	0.439880899
WBGene00013891		ZC434.3	-2.976895035	-0.7175	0.241022942	0.50676114
WBGene00018472	nep-16	F45E4.7	-3.308476782	-0.7175	0.216867171	0.489403914
WBGene00019967	cyp-33C8	R08F11.3	-4.380691787	-0.7175	0.163786917	0.439880899
WBGene00011004		R04B5.6	-4.101509444	-0.72	0.175545128	0.439880899
WBGene00013458		Y67A10A.9	-3.123359656	-0.72	0.230521003	0.50676114
WBGene00015969	glb-6	C18C4.9	-2.837287906	-0.72	0.253763461	0.50676114
WBGene00019283		K01A2.9	-3.716374027	-0.72	0.193737227	0.439880899
WBGene00000990	dhs-27	C04F6.5	-3.641802768	-0.7225	0.198390755	0.439880899
WBGene00011107		R07E3.6	-3.560449531	-0.7225	0.202923814	0.439880899
WBGene00012317	ztf-6	W06H12.1	-2.907970905	-0.7225	0.248455031	0.50676114
WBGene00013848	npr-29	ZC84.4	-4.386118629	-0.7225	0.164724227	0.439880899
WBGene00015763		C14C6.12	-3.751726781	-0.7225	0.192577989	0.439880899
WBGene00022615		ZC449.5	-2.981752863	-0.7225	0.242307137	0.50676114
WBGene00023414		R01H2.7	-3.149261139	-0.7225	0.229418892	0.489403914
WBGene00007174	mboa-1	B0395.2	-4.161883311	-0.725	0.174199983	0.439880899
WBGene00009036		F22B3.5	-3.642689947	-0.725	0.199028743	0.439880899
WBGene00015393		C03G6.5	-2.82866739	-0.725	0.256304436	0.50676114
WBGene00022024		Y64H9A.2	-2.765242497	-0.725	0.262183154	0.593984595
WBGene00012131		T28F3.5	-7.646261881	-0.7275	0.095144531	0
WBGene00119203		T04A8.18	-5.306539666	-0.7275	0.13709499	0.439880899
WBGene00022416		Y102A11A.6	-3.856878536	-0.73	0.18927223	0.439880899
WBGene00000691	col-117	T28C6.4	-3.589293391	-0.7325	0.204079166	0.439880899
WBGene00003143	max-1	C34B4.1	-4.380031803	-0.7325	0.167236229	0.439880899
WBGene00006832	unc-105	C41C4.5	-3.555766561	-0.7325	0.2060034	0.463367002
WBGene00007530		C11H1.5	-5.611391352	-0.7325	0.130538035	0.439880899
WBGene00015356	tag-278	C02F12.7	-3.922115774	-0.7325	0.186761443	0.439880899
WBGene00015601	fbxa-166	C08E3.9	-3.969672904	-0.7325	0.184524019	0.439880899
A_12_P163053			-3.464594653	-0.735	0.21214603	0.463367002
A_12_P178909			-3.680181631	-0.735	0.199718404	0.439880899
WBGene00001490	frm-3	H05G16.1	-5.374575134	-0.735	0.136754996	0.439880899
WBGene00003755	nlp-17	Y45F10A.5	-3.156998781	-0.735	0.232816054	0.489403914
WBGene00020805		T25F10.1	-3.191596451	-0.735	0.230292273	0.489403914
WBGene00008563		F07H5.13	-3.652016174	-0.7375	0.201943246	0.439880899
WBGene00008971		F20C5.6	-4.42088165	-0.7375	0.166821928	0.439880899
WBGene00013084			-4.454346072	-0.7375	0.165568635	0.439880899
WBGene00013387		Y62F5A.9	-4.575530733	-0.7375	0.161183487	0.439880899
A_12_P108315			-2.9436284	-0.74	0.251390427	0.50676114
WBGene00006309	sul-2	D1014.1	-2.91151226	-0.74	0.25416345	0.50676114
WBGene00007736	igdb-2	C25G4.10	-4.93581549	-0.74	0.149924567	0.439880899
WBGene00016221		C29F9.6	-3.705797113	-0.74	0.199687133	0.439880899
WBGene00020626		T20F5.4	-3.572959069	-0.74	0.207111245	0.439880899
WBGene00001705	grd-16	Y69A2AL.1	-3.997999609	-0.7425	0.185717877	0.439880899
WBGene00009304	eva-1	F32A7.3	-3.876954062	-0.7425	0.191516327	0.439880899
WBGene00015883		C17B7.9	-3.983057203	-0.7425	0.186414596	0.439880899
WBGene00020250		T05C1.3	-3.434306701	-0.7425	0.216200842	0.463367002
WBGene00020650		T21D12.11	-4.95125436	-0.7425	0.149961999	0.439880899
WBGene00019426	cutl-16	K06A1.3	-4.023409594	-0.745	0.185166333	0.439880899
A_12_P177940			-4.56863736	-0.7475	0.163615525	0.439880899
WBGene00000037	ace-3	Y48B6A.8	-7.318675685	-0.7475	0.102135965	0
WBGene00003719	nhr-129	C50B6.14	-3.041342172	-0.7475	0.245779645	0.50676114
WBGene00017247		F08D12.3	-3.146688671	-0.7475	0.237551305	0.489403914

WBGene00009094		F23H12.5	-3.384593267	-0.75	0.221592357	0.463367002
WBGene00011343			-2.721108048	-0.75	0.275623013	0.593984595
A_12_P180954			-2.933513857	-0.7525	0.256518304	0.50676114
WBGene00006970	zag-1	F28F9.1	-8.2315731	-0.7525	0.091416305	0
WBGene00016484		C36C5.15	-3.158687764	-0.7525	0.238231841	0.489403914
WBGene00017009		D1007.13	-2.992078443	-0.7525	0.251497417	0.50676114
A_12_P180955			-2.701521994	-0.755	0.279472091	0.593984595
A_12_P180958			-2.778366955	-0.755	0.271742362	0.593984595
WBGene00002110	ins-27	ZC334.11	-2.933690677	-0.755	0.257355012	0.50676114
WBGene00004924	snt-4	T23H2.2	-4.167407566	-0.755	0.181167786	0.439880899
WBGene00007552		C13C12.2	-3.618107406	-0.755	0.208672633	0.439880899
WBGene00009326		F32D8.1	-4.294355696	-0.755	0.175812171	0.439880899
WBGene00011495		T05F1.11	-3.954462799	-0.755	0.190923531	0.439880899
WBGene00019198		H14E04.1	-5.28786676	-0.755	0.142779694	0.439880899
WBGene00019451	nstp-7	K06H6.3	-4.882497769	-0.755	0.154633967	0.439880899
WBGene00004006	pgp-12	F22E10.1	-4.210277468	-0.7575	0.179916883	0.439880899
WBGene00006370			-4.337160696	-0.7575	0.174653432	0.439880899
WBGene00006812	unc-80	F25C8.3	-3.707398621	-0.7575	0.204321164	0.439880899
WBGene00010705	cyp-14A1	K09A11.2	-3.672868552	-0.7575	0.206242066	0.439880899
WBGene00018786	hmbx-1	F54A5.1	-3.928085969	-0.7575	0.192842012	0.439880899
WBGene00020368	ast-1	T08H4.3	-4.236345913	-0.7575	0.178809761	0.439880899
WBGene00044630	bus-17	ZK678.8	-4.617679333	-0.7575	0.164043439	0.439880899
WBGene00008376		D1054.9	-3.890181068	-0.76	0.195363657	0.439880899
WBGene00045246		C29E4.15	-3.107752187	-0.76	0.244549743	0.50676114
WBGene00009653		F43D9.1	-3.403852102	-0.7625	0.224010908	0.463367002
WBGene00009802		F47B8.1	-3.157976859	-0.7625	0.241452054	0.489403914
WBGene00020501		T14B4.5	-3.157976859	-0.7625	0.241452054	0.489403914
WBGene00006363	syd-1	F35D2.5	-4.189218152	-0.765	0.182611641	0.439880899
WBGene00010674		K08E7.6	-5.95612616	-0.765	0.128439187	0
WBGene00011926	sptf-2	T22C8.5	-4.37600517	-0.765	0.174816978	0.439880899
WBGene00044212		Y68A4A.13	-2.975072168	-0.765	0.257136619	0.50676114
WBGene00003071	lrp-1	F29D11.1	-5.473871923	-0.7675	0.140211538	0.439880899
WBGene00008033		C39E9.10	-5.184274754	-0.7675	0.148043851	0.439880899
WBGene00008763		F13G3.3	-4.206422512	-0.7675	0.182459084	0.439880899
WBGene00007607		C15C8.5	-3.725279506	-0.77	0.2066959	0.439880899
WBGene00019968		R08F11.4	-5.488938922	-0.77	0.140282122	0.439880899
WBGene00044921		F53C11.9	-3.615480417	-0.77	0.21297308	0.439880899
WBGene00000743	col-170	T10E10.6	-3.76196247	-0.7725	0.205344951	0.439880899
WBGene00001772	gst-24	F37B1.1	-4.390328005	-0.7725	0.175954963	0.439880899
WBGene00013568		Y75B12B.3	-3.164094215	-0.7725	0.244145701	0.489403914
A_12_P103575			-3.039418516	-0.775	0.254982983	0.50676114
WBGene00001079	dpy-20	T22B3.1	-2.857093837	-0.775	0.271254654	0.50676114
WBGene00003179	mec-18	C52B9.9	-7.55147935	-0.775	0.102628897	0
WBGene00003749	nlp-11	ZK1320.10	-3.696367219	-0.775	0.209665316	0.439880899
WBGene00000979	dhs-16	C10F3.2	-2.868438467	-0.78	0.271924955	0.50676114
WBGene00006750	unc-10	T10A3.1	-3.765015178	-0.78	0.20717048	0.439880899
WBGene00008583	ugt-65	F08G5.5	-3.214266614	-0.78	0.242668109	0.489403914
WBGene00001069	dpy-7	F46C8.6	-2.961175733	-0.7825	0.264253145	0.50676114
WBGene00001626	gly-1	F44F4.6	-3.022736301	-0.7825	0.258871407	0.50676114
WBGene00008538	sqrd-1	F02H6.5	-4.387099859	-0.7825	0.178363845	0.439880899
WBGene00009865		F49B2.6	-4.429969521	-0.7825	0.176637784	0.439880899
WBGene00044640		B0432.14	-3.231269106	-0.7825	0.242164912	0.489403914
WBGene00001570	gei-13	F58A4.11	-3.965531924	-0.785	0.197955789	0.439880899
WBGene00003112	mab-21	F35G12.6	-3.542111662	-0.785	0.221619213	0.463367002
WBGene00010262		F58E10.7	-3.389326263	-0.785	0.231609452	0.463367002
WBGene00010784	twk-48	K11H3.7	-3.823813472	-0.785	0.205292441	0.439880899
WBGene00013899		ZC443.4	-3.300408128	-0.785	0.237849372	0.489403914
WBGene00001159	eff-1	C26D10.5	-3.806254469	-0.7875	0.206896309	0.439880899
WBGene00001824	hbl-1	F13D11.2	-3.66902938	-0.7875	0.214634422	0.439880899
WBGene00006602	tps-1	ZK54.2	-5.041141406	-0.7875	0.156214622	0.439880899
WBGene00001089	dre-1	K04A8.6	-3.032698409	-0.79	0.260494086	0.50676114
WBGene00005833	srw-86	C25F9.7	-2.966211832	-0.79	0.266332968	0.50676114
WBGene00012261	lpr-3	W04G3.8	-3.907679311	-0.79	0.202166027	0.439880899
WBGene00016463		C35E7.11	-6.012456932	-0.79	0.131393873	0
WBGene00017983		F32D1.3	-5.142024485	-0.79	0.153635986	0.439880899
WBGene00001448	flp-5	C03G5.7	-3.763305731	-0.7925	0.210586133	0.439880899
WBGene00015019		B0205.4	-3.113521993	-0.7925	0.254534897	0.50676114
WBGene00003102	mab-5	C08C3.3	-3.192065843	-0.795	0.249055013	0.489403914
WBGene00013073	hmit-1.1	Y51A2D.4	-4.537649926	-0.795	0.175200823	0.439880899
WBGene00001133	eat-2	Y48B6A.4	-2.919606663	-0.7975	0.273153233	0.50676114

WBGene00010790	sodh-1	K12G11.3	-4.13127526	-0.7975	0.193039667	0.439880899
A_12_P179607			-2.709483454	-0.8	0.295259231	0.593984595
WBGene00007818		C30F2.4	-6.534122418	-0.8	0.122434192	0
WBGene00011313		T01B7.8	-2.979633932	-0.8	0.268489357	0.50676114
WBGene00016483		C36C5.14	-3.220121131	-0.8	0.248437859	0.489403914
WBGene00000280	cah-2	D1022.8	-6.08759042	-0.8025	0.131825557	0
WBGene00006667	twk-12	F29F11.4	-4.225758967	-0.8025	0.189906714	0.439880899
WBGene00020902	jmjc-1	T28F2.4	-5.398426843	-0.8025	0.148654418	0.439880899
WBGene00007203	best-1	B0564.3	-4.157220819	-0.805	0.19363898	0.439880899
WBGene00018056		F35F10.6	-3.536508742	-0.805	0.227625621	0.463367002
WBGene00021976		Y58A7A.2	-3.009613025	-0.805	0.267476248	0.50676114
WBGene00001113	dur-1	F25H8.5	-6.484130107	-0.8075	0.124534824	0
WBGene00014046	clec-60	ZK666.6	-4.387897594	-0.8075	0.184028907	0.439880899
WBGene00001444	flp-1	F23B2.5	-4.162693534	-0.81	0.194585547	0.439880899
WBGene00001768	gst-20	Y48E1B.10	-4.085942893	-0.81	0.198240656	0.439880899
WBGene00012437	hum-9	Y11D7A.14	-4.675897137	-0.81	0.173228789	0.439880899
WBGene00016996		D1005.2	-3.786164553	-0.81	0.213936819	0.439880899
WBGene00020616		T20D4.10	-3.567811971	-0.81	0.227029901	0.439880899
WBGene00000398			-4.029551177	-0.8125	0.201635359	0.439880899
WBGene00001163	efn-2	C43F9.8	-3.379092886	-0.8125	0.240449146	0.463367002
WBGene00002182	kap-1	F08F8.3	-3.916996514	-0.8125	0.20742934	0.439880899
WBGene00022274		Y73E7A.8	-4.197551064	-0.8125	0.193565245	0.439880899
A_12_P180957			-3.018180138	-0.815	0.270030271	0.50676114
WBGene00001789	gst-41	R13D7.7	-3.020929563	-0.815	0.269784509	0.50676114
WBGene00017060		D2063.1	-4.952447657	-0.815	0.164565091	0.439880899
WBGene00001733	grl-24	F11E6.2	-3.053095246	-0.8175	0.267761054	0.50676114
WBGene00010589	cyp-33D1	K05D4.4	-2.713776654	-0.8175	0.301240708	0.593984595
WBGene00015757		C14C6.3	-4.666329126	-0.8175	0.175191243	0.439880899
WBGene00016106		C25F6.7	-3.522846408	-0.8175	0.232056668	0.463367002
WBGene00020083		R57.2	-2.721809321	-0.8175	0.300351679	0.593984595
WBGene00000992	dhs-29	F27D9.6	-4.948547985	-0.82	0.165705173	0.439880899
WBGene00011437		T04F3.2	-2.826591352	-0.82	0.290102069	0.50676114
WBGene00012398		Y6E2A.4	-3.500743444	-0.8225	0.234950094	0.463367002
WBGene00013957	sre-23	ZK265.5	-5.787852787	-0.8225	0.142107968	0
WBGene00014254	cyp-13A10	ZK1320.4	-3.317806682	-0.8225	0.247904739	0.489403914
WBGene00015298		C01F1.3	-4.549354925	-0.8225	0.180794863	0.439880899
WBGene00011880		T21B6.3	-2.830596452	-0.825	0.291458007	0.50676114
WBGene00012763		Y41E3.3	-6.69133404	-0.8275	0.123667417	0
WBGene00001432	fkb-7	B0511.1	-3.677857929	-0.83	0.22567484	0.439880899
WBGene00016628		C44B7.6	-3.559309051	-0.83	0.233191327	0.439880899
A_12_P165981			-3.112003897	-0.8325	0.267512518	0.50676114
WBGene00003681	nhr-91	Y15E3A.1	-2.944120003	-0.8325	0.282767006	0.50676114
WBGene00006956	wrt-10	ZK1290.8	-3.54853997	-0.8325	0.234603529	0.463367002
WBGene00022758		ZK488.6	-6.077666366	-0.8325	0.136976917	0
WBGene00011055	arrd-14	R06B9.4	-3.345327792	-0.835	0.249601848	0.489403914
WBGene00011307	mpst-7	R186.6	-3.526534845	-0.835	0.236776336	0.463367002
WBGene00012070		T26H5.8	-4.560031552	-0.835	0.183112768	0.439880899
WBGene00001071	dpy-9	T21D12.2	-2.801060993	-0.8375	0.298993846	0.50676114
WBGene00013104		Y51H4A.8	-4.241797645	-0.8375	0.197439876	0.439880899
WBGene00022156		Y71G12B.18	-6.206529316	-0.8375	0.134938539	0
WBGene00001059	dpf-6	F44B9.1	-3.970801598	-0.84	0.211544188	0.439880899
WBGene00001519	gar-3	Y40H4A.1	-7.829770657	-0.84	0.107282836	0
WBGene00004932	sod-3	C08A9.1	-5.755968839	-0.84	0.145935467	0.439880899
WBGene00008712		F11E6.9	-3.351650528	-0.84	0.250622788	0.463367002
WBGene00010127		F55G11.7	-4.600829736	-0.84	0.182575763	0.439880899
WBGene00019449		K06H6.1	-3.328917105	-0.84	0.25233431	0.489403914
WBGene00010528		K03D3.2	-3.425039869	-0.845	0.246712457	0.463367002
WBGene00006097	str-31	C54F6.10	-5.016039287	-0.8475	0.168958007	0.439880899
WBGene00006949	wrt-3	F38E11.7	-4.489277028	-0.85	0.189340064	0.439880899
WBGene00019450		K06H6.2	-5.034168466	-0.85	0.168846157	0.439880899
WBGene00018743		F53B3.5	-2.943019521	-0.8525	0.289668483	0.50676114
WBGene00018861	cutl-21	F55A4.10	-5.786499008	-0.8525	0.147325697	0
WBGene00015647		C09F5.1	-9.541595797	-0.8625	0.090393684	0
WBGene00002393	lpr-1	Y65B4BR.2	-3.297614354	-0.865	0.262310843	0.489403914
WBGene00016919		C54E4.4	-2.737854642	-0.865	0.315940805	0.593984595
WBGene00019597		K09H9.5	-4.153424885	-0.865	0.208261862	0.439880899
WBGene00010505		K02D3.2	-4.859598743	-0.8675	0.178512681	0.439880899
WBGene00015795		C15F1.2	-4.14315293	-0.87	0.209985008	0.439880899
WBGene00019368		K03H6.2	-4.483053168	-0.87	0.194064172	0.439880899
WBGene00004017	phg-1	F27E5.4	-2.969591659	-0.8725	0.293811439	0.50676114

WBGene00015268		BE0003N10.3	-4.443509661	-0.8725	0.196353798	0.439880899
WBGene00000905	daf-9	T13C5.1	-4.90135687	-0.8775	0.179032056	0.439880899
WBGene00011508		T05H10.3	-5.492431889	-0.8775	0.159765295	0.439880899
WBGene00044303	hst-3.2	F52B10.2	-4.71781671	-0.8775	0.185997052	0.439880899
WBGene00000702	col-128	F12F6.9	-3.544748064	-0.88	0.248254596	0.463367002
WBGene00000753	col-180	C44C10.1	-3.022174785	-0.8825	0.29200826	0.50676114
WBGene00011009	cyp-14A4	R04D3.1	-4.98332331	-0.8825	0.177090657	0.439880899
WBGene00018639	tbc-16	F49E10.1	-6.453845714	-0.8825	0.136740176	0
WBGene00020192		T03F1.11	-3.178348485	-0.8825	0.277659924	0.489403914
WBGene00001699	grd-10	F09D12.1	-3.81279362	-0.885	0.232113271	0.439880899
WBGene00019751		M03D4.4	-3.411206388	-0.885	0.259439008	0.463367002
WBGene00020703		T22F7.4	-3.445695961	-0.8875	0.257567705	0.463367002
WBGene00006965	xtr-1	F54F7.4	-2.916408897	-0.89	0.305169827	0.50676114
WBGene00009061		F22G12.1	-5.737249539	-0.8925	0.155562346	0.439880899
WBGene00013884		ZC412.3	-4.684655009	-0.8975	0.191582944	0.439880899
WBGene00020258		T05E7.1	-3.210569386	-0.8975	0.27954543	0.489403914
WBGene00006852	unc-129	C53D6.2	-4.157850353	-0.9	0.216458007	0.439880899
WBGene00004224	ptr-10	F55F8.1	-3.152895314	-0.9025	0.286244835	0.489403914
WBGene00008519	cyp-13B1	F02C12.5	-2.939159729	-0.9025	0.307060549	0.50676114
WBGene00009586		F40F9.10	-5.151513194	-0.9025	0.175191243	0.439880899
WBGene00019113		F59E11.7	-3.297202294	-0.9025	0.273716903	0.489403914
WBGene00011452	ugt-55	T04H1.7	-3.351476104	-0.905	0.270030271	0.463367002
WBGene00015760		C14C6.6	-5.581841358	-0.9075	0.162580758	0.439880899
WBGene00016422	noah-1	C34G6.6	-4.210520758	-0.9075	0.215531535	0.439880899
WBGene00002116	ins-33	W09C5.4	-4.235746786	-0.91	0.214838149	0.439880899
WBGene00008978	cutl-3	F20D1.8	-4.580170424	-0.91	0.198682563	0.439880899
WBGene00015593		C08E3.1	-3.906564605	-0.91	0.232941239	0.439880899
WBGene00000016	abf-5	T22H6.5	-3.862742232	-0.9125	0.23623114	0.439880899
WBGene00009803		F47B8.2	-4.128703403	-0.915	0.221619213	0.439880899
WBGene00011833		T19B10.5	-4.575725115	-0.915	0.199968306	0.439880899
WBGene00044512		W03F9.11	-3.714123571	-0.9175	0.247030015	0.439880899
WBGene00000742	col-169	T10E10.5	-4.439518305	-0.92	0.20722969	0.439880899
WBGene00018041		F35D2.3	-3.543460141	-0.92	0.259633229	0.463367002
WBGene00008477	clec-17	E03H4.10	-5.704341633	-0.925	0.162157188	0.439880899
WBGene00012255	lpr-6	W04G3.1	-4.9673843	-0.925	0.186214705	0.439880899
WBGene00020873		T28A11.6	-2.829418179	-0.925	0.326922336	0.50676114
WBGene00010835		M03B6.3	-3.767100932	-0.93	0.246874192	0.439880899
WBGene00044315		F41G3.20	-3.223217501	-0.93	0.288531568	0.489403914
WBGene00016180		C28C12.4	-4.206775768	-0.9325	0.2216662	0.439880899
WBGene00011095	gana-1	R07B7.11	-5.420769617	-0.935	0.172484733	0.439880899
WBGene00011676	cyp-13A2	T10B9.7	-3.073202127	-0.935	0.304242924	0.50676114
WBGene00002827	let-653	C29E6.1	-3.82090198	-0.9375	0.245360913	0.439880899
WBGene00005078	src-2	F49B2.5	-8.527023881	-0.94	0.110237759	0
WBGene00021478		Y39H10A.1	-6.632082377	-0.94	0.141735272	0
WBGene00003651	nhr-61	W01D2.2	-4.313609295	-0.945	0.219074083	0.439880899
WBGene00020156		T02B11.4	-5.273753848	-0.945	0.179189251	0.439880899
WBGene00044515		K10C9.9	-5.232989038	-0.945	0.180585129	0.439880899
WBGene00017133		EEED8.2	-5.439452853	-0.9475	0.174190314	0.439880899
WBGene00021529		Y41G9A.5	-3.223651236	-0.9475	0.293921374	0.489403914
WBGene00018303		F41G3.10	-2.742575939	-0.9525	0.347301231	0.593984595
WBGene00010166	gmd-2	F56H6.5	-3.446263091	-0.955	0.277111751	0.463367002
WBGene00001762	gst-14	F37B1.3	-4.371910032	-0.9625	0.220155491	0.439880899
WBGene00013007		Y48E1B.8	-3.292393233	-0.9625	0.292340535	0.489403914
WBGene00015762		C14C6.8	-4.575605547	-0.9625	0.210354671	0.439880899
WBGene00017331	ugt-40	F10D2.5	-3.568814562	-0.9625	0.269697398	0.439880899
WBGene00010216		F57G8.7	-4.011757927	-0.9675	0.241166097	0.439880899
WBGene00010940		M163.8	-3.608280153	-0.9675	0.268133282	0.439880899
WBGene00019212		H19M22.3	-3.32324705	-0.9725	0.292635481	0.489403914
WBGene00018744		F53B3.6	-4.19501453	-0.9825	0.234206579	0.439880899
WBGene00020237	phat-4	T05B4.3	-2.774481874	-0.9875	0.355922311	0.593984595
WBGene00008028	scl-6	C39E9.4	-3.010192703	-0.99	0.328882599	0.50676114
WBGene00009722		F45D3.2	-6.048789268	-0.99	0.163669117	0
WBGene00010742		K10D6.2	-3.434743092	-0.99	0.288231164	0.463367002
WBGene00020975			-4.035926198	-0.99	0.245296854	0.439880899
WBGene00019730	asns-2	M02D8.4	-5.296631109	-0.9925	0.187383259	0.439880899
WBGene00011838		T19C4.1	-6.049352235	-0.9975	0.164893688	0
WBGene00017455		F14D2.7	-3.606946249	-1.0025	0.277935941	0.439880899
WBGene00000968	dhs-4	T05F1.10	-3.509992587	-1.005	0.286325391	0.463367002
WBGene00013636		Y105C5A.12	-4.145906021	-1.005	0.24240781	0.439880899
WBGene00020618		T20D4.12	-4.894859662	-1.01	0.206338909	0.439880899

WBGene00009257		F29G6.1	-6.853401943	-1.0125	0.147736848	0
WBGene00021127		W10C8.4	-2.958130368	-1.025	0.346502646	0.50676114
WBGene00003473	mtl-1	K11G9.6	-5.387438297	-1.0325	0.191649527	0.439880899
WBGene00013753		Y113G7A.16	-3.515913651	-1.0325	0.293664778	0.463367002
WBGene00019495	sdz-24	K07E8.3	-4.226597333	-1.0325	0.244286342	0.439880899
WBGene00002118	ins-35	K02E2.4	-4.614731286	-1.035	0.224281748	0.439880899
WBGene00022757		ZK488.5	-6.381694406	-1.0425	0.163357869	0
WBGene00011362		T02B5.1	-5.41006239	-1.0525	0.194544891	0.439880899
WBGene00011522	srap-1	T06D8.1	-3.425731378	-1.0525	0.307233663	0.463367002
WBGene00018451		F45D11.4	-3.566699783	-1.0525	0.295090718	0.439880899
WBGene00044536		F14F9.8	-3.968983581	-1.055	0.265811127	0.439880899
WBGene00012176		W01C9.2	-3.385036541	-1.0575	0.312404309	0.463367002
WBGene00002128	inx-6	C36H8.2	-2.759133846	-1.0625	0.385084617	0.593984595
WBGene00006642	tsp-16	F01E11.4	-3.147917746	-1.065	0.338318878	0.489403914
WBGene00013515		Y73F4A.2	-3.093718256	-1.07	0.345862135	0.50676114
WBGene00006583	tnc-2	ZK673.7	-2.838932109	-1.0775	0.379544124	0.50676114
WBGene00011674	cyp-13A8	T10B9.4	-4.92432049	-1.08	0.2193196	0.439880899
WBGene00019278		K01A2.3	-3.378824567	-1.08	0.319637785	0.463367002
WBGene00018448		F45D11.1	-3.718885079	-1.085	0.291754108	0.439880899
WBGene00008509		F01G10.6	-2.989192411	-1.09	0.364646985	0.50676114
WBGene00015761		C14C6.7	-6.540107402	-1.1	0.168192957	0
WBGene00008905		F17B5.1	-5.610906049	-1.105	0.19693789	0.439880899
WBGene00000739	col-166	T07H6.3	-3.20006679	-1.115	0.348430228	0.489403914
WBGene00008963		F19H8.2	-3.132927857	-1.115	0.35589712	0.50676114
WBGene00000731	col-158	D2023.7	-12.41790294	-1.1225	0.090393684	0
WBGene00009730	myo-6	F45G2.2	-4.355865902	-1.1225	0.257698475	0.439880899
WBGene00013853		ZC116.1	-2.985714349	-1.1225	0.37595693	0.50676114
WBGene00015622		C09B8.3	-3.525241284	-1.125	0.319127092	0.463367002
WBGene00017184	ncam-1	F02G3.1	-3.785335285	-1.125	0.297199565	0.439880899
WBGene00050897		C47A10.13	-5.269725667	-1.1275	0.213958007	0.439880899
WBGene00020561		T19C3.3	-3.657607541	-1.13	0.30894512	0.439880899
WBGene00015758	nhr-155	C14C6.4	-5.737772743	-1.1325	0.197376238	0.439880899
WBGene00003031	lin-46	R186.4	-3.27017878	-1.1475	0.350898247	0.489403914
WBGene00008591		F08H9.3	-4.325123778	-1.16	0.268200417	0.439880899
WBGene00001702	grd-13	W05E7.3	-5.546125272	-1.1775	0.212310386	0.439880899
WBGene00003623	nhr-25	F11C1.6	-8.811181895	-1.1825	0.13420447	0
WBGene00008490		F01D4.8	-7.722707128	-1.1875	0.153767323	0
WBGene00018215	igcm-1	F39H12.4	-3.967380124	-1.19	0.299946051	0.439880899
WBGene00015879		C17B7.5	-4.018871855	-1.1925	0.296725062	0.439880899
WBGene00007938		C34F6.1	-3.138943497	-1.2025	0.38309068	0.50676114
WBGene00022816	fbn-1	ZK783.1	-4.077890097	-1.2075	0.29610901	0.439880899
WBGene00045207		F13E9.14	-4.912937355	-1.2075	0.245779645	0.439880899
WBGene00007962		C35D6.5	-5.126765497	-1.21	0.236016256	0.439880899
WBGene00019311		K02E7.6	-4.124454183	-1.215	0.294584434	0.439880899
WBGene00015857		C16D9.1	-4.807587095	-1.2175	0.253245542	0.439880899
WBGene00009383		F35B12.3	-3.677742179	-1.23	0.334444325	0.439880899
WBGene00018460		F45D11.15	-5.369781243	-1.255	0.233715294	0.439880899
WBGene00008640		F10B5.3	-4.796362798	-1.2575	0.262177832	0.439880899
WBGene00001068	dpy-6	F16F9.2	-3.290647116	-1.2725	0.386702054	0.489403914
WBGene00014847		T27F6.9	-6.364870551	-1.285	0.201889416	0
WBGene00000399	cdh-7	R05H10.6	-3.678231367	-1.295	0.352071382	0.439880899
WBGene00018295	oac-29	F41E6.14	-6.195578168	-1.3525	0.218300853	0
WBGene00021183		Y9C9A.16	-6.352789898	-1.365	0.214866228	0
WBGene00044201		H39E23.3	-6.773530614	-1.365	0.201519721	0
WBGene00001391	far-7	K01A2.2	-5.268204239	-1.38	0.261948842	0.439880899
WBGene00011328		T01D3.3	-5.100327297	-1.425	0.279393834	0.439880899
WBGene00003514	myo-2	T18D3.4	-2.709683632	-1.455	0.536963055	0.593984595
WBGene00008816		F14F8.8	-3.602933323	-1.455	0.403837615	0.439880899
WBGene00000665	col-90	C29E4.1	-5.907731166	-1.4775	0.250096011	0
WBGene00020550		T17H7.1	-3.208811313	-1.64	0.511092688	0.489403914
WBGene00010738		K10D3.4	-3.506105385	-1.655	0.472033729	0.463367002
A_12_P177507			-6.585785319	-1.7675	0.268381053	0
WBGene00000618	col-41	T10B10.1	-7.477371338	-1.7875	0.239054598	0
A_12_P177508			-7.629425982	-1.825	0.239205414	0
A_12_P177506			-7.772460928	-1.835	0.236089961	0
WBGene00014074		ZK757.2	-17.14381915	-1.8475	0.107764786	0
A_12_P177504			-8.119552013	-1.9125	0.235542552	0
A_12_P177505			-8.641110929	-1.9325	0.223640226	0
A_12_P158985			-6.451331239	-2.1175	0.32822683	0
