# Supplementary material for: Transcription factors CEP‐1/p53 and CEH‐23 collaborate with AAK‐2/AMPK to modulate longevity in Caenorhabditis elegans
Source: Aging Cell. 2017 May 30;16(4):814–24. doi: 10.1111/acel.12619 (PMC5506430; doi:10.1111/acel.12619)
Supplement: Supplementary file 6 — Table S3 Genes that are commonly regulated by CEH‐23 and CEP‐1 in the isp‐1 mutant (identified by Statistical Analysis of Microarray (SAM) 1 class analysis with false discovery rate (FDR) = 0, 1.5 fold change cutoff). [file ACEL-16-814-s006.pdf]

Table S3: Genes are common regulated by CEH-23 and CEP-1 in *isp-1* mutant (identified by 1 class SAM with FDR=0, 1.5 fold change cutoff)

Gene ID	ublic nam	seq name	Score(d)	Numerator(r)	enominator(s+s(	q-value(%)
WBGene00011172		R09E10.2	3.321242093	1.871428571	0.563472496	0
WBGene00000989	dhs-26	ZK816.5	5.304029763	1.59	0.299772073	0
WBGene00022821		ZK813.2	3.406081031	1.558571429	0.457584953	0
WBGene00007459		C08F11.12	3.398065066	1.554285714	0.457403164	0
WBGene00012878		Y45F10C.2	3.36164662	1.528571429	0.454709136	0
WBGene00007507		C10C5.3	4.41292489	1.497142857	0.339263163	0
WBGene00011461			5.461024856	1.484285714	0.271796184	0
WBGene00008912		F17C8.7	3.298662118	1.437142857	0.435674466	0
WBGene00009622		F41E7.6	5.085148178	1.415612245	0.27838171	0
WBGene00019622		K10C2.7	3.34718895	1.381428571	0.412713053	0
WBGene00045105		Y7A9C.11	4.643049991	1.332857143	0.287064999	0
WBGene00003443	msp-50	C34F11.4	4.85740796	1.328571429	0.273514483	0
A_12_P177535			3.948940188	1.317142857	0.33354338	0
WBGene00000710	col-137	Y51H4A.9	3.790559862	1.314285714	0.346726015	0
WBGene00003464	msp-77	F32B6.6	4.656220839	1.31	0.281344044	0
WBGene00022875		ZK1248.4	4.020292492	1.308571429	0.325491598	0
WBGene00003452	msp-59	ZK354.11	4.346821779	1.304285714	0.300055024	0
WBGene00003425	msp-10	K07F5.2	4.277254151	1.301428571	0.3042673	0
WBGene00022002		Y59E9AR.7	4.941275671	1.297142857	0.262511737	0
WBGene00003426	msp-19	F36H12.7	4.795087603	1.292857143	0.269621173	0
WBGene00003465	msp-78	T13F2.11	4.757131519	1.292857143	0.27177242	0
A_12_P177537			4.204781436	1.288571429	0.306453843	0
WBGene00011462	scl-22	T05A10.5	4.843898229	1.282857143	0.264839822	0
WBGene00014675		C12D8.3	4.233844782	1.281428571	0.302663096	0
WBGene00003463	msp-76	ZK1251.6	4.055736624	1.281428571	0.315954582	0
WBGene00003469	msp-142	K05F1.2	4.523841452	1.278571429	0.282629584	0
A_12_P177538			3.883836955	1.277142857	0.328835343	0
A_12_P177536			3.693445848	1.275714286	0.345399483	0
WBGene00003456	msp-63	K05F1.7	4.169038	1.274285714	0.305654617	0
WBGene00003448	msp-55	C09B9.6	4.353330203	1.272857143	0.292386997	0
WBGene00011046		R05H10.7	5.156551855	1.27122449	0.246526075	0
WBGene00003457	msp-64	ZK1248.6	4.38852252	1.264285714	0.288089148	0
A_12_P177534			4.152102811	1.264285714	0.304492873	0
WBGene00022884		ZK1248.17	3.84716863	1.261428571	0.327884918	0
WBGene00013905	ugt-4	ZC455.5	4.018522564	1.26	0.313548071	0
WBGene00021997		Y59E9AR.1	4.794388701	1.251428571	0.261019423	0
WBGene00006047	ssp-19	C55C2.2	3.660937805	1.25	0.341442566	0
WBGene00008394		D1086.7	3.214510547	1.247142857	0.387972862	0
WBGene00003438	msp-45	F58A6.8	4.065258542	1.245714286	0.306429289	0
WBGene00003466	msp-79	T13F2.10	3.955864203	1.237142857	0.312736432	0
WBGene00003462	msp-74	F09C12.7	4.578860421	1.227142857	0.268001805	0
WBGene00003434	msp-38	K08F4.8	4.836645296	1.225714286	0.253422406	0
WBGene00020713		T23B3.5	6.711055053	1.218571429	0.18157673	0
WBGene00003444	msp-51	ZK354.5	4.405458624	1.214285714	0.275632078	0
WBGene00022005		Y59H11AM.:	4.699807195	1.21	0.25745737	0
WBGene00016752		C48E7.7	4.967216721	1.208571429	0.243309583	0
WBGene00014674		C12D8.2	4.467857551	1.204285714	0.26954434	0
WBGene00006056	sss-1	F32B6.5	5.011332135	1.198571429	0.239172219	0
WBGene00013070		Y51A2B.6	5.108201983	1.197959184	0.2345168	0
WBGene00022760		ZK546.3	5.3229933	1.195714286	0.224631935	0
WBGene00011173	acs-18	R09E10.3	3.992371178	1.194285714	0.299141954	0
WBGene00013474		Y69E1A.2	4.915924686	1.191428571	0.24236103	0
WBGene00019026		F58A6.9	5.305015463	1.18	0.22243102	0
WBGene00043743		Y59E9AL.2	4.642478495	1.18	0.254174575	0
WBGene00021207		Y18H1A.1	4.09633878	1.178571429	0.287713368	0
WBGene00017112		E03H12.5	5.304651454	1.177142857	0.221907672	0
WBGene00003424	msp-3	F26G1.7	4.431718667	1.177142857	0.265617686	0
WBGene00003431	msp-33	R05F9.8	4.243273492	1.174285714	0.27674052	0
WBGene00020350		T08B2.12	3.498220779	1.174285714	0.335680847	0
WBGene00011748		T13F2.9	5.076632133	1.172857143	0.231030556	0
WBGene00003435	msp-40	C33F10.9	4.560762097	1.168571429	0.256222843	0
A_12_P136645			4.939213555	1.165714286	0.236012125	0

WBGene00010373		H08M01.1	4.762282666	1.162857143	0.244180622	0
WBGene00009681	gipc-2	F44D12.4	4.831027776	1.161428571	0.240410245	0
WBGene00003449	msp-56	K07F5.3	4.347289619	1.161428571	0.267161536	0
WBGene00009308		F32A11.3	4.309115014	1.161428571	0.26952833	0
WBGene00015026		B0207.1	4.212930452	1.161428571	0.275681876	0
WBGene00016825		C50E10.1	4.64405559	1.157142857	0.249166453	0
WBGene00009695		F44F1.3	4.023480654	1.155714286	0.287242411	0
WBGene00007308		C04G2.9	3.716694111	1.154285714	0.310567854	0
WBGene00014116		ZK858.2	3.789647326	1.152857143	0.304212251	0
WBGene00003437	msp-42	F26G1.8	3.602880845	1.152857143	0.319982034	0
WBGene00020860	nspd-10	T27C10.7	5.548850699	1.151428571	0.207507578	0
WBGene00008541		F07A5.2	4.744096049	1.151428571	0.242707685	0
WBGene00009449		F35H8.4	5.145240621	1.15	0.223507526	0
WBGene00013887	nspa-5	ZC412.6	4.456489721	1.15	0.258050634	0
WBGene00009457		F36A2.10	4.818913595	1.148571429	0.23834655	0
WBGene00012637		Y38H8A.3	4.809647931	1.148571429	0.238805718	0
WBGene00003433	msp-37	K08F4.10	4.716708195	1.145714286	0.242905484	0
WBGene00011336	ubxn-5	T01E8.9	4.348314475	1.145714286	0.263484689	0
WBGene00016288		C31H1.5	4.668848955	1.14	0.244171532	0
WBGene00009682	msd-2	F44D12.5	4.331979507	1.134285714	0.261840046	0
WBGene00010091	ssp-35	F55C5.1	4.770996327	1.131428571	0.237147232	0
WBGene00003442	msp-49	C34F11.6	3.965526807	1.131428571	0.285316082	0
WBGene00003429	msp-31	R05F9.13	4.654810502	1.125714286	0.241838907	0
WBGene00007794		C28D4.7	4.120477888	1.125714286	0.273199934	0
WBGene00014665		C04G2.3	3.713015993	1.125714286	0.303180565	0
WBGene00016742		C48B6.4	4.590779794	1.122571429	0.244527396	0
WBGene00008754		F13E9.5	4.578839148	1.118571429	0.244291488	0
WBGene00012294	nspa-8	W06A7.5	4.481291659	1.117142857	0.24929037	0
WBGene00013290		Y57G11A.2	4.000047117	1.117142857	0.279282425	0
WBGene00044474		F56D6.12	3.602456806	1.117142857	0.31010583	0
WBGene00016512		C38C3.3	4.654119503	1.115714286	0.239726179	0
WBGene00008623		F09E8.1	6.281473573	1.113673469	0.177294938	0
WBGene00009631		F42E8.2	4.641227759	1.112857143	0.239776456	0
WBGene00007159		B0379.7	5.042577886	1.111428571	0.220408806	0
WBGene00015987		C18G1.9	4.261439295	1.107142857	0.259804911	0
WBGene00011176		R09E10.6	4.748136508	1.104285714	0.232572444	0
WBGene00015908		C17F3.3	4.599200423	1.104285714	0.240103847	0
WBGene00008801	acp-3	F14E5.3	3.972177604	1.104285714	0.278005121	0
A_12_P166055			4.525300298	1.102857143	0.243709162	0
WBGene00015244		B0524.5	4.090759315	1.101428571	0.269247953	0
WBGene00009324		F32B6.10	4.355589594	1.1	0.252549047	0
WBGene00007601		C15C6.2	4.338266652	1.1	0.253557489	0
WBGene00012211	ssp-37	W02D9.5	4.888866719	1.097142857	0.224416602	0
WBGene00010486		K01H12.4	4.567959556	1.095714286	0.239869524	0
WBGene00044177		C30G7.3	3.998597462	1.095714286	0.274024654	0
WBGene00006050	ssq-1	K07F5.11	3.508954325	1.095714286	0.312262339	0
WBGene00014158		ZK938.1	4.299564531	1.094285714	0.254510825	0
WBGene00022706		ZK354.3	4.461411148	1.09244898	0.244866241	0
WBGene00009549		F38H4.5	3.571929353	1.092380952	0.305823784	0
WBGene00009884		F49C12.15	3.984271015	1.09	0.273575767	0
WBGene00016410		C34E10.9	4.643232055	1.088571429	0.234442607	0
WBGene00003470	msp-152	ZK546.6	4.117845055	1.088571429	0.264354635	0
WBGene00012595		Y38E10A.17	4.505907756	1.084285714	0.240636465	0
WBGene00016399		C34D4.3	4.230144013	1.084285714	0.256323593	0
WBGene00015907		C17F3.1	5.043215449	1.082857143	0.214715622	0
WBGene00020840		T27A3.4	4.162322358	1.082857143	0.260156963	0
WBGene00012937		Y47D3A.31	4.094755082	1.082857143	0.264449795	0
WBGene00010633	nspd-2	K07F5.5	3.896630424	1.082857143	0.277895778	0
WBGene00017851		F27C1.1	3.818608315	1.08	0.282825551	0
WBGene00016541		C39H7.1	4.191366159	1.078571429	0.257331712	0
WBGene00015937		C17H12.12	4.671206934	1.077142857	0.23059198	0
WBGene00007301		C04F12.7	4.089703718	1.077142857	0.263379191	0
WBGene00022689	math-48	ZK250.6	4.671533838	1.076938776	0.230532158	0
WBGene00014239		ZK1225.5	4.223394918	1.075714286	0.254703694	0
WBGene00008590		F08H9.2	3.49883874	1.075714286	0.307448947	0

WBGene00007248	catp-4	C01G12.8	3.999058776	1.074285714	0.26863464	0
WBGene00007320		C05B5.2	4.670209927	1.071428571	0.229417647	0
WBGene00013858	ssp-34	ZC168.6	4.651491437	1.071428571	0.230340867	0
WBGene00008433	2-Mar	D2089.2	4.581673414	1.071428571	0.233850926	0
WBGene00021996		Y59E9AL.6	4.835681686	1.068571429	0.220976379	0
WBGene00007792		C28D4.4	4.237603525	1.068571429	0.252164088	0
WBGene00009446		F35H8.1	4.558058693	1.068061224	0.23432371	0
WBGene00019430		K06A5.2	4.481652299	1.067142857	0.23811371	0
WBGene00022780		ZK622.1	4.379443466	1.067142857	0.243670883	0
WBGene00004966	spe-12	T02E1.1	3.926284258	1.067142857	0.271794599	0
WBGene00022622		ZC477.10	3.764469753	1.065714286	0.283098114	0
WBGene00013800		Y116A8C.23	4.622451664	1.062040816	0.229757041	0
WBGene00008383		D1081.5	3.98807833	1.061428571	0.266150382	0
WBGene00009005		F21C3.6	3.80351813	1.061428571	0.279064943	0
WBGene00006040	ssp-11	T28H11.6	4.542609575	1.06	0.23334605	0
WBGene00020905		T28H11.7	4.4631573	1.057142857	0.23685987	0
WBGene00009959		F53B6.4	4.646573394	1.051428571	0.226280418	0
WBGene00007977	gska-3	C36B1.10	4.100892783	1.051428571	0.256390164	0
WBGene00007795		C28D4.8	4.673384038	1.05	0.224676592	0
WBGene00016440	gipc-1	C35D10.2	4.830959914	1.047142857	0.216756685	0
WBGene00012357		W09D6.4	4.024790208	1.045714286	0.259818334	0
WBGene00003432	msp-36	C04G2.4	3.504484031	1.045714286	0.298393223	0
WBGene00020986		W03D8.3	4.477637057	1.044285714	0.233222501	0
WBGene00009548		F38H4.4	4.016207147	1.044285714	0.260017892	0
WBGene00016351		C33F10.1	3.96440553	1.044285714	0.263415462	0
WBGene00011460	ttr-14	T05A10.3	4.430707704	1.042857143	0.235370332	0
WBGene00019920	acs-15	R07C3.4	3.673515263	1.042857143	0.283885344	0
WBGene00010137	ztf-26	F55H12.6	4.668963319	1.04	0.22274752	0
WBGene00007793		C28D4.5	4.453807415	1.04	0.233508076	0
WBGene00012925	wht-8	Y47D3A.11	4.49226914	1.038571429	0.231190829	0
WBGene00011295		R102.8	4.149511224	1.038571429	0.250287654	0
WBGene00017902		F28E10.4	3.918674781	1.038571429	0.265031289	0
WBGene00013723		Y106G6H.13	4.742025066	1.037142857	0.218713069	0
WBGene00014104		ZK849.6	4.279265293	1.037142857	0.242364702	0
WBGene00009609	oac-27	F41D3.4	4.407282416	1.036428571	0.235162732	0
WBGene00013696		Y106G6A.4	4.739600211	1.035714286	0.218523555	0
WBGene00015094		B0261.6	4.669971798	1.034285714	0.221475794	0
WBGene00012185		W01F3.2	4.117334655	1.034285714	0.251202732	0
WBGene00016680		C45G9.9	4.02197946	1.034285714	0.257158378	0
WBGene00007307		C04G2.8	3.633376926	1.034285714	0.284662378	0
WBGene00044393		ZK1248.20	4.009038379	1.032857143	0.257632141	0
WBGene00004965	spe-11	F48C1.7	4.685820182	1.031428571	0.220116977	0
WBGene00022763		ZK546.7	4.314380776	1.031428571	0.23906758	0
WBGene00012781	nspd-7	Y43C5A.1	4.333985334	1.03	0.237656549	0
WBGene00011968		T23G11.1	3.939453135	1.03	0.26145761	0
WBGene00014247		ZK1307.4	4.652831967	1.028571429	0.221063523	0
WBGene00010933		M162.7	4.050675535	1.028571429	0.253925899	0
WBGene00013194		Y54E2A.9	4.518159172	1.027142857	0.227336581	0
WBGene00022652		ZK84.5	3.457394333	1.027142857	0.297085828	0
WBGene00006052	ssq-3	ZC477.1	3.996425517	1.025714286	0.256657926	0
WBGene00045355		D1086.17	4.809506739	1.024285714	0.212971053	0
WBGene00022008		Y59H11AM.4	4.146432698	1.022857143	0.246683648	0
WBGene00009948		F52H3.6	4.451730643	1.021428571	0.229445277	0
WBGene00018526		F47B3.2	4.235445787	1.021428571	0.241161999	0
WBGene00022754	nspd-1	ZK484.8	4.182803691	1.018571429	0.243514041	0
WBGene00016058	nspd-3	C24D10.7	4.413584307	1.014285714	0.229809978	0
WBGene00014246		ZK1307.3	4.306840764	1.014285714	0.235505738	0
WBGene00016675		C45G9.4	4.110501944	1.012857143	0.246407168	0
WBGene00022229		Y73B6A.2	3.952181819	1.01244898	0.256174697	0
WBGene00004904	snf-5	Y46G5A.30	4.728990884	1.011428571	0.213878309	0
WBGene00022674		ZK177.9	4.253616942	1.011428571	0.237780831	0
WBGene00008660	clec-153	F10F2.8	3.902091561	1.011428571	0.25920165	0
WBGene00012809		Y43F8A.2	4.144656059	1.01	0.243687289	0
WBGene00023306		F59A7.10	4.090299475	1.01	0.246925685	0
WBGene00013303		Y57G11C.5	4.589600716	1.008571429	0.219751454	0

WBGene00010381		H12D21.5	4.0716297	1.008571429	0.247707062	0
WBGene00007987		C36H8.1	3.717053379	1.008571429	0.271336278	0
WBGene00019950	clec-175	R08C7.6	3.41586918	1.008571429	0.295260555	0
WBGene00022621		ZC477.7	4.162998703	1.007142857	0.241927257	0
WBGene00007060	wht-6	T26A5.1	4.06703463	1.007142857	0.247635673	0
WBGene00011429		T04C12.7	3.997427311	1.007142857	0.25194776	0
WBGene00017059		D2062.7	4.005161038	1.005714286	0.251104581	0
WBGene00010544		K03H1.9	4.747407357	1.004285714	0.211544036	0
WBGene00077519		T27C10.8	4.87313972	1.002857143	0.205792815	0
WBGene00013523		Y73F8A.14	4.73175019	1.002857143	0.211942115	0
WBGene00018134		F37A4.4	3.517693079	1.002857143	0.285089438	0
WBGene00019406	acdh-8	K05F1.3	4.509086337	1.001428571	0.222091239	0
WBGene00020414		T10E9.4	3.534948579	1.001428571	0.283293674	0
WBGene00020353		T08B6.4	4.331182398	1	0.230883835	0
WBGene00015034		B0207.11	4.140915976	1	0.241492463	0
WBGene00015516		C06A8.6	4.550161829	0.998571429	0.219458443	0
WBGene00012223		W03C9.8	4.472191302	0.998571429	0.223284596	0
WBGene00011795		T16A9.5	4.352897809	0.998571429	0.22940383	0
WBGene00008950	wht-5	F19B6.4	3.570327961	0.998571429	0.279686191	0
WBGene00012679		Y39B6A.18	3.654258312	0.997809524	0.273053911	0
WBGene00014106		ZK856.5	4.626808934	0.997142857	0.215514164	0
WBGene00013526		Y73F8A.20	3.643365545	0.995952381	0.273360542	0
WBGene00013888	nspa-3	ZC412.7	3.863952644	0.995714286	0.257693191	0
WBGene00007230		C01G10.1	3.962749021	0.995238095	0.251148405	0
WBGene00044062	snb-6	T14D7.3	4.458887793	0.994285714	0.222989624	0
WBGene00009143		F26A3.5	3.543720597	0.994285714	0.28057678	0
WBGene00017058		D2062.6	4.62861809	0.992857143	0.21450401	0
WBGene00018122	ttbk-2	F36H12.8	3.774985511	0.992857143	0.263009524	0
WBGene00019248		H27M09.5	3.533420588	0.992857143	0.280990366	0
WBGene00006051	ssq-2	T28H11.5	4.083140947	0.991428571	0.242810274	0
WBGene00013713		Y106G6G.4	4.000406977	0.991428571	0.247831927	0
WBGene00004909	snf-10	Y32F6A.2	3.834532008	0.991428571	0.258552692	0
WBGene00012926		Y47D3A.13	6.121709503	0.99	0.161719533	0
WBGene00017325		F10C1.3	4.909326593	0.99	0.201656985	0
WBGene00021113	gsp-3	W09C3.6	4.916959125	0.988571429	0.201053416	0
WBGene00009491		F36G9.15	3.338766177	0.988571429	0.296088847	0
WBGene00010611		K07A1.4	4.759216348	0.987142857	0.207417101	0
WBGene00013956		ZK265.3	4.23388503	0.987142857	0.233152967	0
WBGene00014238		ZK1225.4	3.902753332	0.987142857	0.252934985	0
WBGene00018087		F36D4.1	4.042608756	0.986734694	0.244083648	0
WBGene00011491		T05F1.5	4.586873796	0.985714286	0.214898933	0
WBGene00021969		Y57G7A.6	3.961740186	0.985714286	0.248808412	0
WBGene00014169		ZK945.7	4.167760156	0.982857143	0.235823825	0
WBGene00016274		C30G12.2	8.235350612	0.981428571	0.119172652	0
WBGene00014755		F40F12.8	3.805612521	0.981428571	0.257889779	0
WBGene00010650		K08C9.1	3.929346102	0.98	0.249405365	0
WBGene00019501		K07E12.2	3.459748501	0.98	0.283257584	0
WBGene00044684		T08G11.2	4.399315703	0.978571429	0.222437191	0
WBGene00013473		Y69E1A.1	4.876991207	0.975714286	0.200064803	0
WBGene00011378		T02E1.6	4.205388146	0.975714286	0.232015275	0
WBGene00011214		R10E9.2	5.124864534	0.974285714	0.190109555	0
WBGene00018119		F36H12.3	4.087363626	0.974285714	0.238365314	0
WBGene00011669		T09F5.10	3.924426802	0.972857143	0.247897895	0
WBGene00017386	nspd-5	F11G11.8	3.788922043	0.972857143	0.256763568	0
WBGene00011467	decr-1.3	T05C12.3	3.785934628	0.972857143	0.256966176	0
WBGene00004908	snf-9	C49C3.1	3.305081126	0.972857143	0.294351971	0
WBGene00014127		ZK892.3	3.936207149	0.971428571	0.246793051	0
WBGene00002212	kin-31	B0523.1	4.629427969	0.97	0.209529127	0
WBGene00016707		C46E10.1	4.337400796	0.97	0.223636239	0
WBGene00002204	kin-21	W08D2.8	3.600591195	0.97	0.269400203	0
WBGene00007998		C38C6.5	4.160203739	0.969183673	0.232965435	0
WBGene00010136		F55H12.5	4.752428698	0.968571429	0.203805568	0
WBGene00015106		B0280.11	4.389210163	0.968571429	0.220671007	0
WBGene00021016		W03G9.5	4.209975798	0.968571429	0.230065795	0
WBGene00021288	clec-123	Y25C1A.1	3.573295983	0.968571429	0.27105827	0

WBGene00021678		Y48G1C.5	3.437479493	0.967959184	0.281589806	0
WBGene00016414		C34F11.2	4.403633119	0.967142857	0.219623849	0
WBGene00018081		F36A4.2	3.45606959	0.967142857	0.279838942	0
WBGene00015629		C09B9.4	4.03499546	0.965714286	0.239334665	0
WBGene00019586		K09F6.3	3.881163335	0.965714286	0.248820831	0
WBGene00009685		F44D12.8	3.631652111	0.965714286	0.265915968	0
WBGene00044256		Y45F10C.6	3.294423525	0.965714286	0.293136046	0
WBGene00012547		Y37D8A.5	4.340314408	0.964285714	0.222169554	0
WBGene00008659	clec-151	F10F2.7	4.889743609	0.962857143	0.196913626	0
WBGene00019260		H34I24.1	4.32886993	0.962857143	0.222426906	0
WBGene00003430	msp-32	R05F9.3	4.109056899	0.962857143	0.23432558	0
WBGene00007448		C08F8.6	3.686478639	0.962857143	0.261186145	0
WBGene00009031		F21H7.5	4.234647429	0.961428571	0.227038635	0
WBGene00044447		ZK688.10	3.556450333	0.961428571	0.270333755	0
WBGene00018840		F54H5.3	3.547963989	0.961428571	0.270980363	0
WBGene00022771		ZK616.1	3.540845803	0.961428571	0.271525117	0
WBGene00011954		T23F11.2	4.484533312	0.96	0.214069098	0
WBGene00013165		Y53F4B.19	3.988959562	0.96	0.24066426	0
A_12_P111814			5.043358799	0.958571429	0.190066078	0
WBGene00015689		C10G11.9	4.374481655	0.957142857	0.218801434	0
WBGene00007610		C15H7.3	3.917773815	0.957142857	0.24430784	0
WBGene00018563		F47D12.7	3.519413796	0.957142857	0.271960875	0
WBGene00001603	gln-2	K03H1.1	4.680251846	0.955714286	0.204201465	0
WBGene00019561		K09C6.7	4.236937287	0.955714286	0.225567248	0
WBGene00013879		ZC376.8	4.149024275	0.955714286	0.230346757	0
WBGene00022090		Y69A2AR.19	3.494561376	0.955714286	0.273486193	0
WBGene00009864		F49B2.4	4.351441542	0.954285714	0.219303352	0
WBGene00008725		F13A7.7	3.377535285	0.954285714	0.282539081	0
WBGene00007763		C27B7.6	4.194971379	0.953265306	0.227240002	0
WBGene00013087		Y51B9A.5	4.661881821	0.952857143	0.204393243	0
WBGene00022467		Y119C1B.1	3.833155432	0.952857143	0.248582965	0
WBGene00013452		Y67A10A.3	3.883012839	0.951904762	0.245145922	0
WBGene00013988		ZK512.10	4.003725285	0.951428571	0.237635828	0
WBGene00044333		C01G12.13	3.532768663	0.951428571	0.269315277	0
WBGene00022162		Y71G12B.27	4.293863679	0.95	0.221245962	0
A_12_P181510			4.146954822	0.949591837	0.228985334	0
WBGene00011619		T08G3.7	3.868875639	0.949047619	0.245303211	0
WBGene00050915		Y6E2A.10	4.145416671	0.947142857	0.228479531	0
WBGene00014197		ZK1053.2	3.556502321	0.947142857	0.266313015	0
WBGene00017026		D1037.5	3.81415847	0.944857143	0.24772362	0
WBGene00011617		T08G3.4	3.105825956	0.944761905	0.304190228	0
WBGene00013170		Y53F4B.24	4.954073239	0.944571429	0.190665617	0
WBGene00009714		F44G4.5	4.276779506	0.944285714	0.22079364	0
WBGene00010679		K08F4.5	3.824391676	0.944285714	0.246911351	0
WBGene00017209		F07E5.4	4.244177879	0.942857143	0.222153069	0
WBGene00009344		F32H2.7	3.56535403	0.942857143	0.264449795	0
WBGene00020071		R13H9.5	3.815035869	0.941428571	0.246767948	0
WBGene00008581	acl-13	F08G5.2	3.467471393	0.941428571	0.271502909	0
WBGene00009217	oig-7	F28D1.8	3.559122541	0.940714286	0.264310732	0
A_12_P159210			3.824348157	0.94	0.245793521	0
A_12_P159213			3.79833681	0.94	0.247476737	0
WBGene00018001		F33D11.2	4.430453798	0.938571429	0.211845439	0
WBGene00010510	ent-3	K02E11.1	4.222771738	0.938571429	0.222264306	0
WBGene00019410		K05F1.9	3.998631836	0.937142857	0.234365877	0
WBGene00044674		B0280.17	4.227671407	0.935714286	0.221330893	0
WBGene00009755		F46A9.2	3.483514651	0.935714286	0.268612129	0
WBGene00018235		F40E12.2	3.629253778	0.934285714	0.257431905	0
WBGene00010895		M28.4	3.228656733	0.933571429	0.289151652	0
WBGene00014007		ZK596.2	4.201620136	0.932857143	0.222023199	0
WBGene00009377		F34D10.8	3.925948002	0.932857143	0.237613219	0
WBGene00018497		F46F5.6	3.716130904	0.931428571	0.250644715	0
WBGene00010366		H05L14.1	3.431622025	0.931428571	0.271425164	0
WBGene00015247		B0545.4	4.192417859	0.93122449	0.222121106	0
WBGene00012171		W01B6.4	4.786091774	0.930544218	0.194426739	0
WBGene00015193	clec-117	B0432.12	4.621240578	0.93	0.201244662	0

WBGene00013437		Y66D12A.11	4.329092302	0.93	0.214825634	0
WBGene00010869		M05B5.1	4.048973597	0.93	0.22968784	0
WBGene00020416		T10E9.6	4.566725436	0.928571429	0.203334192	0
WBGene00011466		T05C12.1	4.428924267	0.928571429	0.209660715	0
WBGene00015688		C10G11.8	4.091947597	0.928571429	0.22692652	0
WBGene00010906		M88.3	3.868512317	0.928571429	0.24003321	0
WBGene00021110		W09C3.2	3.763891171	0.928571429	0.246705175	0
WBGene00006048	ssp-31	ZK1225.6	3.711479273	0.928571429	0.250189038	0
A_12_P171290			4.081877452	0.928571429	0.227486356	0
WBGene00009215	thn-2	F28D1.5	4.622458291	0.925714286	0.200264497	0
WBGene00016416		C34F11.5	4.606431908	0.925714286	0.200961244	0
WBGene00022589		ZC317.6	4.235364534	0.925714286	0.218567795	0
WBGene00018347		F42C5.5	4.061239125	0.925714286	0.227938877	0
WBGene00014777		F58A4.12	3.942389633	0.925714286	0.23481045	0
WBGene00017869		F28A10.1	3.170598401	0.924761905	0.29166794	0
WBGene00009185		F27C8.5	4.256108101	0.922857143	0.216831227	0
WBGene00010046		F54D1.1	3.929805395	0.922857143	0.234835329	0
WBGene00021386		Y37F4.5	3.368917771	0.921904762	0.273650123	0
WBGene00005012		F26F4.2	4.510716519	0.921428571	0.204275433	0
WBGene00012711		Y39E4A.1	4.001476562	0.921428571	0.23027214	0
WBGene00014179		ZK1010.5	3.609538211	0.921428571	0.255276026	0
WBGene00015032		B0207.9	3.978556204	0.92	0.231239664	0
A_12_P159214			3.7557316	0.92	0.244958932	0
WBGene00008772	irld-4	F13G11.2	4.740416633	0.918571429	0.193774408	0
WBGene00013809		Y116A8C.33	4.154418916	0.918571429	0.221107078	0
WBGene00008529		F02E9.3	3.943274543	0.917142857	0.232584074	0
WBGene00007306		C04G2.5	3.912622602	0.917142857	0.234406164	0
WBGene00010072		F54F12.1	3.320345267	0.916	0.275874925	0
WBGene00016161		C27D6.3	4.904714017	0.915714286	0.186700852	0
WBGene00013521		Y73F8A.12	4.583580714	0.915714286	0.199781425	0
WBGene00010563		K04G2.4	3.658813749	0.914285714	0.249885831	0
WBGene00010634		K07F5.6	4.016184559	0.912857143	0.22729462	0
WBGene00013279		Y57A10B.7	3.5249631	0.912857143	0.258969276	0
WBGene00020674		T22B7.7	3.337739493	0.912857143	0.273495623	0
WBGene00014681		C25D7.14	4.683404523	0.912380952	0.194811477	0
WBGene00015467	basl-1	C05D2.3	4.592349656	0.911428571	0.19846672	0
WBGene00020987		W03D8.5	4.318046962	0.911428571	0.211074261	0
WBGene00017672		F21F3.2	4.308906473	0.911428571	0.211522013	0
WBGene00018125	rmd-4	F36H12.11	4.308574685	0.91	0.211206737	0
WBGene00010574		K04H4.5	4.136479556	0.91	0.219993835	0
WBGene00020971		W03B1.1	3.813230493	0.91	0.2386428	0
WBGene00009269		F30A10.12	3.734294073	0.91	0.243687289	0
WBGene00018178		F38E1.3	3.5161273	0.91	0.258807467	0
WBGene00015306		C01G5.4	3.24626134	0.91	0.280322471	0
WBGene00044777		T02B11.9	4.39410626	0.909142857	0.206900517	0
WBGene00019255		H32C10.1	4.56557982	0.908571429	0.199004609	0
WBGene00009402		F35C11.3	4.370506412	0.908571429	0.207886991	0
WBGene00013827		Y116F11B.1	3.680098669	0.907346939	0.246555057	0
WBGene00016843		C50F7.3	4.929444825	0.907142857	0.18402536	0
WBGene00006553	tbx-34	Y47D3A.10	4.054592343	0.907142857	0.223732198	0
A_12_P159211			3.711761798	0.907142857	0.244396841	0
WBGene00019024		F58A6.5	4.820464015	0.905714286	0.18788944	0
WBGene00019568		K09D9.11	4.29524586	0.905612245	0.210840607	0
WBGene00007777		C27D8.1	4.327480295	0.904285714	0.208963566	0
WBGene00015765		C14C11.1	4.259305319	0.902857143	0.211972863	0
WBGene00015348		C02F5.5	3.969014986	0.902857143	0.227476375	0
WBGene00002120	ins-37	F08G2.6	4.593829668	0.901428571	0.196225946	0
WBGene00044122		T28B8.6	4.529305073	0.901428571	0.199021386	0
WBGene00007778		C27D8.2	4.422630914	0.901428571	0.203821795	0
WBGene00009956		F53B2.5	4.421575074	0.901428571	0.203870466	0
WBGene00013700		Y106G6D.3	4.143156135	0.901428571	0.217570505	0
WBGene00012689		Y39B6A.30	4.039981	0.901428571	0.223126933	0
WBGene00013052	scrm-7	Y50E8A.9	3.823824097	0.901428571	0.235740073	0
WBGene00010829		M02B1.4	3.656103662	0.901428571	0.246554435	0
WBGene00012087		T27E7.1	4.143216544	0.9	0.217222535	0

WBGene00017910		F28H1.5	4.586878234	0.898571429	0.195900432	0
WBGene00014240	htas-1	ZK1251.1	4.264327173	0.898571429	0.210718219	0
WBGene00013476		Y69E1A.4	4.007338031	0.897142857	0.223875014	0
WBGene00017555	nep-10	F18A12.6	3.658503451	0.897142857	0.245221269	0
WBGene00016612		C43G2.3	3.263532091	0.897142857	0.274899352	0
WBGene00009028		F21H7.2	4.236954088	0.895714286	0.211405238	0
WBGene00016461		C35E7.9	4.032193402	0.895714286	0.222140705	0
WBGene00015422		C04E6.5	3.922200513	0.895714286	0.228370345	0
WBGene00010883		M7.7	3.908906386	0.895714286	0.229147029	0
WBGene00017542		F17E9.5	3.858772829	0.895714286	0.23212413	0
WBGene00020468		T13A10.1	3.610488111	0.895714286	0.248086757	0
WBGene00016753	oac-9	C48E7.8	3.480146365	0.894761905	0.257104676	0
WBGene00008243		C50H2.7	4.33641903	0.894285714	0.206226776	0
WBGene00010651		K08C9.2	3.690785493	0.894285714	0.242302273	0
WBGene00013175		Y53F4B.36	3.613458753	0.894285714	0.247487456	0
A_12_P159212			3.39719618	0.894285714	0.263242294	0
WBGene00009473		F36D3.8	3.314738271	0.894285714	0.269790747	0
WBGene00008650		F10D11.4	3.751788179	0.893214286	0.23807695	0
WBGene00012855		Y44A6D.5	3.750745167	0.892857143	0.238047935	0
WBGene00009471		F36D3.5	3.587305242	0.892857143	0.248893552	0
WBGene00012177	decr-1.2	W01C9.4	3.508985598	0.892857143	0.254448791	0
WBGene00018301		F41G3.5	3.132308235	0.892857143	0.285047663	0
WBGene00006057	sss-2	F47B8.11	4.863584703	0.891428571	0.183286326	0
WBGene00007222		C01F6.2	4.468905103	0.891428571	0.199473596	0
WBGene00015634		C09D4.3	4.433789731	0.891428571	0.201053416	0
WBGene00023424		C53D6.10	3.836586485	0.891428571	0.232349401	0
WBGene00008423		D2045.5	4.523183741	0.888571429	0.196448228	0
WBGene00019015		F57F4.1	4.483655458	0.888571429	0.198180132	0
WBGene00019951		R08C7.8	4.106547701	0.888571429	0.216379181	0
WBGene00022705		ZK354.2	3.583394553	0.888571429	0.247969185	0
WBGene00008899		F16H6.9	3.1615365	0.887755102	0.280798625	0
WBGene00019461		K07A3.3	3.76721315	0.887142857	0.235490486	0
WBGene00020580		T19D12.5	3.559203433	0.887142857	0.249253203	0
WBGene00011790		T15H9.5	3.589799547	0.885102041	0.246560297	0
WBGene00015997		C18H7.7	4.678433836	0.884285714	0.189013192	0
WBGene00009670		F43G9.8	4.623282196	0.884285714	0.191267951	0
WBGene00020223		T05A7.6	3.488873822	0.884285714	0.253458783	0
WBGene00011134	ssp-33	R08A2.3	4.226247207	0.882857143	0.208898604	0
WBGene00015093		B0261.5	4.146664897	0.882857143	0.212907762	0
WBGene00014813		M176.9	4.120459469	0.882857143	0.214261819	0
WBGene00020990		W03D8.9	3.875148657	0.882857143	0.227825361	0
WBGene00013744		Y111B2A.27	3.688021223	0.882857143	0.239385049	0
WBGene00004901	snf-2	F55H12.1	3.100539278	0.8825	0.284627905	0
WBGene00015384			4.393099007	0.881428571	0.20063936	0
WBGene00018792		F54C1.8	4.057880321	0.881428571	0.217214038	0
WBGene00022876		ZK1248.5	3.971349269	0.881428571	0.221946878	0
WBGene00020265	fbxa-196	T05H4.2	3.73813969	0.881428571	0.235793374	0
WBGene00012870		Y45F10B.2	3.517854159	0.881428571	0.250558588	0
WBGene00008154		C47E12.11	3.435600622	0.881428571	0.256557344	0
WBGene00007081		AH6.3	3.141539066	0.881428571	0.280572214	0
WBGene00007337		C05C12.5	4.539498431	0.88	0.193854016	0
WBGene00017955		F31E8.5	4.35425901	0.88	0.202100977	0
WBGene00016954		C55C3.4	3.873228012	0.88	0.22720067	0
WBGene00014129		ZK892.5	3.750456254	0.88	0.234638119	0
WBGene00008487		F01D4.3	3.648887616	0.88	0.24116939	0
WBGene00010447		K01A6.5	3.833024257	0.879438776	0.22943731	0
WBGene00022094		Y69A2AR.23	5.761378055	0.878571429	0.152493279	0
WBGene00044633		F54H12.7	5.660010536	0.878571429	0.155224345	0
WBGene00020019		R12B2.3	4.626814121	0.878571429	0.189886908	0
WBGene00009149		F26D2.10	4.204559174	0.878571429	0.208956847	0
WBGene00021650		Y47G6A.26	3.796609136	0.878571429	0.231409502	0
WBGene00014996		ZK1251.5	3.438902857	0.878571429	0.255480153	0
WBGene00009941		F52F12.8	3.493257413	0.878367347	0.251446499	0
WBGene00021398		Y38C1AA.7	3.878214541	0.877142857	0.226171824	0
WBGene00019949		R08C7.5	3.618911276	0.877142857	0.242377552	0

WBGene00009708		F44G3.7	3.589944369	0.877142857	0.244333273	0
WBGene00044362		F42G2.7	3.302559746	0.877142857	0.265594849	0
WBGene00011425		T04B2.7	4.000402095	0.875714286	0.218906566	0
WBGene00020715	nspd-4	T23B7.1	3.506190882	0.875714286	0.249762296	0
WBGene00019425		K06A1.2	3.410563588	0.875714286	0.256765272	0
WBGene00015192		B0432.11	4.201357893	0.874571429	0.208163991	0
WBGene00013707		Y106G6E.3	4.417811013	0.874285714	0.197900207	0
WBGene00009501		F37A8.1	3.800285072	0.874285714	0.230057929	0
WBGene00015690		C10G11.10	3.794388344	0.874285714	0.230415454	0
WBGene00017057		D2062.5	5.178012593	0.872653061	0.168530502	0
WBGene00044243		F16C3.4	4.469527043	0.871428571	0.194971093	0
WBGene00009513	sfxn-1.2	F37H8.4	4.190565087	0.871428571	0.207950134	0
WBGene00011133		R08A2.2	3.99954798	0.871428571	0.217881765	0
WBGene00018000		F33D11.1	3.357093701	0.871428571	0.259578269	0
WBGene00019785		M70.3	4.189879708	0.87	0.207643193	0
WBGene00011203		R10E4.7	3.939774826	0.87	0.220824803	0
WBGene00019562		K09C6.8	3.932532972	0.87	0.221231457	0
WBGene00015820		C16A11.7	3.770590811	0.87	0.230733072	0
WBGene00011918		T22C1.8	3.216839908	0.87	0.270451755	0
WBGene00013785	nep-23	Y116A8C.4	3.098856809	0.87	0.280748693	0
WBGene00016491	acdh-5	C37A2.3	3.984679098	0.867142857	0.217619245	0
WBGene00044498		Y59E9AR.10	3.933913262	0.867142857	0.220427549	0
WBGene00004972	spe-26	R10H10.2	3.583930737	0.867142857	0.241953018	0
WBGene00019086		F59A6.4	3.422571683	0.867142857	0.253360028	0
WBGene00009039		F22B3.8	3.789438179	0.866326531	0.228616088	0
WBGene00022632		ZC581.2	3.681827345	0.865714286	0.23513169	0
WBGene00011910		T22B3.2	3.624301897	0.865714286	0.238863734	0
WBGene00016949		C55C2.3	4.430764494	0.864571429	0.195129177	0
WBGene00018332		F42A9.3	4.274818001	0.864285714	0.202180704	0
WBGene00000019	abt-1	C24F3.5	4.26927877	0.864285714	0.202443026	0
WBGene00018360	irld-8	F42G8.9	3.495516363	0.862857143	0.246846833	0
A_12_P171291			4.973849854	0.862571429	0.173421284	0
WBGene00010181		F57A8.6	4.44002824	0.862142857	0.194175084	0
A_12_P163028			3.206324128	0.862	0.268843687	0
WBGene00077450		K06B4.15	4.178202903	0.861428571	0.206172029	0
WBGene00014241		ZK1251.3	3.932349863	0.861428571	0.219062037	0
WBGene00020978		W03B1.9	4.993581347	0.860285714	0.172278302	0
WBGene00009213	thn-1	F28D1.3	4.060869802	0.86	0.21177729	0
WBGene00010254		F58E6.5	3.863617092	0.86	0.222589346	0
WBGene00007274		C03C11.1	3.799041827	0.86	0.22637287	0
WBGene00010324		F59C6.3	3.547941538	0.86	0.242394073	0
WBGene00011483		T05E11.7	3.400958429	0.86	0.252869895	0
WBGene00045247		F54H12.8	4.072376447	0.858809524	0.210886576	0
WBGene00007631	wht-3	C16C10.12	4.41927869	0.858571429	0.194278634	0
WBGene00013429		Y66D12A.3	4.320893307	0.858571429	0.198702298	0
WBGene00000389	cdc-25.4	R05H5.2	3.854120433	0.858214286	0.222674486	0
WBGene00004962	spe-8	F53G12.6	3.865715955	0.858095238	0.22197576	0
WBGene00044741		Y57G11C.52	4.155587916	0.857755102	0.206410048	0
WBGene00021720		Y49F6B.8	4.67927732	0.857142857	0.183178469	0
WBGene00007080	sfxn-1.1	AH6.2	4.137239057	0.855714286	0.206832207	0
WBGene00002193	kin-5	T13H10.1	4.378184605	0.855306122	0.195356341	0
WBGene00018336		F42A9.7	4.746365935	0.854285714	0.179987326	0
WBGene00044634		C29E4.14	3.487130026	0.854285714	0.244982466	0
WBGene00017056		D2062.4	3.630798332	0.853928571	0.235190306	0
WBGene00015992		C18H2.5	3.622096128	0.853571429	0.235656757	0
WBGene00008724		F13A7.1	4.163096658	0.852857143	0.20486124	0
WBGene00014121		ZK858.8	3.687386094	0.852857143	0.231290437	0
WBGene00021579	clec-73	Y46C8AL.1	3.559013195	0.852857143	0.239633038	0
WBGene00017553	nep-8	F18A12.4	3.481144369	0.852857143	0.244993328	0
WBGene00018158		F37E3.3	4.281325319	0.851428571	0.198870328	0
WBGene00018529		F47B3.5	3.752001181	0.851428571	0.22692652	0
WBGene00015994		C18H7.4	3.745299302	0.851428571	0.227332585	0
WBGene00010980		R02D5.7	3.737721368	0.851428571	0.227793484	0
WBGene00012173		W01B6.6	3.613565315	0.851428571	0.235620086	0
WBGene00008870		F15H9.1	3.274969078	0.850714286	0.25976254	0

WBGene00015855		C16C8.18	3.613938639	0.85	0.235200452	0
WBGene00009401		F35C11.2	3.246957178	0.85	0.261783557	0
WBGene00008192		C49C3.2	3.695066863	0.849642857	0.229939779	0
WBGene00018949	acbp-4	F56C9.5	4.372888671	0.848571429	0.194052832	0
WBGene00010992		R03D7.8	4.239164958	0.848571429	0.200174194	0
WBGene00018082		F36A4.3	3.523439367	0.848027211	0.240681653	0
WBGene00007572		C14A6.8	4.998461763	0.847142857	0.169480712	0
WBGene00008074	nkb-2	C43F9.6	4.443610817	0.847142857	0.190642901	0
WBGene00018121		F36H12.5	4.243235645	0.847142857	0.199645489	0
WBGene00007378		C06C3.8	3.259509817	0.847142857	0.259898851	0
WBGene00020187	gsp-4	T03F1.5	4.331343604	0.845714286	0.19525449	0
WBGene00007190	rmd-3	B0491.3	4.05357864	0.845714286	0.20863399	0
WBGene00017055		D2062.1	3.72718182	0.845714286	0.226904489	0
A_12_P171289			4.529716843	0.844285714	0.186388188	0
WBGene00013656		Y105C5B.17	4.298608405	0.842857143	0.196076745	0
WBGene00009016		F21D9.2	3.363204548	0.842857143	0.250611323	0
WBGene00009458		F36A2.11	4.405115856	0.841428571	0.191011678	0
WBGene00016010		C23G10.1	4.123663634	0.841428571	0.204048789	0
WBGene00044005		F37C12.18	4.03222536	0.84	0.20832169	0
WBGene00013148		Y53F4A.2	4.001244602	0.84	0.209934679	0
WBGene00010915		M110.7	3.650763891	0.839714286	0.230010571	0
WBGene00013586		Y80D3A.8	3.714198474	0.83952381	0.22603095	0
WBGene00044979		Y41E3.19	4.727686014	0.838571429	0.177374603	0
WBGene00017279		F09C12.8	4.315948238	0.838571429	0.194295988	0
WBGene00019151	pck-3	H04M03.1	4.208511867	0.838571429	0.199256045	0
WBGene00014174		ZK970.8	4.093777041	0.838571429	0.204840523	0
WBGene00019530	scrm-8	K08D10.7	3.784168896	0.838571429	0.221599895	0
WBGene00018083		F36A4.4	3.706125308	0.838571429	0.226266345	0
WBGene00000023	abt-5	Y53C10A.9	4.008645914	0.837619048	0.208953114	0
WBGene00007733		C25G4.6	4.495012587	0.837142857	0.186238156	0
WBGene00012636		Y38H8A.2	4.296995021	0.837142857	0.194820532	0
WBGene00019459		K06H7.8	4.226929148	0.837142857	0.198049891	0
WBGene00014032		ZK637.15	4.075830075	0.837142857	0.205391992	0
WBGene00010672		K08E7.4	3.455345414	0.837142857	0.242274724	0
WBGene00008001		C38C10.3	3.435727284	0.837142857	0.243658122	0
WBGene00012012		T25B9.6	4.176978675	0.836904762	0.200361272	0
WBGene00007641		C17E4.1	4.336492309	0.836530612	0.192904899	0
WBGene00015165		B0361.11	4.579631947	0.836394558	0.182633576	0
WBGene00009463		F36D1.4	4.047395478	0.835714286	0.206481993	0
WBGene00022707		ZK354.6	3.791798926	0.835714286	0.220400475	0
WBGene00009962		F53B6.7	3.434519704	0.835714286	0.243327847	0
WBGene00012172		W01B6.5	3.429120306	0.835714286	0.243710984	0
WBGene00006049	ssp-32	F32B6.7	5.393791636	0.834285714	0.154675184	0
WBGene00016054		C24D10.2	3.970240046	0.834285714	0.210134829	0
WBGene00013304		Y57G11C.6	3.700467257	0.834285714	0.225454154	0
WBGene00018580		F47G6.3	3.432800253	0.834285714	0.243033574	0
WBGene00012784		Y43C5A.4	4.676589379	0.832857143	0.178090714	0
WBGene00020661		T21G5.4	4.077681874	0.832857143	0.2042477	0
WBGene00009652		F43D2.3	4.014760493	0.832857143	0.207448774	0
WBGene00011132		R08A2.1	4.395190845	0.831428571	0.189167797	0
WBGene00022890	fbxa-224	ZK1290.9	4.348314343	0.831428571	0.191207099	0
WBGene00015929		C17H12.3	3.820119167	0.831428571	0.217644669	0
WBGene00010014		F54B3.2	3.608481115	0.831428571	0.230409567	0
WBGene00006039	ssp-10	K07F5.9	5.010543515	0.83	0.165650692	0
WBGene00022617		ZC477.2	3.656764933	0.83	0.22697658	0
WBGene00018745		F53C3.1	3.640182719	0.83	0.228010532	0
WBGene00018926		F56A11.6	3.399604091	0.83	0.244146076	0
A_12_P181511			3.460609963	0.829714286	0.23975955	0
WBGene00014229		ZK1128.3	4.583058028	0.828571429	0.18079008	0
WBGene00010874		M05D6.1	4.008914967	0.828571429	0.206682216	0
WBGene00018839		F54H5.2	3.666723241	0.828571429	0.225970539	0
WBGene00018980		F56F4.3	3.317067474	0.827380952	0.249431451	0
WBGene00010728		K09G1.2	4.473366233	0.827142857	0.184903899	0
WBGene00002263	lea-1	K08H10.1	3.949773005	0.827142857	0.209415289	0
WBGene00022064		Y67D8B.5	3.537605012	0.827142857	0.233814362	0

WBGene00008871	tag-314	F15H10.4	3.96600435	0.826285714	0.20834211	0
WBGene00009693		F44F1.1	3.498406707	0.82622449	0.236171652	0
WBGene00017554	nep-9	F18A12.5	3.630750701	0.825714286	0.227422468	0
WBGene00022709		ZK354.8	3.470964873	0.825714286	0.237891859	0
WBGene00010035		F54C8.1	4.581167192	0.824285714	0.179929193	0
WBGene00016312		C32D5.4	3.773341681	0.824285714	0.218449794	0
WBGene00012486		Y18D10A.21	3.963531966	0.822857143	0.207607041	0
WBGene00007300		C04F12.6	3.320676515	0.822857143	0.247798043	0
WBGene00008299		C54D10.4	3.933289047	0.821714286	0.208912764	0
WBGene00016336		C33C12.4	3.937598831	0.821428571	0.208611544	0
WBGene00019642		K11C4.1	3.465531227	0.821428571	0.237028183	0
WBGene00044392		C25H3.16	3.496507613	0.821142857	0.234846581	0
WBGene00015084		B0244.9	4.884248269	0.82	0.167886634	0
WBGene00007714		C25D7.1	4.575070377	0.82	0.179232216	0
WBGene00007249		C01G12.9	3.81383528	0.82	0.215006664	0
WBGene00020940		W02D7.4	3.711396504	0.82	0.220941093	0
WBGene00017015		D1014.2	3.263462669	0.819387755	0.251079249	0
WBGene00020105		R148.7	4.506231567	0.818571429	0.18165321	0
WBGene00018575	clec-119	F47F6.5	3.290365735	0.818571429	0.24877825	0
WBGene00020991		W03D8.10	3.272134271	0.818571429	0.250164376	0
WBGene00013478		Y69E1A.8	3.257354866	0.818	0.251124005	0
WBGene00044300		D1022.9	3.5591075	0.817714286	0.229752624	0
WBGene00014082		ZK795.2	3.658641393	0.817142857	0.223345983	0
WBGene00010241		F58D2.2	3.302947007	0.817142857	0.247398113	0
WBGene00013085	mpz-6	Y51B9A.3	4.980182259	0.816857143	0.164021536	0
WBGene00010474		K01D12.15	4.238929596	0.815714286	0.192434025	0
WBGene00007559		C14A4.8	3.886016729	0.814285714	0.209542514	0
WBGene00002227	klp-17	W02B12.7	3.859391838	0.814285714	0.21098809	0
WBGene00006058	sst-20	F54C1.9	3.277983039	0.814285714	0.248410594	0
WBGene00044476		F56D6.14	3.380626944	0.812959184	0.240475864	0
WBGene00021908		Y55B1AR.4	4.410805503	0.812857143	0.184287687	0
WBGene00016462		C35E7.10	3.472906933	0.812857143	0.234056702	0
WBGene00009550		F38H4.6	4.319349726	0.811714286	0.187925113	0
WBGene00014851		W01G7.2	5.117351325	0.811428571	0.158564171	0
WBGene00011749	ssp-36	T13F2.12	4.161052145	0.811428571	0.195005624	0
A_12_P101271			4.115501537	0.811428571	0.197163958	0
WBGene00007148		B0334.10	4.020383775	0.811428571	0.201828636	0
WBGene00007508		C10C5.4	4.148013774	0.81	0.195274183	0
WBGene00020072		R13H9.6	3.725836267	0.81	0.217400858	0
WBGene00007082	acs-10	AH10.1	3.375893145	0.81	0.239936504	0
WBGene00022006	mpst-5	Y59H11AM.[illegible]	3.105639627	0.81	0.260815837	0
WBGene00013712	dlc-6	Y106G6G.3	4.358356892	0.809795918	0.18580303	0
WBGene00017215		F07F6.1	3.490970146	0.80952381	0.231890786	0
WBGene00000748	col-175	C35B8.1	3.916978375	0.808571429	0.20642734	0
WBGene00015627		C09B9.2	3.524876353	0.808571429	0.229390012	0
WBGene00012912		Y46G5A.22	3.158094213	0.808571429	0.256031446	0
WBGene00017384		F11G11.4	3.987058492	0.807142857	0.202440686	0
WBGene00077548		F21C3.7	3.184230388	0.807142857	0.2534813	0
WBGene00007584		C14C10.1	4.279542092	0.805714286	0.188271144	0
WBGene00007888	ttr-9	C33A12.15	4.277892939	0.805714286	0.188343724	0
WBGene00010612		K07A1.5	4.264014091	0.802857143	0.1882867	0
WBGene00015696		C10H11.7	4.871500868	0.801428571	0.164513688	0
WBGene00013190		Y54E2A.5	3.67952343	0.801428571	0.21780771	0
WBGene00012638		Y38H8A.4	4.629471902	0.8	0.172805887	0
WBGene00008124		C47A4.3	4.621229288	0.8	0.173114111	0
WBGene00008313		C54G4.3	4.072117403	0.8	0.19645799	0
WBGene00018548	clec-79	F47C12.4	3.958738914	0.8	0.202084557	0
WBGene00019063		F58F12.3	3.221373617	0.798809524	0.24797171	0
WBGene00009313		F32B4.2	3.266718581	0.798571429	0.244456756	0
WBGene00018494		F46F5.2	4.429839666	0.798367347	0.180224886	0
WBGene00012207		W02B12.12	3.57391091	0.797142857	0.223044972	0
WBGene00010543		K03H1.8	3.526038338	0.797142857	0.226073225	0
WBGene00012191		W02A2.8	3.711626558	0.794857143	0.214153318	0
WBGene00022799		ZK688.4	3.280581905	0.794761905	0.242262479	0
WBGene00013524		Y73F8A.15	4.383430661	0.794285714	0.181201843	0

WBGene00013890		ZC412.9	4.052612244	0.794285714	0.195993514	0
WBGene00008382		D1081.4	3.746799855	0.794285714	0.211990431	0
WBGene00022849	acs-6	ZK1127.2	4.607002283	0.792857143	0.172098274	0
WBGene00007224		C01G6.2	4.067514881	0.792857143	0.194924214	0
WBGene00018930		F56B3.6	3.822525431	0.792857143	0.207417101	0
WBGene00001424	fis-1	F41G3.4	3.210797528	0.792857143	0.246934643	0
WBGene00010466		K01D12.7	4.053829328	0.791428571	0.19522987	0
WBGene00007269		C03C10.2	3.771719861	0.791428571	0.209832278	0
WBGene00014168		ZK945.6	3.214546582	0.791428571	0.246202241	0
WBGene00015051		B0218.7	3.964224435	0.79	0.19928236	0
WBGene00008272		C53B4.2	3.793358895	0.79	0.208258702	0
WBGene00016360		C33G8.2	3.272023486	0.79	0.24144081	0
WBGene00014752		F38E11.8	4.408433984	0.789761905	0.179147949	0
WBGene00015931		C17H12.5	4.354398796	0.788571429	0.181097659	0
WBGene00018163		F38A5.6	4.32790444	0.788571429	0.182206294	0
WBGene00011808		T16G12.7	4.10517982	0.788571429	0.192091812	0
WBGene00016083		C25A8.2	3.657311804	0.788571429	0.215615039	0
WBGene00017802		F26A1.3	3.190734754	0.788571429	0.24714415	0
WBGene00013053		Y50E8A.10	3.237398665	0.787857143	0.243361175	0
WBGene00012250		W04E12.5	4.003292916	0.787142857	0.196623848	0
A_12_P164656			3.546702046	0.787142857	0.221936562	0
WBGene00016950		C55C2.4	5.303628408	0.786857143	0.148362042	0
WBGene00009470		F36D3.4	4.376240486	0.785714286	0.17954093	0
WBGene00009321		F32B6.4	3.65594709	0.785714286	0.21491402	0
WBGene00019165	mpz-4	H06H21.9	3.551349084	0.785714286	0.22124389	0
WBGene00007986		C36F7.5	3.253601689	0.785714286	0.241490619	0
A_12_P170017			3.414938431	0.784591837	0.22975285	0
WBGene00012786		Y43C5B.3	3.613522377	0.784285714	0.217041887	0
WBGene00021878		Y54G2A.13	3.3486924	0.784285714	0.234206556	0
WBGene00013388		Y62F5A.10	3.276405092	0.784285714	0.239373854	0
WBGene00009160		F26E4.5	3.200236832	0.784285714	0.245071148	0
WBGene00013449		Y67A6A.1	4.005464596	0.783714286	0.195661269	0
WBGene00011008		R04B5.11	3.958134967	0.783571429	0.197964808	0
WBGene00012010		T25B9.4	3.512348672	0.782857143	0.222887081	0
WBGene00020177		T02H6.7	3.441487678	0.782857143	0.227476375	0
WBGene00008141		C47E8.1	3.23093575	0.782857143	0.242300437	0
A_12_P164040			3.247292223	0.782285714	0.240904009	0
WBGene00016587		C42C1.9	3.631275912	0.781428571	0.21519394	0
WBGene00013651		Y105C5B.11	3.236216994	0.780571429	0.24119873	0
WBGene00007633		C16D2.1	4.72366662	0.778571429	0.164823535	0
WBGene00006509	tag-164	Y76A2A.1	4.347034163	0.778571429	0.179104051	0
WBGene00005650	srp-9	F09C6.5	3.491554277	0.778571429	0.222987062	0
WBGene00016181		C28C12.11	4.266073863	0.775714286	0.181833299	0
WBGene00021633		Y47G6A.3	4.169658572	0.775714286	0.186037843	0
WBGene00050940		C25D7.16	3.334164109	0.775238095	0.232513479	0
WBGene00013579		Y79H2A.2	3.947118171	0.774285714	0.196164817	0
WBGene00012297		W06D4.3	3.764168494	0.774285714	0.205699005	0
WBGene00021537		Y42H9AR.2	3.46494613	0.774285714	0.223462555	0
WBGene00016963		C56C10.6	3.434337762	0.774285714	0.225454154	0
WBGene00019257	dhhc-13	H32C10.3	3.250465587	0.774285714	0.238207633	0
WBGene00045306		ZC250.5	4.017740793	0.772857143	0.192361126	0
A_12_P171288			3.478262305	0.772857143	0.222196337	0
WBGene00044434		Y18H1A.15	3.275600172	0.772857143	0.235943675	0
WBGene00016053		C24D10.1	3.260196897	0.772857143	0.237058425	0
WBGene00010114		F55D12.6	3.868294737	0.771428571	0.199423421	0
WBGene00007239		C01G10.14	3.537220101	0.771428571	0.218088937	0
WBGene00017559	mpz-3	F18C5.4	4.149491032	0.769142857	0.185358361	0
WBGene00019512		K08A2.2	3.77772702	0.768571429	0.203448112	0
A_12_P164654			3.501659511	0.768571429	0.219487767	0
WBGene00012180		W01D2.3	4.17080647	0.767142857	0.18393154	0
WBGene00017387	mpst-4	F11G11.9	3.156720454	0.767142857	0.24301894	0
WBGene00020293	nep-20	T06D4.4	3.393142603	0.766785714	0.225980987	0
WBGene00015097		B0273.1	3.862272752	0.765714286	0.198254845	0
WBGene00016212		C29F5.3	3.221961931	0.765714286	0.237654666	0
WBGene00013299	irld-18	Y57G11B.7	3.153811463	0.765714286	0.242790127	0

WBGene00009605		F40G12.10	3.144035447	0.765714286	0.243545055	0
WBGene00022385		Y95B8A.4	3.492317777	0.764285714	0.2188477	0
WBGene00021447		Y39A3CR.8	4.416269295	0.761142857	0.172349738	0
WBGene00017808	nspd-9	F26A1.10	3.694677781	0.76	0.205701294	0
WBGene00012877		Y45F10C.1	4.994040021	0.75877551	0.151936209	0
WBGene00020898		T28D9.9	3.551677393	0.758707483	0.213619481	0
WBGene00017550	nep-6	F18A12.1	3.727680384	0.758571429	0.203496907	0
WBGene00018999		F57B9.8	3.309899611	0.758571429	0.229182609	0
WBGene00016398		C34D4.2	3.205997044	0.758	0.236431909	0
WBGene00016420		C34G6.3	4.054638968	0.757142857	0.186734963	0
WBGene00011120		R07E5.15	3.722152404	0.755714286	0.203031527	0
WBGene00022735		ZK418.6	3.620021245	0.755714286	0.208759627	0
WBGene00018004		F33D11.7	3.480688921	0.755714286	0.217116296	0
A_12_P164657			3.112788015	0.755714286	0.242777305	0
WBGene00021608		Y46H3D.1	3.967373165	0.754571429	0.190194216	0
WBGene00011438		T04F3.3	3.701764473	0.754285714	0.203763832	0
WBGene00021787		Y51H7C.9	3.40728555	0.754285714	0.221374377	0
WBGene00016807		C50D2.3	4.789141856	0.752857143	0.157200844	0
WBGene00015661		C09H5.7	4.367754586	0.752857143	0.172367089	0
WBGene00006053	ssq-4	T28H11.1	3.376881236	0.752857143	0.222944513	0
WBGene00013310		Y57G11C.14	3.227716511	0.752857143	0.233247604	0
WBGene00015345		C02F5.2	4.95540771	0.752585034	0.151871466	0
WBGene00044261		Y87G2A.20	3.168969674	0.751428571	0.237120783	0
WBGene00017647		F20H11.4	4.332916127	0.75	0.173093588	0
WBGene00011919		T22C1.9	4.015879907	0.75	0.186758573	0
WBGene00002186	kel-10	T16H12.6	3.882171142	0.75	0.193190865	0
WBGene00016441		C35D10.3	3.672510741	0.75	0.204219961	0
WBGene00021792		Y52D5A.1	3.518306462	0.749714286	0.213089534	0
WBGene00017050		D2024.1	3.528216527	0.749285714	0.212369538	0
WBGene00013854	cyc-2.2	ZC116.2	3.73525654	0.748571429	0.200406966	0
WBGene00010265		F58G1.3	3.478102973	0.748571429	0.215224056	0
WBGene00014154		ZK930.4	3.115914499	0.748571429	0.240241325	0
A_12_P171287			3.209627049	0.747380952	0.232856011	0
WBGene00015210		B0496.1	3.416777432	0.747142857	0.218668869	0
WBGene00016322		C32E8.4	4.198827307	0.745714286	0.177600609	0
WBGene00016956		C55C3.6	3.64752286	0.745714286	0.204444034	0
WBGene00007335		C05C12.1	3.106977707	0.745714286	0.240012757	0
WBGene00007457		C08F11.10	3.797169695	0.744285714	0.196010654	0
A_12_P101810			3.501815837	0.744285714	0.212542792	0
WBGene00012307		W06F12.3	3.527879722	0.742857143	0.210567593	0
WBGene00044896		K12C11.5	3.39306603	0.742857143	0.218933889	0
WBGene00012535		Y37A1A.2	3.156657947	0.742857143	0.235330262	0
WBGene00008312		C54G4.2	3.918302811	0.741428571	0.189221867	0
WBGene00012102		T27F6.1	4.604601242	0.741102041	0.160948148	0
WBGene00016388		C34B2.3	3.671697574	0.74	0.201541653	0
WBGene00044127		BE10.5	3.612972523	0.74	0.2048175	0
WBGene00010719		K09E4.1	3.371808068	0.74	0.219466822	0
WBGene00021472		Y39G10AR.1	3.298215624	0.74	0.224363742	0
WBGene00020533		T16A1.2	3.297353267	0.738367347	0.22392728	0
WBGene00009492		F36H1.3	3.986632307	0.738333333	0.185202265	0
WBGene00015944		C18A3.7	3.431423155	0.737142857	0.214821322	0
WBGene00013710		Y106G6G.1	3.255368241	0.737142857	0.226439162	0
WBGene00009129		F25H5.7	3.565294206	0.735714286	0.206354439	0
WBGene00000878	cyn-2	ZK520.5	3.642493717	0.734285714	0.201588739	0
WBGene00019084		F59A6.2	3.250402655	0.734285714	0.225906078	0
WBGene00009759	ttr-12	F46B3.4	3.31938721	0.731428571	0.220350482	0
WBGene00011322	irld-14	T01C3.5	3.234814188	0.731428571	0.226111464	0
WBGene00016946		C55B7.10	3.781387292	0.73	0.193050842	0
WBGene00011241	mpz-5	R11A8.8	3.294317604	0.73	0.221593692	0
WBGene00021639		Y47G6A.13	3.416930385	0.728571429	0.21322396	0
WBGene00017922		F29B9.7	3.396114019	0.727142857	0.21411026	0
WBGene00022650		ZK84.2	3.835464651	0.725714286	0.189211569	0
WBGene00010082		F55A11.11	3.722681666	0.725714286	0.194943955	0
A_12_P114047			3.173934842	0.725714286	0.228648136	0
WBGene00007597		C15A11.2	3.423556414	0.724285714	0.211559451	0

WBGene00011191		R10D12.10	3.214176595	0.724285714	0.225340983	0
WBGene00021428		Y38F2AR.10	3.209344456	0.724285714	0.225680267	0
WBGene00013475		Y69E1A.3	3.400702064	0.724047619	0.212911218	0
WBGene00020174		T02H6.4	3.349629774	0.723428571	0.215972696	0
WBGene00017853		F27C1.3	3.565183648	0.72	0.201953131	0
WBGene00016085		C25A8.5	3.11512657	0.72	0.231130256	0
WBGene00013657		Y105C5B.18	3.845921402	0.718571429	0.186839863	0
WBGene00017175	irld-3	F02C9.4	3.338791786	0.717142857	0.214791129	0
WBGene00010300		F59A1.15	3.375340146	0.71547619	0.211971582	0
A_12_P164658			4.031750262	0.714285714	0.177165168	0
WBGene00001605	gln-4	T25C8.3	3.192535839	0.712857143	0.223288689	0
WBGene00021967		Y57G7A.3	3.431680505	0.712653061	0.207668826	0
WBGene00008235		C50F4.10	3.692759493	0.711428571	0.192654998	0
WBGene00019081		F59A3.8	3.149897143	0.711428571	0.225857715	0
WBGene00023490		F36A2.14	3.382816192	0.707142857	0.209039693	0
WBGene00007863		C32C4.3	3.147028823	0.705714286	0.224247799	0
WBGene00019810		R01H2.2	3.651549621	0.704285714	0.192873105	0
WBGene00010184		F57A10.2	4.295071673	0.701428571	0.163310097	0
WBGene00009590	ttr-4	F40F12.1	3.287977297	0.7	0.212896847	0
WBGene00016730		C46H11.6	3.806789521	0.698367347	0.183453102	0
WBGene00013979		ZK507.3	4.331173947	0.697142857	0.16095933	0
WBGene00014005		ZK593.9	3.260170325	0.695714286	0.213398141	0
WBGene00016778	nep-3	C49D10.10	3.143116243	0.695714286	0.221345388	0
WBGene00008155		C47E12.12	3.930766181	0.694285714	0.176628597	0
WBGene00009416		F35E2.9	3.626549138	0.694285714	0.19144528	0
WBGene00012627		Y38H6C.15	3.182758249	0.694285714	0.218139632	0
WBGene00012169		W01B6.2	3.149068577	0.694285714	0.220473355	0
WBGene00015241		B0524.2	4.827659895	0.692857143	0.143518218	0
WBGene00010371		H06O01.4	4.023036841	0.692857143	0.172222421	0
WBGene00007563		C14A4.13	3.681218268	0.692857143	0.188214089	0
WBGene00050956		C14A6.13	3.569725406	0.692857143	0.19409256	0
A_12_P164042			3.272412582	0.692857143	0.211726708	0
WBGene00004964	spe-10	AC3.10	3.158117315	0.692857143	0.219389299	0
WBGene00022080			3.432644755	0.691836735	0.201546267	0
WBGene00018394		F43E2.6	3.674793254	0.691428571	0.188154414	0
WBGene00010763		K10H10.7	3.681108352	0.691190476	0.187766947	0
WBGene00016320		C32E8.1	3.477074416	0.690979592	0.198724419	0
WBGene00021006	dct-9	W03F11.3	3.727808226	0.69	0.185095358	0
WBGene00000751	col-178	C34F6.2	3.442545695	0.69	0.200433069	0
WBGene00022530		ZC155.2	3.201278863	0.689142857	0.215271111	0
WBGene00021175	math-46	Y8G1A.1	3.974531023	0.688571429	0.173245956	0
WBGene00008854		F15D3.4	4.233523515	0.685952381	0.162028716	0
WBGene00022108		Y71F9AL.2	3.536999257	0.685714286	0.193868937	0
A_12_P164043			3.168289764	0.685714286	0.21643042	0
WBGene00012979		Y48B6A.5	4.157311204	0.684897959	0.164745415	0
WBGene00022085		Y69A2AR.14	3.577938008	0.684761905	0.191384508	0
WBGene00014053		ZK669.3	3.504977193	0.681428571	0.194417405	0
WBGene00012741		Y40H4A.2	3.68454226	0.68	0.184554811	0
WBGene00009043		F22B5.5	3.295264664	0.68	0.206356718	0
WBGene00004906	snf-7	ZK1010.9	3.647983984	0.679285714	0.186208524	0
WBGene00021880		Y54G2A.15	4.369504728	0.678571429	0.155297104	0
WBGene00044529	irld-1	D2063.4	4.077875807	0.678571429	0.166403162	0
WBGene00012720		Y39E4B.11	3.225433428	0.675714286	0.209495654	0
WBGene00009749		F46A8.6	3.176446565	0.675714286	0.212726477	0
WBGene00022700		ZK353.4	3.455382717	0.675	0.195347391	0
WBGene00012138		T28F4.3	3.527965832	0.674285714	0.19112592	0
WBGene00000696	col-122	T05A1.2	3.455198907	0.674285714	0.195151056	0
WBGene00008651		F10D11.5	3.105855891	0.674285714	0.217101417	0
WBGene00018196	nep-13	F39E9.4	3.257200175	0.672857143	0.206575312	0
WBGene00018165		F38A5.8	3.21783625	0.672857143	0.209102357	0
WBGene00007570		C14A6.6	3.859194256	0.671428571	0.173981543	0
WBGene00019626		K10C9.7	3.788331419	0.671428571	0.177235964	0
WBGene00009075		F23B2.7	3.664031378	0.671428571	0.183248587	0
WBGene00006624	try-6	F48A9.3	3.96900371	0.671071429	0.169078055	0
A_12_P159830			3.898153451	0.67	0.171876251	0

WBGene00012296	spe-46	W06D4.2	3.91960882	0.668571429	0.170570957	0
A_12_P108193			4.355519616	0.667142857	0.153171818	0
WBGene00010636		K07F5.8	3.679096933	0.667142857	0.181333319	0
WBGene00013886		ZC412.5	4.789442596	0.665714286	0.138996193	0
WBGene00001839	hdl-1	ZK829.2	4.352436801	0.665714286	0.152952085	0
A_12_P136854			3.622265263	0.665714286	0.183783969	0
WBGene00050916		F55F10.3	3.718734796	0.662857143	0.178248028	0
WBGene00050912		F43E2.12	4.346691163	0.661428571	0.152168292	0
A_12_P136851			3.912125456	0.661428571	0.169071411	0
WBGene00021154		Y4C6A.4	3.384497801	0.660408163	0.195127373	0
WBGene00012402	sfxn-1.3	Y6E2A.9	3.781768565	0.66	0.17452152	0
WBGene00022288		Y75B7B.1	3.124671776	0.66	0.211222185	0
WBGene00019431		K06A5.3	4.425014865	0.658571429	0.148829201	0
WBGene00003839	ocr-2	T09A12.3	4.176307284	0.658571429	0.157692283	0
WBGene00022761		ZK546.4	3.51463121	0.657142857	0.186973488	0
WBGene00013894		ZC434.8	3.316761362	0.655714286	0.197697155	0
WBGene00010538	ttr-3	K03H1.3	3.134865589	0.654285714	0.208712526	0
WBGene00009459		F36A2.12	3.736626873	0.652857143	0.174718313	0
WBGene00012120		T28C6.5	3.381988484	0.651632653	0.192677372	0
WBGene00011297		R102.10	4.020675467	0.650238095	0.161723596	0
WBGene00008493		F01D5.2	3.646489305	0.65	0.178253642	0
WBGene00000707	col-133	F52B11.4	3.139947978	0.648571429	0.206554832	0
WBGene00018147		F37C4.7	3.549375195	0.647142857	0.182325852	0
A_12_P136850			3.391992793	0.647142857	0.190785446	0
WBGene00008153		C47E12.10	3.342592189	0.647142857	0.193605089	0
WBGene00016177		C28C12.1	3.342105101	0.645714286	0.193205859	0
A_12_P136852			3.361957186	0.644285714	0.191640071	0
WBGene00021454		Y39F10A.3	3.260349587	0.644285714	0.197612464	0
WBGene00001538	gcy-12	F08B1.2	3.239884055	0.644285714	0.198860732	0
WBGene00022699		ZK353.3	3.546600483	0.642285714	0.181098976	0
WBGene00015685		C10G11.1	3.668815029	0.641714286	0.174910504	0
WBGene00017737		F23C8.8	4.029384425	0.637142857	0.158124118	0
WBGene00007791		C28A5.6	3.856169144	0.637142857	0.165226896	0
WBGene00010682		K08F8.5	3.304074058	0.637142857	0.192835525	0
WBGene00019555		K09C6.1	3.829148077	0.635714286	0.166019771	0
WBGene00022634		ZC581.7	3.517689721	0.632857143	0.179907039	0
WBGene00045484		F34D10.9	4.56233889	0.631428571	0.13840019	0
WBGene00011777		T14G10.8	4.239683357	0.631428571	0.148932955	0
WBGene00013684		Y105E8A.27	3.239544644	0.631190476	0.194839258	0
WBGene00012961		Y47H10A.5	3.288409003	0.631088435	0.191913	0
WBGene00012827		Y43F8C.5	3.301742461	0.63	0.190808341	0
WBGene00000615	col-38	F54C9.4	4.004155892	0.628571429	0.156979759	0
WBGene00010712		K09B11.5	3.470581091	0.623571429	0.179673493	0
WBGene00019980	chil-14	R09B5.12	3.90226454	0.62	0.158882104	0
WBGene00018120		F36H12.4	3.832719493	0.62	0.161765034	0
WBGene00010155		F56F3.4	3.156440015	0.62	0.196423818	0
WBGene00014085		ZK809.1	3.099460662	0.618571429	0.199573892	0
WBGene00007446	mboa-4	C08F8.4	3.168594224	0.617142857	0.194768662	0
WBGene00018065		F35F11.2	3.666569349	0.615714286	0.167926535	0
WBGene00044763		ZK688.12	3.95177577	0.61	0.154360985	0
WBGene00021010		W03G1.2	3.176882414	0.608367347	0.191498226	0
WBGene00044298		T23B7.2	3.625092447	0.604285714	0.166695256	0
WBGene00044263		Y105C5A.26	3.742736867	0.602857143	0.161073878	0
WBGene00044312		K04C2.8	3.216741716	0.601428571	0.186968251	0
WBGene00010991		R03D7.5	3.125064489	0.6	0.191996038	0
WBGene00007898		C33D9.3	-4.197315351	-0.631836735	0.150533539	0
WBGene00022485	fbxa-63	Y119D3B.7	-4.46685983	-0.638571429	0.14295757	0
WBGene00016996		D1005.2	-4.36120535	-0.691428571	0.158540705	0
WBGene00019426	cutl-16	K06A1.3	-4.354833834	-0.704	0.16165944	0
WBGene00045102		F52B11.8	-5.195783497	-0.705714286	0.135824421	0
WBGene00023500		C11H1.9	-5.559873068	-0.741428571	0.133353507	0
WBGene00044660	fbxa-87	F44G3.13	-4.718137882	-0.746496599	0.158218479	0
WBGene00020902	jmjc-1	T28F2.4	-5.405434111	-0.765714286	0.141656391	0
WBGene00011760		T13H5.6	-4.489824242	-0.784285714	0.174680716	0
WBGene00016659		C45B2.2	-5.275230585	-0.785714286	0.148944065	0

WBGene00045246		C29E4.15	-4.422384752	-0.802857143	0.181543938	0
WBGene00008971		F20C5.6	-4.467902096	-0.821428571	0.183851068	0
WBGene00013866	cbs-1	ZC373.1	-5.017825616	-0.822857143	0.163986795	0
WBGene00020258		T05E7.1	-4.586196834	-0.842857143	0.183781284	0
WBGene00022416		Y102A11A.6	-4.918641506	-0.851428571	0.173102384	0
WBGene00020673		T22B7.4	-4.289388362	-0.878571429	0.204824407	0
WBGene00014254	cyp-13A10	ZK1320.4	-4.945422703	-0.937142857	0.189497018	0
WBGene00011437		T04F3.2	-4.404735138	-1.134285714	0.257515078	0
WBGene00013007		Y48E1B.8	-4.369157492	-1.472857143	0.337103239	0
