# Supplementary material for: Transcription factors CEP‐1/p53 and CEH‐23 collaborate with AAK‐2/AMPK to modulate longevity in Caenorhabditis elegans
Source: Aging Cell. 2017 May 30;16(4):814–24. doi: 10.1111/acel.12619 (PMC5506430; doi:10.1111/acel.12619)
Supplement: Supplementary file 7 — Table S4 Genes that are differentially expressed between aak‐2ca strain and transgenic control strain. (Data from Mair et al. and reanalyzed using SAM analysis. Gene list was identified by SAM 1 class analysis with FDR = 0, 1.5 fold change cutoff.) [file ACEL-16-814-s007.pdf]

Table S4: Genes differentially expressed in aak-2ca vs transgenic control (identified by 1 class SAM with FDR=0, 1.5 fold change cutoff)

Gene ID	Gene Name	Score(d)	Numerator(r)	enominator(s+s	q-value(%)
WBGene00000657	col-81	12.25039704	5.572110514	0.454851422	0
WBGene00000608	col-19	13.46144529	5.364704131	0.398523637	0
WBGene00017806	F26A1.8	13.07041312	5.156815181	0.394541101	0
WBGene00006929	vit-5	17.93614245	4.891221059	0.272701952	0
WBGene00010065	F54F7.3	20.82933796	4.803742219	0.230623855	0
WBGene00000703	col-129	12.89518259	4.567913511	0.354234109	0
WBGene00008681	scrm-4	26.39683083	4.525484457	0.171440446	0
WBGene00006928	vit-4	16.25202468	4.250752213	0.261552163	0
WBGene00000712	col-139	9.11342982	4.035501893	0.442808248	0
WBGene00007823	C30H6.5	12.31739547	4.025535198	0.326817078	0
WBGene00000024	abu-1	10.81008146	3.866165578	0.357644444	0
WBGene00006926	vit-2	15.5613926	3.67004348	0.235842869	0
WBGene00000713	col-140	8.26335824	3.626369206	0.438849327	0
WBGene00021083	W08E12.2	9.204930611	3.551575666	0.385834051	0
WBGene00000663	col-88	12.2107401	3.502748789	0.286858025	0
WBGene00020223	T05A7.6	39.56605307	3.486582476	0.088120553	0
WBGene00019084	F59A6.2	10.71435746	3.407912782	0.31806973	0
WBGene00000729	col-156	9.016218921	3.351377273	0.371705401	0
WBGene00018031	F35B3.4	11.29938811	3.32009915	0.293829995	0
WBGene00016196	C28H8.5	10.62829108	3.300790236	0.310566413	0
WBGene00021084	W08E12.3	19.43769244	3.291844393	0.169353662	0
WBGene00012880	Y45F10C.4	16.33975426	3.289075778	0.201292855	0
WBGene00017743	F23F1.2	9.083536364	3.182732404	0.350384726	0
WBGene00016434	C35B1.4	12.64249551	3.124551819	0.247146761	0
WBGene00016596	C42D4.3	12.13020271	3.120590286	0.257257884	0
WBGene00015987	C18G1.9	11.99930009	3.107788259	0.258997461	0
WBGene00015690	C10G11.10	23.84282219	3.102573611	0.130126106	0
WBGene00017736	F23C8.7	19.64642934	3.091239106	0.157343559	0
WBGene00020670	T22B2.6	9.362135979	3.077498912	0.328717604	0
WBGene00007398	C07A4.3	15.15537403	3.061420495	0.202002306	0
WBGene00021085	W08E12.4	28.52127935	3.055412551	0.107127472	0
WBGene00019307	C17B7.13	17.01183763	3.052118197	0.179411435	0
WBGene00008660	clec-153	14.80472005	3.03668572	0.205116051	0
WBGene00015331	C02B10.6	30.24875777	3.004346107	0.099321305	0
WBGene00020413	T10E9.3	13.87157094	2.97654929	0.214579106	0
WBGene00022530	ZC155.2	24.15526502	2.973083238	0.123082203	0
WBGene00000694	col-120	12.51960995	2.968945632	0.237143621	0
WBGene00010084	F55B11.2	11.37369337	2.966007631	0.260777879	0
WBGene00006930	vit-6	23.03236466	2.956383307	0.128357785	0
WBGene00004099	pqn-5	8.403443517	2.927045994	0.348315067	0
WBGene00009115	F25F2.1	24.24579049	2.905070196	0.119817508	0
WBGene00020533	T16A1.2	17.32738243	2.894789348	0.167064435	0
WBGene00007354	C06A1.3	25.06085943	2.865220219	0.114330485	0
WBGene00000710	col-137	9.121274084	2.858686382	0.31340867	0
WBGene00007305	C04G2.2	29.11351791	2.853209051	0.098002895	0
WBGene00014187	ZK1025.7	39.61152591	2.842922933	0.071770094	0
WBGene00010509	K02E2.8	14.97047415	2.835508774	0.189406745	0
WBGene00020033	R12E2.7	10.06001541	2.834625383	0.281771475	0
WBGene00017073	D2096.6	11.79877909	2.833445294	0.24014733	0
WBGene00006927	vit-3	11.51743431	2.831116488	0.245811386	0
WBGene00000636	col-60	10.40935461	2.820486967	0.270956949	0
WBGene00018783	F54A3.4	21.17160199	2.79123503	0.131838631	0
WBGene00002204	kin-21	41.64440459	2.78209449	0.066805962	0
WBGene00012115	T28B8.4	25.51218402	2.779777296	0.108958813	0
WBGene00022689	math-47	30.75046029	2.779361841	0.090384398	0

WBGene00016327	C32E12.1	43.63611005	2.775117823	0.06359682	0
WBGene00017213	F07E5.8	20.7501438	2.76720875	0.133358534	0
WBGene00000034	abu-11	9.315170429	2.724962049	0.292529489	0
WBGene00013644	Y105C5A.25	18.90579122	2.716869618	0.143705682	0
WBGene00018999	F57B9.8	26.79420249	2.700253634	0.100777533	0
WBGene00022596	ZC395.4	17.97315333	2.69443636	0.149914504	0
WBGene00017672	F21F3.2	18.51445064	2.689570979	0.145268743	0
WBGene00000680	col-106	8.636284742	2.68481364	0.310875998	0
WBGene00009324	F32B6.10	28.23606265	2.676913401	0.094804769	0
WBGene00001703	grd-14	15.75717828	2.669118366	0.169390631	0
WBGene00016636	C44B12.1	10.3939906	2.645108063	0.254484362	0
WBGene00007179	B0457.4	15.12512783	2.635409866	0.174240502	0
WBGene00021639	Y47G6A.13	14.30169036	2.635043216	0.184246977	0
WBGene00016638	C44B12.5	11.31112966	2.62703676	0.232252378	0
WBGene00022707	ZK354.6	18.14755875	2.624375377	0.144613136	0
WBGene00012547	Y37D8A.5	40.95709825	2.620655609	0.063985383	0
WBGene00008590	F08H9.2	16.41211088	2.608802201	0.158955921	0
WBGene00021625	Y47D7A.13	11.0855922	2.605678442	0.235050902	0
WBGene00010634	K07F5.6	21.37678063	2.597262209	0.121499222	0
WBGene00014183	ZK1025.3	14.68617592	2.584567245	0.175986401	0
WBGene00006047	ssp-19	12.23916884	2.578368307	0.210665311	0
WBGene00006044	ssp-16	18.67662378	2.568480663	0.137523821	0
WBGene00012138	T28F4.3	10.56934708	2.557620824	0.241984751	0
WBGene00015049	B0218.5	10.61101688	2.555840093	0.24086665	0
WBGene00012102	T27F6.1	11.4084221	2.551429579	0.223644388	0
WBGene00019409	K05F1.8	18.7350543	2.546631092	0.135928674	0
WBGene00018123	F36H12.9	11.13729144	2.537478197	0.227836203	0
WBGene00009549	F38H4.5	15.90371581	2.529977762	0.159080921	0
WBGene00019717	M01H9.1	11.99462158	2.521827617	0.210246534	0
WBGene00022108	Y71F9AL.2	18.53031956	2.521043037	0.136049626	0
WBGene00011200	R10E4.3	18.55920235	2.513206047	0.135415628	0
WBGene00008125	C47A4.5	19.48642343	2.502990028	0.128447893	0
WBGene00015241	B0524.2	9.251129662	2.502743549	0.270533831	0
WBGene00018178	F38E1.3	17.77625613	2.500425007	0.140660946	0
WBGene00013437	Y66D12A.11	8.787658581	2.49518294	0.283941725	0
WBGene00016085	C25A8.5	10.42266185	2.494325049	0.239317468	0
WBGene00022679	ZK180.5	10.32945585	2.492795591	0.241328839	0
WBGene00000754	col-181	8.965814825	2.490878289	0.277819511	0
WBGene00000696	col-122	9.47032752	2.474326187	0.261271448	0
WBGene00021007	W03F11.4	29.10267311	2.472988509	0.084974617	0
WBGene00009185	F27C8.5	13.25951	2.472166902	0.186444816	0
WBGene00021993	Y59E9AL.2	18.76106336	2.471231302	0.131721281	0
WBGene00010486	K01H12.4	11.54748762	2.446905649	0.211899396	0
WBGene00020580	T19D12.5	26.37660301	2.433543572	0.092261447	0
WBGene00011910	T22B3.2	17.38926119	2.430246847	0.139755612	0
WBGene00019216	H20J04.1	20.39190458	2.422965553	0.118819973	0
WBGene00012689	Y39B6A.30	14.46644123	2.421473952	0.1673856	0
WBGene00001736	grl-27	9.284661895	2.417215927	0.260345067	0
WBGene00010014	F54B3.2	16.65471388	2.416501977	0.145094175	0
WBGene00009149	F26D2.10	10.42749417	2.410926184	0.231208586	0
WBGene00006954	wrt-8	10.07817008	2.409506889	0.239081785	0
WBGene00009492	F36H1.3	12.23597955	2.406809092	0.196699339	0
WBGene00010092	F55C5.2	11.89021413	2.40504728	0.202271149	0
WBGene00018004	F33D11.7	8.475221111	2.404553704	0.283715749	0
WBGene00009401	F35C11.2	16.27248586	2.400666489	0.14752918	0
WBGene00012827	Y43F8C.5	17.67677271	2.400090634	0.135776517	0
WBGene00010679	K08F4.5	23.35850666	2.396446343	0.102594159	0

WBGene00021207	Y18H1A.1	14.86946634	2.395441214	0.161097995	0
	yk582c5	9.459637642	2.394951829	0.253175853	0
WBGene00006048	ssp-31	11.10641651	2.38577467	0.214810481	0
WBGene00013526	Y73F8A.20	44.35143652	2.381129936	0.053687775	0
WBGene00010510	ent-3	23.41432318	2.376778334	0.10150959	0
WBGene00011171	C17G10.3	12.68506616	2.374083537	0.187155787	0
WBGene00018001	F33D11.2	20.95354647	2.364380575	0.11283916	0
WBGene00008001	C38C10.3	21.73468337	2.35534564	0.108368068	0
WBGene00018563	F47D12.7	27.12969188	2.35406411	0.086770765	0
WBGene00003497	mup-4	8.258437748	2.353198132	0.284944708	0
WBGene00009897	F49E12.1	20.24196849	2.349573174	0.116074342	0
WBGene00018573	oac-30	9.107594601	2.347161251	0.257714726	0
WBGene00009075	F23B2.7	21.43903085	2.345260801	0.109392109	0
WBGene00018347	F42C5.5	8.516367133	2.340870031	0.274867205	0
WBGene00016130	C26B2.8	9.680115792	2.340458212	0.241779981	0
WBGene00016416	C34F11.5	9.656631727	2.337756974	0.242088239	0
WBGene00011260	R13G10.4	14.69119426	2.336593265	0.159047197	0
WBGene00013858	ZC168.6	20.62740565	2.323768205	0.11265441	0
WBGene00007230	C01G10.1	14.36376041	2.3174747	0.161341782	0
WBGene00011530	T06D8.10	12.73913818	2.316486185	0.181840102	0
WBGene00019812	R01H2.4	12.68995132	2.315355404	0.182455814	0
WBGene00019086	F59A6.4	9.176266521	2.31437405	0.252213037	0
WBGene00013712	dlc-6	15.24233483	2.314189706	0.151826458	0
WBGene00010366	H05L14.1	14.92110984	2.312723004	0.154996715	0
WBGene00007049	tag-191	10.65805979	2.31057087	0.216790947	0
WBGene00000757	col-184	8.172799375	2.303135132	0.281804927	0
WBGene00017393	F12A10.4	26.72390802	2.300919079	0.086099648	0
WBGene00003562	ncr-2	24.35139357	2.299632487	0.094435355	0
WBGene00016953	C55C3.3	16.98145321	2.298054811	0.135327335	0
WBGene00014154	ZK930.4	17.64252341	2.286062455	0.129576841	0
WBGene00022771	ZK616.1	21.57940654	2.279791952	0.105646647	0
WBGene00017441	F13H8.8	15.75262354	2.278763543	0.144659303	0
WBGene00007597	C15A11.2	9.3038511	2.272738715	0.244279352	0
WBGene00015929	C17H12.3	10.91505064	2.270745963	0.20803806	0
WBGene00007806	clec-230	14.53062504	2.269542723	0.156190303	0
WBGene00006449	tag-76	46.44112248	2.260984398	0.048684964	0
WBGene00014201	ZK1053.6	22.04054097	2.260833203	0.102576121	0
WBGene00008950	wht-5	16.93446877	2.245995252	0.132628622	0
WBGene00010254	F58E6.5	16.38182945	2.236926767	0.136549265	0
WBGene00010906	M88.3	31.98843536	2.236788859	0.069924922	0
WBGene00015034	B0207.11	14.64423938	2.233614655	0.15252514	0
WBGene00021878	Y54G2A.13	13.39531659	2.231211367	0.166566527	0
WBGene00018531	F47B3.7	12.98581143	2.230141195	0.171736761	0
WBGene00011790	T15H9.5	18.46771672	2.218217637	0.120113259	0
WBGene00007639	C17D12.5	18.45707157	2.214760516	0.119995228	0
WBGene00009129	F25H5.7	16.31079826	2.204724194	0.135169607	0
WBGene00018196	F39E9.4	12.47123463	2.200752145	0.176466261	0
WBGene00017050	D2024.1	19.131586	2.198040548	0.11489066	0
WBGene00016698	C46A5.1	13.59617507	2.194543688	0.161408902	0
WBGene00013053	Y50E8A.10	13.19097419	2.191849435	0.166162817	0
WBGene00016963	C56C10.6	16.78622447	2.191389549	0.130546899	0
WBGene00020039	R12E2.14	13.85879289	2.190341338	0.15804705	0
WBGene00014179	ZK1010.5	15.90295343	2.186120864	0.137466344	0
WBGene00015765	C14C11.1	9.741866137	2.176193582	0.2233857	0
WBGene00016054	C24D10.2	12.11467906	2.171975167	0.179284582	0
WBGene00017542	F17E9.5	10.20954639	2.166318591	0.212185587	0
WBGene00017354	F10E9.2	14.70815724	2.165607112	0.147238507	0

WBGene00017815	F26B1.1	28.67709058	2.156738087	0.075207702	0
WBGene00012011	T25B9.5	22.24678715	2.15277361	0.096767843	0
WBGene00015500	C06A5.2	14.57573199	2.148579267	0.147407984	0
WBGene00016752	C48E7.7	16.99297063	2.144206083	0.126181945	0
WBGene00010920	M117.4	9.594150713	2.1432386	0.223390133	0
WBGene00020686	T22D1.8	13.135702	2.136664316	0.16266084	0
WBGene00012679	Y39B6A.18	15.08586493	2.136434413	0.141618291	0
WBGene00007489	C09G5.7	25.55793412	2.13505199	0.083537737	0
WBGene00016954	C55C3.4	14.93896591	2.134496385	0.142881134	0
WBGene00010467	K01D12.8	9.473388034	2.129479472	0.224785416	0
WBGene00018528	F47B3.4	14.77525798	2.12806429	0.144028909	0
WBGene00015215	B0496.6	24.82140493	2.125300659	0.085623705	0
WBGene00009160	F26E4.5	12.15166447	2.121191876	0.17455978	0
WBGene00012172	W01B6.5	9.362702863	2.121027777	0.226540114	0
WBGene00013085	Y51B9A.3	18.45229903	2.117373285	0.114748481	0
WBGene00008124	C47A4.3	20.49597937	2.116591577	0.103268624	0
WBGene00012871	Y45F10B.3	15.30624719	2.115120595	0.138186753	0
WBGene00014116	ZK858.2	30.53771206	2.109368221	0.069074206	0
WBGene00007610	C15H7.3	12.18101432	2.108083622	0.173063061	0
WBGene00011501	rmd-1	14.04660445	2.107411722	0.150029976	0
WBGene00022229	Y73B6A.2	14.1201622	2.10735543	0.149244421	0
WBGene00016053	C24D10.1	14.16605713	2.105840318	0.148653948	0
WBGene00008312	C54G4.2	15.11573182	2.105124685	0.139267136	0
WBGene00010468	K01D12.9	12.7652674	2.104329387	0.164848046	0
WBGene00017802	F26A1.3	8.647502987	2.104118643	0.24332095	0
WBGene00009031	F21H7.5	29.78160814	2.103879913	0.070643597	0
WBGene00017804	F26A1.6	16.69999129	2.100503211	0.125778701	0
WBGene00022730	ZK402.3	23.07679155	2.099742657	0.090989367	0
WBGene00020040	R12E2.15	10.52989042	2.099304983	0.199366271	0
WBGene00007381	C06C6.7	12.87065024	2.089428434	0.16234055	0
WBGene00018630	F49D11.6	9.024993635	2.088953826	0.231463191	0
WBGene00007306	C04G2.5	21.20190382	2.087678189	0.098466544	0
WBGene00009471	F36D3.5	13.67441992	2.080558908	0.152149701	0
WBGene00022632	ZC581.2	9.839269214	2.079891797	0.211386817	0
WBGene00004901	snf-2	15.31306431	2.072741757	0.135357739	0
WBGene00022654	ZK105.3	8.176033679	2.072347013	0.253466056	0
WBGene00011134	R08A2.3	16.33796746	2.069839628	0.126688931	0
WBGene00019997	R10F2.6	13.98167322	2.069689174	0.148028719	0
WBGene00044475	F56D6.13	16.95553658	2.0694379	0.122050865	0
WBGene00018870	F55C12.2	24.7011973	2.068089475	0.08372426	0
WBGene00019586	K09F6.3	8.491266191	2.067524498	0.243488362	0
WBGene00007767	C27C7.2	14.31254938	2.064925364	0.144273763	0
WBGene00011191	R10D12.10	9.972661508	2.055921876	0.206155786	0
WBGene00016389	C34B2.4	16.89724839	2.055486493	0.121646226	0
WBGene00018525	F47B3.1	20.32427972	2.05440013	0.101081079	0
WBGene00015944	C18A3.7	12.19871417	2.050486631	0.168090391	0
WBGene00012219	W03C9.1	16.8151687	2.049369591	0.121876243	0
WBGene00020766	T24C12.4	9.853667762	2.042979299	0.207331863	0
WBGene00007740	C26C6.6	10.08930441	2.041414549	0.202334518	0
WBGene00017384	F11G11.4	14.32295392	2.040781279	0.142483268	0
WBGene00014062	ZK673.6	11.79091324	2.039527872	0.172974547	0
WBGene00012925	wht-8	15.7387606	2.039426541	0.129579869	0
WBGene00002193	kin-5	12.97697589	2.038337334	0.15707337	0
WBGene00015689	C10G11.9	12.83329894	2.037532303	0.158769176	0
WBGene00006719	ubc-24	13.04917733	2.036151752	0.156036791	0
WBGene00018336	F42A9.7	21.23000292	2.034252753	0.095819711	0
WBGene00022777	ZK616.7	11.27257784	2.031158031	0.180185762	0

WBGene00020653	T21E3.2	11.42114555	2.024022082	0.177217082	0
WBGene00011039	R05H5.4	13.90839914	2.023747145	0.145505397	0
WBGene00001387	far-3	10.90831977	2.016687214	0.184876063	0
WBGene00004906	snf-7	21.69919606	2.014432376	0.092834424	0
WBGene00011322	T01C3.5	19.25676704	2.014300639	0.104602223	0
WBGene00005012	F26F4.2	14.12248228	2.011613229	0.142440485	0
WBGene00014168	ZK945.6	22.93203009	2.00893347	0.087603821	0
WBGene00012010	T25B9.4	13.78212254	2.001232506	0.145204957	0
WBGene00010072	F54F12.1	16.22011669	2.000821945	0.12335435	0
WBGene00010373	H08M01.1	17.18428684	1.996107514	0.11615888	0
WBGene00018526	F47B3.2	15.85198399	1.991164665	0.125609808	0
WBGene00022102	Y69F12A.1	8.921379403	1.991036825	0.223175894	0
WBGene00018605	F48E3.4	22.88131676	1.990357317	0.086986135	0
WBGene00003887	osm-7	10.09545099	1.990051193	0.197123556	0
WBGene00014007	ZK596.2	8.595254899	1.988350377	0.231331171	0
WBGene00016382	C33H5.16	14.94098206	1.985210456	0.132870145	0
WBGene00011491	T05F1.5	11.66671188	1.98481619	0.170126443	0
WBGene00009028	F21H7.2	22.2543172	1.981918463	0.089057707	0
WBGene00014182	ZK1025.2	14.44163432	1.974164049	0.13669949	0
WBGene00007512	C10C6.3	12.58550611	1.972814189	0.156752869	0
WBGene00016010	C23G10.1	8.763137058	1.971476844	0.224973869	0
WBGene00020074	R52.2	39.23163505	1.967828219	0.05015922	0
WBGene00017955	F31E8.5	9.973515573	1.963773803	0.196898856	0
WBGene00013304	Y57G11C.6	13.97936608	1.96167229	0.140326269	0
WBGene00016765	C49C8.1	11.16689924	1.960602491	0.175572686	0
WBGene00013586	Y80D3A.8	13.21533679	1.955670359	0.147984905	0
WBGene00016843	C50F7.3	9.489707087	1.945754282	0.205038392	0
WBGene00000734	col-161	11.3779762	1.945352557	0.17097527	0
WBGene00006661	C27F2.6	8.732905541	1.94389657	0.22259448	0
WBGene00000771	cpb-2	35.63500224	1.942115942	0.054500233	0
WBGene00012345	W08E3.4	12.80065199	1.941272705	0.151654205	0
WBGene00002227	klp-17	9.350473683	1.941045988	0.207587985	0
WBGene00009143	F26A3.5	12.02270592	1.940961317	0.161441304	0
WBGene00018980	F56F4.3	12.43804215	1.940155546	0.155985606	0
WBGene00007777	C27D8.1	15.82258901	1.939682961	0.12258948	0
WBGene00021579	clec-73	11.45649667	1.939234796	0.169269442	0
WBGene00017305	nspb-12	9.547526469	1.937654156	0.202948288	0
WBGene00007082	AH10.1	19.24732211	1.936622264	0.100617751	0
WBGene00010992	R03D7.8	16.73576743	1.928163753	0.11521215	0
WBGene00010265	F58G1.3	14.58653942	1.928047041	0.132179881	0
WBGene00012711	Y39E4A.1	13.11051064	1.926735983	0.14696117	0
WBGene00001725	grl-16	10.3520319	1.926235703	0.1860732	0
WBGene00015994	C18H7.4	36.76370013	1.925568812	0.052376905	0
WBGene00007699	C24H11.1	25.40659615	1.923190754	0.075696514	0
WBGene00000732	col-159	8.738501376	1.92298094	0.220058435	0
WBGene00019561	K09C6.7	13.0265786	1.916258822	0.14710377	0
WBGene00007307	C04G2.8	16.09854883	1.91235913	0.118790777	0
WBGene00020713	T23B3.5	11.63069984	1.910194858	0.16423731	0
WBGene00006039	ssp-10	11.17487405	1.90960389	0.170883706	0
WBGene00022700	ZK353.4	24.36944188	1.905264569	0.078182528	0
WBGene00013771	Y113G7C.1	10.45635969	1.904980716	0.182183931	0
WBGene00018548	clec-73	15.94104578	1.904572551	0.11947601	0
WBGene00016440	C35D10.2	9.129790148	1.903090283	0.208448415	0
WBGene00013978	ZK507.1	11.27904344	1.902328363	0.168660434	0
WBGene00007986	C36F7.5	14.75541566	1.900746072	0.128816844	0
WBGene00007081	AH6.3	14.58990186	1.900357667	0.130251573	0
WBGene00004909	snf-10	21.8155547	1.900196637	0.087102834	0

WBGene00015192	B0432.11	21.6179855	1.899493963	0.087866372	0
WBGene00020187	gsp-4	12.82998414	1.897912193	0.147927867	0
WBGene00015931	C17H12.5	9.894268966	1.893998915	0.191423835	0
WBGene00008141	C47E8.1	19.93037432	1.893964606	0.095029053	0
WBGene00015629	C09B9.4	24.49102756	1.892027702	0.077253913	0
WBGene00010563	K04G2.4	17.67399387	1.890054708	0.106939876	0
WBGene00013979	ZK507.3	16.42373277	1.889822103	0.11506654	0
WBGene00009457	F36A2.10	27.6917716	1.882274781	0.06797235	0
WBGene00012008	T25B9.2	10.28873656	1.877441908	0.182475457	0
WBGene00019810	R01H2.2	13.48851332	1.875216756	0.139023235	0
WBGene00004962	spe-8	11.94511576	1.874532204	0.15692876	0
WBGene00014238	ZK1225.4	12.06325283	1.870581416	0.155064429	0
WBGene00009250	F29D10.1	21.89108847	1.869858928	0.085416444	0
WBGene00015026	B0207.1	8.843269081	1.868838915	0.211328966	0
WBGene00009502	F37A8.2	11.48895457	1.868323733	0.162619124	0
WBGene00022753	ZK484.7	10.66115641	1.86517281	0.174950328	0
WBGene00022243	Y73B6BL.22	8.129341106	1.864812619	0.229392837	0
WBGene00003664	nhr-244	12.41099711	1.863987283	0.150188359	0
WBGene00013999	ZK550.5	11.41268414	1.859931838	0.162970587	0
WBGene00007977	gska-3	12.17554033	1.859830283	0.152751355	0
WBGene00008272	C53B4.2	8.130938573	1.859626956	0.228709999	0
WBGene00009344	F32H2.7	13.55282342	1.856871124	0.137009911	0
WBGene00010612	K07A1.5	14.94393616	1.853869065	0.124054937	0
WBGene00021006	dct-9	9.793730089	1.853649494	0.189268999	0
WBGene00012831	Y43F8C.9	8.417173276	1.842867115	0.21894133	0
WBGene00018120	F36H12.4	14.53953356	1.842658648	0.126734371	0
WBGene00016357	C33F10.11	19.28127627	1.842405049	0.095554102	0
WBGene00018119	F36H12.3	10.61724595	1.840793755	0.173377707	0
WBGene00009643	F42G4.6	13.66849657	1.840286153	0.134637057	0
WBGene00002208	kin-26	25.13080076	1.837490079	0.073117053	0
WBGene00007300	C04F12.6	12.59238986	1.837049792	0.145885715	0
WBGene00017902	F28E10.4	9.067279597	1.833969274	0.202262349	0
WBGene00006056	sss-1	11.36749191	1.832781971	0.16123011	0
WBGene00010728	K09G1.2	13.20930206	1.83182901	0.138677199	0
WBGene00014197	ZK1053.2	14.66063315	1.829823602	0.124812045	0
WBGene00016125	C26B2.2	27.49664379	1.82975359	0.066544616	0
WBGene00010692	K08H2.5	17.87180687	1.828111256	0.102290231	0
WBGene00007244	C01G12.3	11.67151885	1.828099095	0.156629066	0
WBGene00007060	wht-6	11.14687668	1.827988859	0.163991126	0
WBGene00016398	C34D4.2	15.34958787	1.82756352	0.119062709	0
WBGene00015230	tag-344	15.70892299	1.827311929	0.116323183	0
WBGene00018301	F41G3.5	29.31867907	1.823270584	0.062188019	0
WBGene00017910	F28H1.5	15.01834156	1.821068284	0.121256284	0
WBGene00013429	Y66D12A.3	9.647539104	1.820247034	0.188674751	0
WBGene00015937	C17H12.12	10.59285199	1.816340234	0.17146848	0
WBGene00013696	Y106G6A.4	10.71277127	1.81237427	0.169178845	0
WBGene00022705	ZK354.2	40.95571739	1.809491215	0.044181651	0
WBGene00019229	H23L24.2	9.562503061	1.805973381	0.188859901	0
WBGene00011114	R07E5.6	12.89544602	1.805271723	0.139992965	0
WBGene00007611	C15H7.4	12.16443354	1.80327519	0.148241608	0
WBGene00021858	Y54F10BM.3	22.97564685	1.803179276	0.078482198	0
WBGene00009390	F35C5.1	19.99283277	1.800675456	0.090066049	0
WBGene00008912	F17C8.7	20.7711243	1.800317015	0.086674028	0
WBGene00012819	Y43F8B.9	9.042007981	1.791408675	0.198120669	0
WBGene00013886	ZC412.5	14.15900817	1.791177522	0.126504449	0
WBGene00010574	K04H4.5	10.54229678	1.787096913	0.169516847	0
WBGene00018983	F56F4.7	21.91695373	1.786010051	0.081489886	0

WBGene00023424	C53D6.10	8.708597083	1.784473831	0.204909449	0
WBGene00013879	ZC376.8	11.26610849	1.781589274	0.15813706	0
WBGene00010460	clec-142	11.05834979	1.777291112	0.160719379	0
WBGene00014158	ZK938.1	27.69064838	1.775894809	0.064133378	0
WBGene00012642	Y39A1A.2	11.77221597	1.773915446	0.150686621	0
WBGene00007448	C08F8.6	11.2874314	1.773312371	0.157105041	0
WBGene00010682	K08F8.5	11.20464789	1.772476393	0.158191173	0
WBGene00016356	C33F10.8	24.52249556	1.770504528	0.072199199	0
WBGene00018926	F56A11.6	13.94719401	1.770463	0.126940444	0
WBGene00009708	F44G3.7	12.28718383	1.768540497	0.143933754	0
WBGene00013775	Y116A8A.4	8.869226611	1.768066861	0.199348482	0
WBGene00013303	Y57G11C.5	20.02633414	1.766810589	0.088224364	0
WBGene00015004	B0034.4	12.71620468	1.766442762	0.138912734	0
WBGene00009959	F53B6.4	11.58101715	1.764626121	0.152372292	0
WBGene00006394	taf-11.2	10.71945423	1.763987868	0.164559485	0
WBGene00016351	C33F10.1	22.43071991	1.762249193	0.078564094	0
WBGene00015345	C02F5.2	28.77510121	1.759954735	0.061162417	0
WBGene00003763	nlp-25	21.14917537	1.75833128	0.083139472	0
WBGene00014029	ZK637.12	12.17046037	1.755245236	0.144221762	0
WBGene00017112	E03H12.5	8.571096622	1.75401335	0.204642816	0
WBGene00020968	W03A5.4	12.47127428	1.753941496	0.140638515	0
WBGene00022634	ZC581.7	15.70934847	1.753730698	0.111636119	0
WBGene00017387	F11G11.9	8.953447373	1.748543992	0.195292821	0
WBGene00012636	Y38H8A.2	13.14277308	1.74626757	0.132869035	0
WBGene00009402	F35C11.3	10.29535761	1.745624177	0.169554497	0
WBGene00007457	C08F11.10	12.17089304	1.740011506	0.142964982	0
WBGene00009550	F38H4.6	21.65764993	1.739396636	0.080313268	0
WBGene00017550	F18A12.1	9.648965721	1.737100757	0.180029737	0
WBGene00021010	W03G1.2	14.36115755	1.734479789	0.120775765	0
WBGene00008063	C41G7.6	13.14694033	1.733359543	0.131845091	0
WBGene00011493	C33F10.12	12.00910777	1.731817734	0.144208693	0
WBGene00008559	F07H5.8	9.031961714	1.729884648	0.191529227	0
WBGene00011795	T16A9.5	12.58608418	1.729674314	0.137427518	0
WBGene00007563	C14A4.13	9.758330781	1.729581563	0.177241539	0
WBGene00002212	kin-31	14.78095101	1.728848467	0.11696463	0
WBGene00018122	F36H12.8	13.67818788	1.72781716	0.126319157	0
WBGene00019783	M70.1	23.88932725	1.726966633	0.0722903	0
WBGene00007649	C17G1.2	8.964925925	1.726350258	0.192567153	0
WBGene00016461	C35E7.9	15.52768735	1.722886116	0.110955745	0
WBGene00007209	BE10.1	16.07123448	1.722851881	0.107200967	0
WBGene00022090	Y69A2AR.19	9.569383388	1.722588344	0.18001038	0
WBGene00021787	Y51H7C.9	9.264471056	1.720569525	0.185716973	0
WBGene00011297	R102.10	13.70548005	1.720515422	0.125534853	0
WBGene00008652	F10D11.6	9.59487048	1.719865715	0.179248456	0
WBGene00015246	scl-23	8.676749812	1.718963685	0.198111473	0
WBGene00021113	gsp-3	23.44511075	1.71289613	0.073059844	0
WBGene00007239	C01G10.14	12.52072216	1.712809675	0.136797994	0
WBGene00020105	R148.7	16.16274443	1.712064021	0.105926566	0
WBGene00022888	rnh-1.1	10.57907777	1.710352654	0.161673134	0
WBGene00012784	Y43C5A.4	8.412117401	1.709287697	0.203193514	0
WBGene00013107	Y24D9B.1	22.12874742	1.705288705	0.077062143	0
WBGene00020659	T21G5.1	15.548112	1.704066684	0.109599589	0
WBGene00007335	C05C12.1	13.60942522	1.703830768	0.12519491	0
WBGene00019260	H34I24.1	26.24162169	1.701426648	0.064836947	0
WBGene00018000	F33D11.1	11.39129784	1.699596506	0.149201305	0
WBGene00011918	T22C1.8	10.30657643	1.697903957	0.16473986	0
WBGene00018359	F42G8.8	9.966886512	1.696021985	0.170165676	0

WBGene00016462	C35E7.10	9.044476237	1.692799092	0.187163861	0
WBGene00013121	spe-38	30.21300235	1.689517893	0.055920225	0
WBGene00020289	T06C10.3	10.43701744	1.687393971	0.161673963	0
WBGene00016605	C43E11.5	9.019686664	1.686482328	0.186977929	0
WBGene00019255	H32C10.1	9.956762657	1.683884914	0.16911972	0
WBGene00012401	Y6E2A.8	8.974926189	1.682672745	0.18748597	0
WBGene00012169	W01B6.2	29.12675853	1.679399941	0.057658319	0
WBGene00017026	ID/source/sourc	34.68539118	1.678982196	0.048406033	0
WBGene00009446	F35H8.1	12.57841412	1.678778488	0.133465036	0
WBGene00009548	F38H4.4	8.338470988	1.675635146	0.200952327	0
WBGene00021633	Y47G6A.3	18.49202609	1.674414573	0.090547924	0
WBGene00020990	W03D8.10	11.26837861	1.673841663	0.148543257	0
WBGene00012086	clec-118	8.392447064	1.670954604	0.199102192	0
WBGene00006409	tag-19	13.89276756	1.670744999	0.120260056	0
WBGene00016181	C28C12.11	11.374995	1.666611724	0.146515381	0
WBGene00022507	ZC21.8	10.64286994	1.666116912	0.15654771	0
WBGene00021632	Y47D9A.5	31.00906928	1.664841446	0.053688856	0
WBGene00021996	Y59E9AL.6	13.20726671	1.663009748	0.125916269	0
WBGene00045306	cyn-17	10.15712972	1.662620358	0.16368998	0
WBGene00018930	F56B3.6	10.12757579	1.660089932	0.163917799	0
WBGene00011617	T08G3.4	26.7653065	1.659625684	0.062006601	0
WBGene00044674	B0280.17	25.06361352	1.657986732	0.066151145	0
WBGene00019785	M70.3	11.63456478	1.657853235	0.14249379	0
WBGene00007159	B0379.7	19.57935317	1.656041589	0.084581016	0
WBGene00007269	C03C10.2	8.552575901	1.655970004	0.193622369	0
WBGene00007458	C08F11.11	10.19090736	1.655929577	0.162490887	0
WBGene00017386	nspd-5	19.99399073	1.65500205	0.082774973	0
WBGene00011669	T09F5.10	8.807418781	1.649761887	0.187315027	0
WBGene00012486	Y18D10A.21	14.87332251	1.647941825	0.1107985	0
WBGene00015024	B0205.10	10.14418219	1.646833584	0.162342666	0
WBGene00007337	C05C12.5	11.66751321	1.643100734	0.140826987	0
WBGene00007156	B0379.2	9.771618263	1.633557419	0.167173684	0
WBGene00010035	F54C8.1	9.303648733	1.633385451	0.175563964	0
WBGene00011120	R07E5.15	17.80081754	1.633200307	0.091748612	0
WBGene00011954	T23F11.2	20.42817658	1.629896363	0.079786679	0
WBGene00016288	C31H1.5	13.15046295	1.628959674	0.123870899	0
WBGene00019062	F58F12.2	8.298899363	1.628697684	0.196254661	0
	Y39B6B.N	11.48420789	1.628279274	0.141784204	0
WBGene00007987	C36H8.1	9.411113461	1.626514725	0.172829148	0
WBGene00009463	F36D1.4	9.454864517	1.626475968	0.172025307	0
WBGene00012357	W09D6.4	12.1962944	1.62606437	0.13332446	0
WBGene00015097	B0273.1	15.83107026	1.624329264	0.102603882	0
WBGene00000389	cdc-25.4	24.3330889	1.624125588	0.066745558	0
WBGene00013987	ZK512.8	13.80708656	1.622614326	0.117520399	0
WBGene00006050	ssq-1	13.41517809	1.622601099	0.120952632	0
WBGene00009705	F44F4.10	14.92741246	1.621875093	0.108650786	0
WBGene00020715	nspd-4	12.7221219	1.620232733	0.127355542	0
WBGene00019151	H04M03.1	8.333237402	1.615713263	0.193887824	0
WBGene00007778	C27D8.2	12.66153167	1.60832372	0.12702442	0
WBGene00007243	C01G12.13	12.24817364	1.605183688	0.131054942	0
WBGene00004084	pph-2	12.62430554	1.604190823	0.127071609	0
WBGene00009683	F44D12.6	10.57724068	1.603471336	0.151596374	0
WBGene00016058	nspd-3	10.22896833	1.600927256	0.156509161	0
WBGene00016040	C24A11.1	15.8079691	1.600887559	0.101270919	0
WBGene00020235	T05A12.4	10.29749913	1.598268099	0.155209345	0
WBGene00012191	W02A2.8	19.750271	1.597598209	0.080889939	0
WBGene00020986	W03D8.3	12.38361474	1.594641542	0.12877028	0

WBGene00019081	F59A3.8	17.63256583	1.594616265	0.090435861	0
WBGene00020218	T04G9.7	19.30651977	1.592995561	0.082510757	0
WBGene00010651	K08C9.2	13.37433811	1.592188494	0.119048022	0
WBGene00022849	ZK1127.2	9.822501413	1.589475043	0.161819783	0
WBGene00020914	sulp-6	8.109562407	1.586230001	0.19559995	0
WBGene00020985	W03D8.2	15.35905556	1.585567229	0.103233381	0
WBGene00007721	C25D7.12	15.13921633	1.583630285	0.104604509	0
WBGene00016445	C35D10.8	12.64043305	1.581289615	0.125097741	0
WBGene00022542	ZC190.8	12.83205386	1.580318515	0.123153981	0
WBGene00001538	gcy-12	12.41688891	1.57971658	0.127223219	0
WBGene00019431	K06A5.3	11.97043417	1.579045606	0.131912142	0
WBGene00044684	T08G11.2	9.08987275	1.577453027	0.173539616	0
WBGene00018125	rmd-4	13.96002299	1.575544053	0.112861136	0
WBGene00010466	K01D12.15	14.16946313	1.573717481	0.111064016	0
WBGene00000665	col-90	8.806330912	1.572539302	0.178569181	0
WBGene00012912	Y46G5A.22	11.35120929	1.572426427	0.138525014	0
WBGene00009470	F36D3.4	9.721357042	1.5689062	0.161387571	0
WBGene00010091	F55C5.1	10.99770666	1.567989009	0.14257418	0
WBGene00017279	F09C12.8	10.10965119	1.567883833	0.155087827	0
WBGene00010829	M02B1.4	13.1868348	1.561434065	0.118408556	0
WBGene00006057	sss-2	26.34169084	1.55953787	0.059204167	0
WBGene00011460	ttr-14	17.40937768	1.557422517	0.089458828	0
WBGene00017438	F13H8.5	10.36226206	1.557273496	0.150283161	0
WBGene00010378	H12D21.2	13.36903749	1.555801441	0.116373482	0
WBGene00009308	F32A11.3	14.06494632	1.552024086	0.110346961	0
WBGene00019642	K11C4.1	11.31403215	1.551643279	0.137143262	0
WBGene00008541	F07A5.2	13.32897573	1.551025909	0.116364974	0
WBGene00015627	C09B9.2	8.155092885	1.548616419	0.18989562	0
WBGene00017215	F07F6.1	8.34316657	1.546156161	0.185320064	0
WBGene00011365	T02C1.1	10.84228941	1.546051422	0.142594554	0
WBGene00007998	C38C6.5	10.28375502	1.54537717	0.150273627	0
WBGene00011951	T23F6.3	16.17440674	1.545248597	0.095536648	0
WBGene00014997	AC7.3	15.35988618	1.543357021	0.100479717	0
WBGene00015634	C09D4.3	12.25406002	1.542704387	0.125893327	0
WBGene00004969	spe-15	13.20692038	1.542560775	0.11679943	0
WBGene00007733	C25G4.6	10.15311383	1.540722157	0.151748733	0
WBGene00016612	C43G2.3	8.586352732	1.539662131	0.179315034	0
WBGene00004972	spe-26	9.817989126	1.539048974	0.156758065	0
WBGene00009458	F36A2.11	10.54408333	1.535665368	0.145642378	0
WBGene00008423	D2045.5	18.70783417	1.535115145	0.082057342	0
WBGene00015796	C15F1.5	18.7475421	1.535033135	0.081879167	0
WBGene00010719	K09E4.1	9.490857523	1.5301552	0.161224125	0
WBGene00015696	C10H11.7	21.5707287	1.527363716	0.070807238	0
WBGene00017737	F23C8.8	8.345748051	1.527236757	0.18299579	0
WBGene00012741	Y40H4A.2	8.376326803	1.525181194	0.182082341	0
WBGene00015051	B0218.7	9.400241731	1.524691022	0.162197001	0
WBGene00012360	tat-3	9.603879609	1.523747922	0.158659623	0
WBGene00009714	F44G4.5	10.32084163	1.521844401	0.147453517	0
WBGene00012637	C39H7.1	21.5590703	1.519638285	0.07048719	0
WBGene00012720	Y39E4B.11	21.1647622	1.518950069	0.071767878	0
WBGene00018360	F42G8.9	11.35383174	1.518284259	0.133724393	0
WBGene00043998	T21C12.4	12.77630662	1.516658335	0.118708668	0
WBGene00013190	Y54E2A.5	11.50749781	1.514805594	0.131636401	0
WBGene00013052	scrm-7	8.571194227	1.513847232	0.176620339	0
WBGene00008313	C54G4.3	9.567131268	1.510841551	0.157920019	0
WBGene00016413	C34F11.1	13.13602559	1.508948085	0.114870976	0
WBGene00007601	C15C6.2	19.71247391	1.507521244	0.076475497	0

WBGene00010712	K09B11.5	20.44524862	1.50639725	0.073679576	0
WBGene00002206	kin-24	15.3347269	1.502569149	0.097984735	0
WBGene00011968	T23G11.1	15.83927948	1.50238006	0.094851541	0
WBGene00007788	C28A5.1	8.859680652	1.501504208	0.169476109	0
WBGene00018087	F36D4.1	11.64611387	1.497873081	0.128615699	0
WBGene00009449	F35H8.4	9.946965804	1.497311084	0.150529429	0
WBGene00000681	col-107	8.473129954	1.496139993	0.176574654	0
WBGene00000601	col-12	10.01195664	1.492499221	0.149071683	0
WBGene00011133	R08A2.2	9.335958056	1.492298724	0.159844198	0
WBGene00009680	F44D12.3	11.55163775	1.487172755	0.128741291	0
WBGene00008651	F10D11.5	8.363812695	1.484987146	0.177549068	0
WBGene00045484	F34D10.9	10.64461669	1.483308152	0.139348198	0
WBGene00008259	C52E4.7	8.758726304	1.482430926	0.169251884	0
	yk116f3	10.49772805	1.481485546	0.141124398	0
WBGene00021381	Y37E11B.10	8.270316058	1.479256847	0.1788634	0
WBGene00022385	Y95B8A.4	11.34980487	1.478822149	0.13029494	0
WBGene00015970	C18E3.1	13.45001326	1.476121842	0.109748728	0
WBGene00010991	R03D7.5	14.88462336	1.475304834	0.099116034	0
WBGene00014225	ZK1098.9	10.15446994	1.474789009	0.14523545	0
WBGene00007715	C25D7.2	13.8187671	1.473620979	0.106639107	0
WBGene00013192	Y54E2A.7	12.02087709	1.472735559	0.122514817	0
WBGene00000704	col-130	8.777501232	1.472683113	0.167779311	0
WBGene00009685	F44D12.8	13.41880055	1.471938461	0.109692253	0
WBGene00018792	F54C1.8	8.953483968	1.46999175	0.164180977	0
WBGene00013847	ZC84.3	16.71505623	1.468757395	0.087870323	0
WBGene00013684	Y105E8A.27	27.48273201	1.465387004	0.053320281	0
WBGene00019410	K05F1.9	15.72512611	1.463634102	0.093076144	0
WBGene00021630	Y47D9A.3	8.473190536	1.462715126	0.172628612	0
WBGene00022761	ZK546.4	15.25370399	1.462134534	0.095854393	0
WBGene00012207	W02B12.12	9.050410435	1.461267498	0.161458699	0
WBGene00020905	T28H11.7	11.05027723	1.459602045	0.132087369	0
WBGene00022780	ZK622.1	16.85929619	1.456411837	0.086386277	0
WBGene00020353	T08B6.4	12.24867753	1.456201152	0.118886398	0
WBGene00008508	F01G10.5	17.15917743	1.455334492	0.084813768	0
WBGene00016153	C27A2.5	11.010025	1.449434964	0.131646837	0
WBGene00012171	W01B6.4	12.49652063	1.444638147	0.11560323	0
WBGene00020660	T21G5.2	10.29267516	1.444381113	0.140330972	0
WBGene00013700	Y106G6D.3	9.110965355	1.443131993	0.15839507	0
WBGene00009948	F52H3.6	25.46307396	1.440404877	0.056568381	0
WBGene00008074	C43F9.6	11.50469506	1.440343225	0.125196124	0
WBGene00012120	T28C6.5	21.34820587	1.439949274	0.067450599	0
WBGene00022708	ZK354.7	9.126505079	1.436141222	0.157359385	0
WBGene00017853	F27C1.3	11.77402313	1.426890287	0.121189696	0
WBGene00010547	K03H1.12	14.09728574	1.425532969	0.101121095	0
WBGene00011587	T07F10.1	10.27769317	1.420559224	0.138217711	0
WBGene00008383	D1081.5	8.830011785	1.412822069	0.160002286	0
WBGene00017114	E03H12.7	19.84084611	1.412677942	0.071200489	0
WBGene00008230	C50F4.2	24.0317666	1.412648078	0.058782532	0
WBGene00016742	C48B6.4	8.165272912	1.410774373	0.172777369	0
WBGene00011203	R10E4.7	16.13277073	1.410646954	0.087439844	0
WBGene00006038	ssp-11	16.50239697	1.410224477	0.085455736	0
WBGene00007190	rmd-3	9.753485696	1.410084933	0.14457241	0
WBGene00003837	oat-1	9.260553678	1.409295432	0.152182632	0
WBGene00012627	Y38H6C.15	19.6719293	1.40668681	0.071507313	0
WBGene00021386	Y37F4.5	18.41911734	1.405971694	0.076332197	0
WBGene00019024	F58A6.5	8.254897826	1.405232208	0.170230115	0
WBGene00007415	C07E3.4	17.63926536	1.400013926	0.079369174	0

WBGene00020940	W02D7.4	9.785385855	1.398277962	0.142894515	0
WBGene00016161	C27D6.3	10.76973274	1.397254779	0.129739039	0
WBGene00013290	Y57G11A.2	11.68674424	1.396000881	0.11945165	0
WBGene00013318	Y57G11C.23	10.63047538	1.395801097	0.131301851	0
WBGene00022778	ZK616.8	9.476703481	1.393709216	0.14706688	0
WBGene00008620	F09C6.10	17.22958793	1.389371782	0.080638712	0
WBGene00011262	R13H4.3	12.05262129	1.389027513	0.115246923	0
WBGene00016212	C29F5.3	8.138075604	1.384933029	0.170179425	0
WBGene00015630	C09B9.7	8.357659489	1.384638713	0.165673023	0
WBGene00020372	T09A12.1	16.61968301	1.383659454	0.083254263	0
WBGene00016541	C39H7.1	10.83117393	1.382486902	0.127639618	0
WBGene00018158	F37E3.3	10.16545098	1.382445249	0.135994483	0
WBGene00018497	F46F5.6	12.61108028	1.381467215	0.109543924	0
WBGene00013297	Y57G11B.5	9.413713454	1.380595286	0.146657883	0
WBGene00022834	ZK973.8	12.46524408	1.379434332	0.110662441	0
WBGene00014223	ZK1098.6	9.286147883	1.379182128	0.148520371	0
WBGene00022417	Y102A11A.7	9.2470301	1.378973018	0.149126044	0
WBGene00010155	F56F3.4	8.872495187	1.377324782	0.155235337	0
WBGene00016825	C50E10.1	13.48300876	1.375751158	0.102035917	0
WBGene00022162	Y71G12B.27	8.240057598	1.373140851	0.166642142	0
WBGene00021608	Y46H3D.1	8.449492918	1.371324529	0.162296666	0
WBGene00014127	ZK892.3	8.704159324	1.367749267	0.157137435	0
WBGene00013956	ZK265.3	20.03040054	1.364791882	0.068136025	0
WBGene00021650	Y47G6A.26	8.381401128	1.361967817	0.162498823	0
WBGene00010650	K08C9.1	19.7428883	1.359457104	0.068858066	0
WBGene00009079	F23B12.1	14.94928893	1.355570089	0.090677897	0
WBGene00007709	clec-87	9.380393814	1.352238689	0.144155855	0
WBGene00001424	fis-1	10.98954324	1.350465604	0.122886418	0
WBGene00010362	H04D03.1	11.87245188	1.349080695	0.113631178	0
WBGene00010869	M05B5.1	8.712264108	1.347597459	0.154678215	0
WBGene00021398	Y38C1AA.7	10.8076867	1.340156889	0.124000346	0
WBGene00021153	Y4C6A.3	9.172408017	1.339146315	0.145997247	0
WBGene00018081	F36A4.2	9.185835991	1.337555872	0.145610685	0
WBGene00021720	Y49F6B.8	17.29185468	1.335462563	0.07723073	0
WBGene00015467	C05D2.3	14.98345649	1.332670935	0.088942824	0
WBGene00009682	F44D12.5	8.35102983	1.332640487	0.159577982	0
WBGene00010241	F58D2.2	12.93668675	1.330809205	0.102870946	0
WBGene00022140	Y71G12A.4	9.200839784	1.328997247	0.144443038	0
WBGene00010779	K11H3.2	11.11809644	1.328866154	0.119522812	0
WBGene00015094	B0261.6	12.84198287	1.327477459	0.103370132	0
WBGene00007688	C18E9.8	12.338316	1.327332692	0.107578108	0
WBGene00007572	C14A6.8	9.509342212	1.320363661	0.138849106	0
WBGene00018083	F36A4.4	10.3802197	1.31889425	0.127058414	0
WBGene00006591	toh-1	11.90485854	1.318152376	0.110723901	0
WBGene00016512	C38C3.3	14.92182775	1.316332183	0.088215211	0
WBGene00017851	F27C1.1	8.756826613	1.311040394	0.149716382	0
WBGene00018199	F39E9.7	10.76703038	1.301312527	0.120860858	0
WBGene00019406	acdh-8	12.06211389	1.30113269	0.107869375	0
WBGene00022467	Y119C1B.1	11.78698405	1.300323377	0.110318583	0
WBGene00006624	try-6	10.43203367	1.298927957	0.124513398	0
WBGene00007631	wht-3	8.57971812	1.298798524	0.151380093	0
WBGene00021880	Y54G2A.15	23.72062222	1.295490085	0.054614507	0
WBGene00021786	Y51H7C.8	16.32620779	1.294926759	0.079315832	0
WBGene00021995	Y59E9AL.5	8.551895329	1.293040948	0.151199342	0
WBGene00019531	scrm-5	9.439045457	1.291208955	0.136794442	0
WBGene00001487	frk-1	8.523013857	1.2840656	0.150658631	0
WBGene00001245	elo-7	10.72259086	1.28385109	0.119733291	0

WBGene00012809	Y43F8A.2	18.55573721	1.282800896	0.069132306	0
WBGene00012859	Y45F3A.1	12.17140958	1.280549006	0.105209589	0
WBGene00021472	Y39G10AR.E	13.11594278	1.278426898	0.097471216	0
WBGene00011129	R07H5.9	9.071300662	1.275020074	0.140555376	0
WBGene00018163	F38A5.6	9.275240464	1.27133004	0.137067071	0
WBGene00007301	C04F12.7	11.74483576	1.268839342	0.108033809	0
WBGene00019912	R06B10.1	15.41733427	1.268037226	0.082247502	0
WBGene00016942	C55B7.3	10.69466615	1.265044489	0.118287422	0
WBGene00010452	K01A11.5	8.125469826	1.259736492	0.155035526	0
WBGene00016322	C32E8.4	13.14410292	1.259339946	0.095810262	0
WBGene00018098	F36F12.7	10.55360066	1.258732858	0.119270465	0
WBGene00007195	B0513.2	8.81283375	1.246943468	0.141491773	0
WBGene00003879	ora-1	14.24710302	1.245783682	0.087441193	0
WBGene00014169	ZK945.7	13.57787549	1.244471534	0.091654363	0
WBGene00010181	F57A8.6	9.609057143	1.24372937	0.129433029	0
WBGene00019773	M04G7.2	12.36828088	1.242176043	0.100432393	0
WBGene00004904	snf-5	11.02267083	1.242164277	0.112691769	0
WBGene00013175	Y53F4B.36	19.39475259	1.241585628	0.064016575	0
WBGene00009738	F45H7.6	9.629948316	1.240850858	0.128853325	0
WBGene00009043	F22B5.5	13.50874206	1.238803376	0.091703829	0
WBGene00019430	K06A5.2	10.49440104	1.238576585	0.118022608	0
WBGene00004955	spd-5	12.02582792	1.229749598	0.102259038	0
WBGene00013198	Y54E5A.2	8.958954015	1.22852469	0.137128139	0
WBGene00013474	Y69E1A.2	12.76292846	1.22815563	0.096228356	0
WBGene00018134	F37A4.4	25.55526572	1.227919945	0.048049586	0
WBGene00018500	F46F5.9	11.9810944	1.227245799	0.102431861	0
WBGene00011214	R10E9.2	13.0009089	1.226917689	0.094371686	0
WBGene00010623	K07A12.5	11.00657629	1.224996628	0.111296792	0
WBGene00017271	F08F8.6	9.890936768	1.224366992	0.123786758	0
WBGene00019184	H10E21.4	8.87301717	1.224133008	0.137961303	0
WBGene00015211	B0496.2	12.95696722	1.222638672	0.094361485	0
WBGene00007308	C04G2.9	14.96670461	1.22162003	0.081622512	0
WBGene00003663	nhr-245	12.42327308	1.219825065	0.098188703	0
WBGene00022228	Y73B6A.1	21.89389092	1.218695399	0.055663719	0
WBGene00007196	B0513.4	8.287385321	1.206257877	0.145553492	0
WBGene00021969	Y57G7A.6	8.681992402	1.201117383	0.138345823	0
WBGene00014241	ZK1251.3	9.627581306	1.199512104	0.12459122	0
WBGene00003469	msp-142	9.720930862	1.199302143	0.123373179	0
WBGene00019020	F57H12.5	10.27922932	1.197232992	0.116471085	0
WBGene00022760	ZK546.3	22.75786701	1.195567333	0.052534244	0
WBGene00014156	ZK930.6	9.967006208	1.192509759	0.119645733	0
WBGene00009321	F32B6.4	9.086218127	1.189164562	0.130875634	0
WBGene00018253	F40H6.1	12.93343859	1.188226452	0.091872432	0
WBGene00004078	pos-1	9.815286367	1.186858678	0.120919414	0
WBGene00001977	hmg-12	12.89816089	1.18464116	0.091845742	0
WBGene00005013	F43G6.6	11.2715079	1.183768823	0.105023111	0
WBGene00000707	col-133	13.9882413	1.182302473	0.084521167	0
WBGene00007559	C14A4.8	9.670751693	1.179662891	0.121982544	0
WBGene00009581	F40F9.3	9.477229897	1.176506734	0.12414036	0
WBGene00022650	ZK84.2	8.949046818	1.17241508	0.131010051	0
WBGene00004964	spe-10	18.818444	1.166260889	0.061974353	0
WBGene00016780	C49G7.1	8.56573322	1.165083833	0.136016825	0
WBGene00021360	Y37E11AL.4	19.65359813	1.163524998	0.059201628	0
WBGene00015386	C03B8.3	10.42733968	1.161518331	0.111391627	0
WBGene00009125	F25H5.2	9.973827123	1.160284618	0.116332939	0
WBGene00017175	F02C9.4	12.74748531	1.160281371	0.091020412	0
WBGene00003456	msp-63	9.545444057	1.159552457	0.121477058	0

WBGene00015384	C03B8.1	10.18096648	1.159512414	0.11389021	0
WBGene00022884	ZK1248.17	8.674414488	1.156074632	0.133274083	0
WBGene00007795	C28D4.8	8.312396164	1.151479214	0.138525546	0
WBGene00004174	pqn-95	8.498837857	1.149810838	0.135290361	0
WBGene00020293	T06D4.4	16.68753984	1.149138837	0.068862088	0
	Y67D8B.A	10.62458219	1.147510748	0.108005259	0
WBGene00007446	C08F8.4	10.29780045	1.146134764	0.111298988	0
WBGene00011537	T06E4.10	8.567431603	1.142294759	0.133329895	0
WBGene00013785	Y116A8C.4	11.26858652	1.132493926	0.100500087	0
WBGene00022288	Y75B7B.1	8.168270985	1.130497432	0.138401068	0
WBGene00022875	ZK1248.4	12.27017184	1.12635691	0.091796344	0
WBGene00016414	C34F11.2	8.858366355	1.116385934	0.126026164	0
WBGene00007080	sfxn-1.1	11.99785905	1.115299455	0.092958206	0
WBGene00012549	Y37D8A.8	8.339669818	1.109475496	0.133035902	0
WBGene00020380	T09B4.6	10.70147271	1.107558284	0.103495875	0
WBGene00014170	ZK945.8	11.35471994	1.107431344	0.097530485	0
WBGene00013723	Y106G6H.13	8.588577636	1.107103437	0.128904166	0
WBGene00019169	H06I04.5	10.27430753	1.099901606	0.1070536	0
WBGene00003526	nas-7	9.572498184	1.09922416	0.114831483	0
WBGene00015348	C02F5.5	11.68051008	1.098954655	0.094084475	0
WBGene00020414	T10E9.4	10.84473918	1.093744027	0.100854802	0
WBGene00022098	Y69A2AR.27	9.735498765	1.093350266	0.112305521	0
WBGene00003462	msp-74	12.17826546	1.09187223	0.08965745	0
WBGene00010082	F55A11.11	13.65262273	1.090148228	0.079848997	0
WBGene00012273	W05B2.7	10.05542762	1.086855336	0.108086436	0
WBGene00006052	ssq-3	8.447109358	1.079848252	0.127836424	0
WBGene00022110	Y71F9AL.4	8.858971374	1.076359312	0.121499355	0
WBGene00013449	Y67A6A.1	10.31167403	1.076159882	0.104363256	0
WBGene00011320	T01C3.3	10.04388172	1.07190472	0.106722157	0
WBGene00012905	Y46G5A.14	10.03093676	1.070043792	0.106674363	0
WBGene00012087	T27E7.1	11.2548478	1.0671025	0.094812699	0
	yk730h1	15.83296712	1.06308203	0.067143576	0
	Y39G10BM.E	8.403697942	1.062791807	0.126467159	0
	yk716c10	9.773760963	1.06183711	0.108641608	0
WBGene00009313	F32B4.2	8.961891469	1.054554566	0.117670982	0
WBGene00009501	F37A8.1	9.99744395	1.049164008	0.104943225	0
WBGene00015556	C06G3.12	8.750911769	1.045416145	0.119463682	0
WBGene00015572	C07G1.6	9.474395372	1.042646544	0.110048874	0
WBGene00001569	C36C9.1	8.122639453	1.037305354	0.127705453	0
WBGene00017392	F12A10.3	9.239378121	1.028986228	0.111369641	0
WBGene00010114	F55D12.6	8.171088577	1.024146457	0.125337823	0
WBGene00015820	C16A11.7	14.87690421	1.015267326	0.068244529	0
WBGene00007246	nspb-10	9.022959221	1.012183244	0.112178634	0
WBGene00021445	Y39A3CR.5	10.82926448	1.011575274	0.093411263	0
WBGene00016147	cyp-32A1	8.851781	1.006594798	0.11371664	0
WBGene00005444	srh-237	12.93497476	0.99730304	0.077101275	0
WBGene00021151	Y4C6A.1	13.75890574	0.994724329	0.072296762	0
WBGene00020463	T12E12.2	9.810743427	0.993266178	0.101242703	0
WBGene00009704	F44F4.9	11.03223817	0.992187502	0.089935287	0
WBGene00008677	F11A5.9	8.727677987	0.990706113	0.113513138	0
WBGene00006049	ssp-32	9.857861587	0.98974892	0.10040199	0
WBGene00003431	msp-33	8.277183182	0.981332507	0.118558752	0
WBGene00008487	F01D4.3	11.48739722	0.980866534	0.085386316	0
WBGene00011434	T04D3.5	18.51418421	0.976258066	0.052730277	0
WBGene00022833	ZK973.4	12.21039697	0.9759837	0.079930546	0
WBGene00020349	T08B2.11	10.88467298	0.968278309	0.08895796	0
WBGene00011554	T07A5.1	9.359359238	0.954985536	0.102035354	0

WBGene00010485	ant-1.3	8.215966408	0.94939237	0.115554558	0
WBGene00000248	ben-1	14.64823108	0.938965029	0.064100916	0
WBGene00003579	ndx-2	9.111796747	0.936404353	0.102768354	0
WBGene00001249	elt-1	15.24353636	0.934234098	0.061287229	0
WBGene00011088	R07B7.4	12.44050812	0.933637372	0.07504817	0
WBGene00013306	Y57G11C.8	10.11259485	0.931605721	0.092123311	0
WBGene00000727	col-154	8.348419567	0.931090357	0.111528937	0
WBGene00013701	Y106G6D.4	14.31188907	0.929027587	0.064912995	0
WBGene00004908	snf-9	15.38791278	0.927617932	0.060282245	0
WBGene00009398	nspb-11	13.56655466	0.91702481	0.067594524	0
WBGene00008662	F10G8.2	15.72990956	0.916628423	0.058272962	0
WBGene00017553	F18A12.4	11.83478988	0.898563326	0.075925583	0
WBGene00022628	ZC513.3	13.24014686	0.896153384	0.06768455	0
WBGene00003442	msp-49	9.444405029	0.893456023	0.094601621	0
WBGene00003438	msp-45	16.05696979	0.888614853	0.055341379	0
WBGene00021358	Y37E11AL.2	12.98020828	0.887421032	0.068367241	0
WBGene00000642	col-66	8.439533161	0.883216247	0.104652263	0
WBGene00013394	Y62H9A.6	10.80330351	0.878583483	0.081325447	0
WBGene00016807	C50D2.3	10.97583744	0.864132954	0.07873048	0
WBGene00019405	K05F1.1	11.37333415	0.853725913	0.07506382	0
WBGene00003424	msp-3	9.035307949	0.848627433	0.093923465	0
WBGene00010507	K02E2.6	9.35950822	0.847867197	0.090588862	0
WBGene00000878	cyn-2	10.88827784	0.846614707	0.077754694	0
WBGene00014246	ZK1307.3	13.15359994	0.831070853	0.063182008	0
WBGene00018127	F36H12.14	8.257722533	0.830993491	0.100632285	0
WBGene00017058	D2062.6	10.775963	0.825848733	0.076638045	0
WBGene00003443	msp-50	10.47679119	0.825712763	0.078813517	0
WBGene00003470	msp-152	9.132571881	0.81802586	0.089572343	0
WBGene00007461	C08F11.14	8.578967077	0.815033522	0.095003689	0
WBGene00015098	B0273.3	9.891382613	0.81137344	0.082028314	0
WBGene00003463	msp-76	10.48196319	0.81134467	0.077403885	0
WBGene00021273	Y23H5B.2	9.27192247	0.810748474	0.087441248	0
WBGene00003434	msp-38	8.563062308	0.808424763	0.094408371	0
WBGene00020356	T08B6.9	8.281053773	0.808107266	0.097585077	0
WBGene00020270	T05H4.7	9.255955407	0.788103857	0.085145598	0
WBGene00006051	ssq-2	13.36021458	0.787152491	0.058917653	0
WBGene00008891	clec-42	16.38566652	0.782307115	0.047743381	0
WBGene00007926	C34C12.7	9.753766901	0.77312541	0.07926429	0
WBGene00016707	C46E10.1	12.55254435	0.772389022	0.061532467	0
WBGene00013495	clec-236	8.340819224	0.769485074	0.092255335	0
WBGene00013524	Y73F8A.15	10.48683573	0.762732781	0.072732405	0
WBGene00015759	C14C6.5	8.372932764	0.752575682	0.089881969	0
WBGene00012660	Y39A1A.24	9.186175112	0.74755848	0.081378645	0
WBGene00003425	msp-56	12.12714997	0.742923952	0.061261216	0
WBGene00003464	msp-77	9.141467887	0.736831557	0.080603199	0
WBGene00015084	B0244.9	8.947491653	0.726690757	0.08121726	0
WBGene00003466	msp-81	13.08329927	0.680787432	0.052034844	0
WBGene00003429	msp-31	12.74209871	0.66142486	0.051908628	0
WBGene00003548	nas-30	16.18227719	0.66116883	0.040857589	0
WBGene00018470	F45E4.5	8.696724148	0.657546564	0.075608534	0
WBGene00001832	hcp-4	10.9628524	0.631692283	0.057621161	0
WBGene00003465	msp-78	10.49417562	0.626322863	0.059682903	0
WBGene00000865	cyb-1	8.718106862	0.609471389	0.069908685	0
WBGene00010010	F53H4.5	-15.10995194	-0.617356205	0.040857589	0
WBGene00015894	acdh-2	-15.41512724	-0.629824932	0.040857589	0
WBGene00010225	ttr-31	-11.05507914	-0.630881673	0.057067133	0
WBGene00006634	tsp-8	-12.76577654	-0.644973948	0.050523675	0

WBGene00020859	bath-25	-15.78979145	-0.645132808	0.040857589	0
WBGene00008577	F08G2.5	-11.93106845	-0.652350177	0.054676593	0
WBGene00002039	hum-6	-12.24590531	-0.681667715	0.055664951	0
WBGene00008532	F02E9.9	-16.1063486	-0.682259337	0.042359653	0
WBGene00002012	hsp-12.3	-14.69083524	-0.703592152	0.047893271	0
WBGene00015361	C02H6.1	-16.01072027	-0.719000934	0.04490747	0
WBGene00020590	T19H12.6	-17.63342296	-0.720459147	0.040857589	0
WBGene00003649	nhr-59	-11.98639484	-0.734852687	0.061307232	0
WBGene00004155	pqn-73	-13.63666483	-0.755169513	0.055377874	0
WBGene00020436	T12A2.1	-14.27791959	-0.782221992	0.054785432	0
WBGene00001160	efk-1	-11.87750553	-0.783943191	0.066002343	0
WBGene00015423	C04E6.7	-11.13159219	-0.798077142	0.071694788	0
WBGene00012252	W04E12.7	-11.26159188	-0.805011569	0.071482929	0
WBGene00012532	crp-1	-12.34795167	-0.814865025	0.065991919	0
WBGene00018282	F41D9.2	-11.1198026	-0.83228022	0.074846672	0
WBGene00009586	F40F9.10	-13.97618304	-0.83254667	0.059568959	0
WBGene00019944	R07G3.8	-13.00150404	-0.834154341	0.064158296	0
WBGene00009914	F49H6.13	-16.23976758	-0.838572732	0.051636991	0
WBGene00004249	pvf-1	-12.61930172	-0.840309934	0.066589258	0
WBGene00017243	F08C6.5	-19.01910187	-0.844252758	0.044389728	0
WBGene00000726	col-153	-11.79954496	-0.844342448	0.071557204	0
WBGene00018485	F46C8.3	-15.85875626	-0.846418769	0.05337233	0
WBGene00001045	dnj-27	-16.33482221	-0.849601196	0.052011659	0
WBGene00004207	ptb-1	-12.78517987	-0.870443384	0.068082217	0
WBGene00016996	D1005.2	-19.66466113	-0.891366502	0.045328343	0
WBGene00019683	K12H6.2	-13.10406082	-0.895890649	0.068367406	0
WBGene00010884	M7.8	-12.84623945	-0.902987725	0.070291989	0
WBGene00012866	Y45F10A.3	-11.4467229	-0.914595024	0.079900163	0
WBGene00008593	clec-227	-12.69266806	-0.928734006	0.073170905	0
	yk46h2	-19.21464695	-0.94204126	0.049027248	0
WBGene00011838	T19C4.1	-20.7688647	-0.943062605	0.045407518	0
WBGene00017666	F21E9.2	-14.47314876	-0.954838695	0.065973114	0
WBGene00015622	C09B8.3	-16.86243343	-0.980089518	0.058122662	0
WBGene00010211	F57G4.6	-20.12513238	-0.986838875	0.04903515	0
WBGene00004140	pqn-55	-12.50018909	-0.9924855	0.079397639	0
WBGene00008270	C53A5.13	-20.15169316	-1.012986452	0.050268057	0
WBGene00007170	B0393.5	-16.39215097	-1.024142182	0.062477596	0
WBGene00013643	Y105C5A.24	-14.06763664	-1.044773716	0.074267892	0
WBGene00000521	cky-1	-13.93902072	-1.081195591	0.077566108	0
WBGene00012255	lpr-6	-12.5963528	-1.081391067	0.085849538	0
WBGene00017426	F13C5.5	-12.77669209	-1.082326968	0.084711047	0
WBGene00011572	T07C12.8	-21.65911184	-1.082333377	0.049971272	0
WBGene00021526	Y41G9A.2	-17.49773024	-1.105699161	0.063191005	0
WBGene00022517	ZC123.1	-16.5324538	-1.11063105	0.067178839	0
WBGene00017998	F33D4.6	-12.04184169	-1.113352769	0.092457018	0
WBGene00009318	F32B4.8	-27.4036171	-1.119645723	0.040857589	0
WBGene00008392	D1086.5	-11.35876385	-1.156968205	0.101856876	0
WBGene00021121	W09G12.7	-14.92757329	-1.189878132	0.079710085	0
WBGene00014008	ZK596.3	-13.04729491	-1.218282717	0.093374353	0
	yk385d5	-13.59308447	-1.223072757	0.089977573	0
WBGene00007898	C33D9.3	-27.69659033	-1.240265889	0.044780454	0
WBGene00010033	F54B11.10	-13.49899022	-1.288671631	0.095464298	0
WBGene00000233	avr-15	-17.7944093	-1.300895781	0.073106994	0
WBGene00010209	fbxa-191	-15.39750107	-1.303482298	0.084655445	0
WBGene00015797	C15F1.8	-16.08537734	-1.316780222	0.081861942	0
WBGene00019550	K09C4.5	-27.81221408	-1.324633493	0.047627761	0
WBGene00018464	F45E1.3	-13.37048281	-1.351293998	0.10106546	0

WBGene00019285	cbn-1	-11.07539166	-1.387271525	0.125257108	0
WBGene00012917	Y46G5A.29	-26.02844241	-1.394612093	0.053580313	0
WBGene00015313	C01G8.6	-13.24668651	-1.401082292	0.10576851	0
WBGene00018039	F35D2.1	-15.48459246	-1.426433254	0.092119522	0
WBGene00011839	T19C4.5	-15.1728011	-1.448839484	0.095489256	0
WBGene00020281	T06A4.1	-15.07302373	-1.458879816	0.096787469	0
WBGene00003616	nhr-17	-20.10248617	-1.462762929	0.072765275	0
WBGene00017560	F18C5.5	-11.55071337	-1.498313862	0.129716132	0
WBGene00013348	fbxa-106	-14.59521023	-1.589869374	0.108930899	0
WBGene00016029	C24A1.2	-11.66447925	-1.663047804	0.142573686	0
WBGene00012324	rhy-1	-11.16586213	-1.792621349	0.160544822	0
WBGene00015014	bath-24	-11.61078956	-1.807574736	0.155680604	0
WBGene00007299	C04F12.5	-13.85826768	-1.808426432	0.130494408	0
WBGene00016136	C26B9.7	-11.38050627	-1.833111934	0.161074727	0
WBGene00019640	dos-2	-15.20722726	-1.833549249	0.120570911	0
WBGene00018591	F48B9.5	-34.88828074	-1.954740849	0.056028581	0
WBGene00016132	C26B9.2	-18.90319636	-2.0707579	0.109545384	0
WBGene00011023	R05A10.6	-12.186315	-2.072537628	0.170070906	0
WBGene00017219	F07F6.7	-11.66484258	-2.076905063	0.178048272	0
WBGene00021731	Y49G5A.1	-11.55326745	-2.092898227	0.181152062	0
WBGene00018668	bath-2	-15.48939066	-2.104875405	0.135891427	0
WBGene00011950	T23F6.2	-13.49099723	-2.118012597	0.156994517	0
WBGene00012085	T27D12.1	-20.71086142	-2.224815575	0.107422648	0
WBGene00009891	scl-10	-19.41511769	-2.343486756	0.120704226	0
WBGene00020232	T05A8.6	-35.82574496	-2.596123859	0.072465314	0
WBGene00018659	F52C6.2	-30.22625711	-3.011735309	0.099639704	0
WBGene00012564	fbxa-107	-31.12513625	-3.024883866	0.097184598	0
WBGene00018667	bath-7	-30.66486945	-3.042426336	0.099215369	0
WBGene00019141	bath-5	-12.66125031	-3.06012872	0.241692459	0
WBGene00018902	F55G1.6	-29.54762675	-3.43145287	0.116132944	0
WBGene00015843	C16C8.5	-13.92048662	-3.511738673	0.252271258	0
WBGene00010295	fbxa-193	-72.37942234	-3.562821304	0.04922423	0
WBGene00015842	C16C8.4	-24.49790433	-3.882522881	0.158483878	0
WBGene00004044	plk-3	-36.71416136	-6.742970439	0.183661295	0
