# Supplementary material for: Transcription factors CEP‐1/p53 and CEH‐23 collaborate with AAK‐2/AMPK to modulate longevity in Caenorhabditis elegans
Source: Aging Cell. 2017 May 30;16(4):814–24. doi: 10.1111/acel.12619 (PMC5506430; doi:10.1111/acel.12619)
Supplement: Supplementary file 8 — Table S5 Genes that are commonly regulated by CEH‐23, CEP‐1 and AAK‐2 (identified by overlap between gene lists in Table S3 and S4). [file ACEL-16-814-s008.pdf]

CEP- 1 and AAK-2 (identified by overlap between gene lists in Table S3 and S4)

Wormbase ID	Gene Name	Sequence Name	GO term	Enriched GOterm	Gene > Brief Description
WBGene00001538	gcy-12	F08B1.2	Kinase	Dual/tyrosine and Ser/Thr Kinase	gcy-12 encodes a membrane guanylyl cyclase; a gcy-12::GFP reporter is expressed in the PHA neurons as well as in a number of head sensory neurons including AFD, AWC, and ASE; when expressed in COS-M6 cells, GCY-12 exhibits temperature-dependent guanylyl cyclase activity.
WBGene00002212	kin-31	B0523.1	Kinase	Dual/tyrosine and Ser/Thr Kinase	kin-31 encodes a predicted tyrosine protein kinase expressed in the nucleus and cytoplasm of body-wall muscle cells; the expression of kin-31 was experimentally verified by RT-PCR.
WBGene00010991		R03D7.5	Kinase	Dual/tyrosine and Ser/Thr Kinase	
WBGene00012172		W01B6.5	Kinase	Dual/tyrosine and Ser/Thr Kinase	
WBGene00012207		W02B12.12	Kinase	Dual/tyrosine and Ser/Thr Kinase	
WBGene00017672		F21F3.2	Kinase	Dual/tyrosine and Ser/Thr Kinase	
WBGene00017737		F23C8.8	Kinase	Dual/tyrosine and Ser/Thr Kinase	
WBGene00022634		ZC581.7	Kinase	Dual/tyrosine and Ser/Thr Kinase	
WBGene00007977	gska-3	C36B1.10	Kinase	Dual/tyrosine and Ser/Thr Kinase	
WBGene00007448		C08F8.6	Kinase	Dual/tyrosine and Ser/Thr Kinase (TTBK)	
WBGene00007563		C14A4.13	Kinase	Dual/tyrosine and Ser/Thr Kinase (TTBK)	
WBGene00007777		C27D8.1	Kinase	Dual/tyrosine and Ser/Thr Kinase (TTBK)	
WBGene00009324		F32B6.10	Kinase	Dual/tyrosine and Ser/Thr Kinase (TTBK)	

WBGene00009402		F35C11.3	Kinase	Dual/tyrosine and Ser/Thr Kinase (TTBK)	
WBGene00012169		W01B6.2	Kinase	Dual/tyrosine and Ser/Thr Kinase (TTBK)	
WBGene00012637		Y38H8A.3	Kinase	Dual/tyrosine and Ser/Thr Kinase (TTBK)	
WBGene00014007		ZK596.2	Kinase	Dual/tyrosine and Ser/Thr Kinase (TTBK)	
WBGene00016541		C39H7.1	Kinase	Dual/tyrosine and Ser/Thr Kinase (TTBK)	
WBGene00016963		C56C10.6	Kinase	Dual/tyrosine and Ser/Thr Kinase (TTBK)	
WBGene00018178		F38E1.3	Kinase	Dual/tyrosine and Ser/Thr Kinase (TTBK)	
WBGene00018301		F41G3.5	Kinase	Dual/tyrosine and Ser/Thr Kinase (TTBK)	
WBGene00019642		K11C4.1	Kinase	Dual/tyrosine and Ser/Thr Kinase (TTBK)	
WBGene00021639		Y47G6A.13	Kinase	Dual/tyrosine and Ser/Thr Kinase (TTBK)	
WBGene00022707		ZK354.6	Kinase	Dual/tyrosine and Ser/Thr Kinase (TTBK)	
WBGene00015634		C09D4.3	Kinase	Dual/tyrosine and Ser/Thr Kinase (TTBK)	
WBGene00002193	kin-5	T13H10.1	Kinase	Dual/tyrosine and Ser/Thr Kinase(FER)	kin-5 encodes a predicted protein tyrosine kinase that is most closely related to the non-receptor tyrosine kinases Fes/Fps and Fer that contain an SH2 domain and a tyrosine kinase domain (OMIM:190030, murine Fes appears to be required for hemopoietic homeostasis); as loss of kin-5 activity via large-scale RNAi screens does not result in any obvious abnormalities, the precise role of kin-5 in C. elegans development and/or behavior is not yet known.

WBGene00002204	kin-21	W08D2.8	Kinase	Dual/tyrosine and Ser/Thr Kinase(FER)	
WBGene00004962	spe-8	F53G12.6	Kinase	Dual/tyrosine and Ser/Thr Kinase(FER)	spe-8 is predicted to encode a non-receptor tyrosine kinase with a SH2 domain; spe-8 is required for hermaphrodite spermatogenesis or sperm activation but not for male-derived sperm activation (sperm activation is the morphogenesis of a round non-motile spermatid to an amoeboid crawling sperm); however, mutant hermaphrodite spe-8 spermatids can transactivate and form functional sperm during mating with males; genetic and phenotypic evidence suggests that spe-8 acts in a common pathway with spe-12, spe-19, spe-27 and spe-29.
WBGene00009160		F26E4.5	Kinase	Dual/tyrosine and Ser/Thr Kinase(FER)	F26E4.5 encodes an SH2 domain-containing tyrosine kinase related to the vertebrate FER non-receptor protein tyrosine kinase; loss of F26E4.5 activity in an RNAi-hypersensitive strain results in axon guidance defects indicating that F26E4.5 likely plays a role in regulation of axon navigation.
WBGene00010712		K09B11.5	Kinase	Dual/tyrosine and Ser/Thr Kinase(FER)	
WBGene00012010		T25B9.4	Kinase	Dual/tyrosine and Ser/Thr Kinase(FER)	
WBGene00015994		C18H7.4	Kinase	Dual/tyrosine and Ser/Thr Kinase(FER)	
WBGene00016085		C25A8.5	Kinase	Dual/tyrosine and Ser/Thr Kinase(FER)	
WBGene00016416		C34F11.5	Kinase	Dual/tyrosine and Ser/Thr Kinase(FER)	
WBGene00016954		C55C3.4	Kinase	Dual/tyrosine and Ser/Thr Kinase(FER)	

WBGene00019081		F59A3.8	Kinase	Dual/tyrosine and Ser/Thr Kinase(FER)	
WBGene00022780		ZK622.1	Kinase	Dual/tyrosine and Ser/Thr Kinase(FER)	
WBGene00018999		F57B9.8	Kinase	Dual/tyrosine and Ser/Thr Kinase(FER)	
WBGene00016462		C35E7.10	Kinase	Dual/tyrosine and Ser/Thr Kinase(FER)	
WBGene00010366		H05L14.1	Kinase	non-receptor serine/threonine protein kinase	
WBGene00015629		C09B9.4	Kinase	non-receptor serine/threonine protein kinase	
WBGene00007269		C03C10.2	Kinase	non-receptor serine/threonine protein kinase (TTBK)	
WBGene00007335		C05C12.1	Kinase	non-receptor serine/threonine protein kinase (TTBK)	
WBGene00008423		D2045.5	Kinase	non-receptor serine/threonine protein kinase (TTBK)	
WBGene00011191		R10D12.10	Kinase	non-receptor serine/threonine protein kinase (TTBK)	
WBGene00013979		ZK507.3	Kinase	non-receptor serine/threonine protein kinase (TTBK)	
WBGene00017050		D2024.1	Kinase	non-receptor serine/threonine protein kinase (TTBK)	
WBGene00017802		F26A1.3	Kinase	non-receptor serine/threonine protein kinase (TTBK)	
WBGene00018004		F33D11.7	Kinase	non-receptor serine/threonine protein kinase (TTBK)	
WBGene00018122	ttbk-2	F36H12.8	Kinase	non-receptor serine/threonine protein kinase (TTBK)	

WBGene00019086		F59A6.4	Kinase	non-receptor serine/threonine protein kinase (TTBK)	
WBGene00020223		T05A7.6	Kinase	non-receptor serine/threonine protein kinase (TTBK)	
WBGene00020580		T19D12.5	Kinase	non-receptor serine/threonine protein kinase (TTBK)	
WBGene00022108		Y71F9AL.2	Kinase	non-receptor serine/threonine protein kinase (TTBK)	
WBGene00022229		Y73B6A.2	Kinase	non-receptor serine/threonine protein kinase (TTBK)	
WBGene00022632		ZC581.2	Kinase	non-receptor serine/threonine protein kinase (TTBK)	
WBGene00022705		ZK354.2	Kinase	non-receptor serine/threonine protein kinase (TTBK)	
WBGene00004901	snf-2	F55H12.1	transmitter symporter		
WBGene00004904	snf-5	Y46G5A.30	transmitter symporter		
WBGene00004906	snf-7	ZK1010.9	transmitter symporter		
WBGene00004908	snf-9	C49C3.1	transmitter symporter		
WBGene00004909	snf-10	Y32F6A.2	transmitter symporter		snf-10 encodes a member of the sodium:neurotransmitter symporter family.
WBGene00018980		F56F4.3	transmitter symporter		
WBGene00009129		F25H5.7	Phosphatases	Dual specific	
WBGene00009492		F36H1.3	Phosphatases	Dual specific	
WBGene00009548		F38H4.4	Phosphatases	Dual specific	
WBGene00010072		F54F12.1	Phosphatases	Dual specific	
WBGene00010634		K07F5.6	Phosphatases	Dual specific	
WBGene00010869		M05B5.1	Phosphatases	Dual specific	
WBGene00011918		T22C1.8	Phosphatases	Dual specific	
WBGene00012138		T28F4.3	Phosphatases	Dual specific	
WBGene00012679		Y39B6A.18	Phosphatases	Dual specific	
WBGene00012689		Y39B6A.30	Phosphatases	Dual specific	
WBGene00013586		Y80D3A.8	Phosphatases	Dual specific	
WBGene00015026		B0207.1	Phosphatases	Dual specific	
WBGene00015929		C17H12.3	Phosphatases	Dual specific	
WBGene00018526		F47B3.2	Phosphatases	Dual specific	
WBGene00019586		K09F6.3	Phosphatases	Dual specific	
WBGene00009401		F35C11.2	Phosphatases	Dual specific	
WBGene00013304		Y57G11C.6	Phosphatases	Dual specific	
WBGene00016053		C24D10.1	Phosphatases	Dual specific	
WBGene00015931		C17H12.5	Phosphatases	Dual specific	

WBGene00022090		Y69A2AR.19	Phosphatases	Dual specific	
WBGene00007610		C15H7.3	Phosphatases	Dual specific	
WBGene00010992		R03D7.8	Phosphatases	S/Thr specific/PPP1CA	
WBGene00012741		Y40H4A.2	Phosphatases	S/Thr specific/PPP1CA	
WBGene00016010		C23G10.1	Phosphatases	S/Thr specific/PPP1CA	
WBGene00021113	gsp-3	W09C3.6	Phosphatases	S/Thr specific/PPP1CA	
WBGene00008124		C47A4.3	Phosphatases	S/Thr specific/PPP1CB	
WBGene00016398		C34D4.2	Phosphatases	S/Thr specific/PPP1CB	
WBGene00020187	gsp-4	T03F1.5	Phosphatases	S/Thr specific/PPP1CB	
WBGene00009948		F52H3.6	Phosphatases	S/Thr specific/PPP1CC	
WBGene00010265		F58G1.3	Phosphatases	S/Thr specific/PPP1CC	
WBGene00011133		R08A2.2	Phosphatases	S/Thr specific/PPP1CC	
WBGene00014158		ZK938.1	Phosphatases	S/Thr specific/PPP1CC	
WBGene00000389	cdc-25.4	R05H5.2	Phosphatases	tyrosine specific	a homolog of dual specificity phosphatase Cdc25.
WBGene00016742		C48B6.4	Phosphatases	tyrosine specific	
WBGene00018347		F42C5.5	Phosphatases	tyrosine specific	
WBGene00021207		Y18H1A.1	Phosphatases	tyrosine specific	
WBGene00015084		B0244.9	Structural/MSP	B0244.9	
WBGene00007239		C01G10.14	Structural/MSP	C01G10.14	
WBGene00015696		C10H11.7	Structural/MSP	C10H11.7	
WBGene00007559		C14A4.8	Structural/MSP	C14A4.8	
WBGene00016461		C35E7.9	Structural/MSP	C35E7.9	
WBGene00007987		C36H8.1	Structural/MSP	C36H8.1	
WBGene00000696	col-122	T05A1.2	Structural/MSP	COLlagen	
WBGene00000707	col-133	F52B11.4	Structural/MSP	COLlagen	
WBGene00000710	col-137	Y51H4A.9	Structural/MSP	COLlagen	
WBGene00021006	dct-9	W03F11.3	Structural/MSP	DAF-16/FOXO Controlled, germline Tumor affecting	
WBGene00009031		F21H7.5	Structural/MSP	F21H7.5	
WBGene00009470		F36D3.4	Structural/MSP	F36D3.4	
WBGene00018119		F36H12.3	Structural/MSP	F36H12.3	
WBGene00018336		F42A9.7	Structural/MSP	F42A9.7	
WBGene00009682	msd-2	F44D12.5	Structural/MSP	F44D12.3; F44D12.5; F44D12.7; C35D10.11	
WBGene00009959		F53B6.4	Structural/MSP	F53B6.4	

WBGene00010091	ssp-35	F55C5.1	Structural/MSP	F55C5.1	
WBGene00010114		F55D12.6	Structural/MSP	F55D12.6	
WBGene00010254		F58E6.5	Structural/MSP	F58E6.5	
WBGene00019410		K05F1.9	Structural/MSP	K05F1.9	
WBGene00019431		K06A5.3	Structural/MSP	K06A5.3	
WBGene00003424	msp-3	F26G1.7	Structural/MSP	Major Sperm Protein	msp-3 encodes a protein that belongs to a family of proteins called the Major Sperm Proteins (MSPs) that is conserved in nematodes; msp-3 is similar in nucleotide sequence to the msp genes from the nematode Onchocerca volvulus; the msp gene family consists of closely related, small, basic proteins that make up 15% of sperm protein; this multigene family consists of over fifty genes, including many pseudogenes; MSPs are involved in both extracellular signaling and cytoskeletal functions during reproduction-MSP antagonizes Eph/ephrin signaling, in part, by binding VAB-1 Eph receptor tyrosine kinase on oocytes and sheath cells to promote oocyte maturation and MAPK activation; MSP proteins assemble into fibrous networks that drive movement of the C. elegans sperm; msp genes are expressed only in late primary spermatocytes.
WBGene00003431	msp-33	R05F9.8	Structural/MSP	Major Sperm Protein	msp-33 encodes a protein that belongs to a family of proteins called the Major Sperm Proteins (MSPs) that is conserved in nematodes; msp-33 has a C. briggsae homolog as predicted by the Wobble Aware Bulk Aligner (WABA); the MSP family consists of closely related, small, basic proteins that make up 15% of sperm protein; this multigene family consists of over fifty genes, including many pseudogenes; MSPs are involved in both extracellular signaling and cytoskeletal functions during reproduction-MSP antagonizes Eph/ephrin signaling, in part, by binding VAB-1 Eph receptor tyrosine kinase on oocytes and sheath cells to promote oocyte maturation and MAPK activation; MSPs assemble into fibrous networks that drive movement of the C. elegans sperm; msp genes are expressed only in late primary spermatocytes.
WBGene00003434	msp-38	K08F4.8	Structural/MSP	Major Sperm Protein	msp-38 encodes a member of the major sperm protein family; msp-38 has a C. briggsae homolog as predicted by the Wobble Bulk Aware Bulk Aligner (WABA).

WBGene00003465	msp-78	T13F2.11	Structural/MSP	Major Sperm Protein	msp-78 encodes a protein that belongs to a family of proteins called the Major Sperm Proteins (MSPs) that is conserved in nematodes; this family consists of closely related, small, basic proteins that make up 15% of sperm protein; this multigene family consists of over fifty genes, including many pseudogenes; MSPs are involved in both extracellular signaling and cytoskeletal functions during reproduction-MSP antagonizes Eph/ephrin signaling, in part, by binding VAB-1 Eph receptor tyrosine kinase on oocytes and sheath cells to promote oocyte maturation and MAPK activation; MSPs assemble into fibrous networks that drive movement of the C. elegans sperm; msp genes are expressed only in late primary spermatocytes.
WBGene00022875		ZK1248.4	Structural/MSP	Major Sperm Protein	
WBGene00011134	ssp-33	R08A2.3	Structural/MSP	R08A2.3	
WBGene00006039	ssp-10	K07F5.9	Structural/MSP	Sperm-Specific family, class P	
WBGene00006047	ssp-19	C55C2.2	Structural/MSP	Sperm-Specific family, class P	ssp-19 encodes a member of the C. elegans sperm-specific protein (ssp) family; loss of ssp-19 function via RNAi has been reported to result in a moderate increase of fat content; in situ hybridization experiments detect ssp-19 mRNA in the spermatheca; the SSP-19 crystal structure has been determined and reveals monomer-monomer interactions between SSP-19 molecules to be strikingly different from equivalent interactions between MSP (major sperm protein) monomers.
WBGene00006048	ssp-31	ZK1225.6	Structural/MSP	Sperm-Specific family, class P	
WBGene00006049	ssp-32	F32B6.7	Structural/MSP	Sperm-Specific family, class P	
WBGene00013858	ssp-34	ZC168.6	Structural/MSP	Sperm-specific protein ZC168.6	
WBGene00009550		F38H4.6	Structural/MSP	Uncharacterized protein R05D3.5; Uncharacterized protein ZC262.1	

WBGene00014246		ZK1307.3	Structural/MSP	Uncharacterized protein ZK1307.3	
WBGene00013290		Y57G11A.2	Structural/MSP	Y57G11A.2	
WBGene00022884		ZK1248.17	Structural/MSP	ZK1248.17	
WBGene00022760		ZK546.3	Structural/MSP	ZK546.3	
WBGene00003425	msp-10	K07F5.2	Structural/MSP		msp-10 encodes a protein that belongs to a family of proteins called the Major Sperm Proteins (MSPs) that is conserved in nematodes; this family consists of closely related, small, basic proteins that make up 15% of sperm protein; this multigene family consists of over fifty genes, including many pseudogenes; MSPs are involved in both extracellular signaling and cytoskeletal functions during reproduction-MSP antagonizes Eph/ephrin signaling, in part, by binding VAB-1 Eph receptor tyrosine kinase on oocytes and sheath cells to promote oocyte maturation and MAPK activation; MSPs assemble into fibrous networks that drive movement of the C. elegans sperm; msp genes are expressed only in late primary spermatocytes.
WBGene00003429	msp-31	R05F9.13	Structural/MSP		msp-31 encodes a member of the major sperm protein family; msp-31 has a C. briggsae homolog as predicted by the Wobble Aware Bulk Aligner (WABA); expression is enriched in sperm.
WBGene00003438	msp-45	F58A6.8	Structural/MSP		msp-45 encodes a member of the major sperm protein family.

WBGene00003442	msp-49	C34F11.6	Structural/MSP		msp-49 encodes a protein that belongs to a family of proteins called the Major Sperm Proteins (MSPs) that is conserved in nematodes; this family consists of closely related, small, basic proteins that make up 15% of sperm protein; this multigene family consists of over fifty genes, including many pseudogenes; MSPs are involved in both extracellular signaling and cytoskeletal functions during reproduction-MSP antagonizes Eph/ephrin signaling, in part, by binding VAB-1 Eph receptor tyrosine kinase on oocytes and sheath cells to promote oocyte maturation and MAPK activation; MSPs assemble into fibrous networks that drive movement of the C. elegans sperm; msp genes are expressed only in late primary spermatocytes.
WBGene00003443	msp-50	C34F11.4	Structural/MSP		msp-50 encodes a protein that belongs to a family of proteins called the Major Sperm Proteins (MSPs) that is conserved in nematodes; this family consists of closely related, small, basic proteins that make up 15% of sperm protein; this multigene family consists of over fifty genes, including many pseudogenes; MSPs are involved in both extracellular signaling and cytoskeletal functions during reproduction-MSP antagonizes Eph/ephrin signaling, in part, by binding VAB-1 Eph receptor tyrosine kinase on oocytes and sheath cells to promote oocyte maturation and MAPK activation; MSPs assemble into fibrous networks that drive movement of the C. elegans sperm; msp genes are expressed only in late primary spermatocytes.

WBGene00003456	msp-63	K05F1.7	Structural/MSP		msp-63 encodes a protein that belongs to a family of proteins called the Major Sperm Proteins (MSPs) that is conserved in nematodes; this family consists of closely related, small, basic proteins that make up 15% of sperm protein; this multigene family consists of over fifty genes, including many pseudogenes; MSPs are involved in both extracellular signaling and cytoskeletal functions during reproduction-MSP antagonizes Eph/ephrin signaling, in part, by binding VAB-1 Eph receptor tyrosine kinase on oocytes and sheath cells to promote oocyte maturation and MAPK activation; MSPs assemble into fibrous networks that drive movement of the C. elegans sperm; msp genes are expressed only in late primary spermatocytes.
WBGene00003462	msp-74	F09C12.7	Structural/MSP		msp-74 encodes a protein that belongs to a family of proteins called the Major Sperm Proteins (MSPs) that is conserved in nematodes; this family consists of closely related, small, basic proteins that make up 15% of sperm protein; this multigene family consists of over fifty genes, including many pseudogenes; MSPs are involved in both extracellular signaling and cytoskeletal functions during reproduction-MSP antagonizes Eph/ephrin signaling, in part, by binding VAB-1 Eph receptor tyrosine kinase on oocytes and sheath cells to promote oocyte maturation and MAPK activation; MSPs assemble into fibrous networks that drive movement of the C. elegans sperm; msp genes are expressed only in late primary spermatocytes.

WBGene00003463	msp-76	ZK1251.6	Structural/MSP		msp-76 encodes a protein that belongs to a family of proteins called the Major Sperm Proteins (MSPs) that is conserved in nematodes; this family consists of closely related, small, basic proteins that make up 15% of sperm protein; this multigene family consists of over fifty genes, including many pseudogenes; MSPs are involved in both extracellular signaling and cytoskeletal functions during reproduction-MSP antagonizes Eph/ephrin signaling, in part, by binding VAB-1 Eph receptor tyrosine kinase on oocytes and sheath cells to promote oocyte maturation and MAPK activation; MSPs assemble into fibrous networks that drive movement of the C. elegans sperm; msp genes are expressed only in late primary spermatocytes.
WBGene00003464	msp-77	F32B6.6	Structural/MSP		msp-77 encodes a protein that belongs to a family of proteins called the Major Sperm Proteins (MSPs) that is conserved in nematodes; this family consists of closely related, small, basic proteins that make up 15% of sperm protein; this multigene family consists of over fifty genes, including many pseudogenes; MSPs are involved in both extracellular signaling and cytoskeletal functions during reproduction-MSP antagonizes Eph/ephrin signaling, in part, by binding VAB-1 Eph receptor tyrosine kinase on oocytes and sheath cells to promote oocyte maturation and MAPK activation; MSPs assemble into fibrous networks that drive movement of the C. elegans sperm; msp genes are expressed only in late primary spermatocytes.
WBGene00003466	msp-79	T13F2.10	Structural/MSP		msp-79 encodes a member of the major sperm protein family.

WBGene00003469	msp-142	K05F1.2	Structural/MSP		msp-142 encodes a protein that belongs to a family of proteins called the Major Sperm Proteins (MSPs) that is conserved in nematodes; this family consists of closely related, small, basic proteins that make up 15% of sperm protein; this multigene family consists of over fifty genes, including many pseudogenes; MSPs are involved in both extracellular signaling and cytoskeletal functions during reproduction-MSP antagonizes Eph/ephrin signaling, in part, by binding VAB-1 Eph receptor tyrosine kinase on oocytes and sheath cells to promote oocyte maturation and MAPK activation; MSPs assemble into fibrous networks that drive movement of the C. elegans sperm; msp genes are expressed only in late primary spermatocytes.
WBGene00003470	msp-152	ZK546.6	Structural/MSP		msp-152 encodes a protein that belongs to a family of proteins called the Major Sperm Proteins (MSPs) that is conserved in nematodes; this family consists of closely related, small, basic proteins that make up 15% of sperm protein; this multigene family consists of over fifty genes, including many pseudogenes; MSPs are involved in both extracellular signaling and cytoskeletal functions during reproduction-MSP antagonizes Eph/ephrin signaling, in part, by binding VAB-1 Eph receptor tyrosine kinase on oocytes and sheath cells to promote oocyte maturation and MAPK activation; MSPs assemble into fibrous networks that drive movement of the C. elegans sperm; msp genes are expressed only in late primary spermatocytes.
WBGene00000878	cyn-2	ZK520.5			cyn-2 is a predicted member of the cytosolic Cyclosporin A-binding cyclophilin family that is functional when expressed in E. coli.

WBGene00001424	fis-1	F41G3.4			fis-1 encodes a protein similar to the yeast and human Fis1 proteins involved in mitochondrial fission; fis-1 does not seem to be required for mitochondrial fission in C. elegans, but it is possible that it may function redundantly along with fis-2 and other genes; fis-1 expression is enriched in the germline.
WBGene00002227	klp-17	W02B12.7			klp-17 encodes a C-terminal kinesin motor protein orthologous to Drosophila NCD and Saccharomyces cerevisiae KAR3; by homology, KLP-17 is predicted to function as a minus-end directed motor; loss of klp-17 activity via RNAi results in embryonic lethality generally at the one- or two-cell stage with disorganized mitotic spindles and polyploid nuclei, suggesting that KLP-17 plays a role in chromosome segregation and germline development; in situ hybridization studies reveal that klp-17 mRNA is localized specifically to cell nuclei during early development, from the one-cell stage of embryogenesis until early larval stages; a klp-17::gfp transgene did not yield detectable GFP expression, but did result in a small percentage of morphologically abnormal males and intersexual animals that grew slowly and died upon reaching maturity, consistent with a role for klp-17 in chromosome dynamics.
WBGene00004964	spe-10	AC3.10			spe-10 encodes a DHHC-CRD family zinc-finger transmembrane protein with homologs in other eukaryotes and is predicted to be a palmitoyl transferase; spe-10 is required during spermatogenesis and thus for fertility; specifically, spe-10 is required for the proper biogenesis and functioning of specialized Golgi-derived fibrous body-membranous organelle (FB-MO) complexes during spermatid formation; spe-10 localizes to the FB-MOs of spermatids.

WBGene00004972	spe-26	R10H10.2			spe-26 encodes a Kelch motif-containing protein similar to the Drosophila proteins kelch and diablo and the Limulus (horseshoe crab) actin-bundling protein scruin; SPE-26 activity is required for several processes including embryogenesis, spermatogenesis, thermotolerance, and regulation of lifespan; SPE-26 mRNA is detected in spermatogonial cells and spermatocytes, but not in spermatids.
WBGene00005012		F26F4.2			F26F4.2 encodes a novel protein with high similarity to C. elegans F37A8.1 and C. briggsae CBG18186.
WBGene00006050	ssq-1	K07F5.11			
WBGene00006051	ssq-2	T28H11.5			
WBGene00006052	ssq-3	ZC477.1			
WBGene00006056	sss-1	F32B6.5			
WBGene00006057	sss-2	F47B8.11			
WBGene00006624	try-6	F48A9.3			
WBGene00007060	wht-6	T26A5.1			
WBGene00007080	sfxn-1.1	AH6.2			
WBGene00007081		AH6.3			
WBGene00007082	acs-10	AH10.1			
WBGene00007159		B0379.7			
WBGene00007190	rmd-3	B0491.3			
WBGene00007230		C01G10.1			
WBGene00007301		C04F12.7			
WBGene00007306		C04G2.5			
WBGene00007307		C04G2.8			
WBGene00007308		C04G2.9			
WBGene00007337		C05C12.5			

WBGene00007446	mboa-4	C08F8.4			mboa-4 encodes a putative transmembrane O-acyl transferase (MBOAT) orthologous to human MBOAT1 (OMIM:611732), and paralogous to C54G7.2 and ZK550.1; MBOA-4 has no obvious function in mass RNAi assays, and mboa-4(RNAi) animals incorporate exogenous arachidonic acid into phosphatidylcholine (PC), phosphatidylserine (PS) and phosphatidylethanolamine (PE) normally.
WBGene00007457		C08F11.10			
WBGene00007572		C14A6.8			
WBGene00007597		C15A11.2			
WBGene00007601		C15C6.2			
WBGene00007631	wht-3	C16C10.12			wht-3 encodes an ATP-binding cassette (ABC) transporter; wht-3 activity is required for efficient RNA interference (RNAi) of a germline-expressed target, pop-1, as well as for normal pharyngeal and body morphology.
WBGene00007733		C25G4.6			
WBGene00007778		C27D8.2			
WBGene00007795		C28D4.8			
WBGene00007898		C33D9.3			
WBGene00007986		C36F7.5			
WBGene00007998		C38C6.5			
WBGene00008001		C38C10.3			
WBGene00008074	nkb-2	C43F9.6			
WBGene00008141		C47E8.1			
WBGene00008272		C53B4.2			
WBGene00008312		C54G4.2			
WBGene00008313		C54G4.3			
WBGene00008383		D1081.5			
WBGene00008541		F07A5.2			
WBGene00008590		F08H9.2			
WBGene00008651		F10D11.5			
WBGene00008660	clec-153	F10F2.8			
WBGene00008912		F17C8.7			
WBGene00008950	wht-5	F19B6.4			
WBGene00009028		F21H7.2			
WBGene00009043		F22B5.5			
WBGene00009075		F23B2.7			
WBGene00009143		F26A3.5			
WBGene00009149		F26D2.10			
WBGene00009185		F27C8.5			

WBGene00009308		F32A11.3			
WBGene00009313		F32B4.2			
WBGene00009321		F32B6.4			
WBGene00009344		F32H2.7			
WBGene00009446		F35H8.1			
WBGene00009449		F35H8.4			
WBGene00009457		F36A2.10			
WBGene00009458		F36A2.11			
WBGene00009463		F36D1.4			
WBGene00009471		F36D3.5			
WBGene00009501		F37A8.1			
WBGene00009549		F38H4.5			
WBGene00009685		F44D12.8			
WBGene00009708		F44G3.7			
WBGene00009714		F44G4.5			
WBGene00010014		F54B3.2			
WBGene00010035		F54C8.1			
WBGene00010082		F55A11.11			F55A11.11 encodes a member of the histidine phosphatase superfamily orthologous to Bombyx mori ecdysteroid phosphate phosphatase (EPP), human STS1 (OMIM:609201) and human UBASH3A (OMIM:605736); F55A11.11 lacks the consensus sequence for phosphatase activity, and thus is predicted to be catalytically inactive; F55A11.11 is also paralogous to C. elegans C52E4.7, F09C12.8, F53B6.7, and T07F12.1; loss of F55A11.11 activity via large-scale RNAi results in no obvious defects.
WBGene00010155		F56F3.4			
WBGene00010181		F57A8.6			
WBGene00010241		F58D2.2			
WBGene00010373		H08M01.1			
WBGene00010466		K01D12.7			
WBGene00010486		K01H12.4			
WBGene00010510	ent-3	K02E11.1			
WBGene00010563		K04G2.4			
WBGene00010574		K04H4.5			
WBGene00010612		K07A1.5			
WBGene00010650		K08C9.1			
WBGene00010651		K08C9.2			
WBGene00010679		K08F4.5			
WBGene00010682		K08F8.5			
WBGene00010719		K09E4.1			
WBGene00010728		K09G1.2			
WBGene00010829		M02B1.4			
WBGene00010906		M88.3			
WBGene00011120		R07E5.15			

WBGene00011203		R10E4.7			
WBGene00011214		R10E9.2			
WBGene00011297		R102.10			
WBGene00011322	irld-14	T01C3.5			
WBGene00011460	ttr-14	T05A10.3			
WBGene00011491		T05F1.5			
WBGene00011669		T09F5.10			
WBGene00011790		T15H9.5			
WBGene00011795		T16A9.5			
WBGene00011910		T22B3.2			alg-3 encodes an Argonaute (AGO) protein that is a member of the AGO clade of Argonaute small RNA-binding proteins; together with its paralog, alg-4, alg-3 is required for normal brood sizes at elevated temperatures, specifically for the process of spermiogenesis in which spermatids are converted to mobile ameboid sperm; alg-3/4 is also required for accumulation of male germline-specific 26G-RNAs, suggesting that alg-3/4 mediate thermotolerant fertility via a 26G-RNA-mediated pathway; a GFP::ALG-3 rescuing fusion protein is expressed in germ cells during spermatogenesis and localizes to the cytoplasm and to P granules.
WBGene00011954		T23F11.2			
WBGene00011968		T23G11.1			
WBGene00012087		T27E7.1			
WBGene00012102		T27F6.1			
WBGene00012120		T28C6.5			
WBGene00012171		W01B6.4			
WBGene00012191		W02A2.8			
WBGene00012357		W09D6.4			
WBGene00012486		Y18D10A.21			
WBGene00012547		Y37D8A.5			
WBGene00012627		Y38H6C.15			
WBGene00012636		Y38H8A.2			
WBGene00012711		Y39E4A.1			
WBGene00012720		Y39E4B.11			
WBGene00012784		Y43C5A.4			
WBGene00012809		Y43F8A.2			
WBGene00012827		Y43F8C.5			
WBGene00012912		Y46G5A.22			
WBGene00012925	wht-8	Y47D3A.11			
WBGene00013052	scrm-7	Y50E8A.9			scrm-7 encodes a putative phospholipid scramblase homologous to human PLSCR1-5, and paralogous to other C. elegans SCRMs (e.g., SCRM-1); scrm-7(RNAi) animals have no obvious phenotype.

WBGene00013053		Y50E8A.10			
WBGene00013085	mpz-6	Y51B9A.3			
WBGene00013175		Y53F4B.36			
WBGene00013190		Y54E2A.5			
WBGene00013303		Y57G11C.5			
WBGene00013429		Y66D12A.3			
WBGene00013437		Y66D12A.11			
WBGene00013449		Y67A6A.1			
WBGene00013474		Y69E1A.2			
WBGene00013524		Y73F8A.15			
WBGene00013526		Y73F8A.20			
WBGene00013684		Y105E8A.27			
WBGene00013696		Y106G6A.4			
WBGene00013700		Y106G6D.3			
WBGene00013712	dlc-6	Y106G6G.3			dlc-6 encodes a putative dynein light chain 1; DLC-6 is orthologous to human DYNLL1 (OMIM:601562) and DYNLL2 (OMIM:608942), but much more divergent from them than its paralog DLC-1; DLC-6's other paralogs are DLC-2/-5; DLC-6 has no obvious function in mass RNAi assays.
WBGene00013723		Y106G6H.13			
WBGene00013785	nep-23	Y116A8C.4			Y116A8C.4 encodes a neprilysin; neprilysins are thermolysin-like zinc metallopeptidases, found on the outer surface of animal cells, that negatively regulate small signalling peptides (e.g., enkephalin, tachykinin, insulin, and natriuretic peptides) by cleaving them; Y116A8C.4 has no clear orthologs in other organisms.
WBGene00013879		ZC376.8			
WBGene00013886		ZC412.5			
WBGene00013956		ZK265.3			
WBGene00014116		ZK858.2			
WBGene00014127		ZK892.3			
WBGene00014154		ZK930.4			
WBGene00014168		ZK945.6			
WBGene00014169		ZK945.7			
WBGene00014179		ZK1010.5			
WBGene00014197		ZK1053.2			
WBGene00014238		ZK1225.4			
WBGene00014241		ZK1251.3			

WBGene00015034		B0207.11			B0207.11 encodes a nematode-specific protein probably required for a normally high ovulation rate; B0207.11 has no obvious non-nematode homologs, but does have a putative N-terminal coiled-coil domain and an SH2 motif, and is is paralogous to four other C. elegans proteins (F42G4.6, F44F4.10, T08G11.2, and Y81G3A.1); B0207.11(tm322) hermaphrodites show abnormal egg-laying, retaining significantly fewer eggs than wild-type (perhaps due to a lowered ovulation rate) while retaining late-stage embryos; B0207.11 has no obvious phenotype in mass RNAi experiments, possibly because of genetic redundancy with its paralogs.
WBGene00015051		B0218.7			
WBGene00015094		B0261.6			
WBGene00015097		B0273.1			
WBGene00015192		B0432.11			
WBGene00015241		B0524.2			
WBGene00015345		C02F5.2			
WBGene00015348		C02F5.5			
WBGene00015467	basl-1	C05D2.3			C05D2.3 generally resembles aromatic-L-amino-acid/L-histidine decarboxylases; however, its predicted protein sequence lacks six residues critical for AADC function.
WBGene00015627		C09B9.2			
WBGene00015689		C10G11.9			
WBGene00015690		C10G11.10			
WBGene00015765		C14C11.1			
WBGene00015820		C16A11.7			
WBGene00015937		C17H12.12			
WBGene00015944		C18A3.7			
WBGene00015987		C18G1.9			
WBGene00016054		C24D10.2			
WBGene00016058	nspd-3	C24D10.7			
WBGene00016161		C27D6.3			
WBGene00016181		C28C12.11			
WBGene00016212		C29F5.3			C29F5.3 encodes a cytidine deaminase; by homology the product of C29F5.3 is predicted to function in deamination of cytidine to uridine.
WBGene00016288		C31H1.5			
WBGene00016322		C32E8.4			
WBGene00016351		C33F10.1			
WBGene00016414		C34F11.2			

WBGene00016440	gipc-1	C35D10.2			
WBGene00016512		C38C3.3			
WBGene00016612		C43G2.3			
WBGene00016707		C46E10.1			
WBGene00016752		C48E7.7			
WBGene00016807		C50D2.3			
WBGene00016825		C50E10.1			
WBGene00016843		C50F7.3			
WBGene00016996		D1005.2			
WBGene00017026		D1037.5			D1037.5 encodes a phospholipase A2.
WBGene00017058		D2062.6			
WBGene00017112		E03H12.5			
WBGene00017175	irld-3	F02C9.4			
WBGene00017215		F07F6.1			
WBGene00017279		F09C12.8			F09C12.8 encodes an ortholog, predicted to be catalytically inactive, of Bombyx mori ecdysteroid phosphate phosphatase (EPP), human STS1 (OMIM:609201) and human UBASH3A (OMIM:605736); F09C12.8 is also paralogous to C. elegans C52E4.7, F53B6.7, F55A11.11, and T07F12.1.
WBGene00017384		F11G11.4			
WBGene00017386	nspd-5	F11G11.8			
WBGene00017387	mpst-4	F11G11.9			
WBGene00017542		F17E9.5			F17E9.5 encodes a novel protein conserved amongst nematodes.
WBGene00017550	nep-6	F18A12.1			F18A12.1 encodes a neprilysin; neprilysins are thermolysin-like zinc metallopeptidases, found on the outer surface of animal cells, that negatively regulate small signalling peptides (e.g., enkephalin, tachykinin, insulin, and natriuretic peptides) by cleaving them; F18A12.1 has no clear orthologs in other organisms.

WBGene00017553	nep-8	F18A12.4			F18A12.4 encodes a neprilysin; neprilysins are thermolysin-like zinc metallopeptidases, found on the outer surface of animal cells, that negatively regulate small signalling peptides (e.g., enkephalin, tachykinin, insulin, and natriuretic peptides) by cleaving them; F18A12.4 has no clear orthologs in other organisms.
WBGene00017851		F27C1.1			
WBGene00017853		F27C1.3			
WBGene00017902		F28E10.4			
WBGene00017910		F28H1.5			
WBGene00017955		F31E8.5			
WBGene00018000		F33D11.1			
WBGene00018001		F33D11.2			
WBGene00018081		F36A4.2			
WBGene00018083		F36A4.4			
WBGene00018087		F36D4.1			
WBGene00018120		F36H12.4			
WBGene00018125	rmd-4	F36H12.11			
WBGene00018134		F37A4.4			
WBGene00018158		F37E3.3			
WBGene00018163		F38A5.6			
WBGene00018196	nep-13	F39E9.4			F39E9.4 encodes a neprilysin; neprilysins are thermolysin-like zinc metallopeptidases, found on the outer surface of animal cells, that negatively regulate small signalling peptides (e.g., enkephalin, tachykinin, insulin, and natriuretic peptides) by cleaving them; F39E9.4 has no clear orthologs in other organisms.
WBGene00018360	irld-8	F42G8.9			
WBGene00018497		F46F5.6			
WBGene00018548	clec-79	F47C12.4			
WBGene00018563		F47D12.7			
WBGene00018792		F54C1.8			
WBGene00018926		F56A11.6			
WBGene00018930		F56B3.6			
WBGene00019024		F58A6.5			
WBGene00019084		F59A6.2			

WBGene00019151	pck-3	H04M03.1			H04M03.1 is one of three C. elegans genes that encodes proteins with similarity to phosphoenolpyruvate carboxykinase (PEPCK); by similarity, the products of H04M03.1 are predicted to function in gluconeogenesis.
WBGene00019255		H32C10.1			
WBGene00019260		H34I24.1			
WBGene00019406	acdh-8	K05F1.3			
WBGene00019430		K06A5.2			
WBGene00019561		K09C6.7			
WBGene00019785		M70.3			
WBGene00019810		R01H2.2			
WBGene00020105		R148.7			
WBGene00020293	nep-20	T06D4.4			T06D4.4 encodes a neprilysin; neprilysins are thermolysin-like zinc metallopeptidases, found on the outer surface of animal cells, that negatively regulate small signalling peptides (e.g., enkephalin, tachykinin, insulin, and natriuretic peptides) by cleaving them; T06D4.4 has no clear orthologs in other organisms.
WBGene00020353		T08B6.4			
WBGene00020414		T10E9.4			
WBGene00020533		T16A1.2			
WBGene00020713		T23B3.5			
WBGene00020715	nspd-4	T23B7.1			
WBGene00020905		T28H11.7			
WBGene00020940		W02D7.4			
WBGene00020986		W03D8.3			
WBGene00020990		W03D8.9			
WBGene00021010		W03G1.2			
WBGene00021386		Y37F4.5			
WBGene00021398		Y38C1AA.7			
WBGene00021472		Y39G10AR.16			
WBGene00021579	clec-73	Y46C8AL.1			
WBGene00021608		Y46H3D.1			
WBGene00021633		Y47G6A.3			
WBGene00021650		Y47G6A.26			
WBGene00021720		Y49F6B.8			
WBGene00021787		Y51H7C.9			
WBGene00021878		Y54G2A.13			
WBGene00021880		Y54G2A.15			
WBGene00021969		Y57G7A.6			
WBGene00021996		Y59E9AL.6			
WBGene00022162		Y71G12B.27			

WBGene00022288		Y75B7B.1			
WBGene00022385		Y95B8A.4			
WBGene00022467		Y119C1B.1			
WBGene00022530		ZC155.2			
WBGene00022650		ZK84.2			
WBGene00022689	math-48	ZK250.6			
WBGene00022700		ZK353.4			
WBGene00022761		ZK546.4			
WBGene00022771		ZK616.1			
WBGene00022849	acs-6	ZK1127.2			
WBGene00023424		C53D6.10			
WBGene00044674		B0280.17			
WBGene00044684		T08G11.2			T08G11.2 encodes a nematode-specific sperm protein, required for a normally high ovulation rate, that interacts with EGL-32; despite not being EGL-32, and although T08G11.2(tm336) complements egl-32(n155), T08G11.2 can partially rescue egl-32 mutants when expressed transgenically, implying that T08G11.2 can be a nonorthologous replacement for EGL-32 function in vivo; T08G11.2 has an SH2 motif, but this motif lacks a critical arginine residue; T08G11.2 has no obvious non-nematode homologs, but is paralogous to four other C. elegans proteins (B0207.11, F42G4.6, F44F4.10, and Y81G3A.1); T08G11.2(tm336) hermaphrodites have a lowered ovulation rate (and thus a lowered rate of egg-laying), retaining significantly fewer eggs than wild-type, yet also retaining late-stage embryos; T08G11.2 has no obvious phenotype in mass RNAi experiments, possibly because of genetic redundancy with its paralogs; T08G11.2 expression is strongly enriched in spermatogenesis.
WBGene00045306		ZC250.5			
WBGene00045484		F34D10.9			
