# Supplementary material for: Transcription factors CEP‐1/p53 and CEH‐23 collaborate with AAK‐2/AMPK to modulate longevity in Caenorhabditis elegans
Source: Aging Cell. 2017 May 30;16(4):814–24. doi: 10.1111/acel.12619 (PMC5506430; doi:10.1111/acel.12619)
Supplement: Supplementary file 9 — Data S1 Supplemental experimental procedures. [file ACEL-16-814-s009.docx]

**Supplemental experiment procedure.**

**Microarray analysis.** Raw microarray data were normalized using Agilent feature extraction software. The normalized data were uploaded onto to the Princeton University MicroArray database (PUMA [http://puma.princeton.edu]). The raw data were retrieved by SUID (Sequence Unique IDentifier) then averaged by wormbase ID. Log2-transformed fold-changes were acquired after filtering out genes with <80% good data. The data were analyzed and visualized using Cluster 3 and Tree View (Saldanha 2004; Tusher et al. 2001). Gene sets that were regulated by CEH-23 in the *isp-1(qm150)* mutant were determined by SAM 1 class analysis (Tusher et al. 2001)with a FDR=0.59% and 1.5 fold gene cutoff. Ten genes were randomly selected to validate the quality of this gene list using RT-qPCR. Similar 1 class analysis was performed with stringent criteria (FDR=0% with 1.5 fold gene cutoff) using the *isp-1 vs. cep-1; isp-1* and *isp-1 vs. ceh-23; isp-1* datasets to identify genes that are commonly regulated by CEH-23 and CEP-1 under ETC stress. We identified 916 common targets of CEH-23 and CEP-1 (Supplementary Table S3) from this analysis. genome-wide CA-AAK-2 vs. wildtype microarray data were obtained from GEO (GSE25513) and 1 class SAM analysis was used to identify the genes that show significant changes in gene expression in CA-AAK-2 animals compare to wildtype. Genes that are common regulated by CEH-23, CEP-1 and AMPK represent the overlap between the CEH-23 and CEP-1 commonly regulated gene list and the CA-AAK-2 regulated gene list described above.

**Gene Ontology classification**. Gene sets identified by SAM analysis were input into the Functional annotation clustering tool in DAVID (http://david.abcc.ncifcrf.gov/) (Huang et al. 2009) for gene annotation enrichment analysis. Functional annotation clustering was performed with the default setting. Annotated clusters with enrichment scores were reported from this analysis. Kinases and phosphatases classification were based on GO term molecular function.

**Statistical significance of overlap between gene lists.** The statistical significance between CEH-23 and CEP-1 common target genes and AAK-2 CA targets or ROS-responsive targets were determined using the web-based tool (http://nemates.org/MA/progs/overlap_stats.html). A representation factor and p-value (determined by hypergeometric probability test) were reported.
